# Accelerated Molecular Dynamics for Peptide Folding: Benchmarking Different Combinations of Force Fields and Explicit Solvent Models

**DOI:** 10.1021/acs.jcim.3c00138

**Published:** 2023-05-10

**Authors:** Crescenzo Coppa, Andrea Bazzoli, Maral Barkhordari, Alessandro Contini

**Affiliations:** Dipartimento di Scienze Farmaceutiche − Sezione di Chimica Generale e Organica “Alessandro Marchesini”, Università degli Studi di Milano, Via Venezian, 21, 20133 Milano, Italy

## Abstract

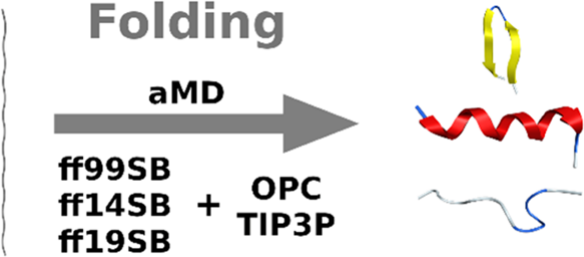

Accelerated molecular dynamics (aMD) protocols were assessed on predicting the secondary structure of eight peptides, of which two are helical, three are β-hairpins, and three are disordered. Protocols consisted of combinations of three force fields (ff99SB, ff14SB, ff19SB) and two explicit solvation models (TIP3P and OPC), and were evaluated in two independent aMD simulations, one starting from an extended conformation, the other starting from a misfolded conformation. The results of these analyses indicate that all three combinations performed well on helical peptides. As for β-hairpins, ff19SB performed well with both solvation methods, with a slight preference for the TIP3P solvation model, even though performance was dependent on both peptide sequence and initial conformation. The ff19SB/OPC combination had the best performance on intrinsically disordered peptides. In general, ff14SB/TIP3P suffered the strongest helical bias.

## Introduction

Several biological processes, such as signal transduction, growth, proliferation, differentiation, and apoptosis, involve protein–protein interactions (PPIs) that are established by secondary structure motifs at the protein–protein interface.^1−3^ Thus, recent years have witnessed a growing interest in biochemical tools to modulate PPIs.^4−6^ Among these tools, both natural and non-natural peptides represent an opportunity due to the ease of synthesis and biocompatibility.^7,8^ For this reason, efforts have been made to develop computational methods for predicting the secondary structure of peptides.^9−13^ Due to computer hardware improvements in the last decades, and thanks to the use of enhanced sampling methods that improve the exploration of conformational space,^14−17^ peptide PPI modulators can be efficiently designed *in silico*.

Accelerated molecular dynamics (aMD) is a modern enhanced sampling technique that proved to be able to reproduce the folding behavior of peptides.^17^ Indeed, 500 ns of aMD simulations provided a sampling power comparable to 1 ms of a classical MD simulation for the bovine pancreatic trypsin inhibitor.^18^ Tyagi and colleagues performed four aMD simulations,^19^ changing simulation time and boost parameters, to evaluate Alamethicin F30/3 folding behavior. The first three simulations were consecutive: the first starting from an unfolded conformation with low boost parameter values; the second starting from a folded conformation obtained from the first simulation and increasing the boost values; and the third one by starting from a conformation very similar to the native one obtained from simulation 2 and using the same boost of the second simulation. The fourth simulation was run starting from the same unfolded conformation used in the first one and using the boost values of the second one. As a result, they proved that the first three simulations (∼900 ns each), if combined in a meta-trajectory, were able to obtain the same result obtained from the fourth simulation (∼1 μs long). Both the meta-trajectory and the 1 μs long simulation were able to fold the peptide in the native conformation, but the second provided a faster convergence due to the optimized boost parameters. In the same year, Duan et al.^20^ studied the ability of aMD and ff14SB in folding eight helical proteins. In this case, aMD simulations were able to correctly fold these proteins, starting from an extended conformation, in a few nanoseconds (from 54 to 196 ns). However, the choice of a suitable force field is still critical for a correct prediction of peptide secondary structure.^21−23^ Indeed, force fields have been constantly upgraded by changing a few parameters derived from more accurate quantomechanical calculations. Most of the force field comparisons already performed focused on the ability of simulated peptides to fold into helices or β-hairpins,^24−29^ while few tests considered intrinsically disordered (ID) proteins also.^30−33^ In a previous work, we compared different force fields and implicit water models by using Temperature Replica Exchange Molecular Dynamics (T-REMD) simulations.^34^ We found that no combination correctly described all three classes of peptides (α-helices, β-hairpins, and ID), as also observed by others later.^35^ Indeed, using explicit solvent might be necessary to achieve optimal results. Additionally, T-REMD is rather expensive, compared to aMD, and explicit solvent simulations make T-REMD still prohibitive on standard hardware.^36,37^ In this work, we tested aMD simulations with combinations of three AMBER force fields (ff99SB,^38,39^ ff14SB,^40^ ff19SB^41^) and two explicit solvent models (TIP3P^42^ and OPC^43^) on reproducing the secondary structure of two α-helices, three β-hairpins, and three ID peptides. The same benchmark set used previously^34^ was chosen here to provide a direct comparison of results. We have narrowed it down to the three most modern Amber force fields due to their popularity and to better highlight any advances or setbacks made during their development. Moreover, we were interested in evaluating how the two different solvent models could dampen or enhance the force field bias, if any, forcing the folding toward a particular secondary structure.

The eight peptides used in this work, H1, H2, B1, B2, B3, ID1, ID2, and ID3, were selected due to their known native structures (Table 1). H1 is the QK VEGF modulator, and its helicity was proven by both CD and NMR.^44^ H2 (Ac-Ala-Aib-Ala-Aib-Ala-NHMe) is also helical,^45^ but its structure was resolved by X-ray experiments. B1, B2, and B3 have a β-hairpin structure. B1 is the C-terminal sequence of the streptococcal protein G, whose structure was solved by NMR (PDB entry 2GB1). The structure of the B1 isolated peptide was also evaluated in solution by NMR, and in this case, a β-hairpin population of about 40% was found.^46^ B2 is the trpzip2 tryptophan zipper, and its structure was also obtained by NMR in water (PDB entry 1LE1).^47^ B3 is the N-terminus of ubiquitin, whose structure has been solved by X-ray (PDB entry 1UBQ).^48^ ID1 is the Polybia-MPII sequence,^49^ whose ID geometry was evidenced by CD experiments. ID2 and ID3 correspond to the TRTK-12 CapZ peptide,^50^ and p53’s C-terminal sequence,^51,52^ respectively, and their secondary structure was solved by X-ray experiments^44−48^ (PDB entries 1MWN(50) and 1DT7, respectively^51,52^).

**Table 1:** Peptides Used in This Study

peptide	sequence	secondary structure	experimental data	reference
H1	ACE-KLTWQELYQLKYKGI-NHE	helix	CD (water, 20 °C, pH 7.1)	(44)
H2	ACE-Ala-Aib-Ala-Aib-Aib-NHE	3_10_-helix	X-ray	(45)
B1	ACE-GEWTYDDATKTFTVTE-NHE	β-hairpin	NMR (H_2_O/10% D_2_O, pH 6.3)	(46)
B2	ACE-SWTWENGKWTWK-NHE	β-hairpin	NMR (H_2_O/8% D_2_O, pH 5.5)	(47)
B3	ACE-QIFVKTLTGKTITLE-NHE	β-hairpin	X-ray	(48)
ID1	ACE-INWLKLGKMVIDAL-NHE	ID	CD (water, 25 °C)	(49)
ID2	ACE-TRTKIDWNKILS-NHE	ID	NMR (H_2_O/10% D_2_O, pH 7.2)	(50)
ID3	ACE-STSRHKKLMTKTE-NHE	ID	NMR (D_2_O, 37 °C)	(51, 52)

## Methods

Each peptide was capped with acetyl (ACE) and amino (NHE) groups. Two independent aMD runs were performed on each tested peptide starting from an extended (ψ = φ = ω = 180°) or a misfolded conformation (i.e., α-helix for B1–3 and ID1–3, β-hairpin for H1 and H2), hereafter referred as “extended” or “misfolded” simulation, respectively. The topological and the starting coordinates files were generated with *tLEaP*,^53^ and parameters of the non-natural amino acid α-aminoisobutyric acid (Aib), included in H2, were downloaded from the RED database^54^ and used with ff99SB, ff14SB, or ff19SB force fields, or taken from ff15ipq-m when using it.^55^ 20-ns-long cMD simulations, using the isothermal isobaric ensemble (NPT), were run to obtain the average dihedral and potential energies needed to derive aMD boost parameters (Tables S1 and S2). Overall, 1500 ns were simulated by each aMD run. The aMD simulations were run in NPT (Langevin thermostat, Berendsen barostat), with a timestep of 2 fs saving 750 000 frames (ntwx = 1000). The cutoff for nonbonded interactions was set to 8 Å; beyond this value, long-range interactions were calculated by particle mesh Ewald. SHAKE algorithm was activated to constrain bonds involving hydrogen. Convergence of trajectories was evaluated by time-dependent RMSD and DSSP analyses. RMSD was evaluated by using the native structure of H2, B1, B2, B3, ID2, and ID3 as the reference. For H1 and ID1, the main cluster of the ff14SB/TIP3P extended simulation, calculated on the last 500 ns, was used as the reference due to its well-structured α-helical conformation (Figures S1–S8). DSSP components were instead calculated every 250 ns (Figures S9–S16). Most of the simulations were converged in the last 500 ns of the simulations. The B2 misfolded simulation using ff19SB/TIP3P combination is an exception since it converged into a stable β-sheet structure between 600 and 1100 ns, as shown in Figure S17. Consequently, trajectory analyses were generally conducted with *cpptraj* on the last 500 ns portion of the simulations,^56^ while the analyses on the B2 misfolded simulation were conducted both on the 1000–1500 ns and on the 600–1100 ns interval of the aMD trajectory. Cluster analyses were performed on Cα atoms by sampling one every 10 frames, using the average-linkage algorithm, and requesting 10 clusters. RMSDs were calculated by superimposing backbone atoms (C, O, Cα, N, H) of a representative conformation of the main or the second cluster with the native structure. H-bonds involving backbone N–H and C=O were computed with *cpptraj* using default settings. Secondary structure analyses were conducted by the Define Secondary Structure of Proteins (DSSP) algorithm.^57^ The φ and ψ dihedral distributions were obtained by analyzing the aMD trajectories and compared to the corresponding reference values measured on the native structures. The aMD trajectories were reweighted using a 10th-order Maclaurin series expansion, and a discretization of 3 for both the X- and Y-dimensions, to compute accurate potentials of mean force (PMF). Bidimensional (2D) and tridimensional (3D) plots were generated using the corresponding *PyReweighting* scripts by Miao et al.,^58^ available at https://miaolab.ku.edu/PyReweighting/.

CD spectra were obtained by using Structure-Based Empirical Spectrum Calculation Algorithm (SESCA) developed by Grubmüller et al.,^59^ downloaded from https://www.mpinat.mpg.de/sesca. The CD spectra were calculated using the DS5-4 library optimized for loop structures, providing either the last 500 ns of the aMD trajectory (in PDB format, saving one every 10 frames of the original trajectory) or the main cluster of H1 and ID1 simulations as the input.

The ACE capping group was considered as residue 1 and NHE as the last residue in the data reported. Thus, the peptides are composed of 17 (H1), 7 (H2), 18 (B1), 14 (B2), 17 (B3), 16 (ID1), 14 (ID2), and 15 (ID3) amino acids, respectively.

## Results

We focused on the ability of three force fields, ff99SB, ff14SB, and ff19SB, combined with two solvation models, TIP3P and OPC, to reproduce the native structure of eight peptides, H1, H2, B1, B2, B3, ID1, ID2, and ID3 (Table 1). Additionally, we assessed whether the well-known helical bias of recent force fields^60−62^ was somehow dampened in the newer versions.

Both the main cluster representative conformation and the group of conformations generated in the last 500 ns of the simulations were considered to evaluate the correct folding of peptides. The main cluster and the ϕ and ψ values of the lowest energy conformation were indeed compared to the native structures for both the structured and ID peptides. Moreover, the behavior of the peptides was also studied considering the DSSP component along all of the analyzed trajectories for both structured and ID peptides, and by evaluating the frequency distribution of the radius of gyration values during the same period of time for ID peptides alone. Finally, CD spectra for H1 and ID1 were calculated on the last 500 ns of the aMD trajectory and on the main cluster conformations, and compared to experiments.

### Helical Peptides

#### H1

**Figure 1:**
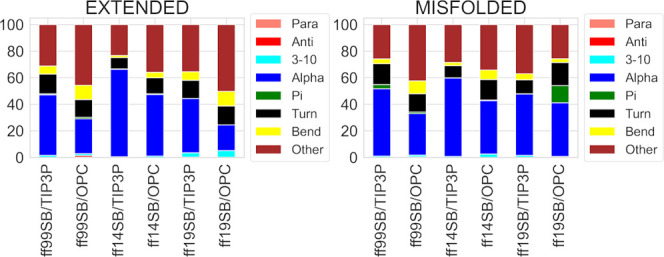
DSSP analysis of H1 trajectories from extended and misfolded simulations. Values are expressed as a percentage of the distributions of all of the residues considering the last 500 ns frames.

By looking at the DSSP results from the H1 simulations (Figure 1), the difference between the two solvation methods is clear, with TIP3P simulations showing higher percentages of α- and 3-10 helices, compared to OPC. Compared to the results reported for the same structure previously,^34^ ff99SB/TIP3P shows similar results to the ff99SB simulations in all of the implicit solvents, while ff14SB/TIP3P shows similar results to ff12SB in all of the implicit solvents. Conversely, the ff19SB/TIP3P and all of the OPC simulations considerably reduce the α-component in DSSP analyses (an α-component of 19.3% was obtained from the ff19SB/OPC extended simulation).^34^

To identify the lowest energy conformation, aMD trajectories were reweighted using φ and ψ angles as the reaction coordinates (Figures S18–S29). Results show that the lowest energy conformation of each simulation, as obtained from the PMF of dihedral distributions, presents φ and ψ angles in the α-helix region (Figures S18–S29). The β-region was poorly explored by all combinations involving the TIP3P. Conversely, the α-helix region was extensively sampled by all force fields. When using the OPC solvent, both the ff99SB and the ff14SB showed a wider exploration of the β-region. Surprisingly, an even weaker exploration of the β-region was obtained by ff19SB/OPC, compared to TIP3P. These data suggest that the OPC solvent might be able to dampen the helical bias in ff99SB and ff14SB, while improving ff19SB’s efficacy in identifying the correct conformation of residues.

**Figure 2:**
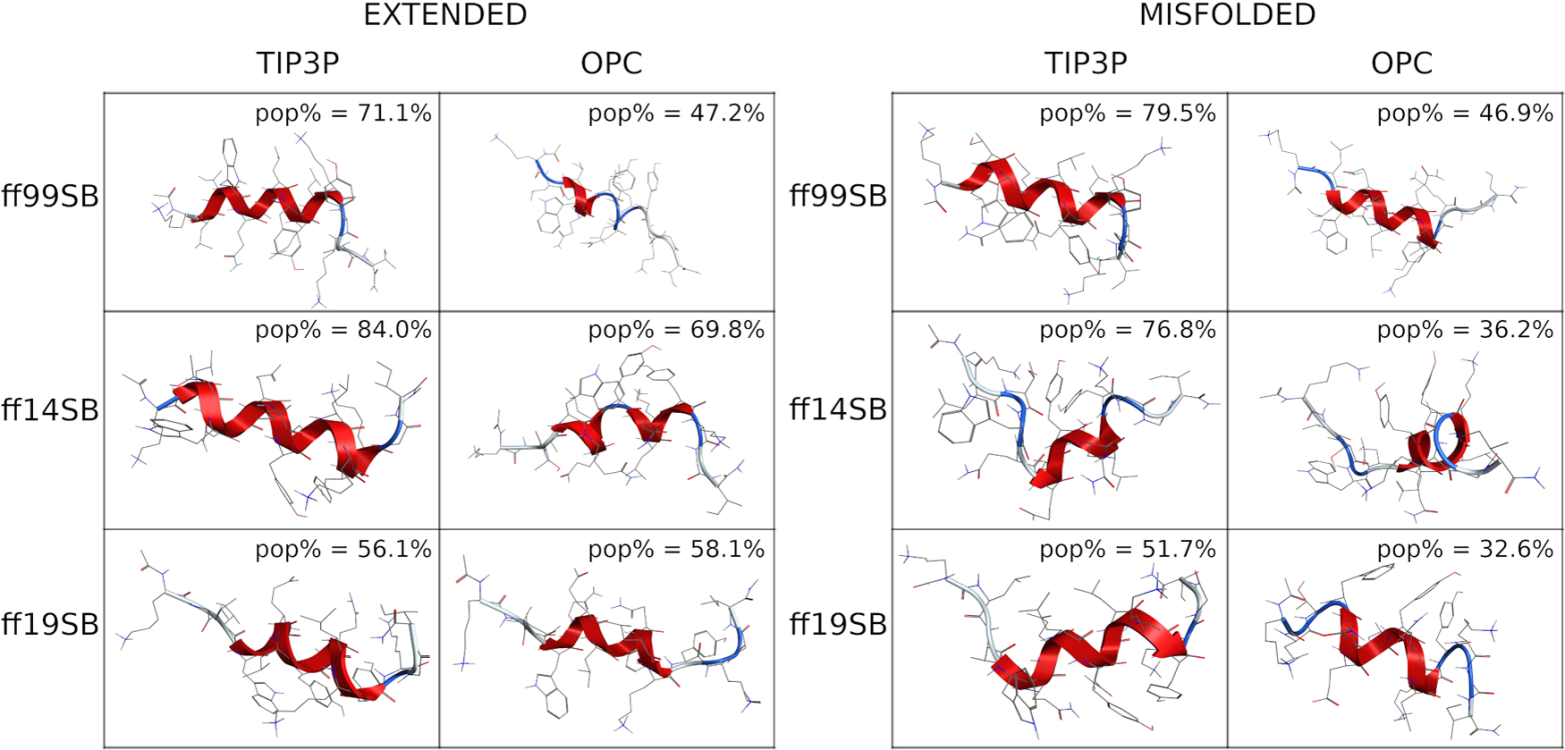
Representative conformation of the main cluster of H1. Clusters obtained from extended simulations are shown on the left; clusters obtained from misfolded simulations are shown on the right. The percentage of frames (pop%) comprising the cluster and the backbone RMSD between the representative conformation and the native conformation are also shown.

These findings are also supported by cluster analyses that show a larger population (pop%) of the main cluster for TIP3P runs, compared to OPC, except for the ff19SB extended run (Figures 2 and S30), thus indicating a reduction in the helical bias by OPC solvent. Moreover, the high pop% suggests that stable conformations were found during all the simulations, except for the ff14SB/OPC and ff19SB/OPC misfolded simulations. This aspect does not deny the possibility of forming helices for runs where the OPC solvation was used. In fact, H-bond analyses (Table S3) show, for every combination, the classical i → i + 4 or i → i + 3 backbone interactions typically formed in α- and 3-10-helical peptides, respectively. However, the occupancy of these interactions is generally higher for TIP3P than for OPC combinations (Table S3).

**Figure 3:**
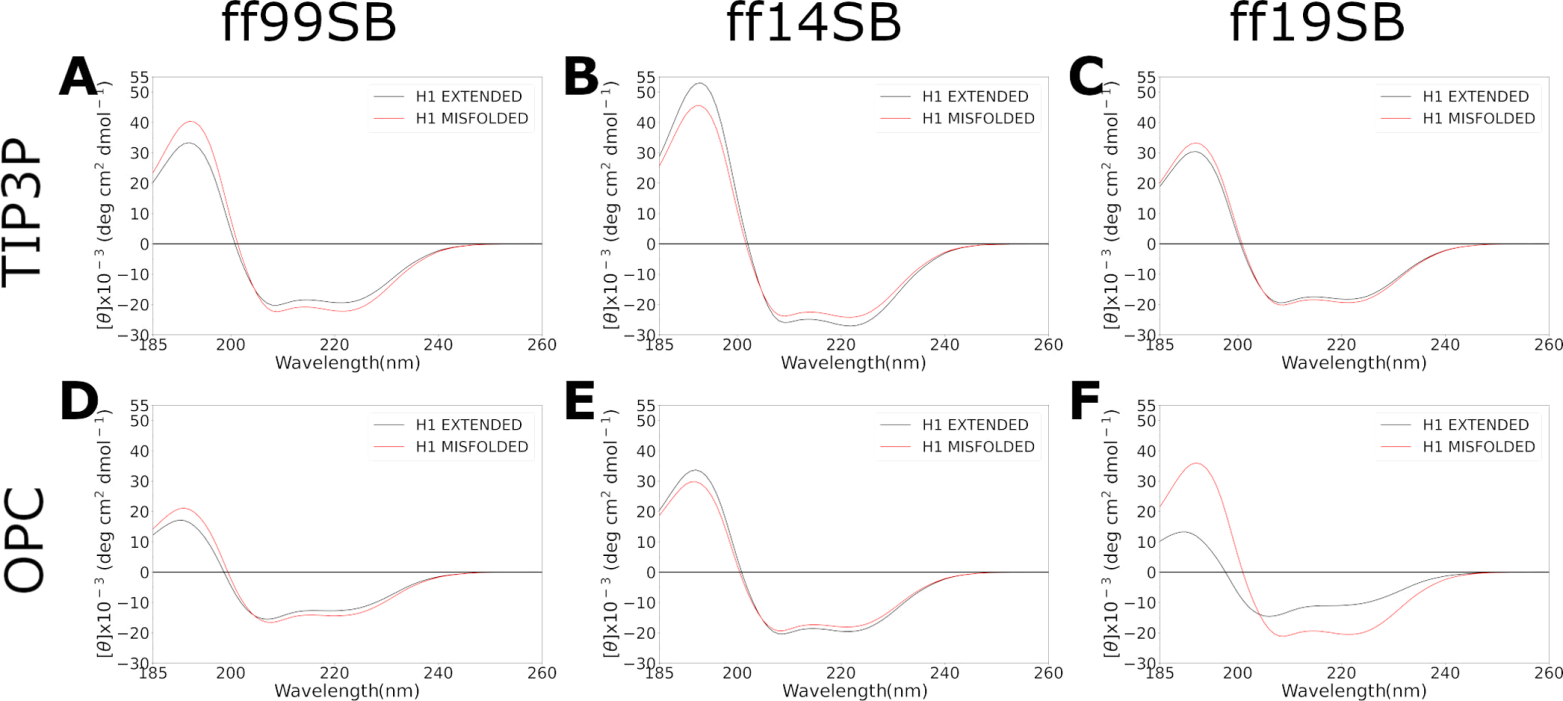
CD spectra of H1 simulations calculated on the last 500 ns frames for each combination using SESCA software. Results from extended and misfolded simulations are reported in black and red lines, respectively.

Unfortunately, the lack of an experimental 3D structure for H1 does not help to assess which combination better reproduces its native folding. However, since the experimental CD is available, CD spectra were calculated by using SESCA,^59^ an algorithm used to generate CD spectra from single structure or trajectories. As a result, spectra calculated from the last 500 ns of the simulations (Figure 3), as well as the ones calculated from the main clusters (Figure S31), showed that all of the combinations well reproduced the experimental CD spectrum that suggests an α-helix folding for the H1 peptide.^44^

These data, together with the results from DSSP (Figure 1), PMF (Figures S18–S29), and H-bond analyses (Table S3), as well as the main cluster conformations and corresponding populations (Figures 2 and S30), suggest ff14SB/TIP3P as the best combination to reproduce the α-helical folding of this peptide. However, similar results were obtained by the other combinations also.

#### H2

**Figure 4:**
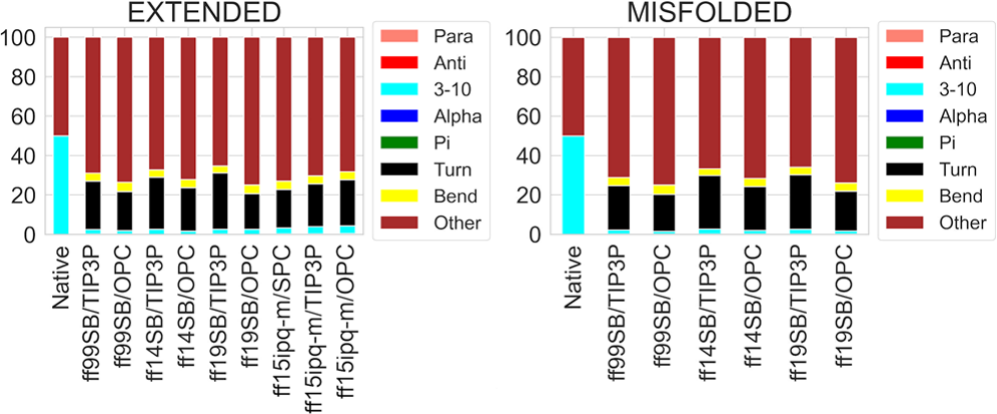
DSSP analysis of H2 trajectories from extended and misfolded simulations. Values are expressed as percentage of the distributions of all of the residues considering the last 500 ns frames.

H2 is a 5-residue peptide containing Ala, and three Aib residues at positions 2, 4, and 5. As expected for a short Aib-containing peptide, its native X-ray structure is a 3-10 helix.^45^ The secondary structure assignments obtained from the DSSP analysis of each simulation were compared to those calculated on the native structure (Figure 4), but no combination was able to reproduce the native DSSP distribution. In this case, TIP3P solvation appears to reduce helicity, as TIP3P’s 3-10 percentages are lower than the OPC ones for misfolded simulations (except for ff19SB, as shown in Figure 4), vice versa for extended simulations (except for the ff19SB/OPC extended simulation, that presents values similar to those of TIP3P).

Moreover, compared to the previous work, the explicit solvation model decreased the 3-10 and zeroed the α component, while increasing the disordered component (“other”, in Figure 4) by about 30%.^34^ From these data, it seems that all the implicit solvent models used in the previous work in combination with all the tested force fields, except for ff96, work better than the explicit ones for this peptide. However, it should be considered that the native structure considered herein derives from X-ray, and the peptide might behave differently in water. Indeed, Schweitzer-Stenner et al. observed a predominance of extended conformations for Ac-Ala-Ala-Aib-OMe and Ac-Ala-Aib-Ala-OMe peptides by performing infrared, isotropic Raman, anisotropic Raman, and vibrational circular dichroism analyses in D_2_O solvent.^63^

**Figure 5:**
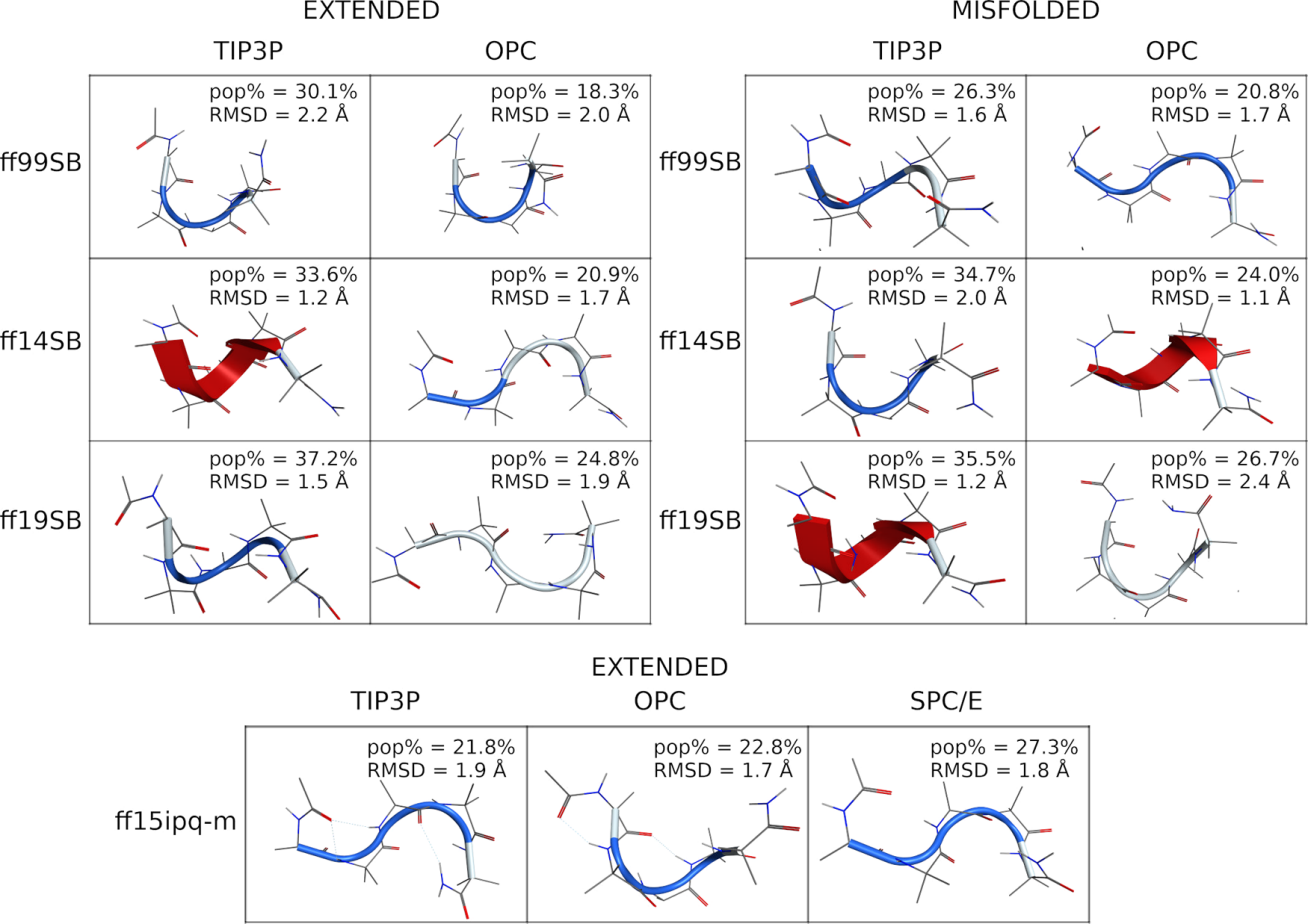
Representative conformation of the main cluster of H2. (Left) Clusters obtained from extended simulations; (right) clusters obtained from misfolded simulations; (bottom) clusters obtained from ff15ipq-m simulations. The percentage of frames (pop%) comprising the cluster and the backbone RMSD between the representative conformation and the native conformation are also shown.

PMFs obtained from the φ and ψ dihedral distributions of all the simulations (Figures S32–S37) show an almost identical behavior for all the combinations. Here, the lowest energy conformation of each residue was found within a region containing the corresponding φ and ψ dihedrals of the native X-ray structure (Figures S32–S37). The only exception is Ala4, where the native ψ angle lies in the upper border of the most sampled region, or slightly above. This discrepancy might be the reason for the highly frequent “other” assignment in the DSSP analysis discussed earlier. Cluster analyses (Figures 5 and S38) show that the representative 3D conformation of the main cluster of the ff99SB/TIP3P misfolded simulation, both the ff99SB/OPC, ff14SB and ff19SB/TIP3P simulations, and the ff19SB/OPC extended simulation, is very similar to the native X-ray conformation (RMSD ≤ 2 Å, Figure 5). However, a rather low pop% was found for the main cluster, suggesting that the sampling of the native X-ray conformation was either temporary or late in the simulation.

The ff14SB/OPC misfolded simulation provided the lowest RMSD (1.1 Å). Conversely, excluding the H-bonds involving capping groups, only the ff99SB/TIP3P and ff19SB/TIP3P misfolded simulations sampled the native interactions (Table S4). Additionally, while the TIP3P model generally favors helicity (or enhances the helical bias) in extended simulations, this is not true for the misfolded one. The discrepancy between the extended and misfolded simulations, as well as the odd behavior of the TIP3P and OPC solvent models, suggests that a 1.5 μs aMD simulation might not be long enough for this peptide, even if it is only 5 residues long. On the other hand, the Aib parameters used in these simulations might not be optimal. Thus, we repeated the simulations, starting from the extended conformation and using the ff15ipq-m force field,^55^ that contains specific parameters for Aib. The simulations were performed with OPC, TIP3P, or SPC/E,^64^ the water model used by the force field developers.^55^ Results show that neither the force field nor the solvation model improved the match of H2 to the X-ray structure. Indeed, anti and 3-10 components did not increase significantly (Figure 4). PMFs obtained from φ and ψ dihedral distributions show a behavior like the one obtained by the other combinations (Figures S39–S41). Moreover, the representative conformation of the main cluster shows RMSDs of 1.9, 1.7, and 1.8 Å to the native conformation, for the ff15ipq-m/TIP3P, ff15ipq-m/OPC, and ff15ipq-m/SPC/E combinations, respectively (Figure 5). Notably, ff15ipq-m/OPC is the only combination forming a native H-bond, excluding the ones involving caps (Table S4). Although these results seem to be in contrast to the reference X-ray structure, contrary to that observed using implicit solvation, they are in line with experiments done on similar peptides in solution.^63^

### β-Hairpin Peptides

#### B1

**Figure 6:**
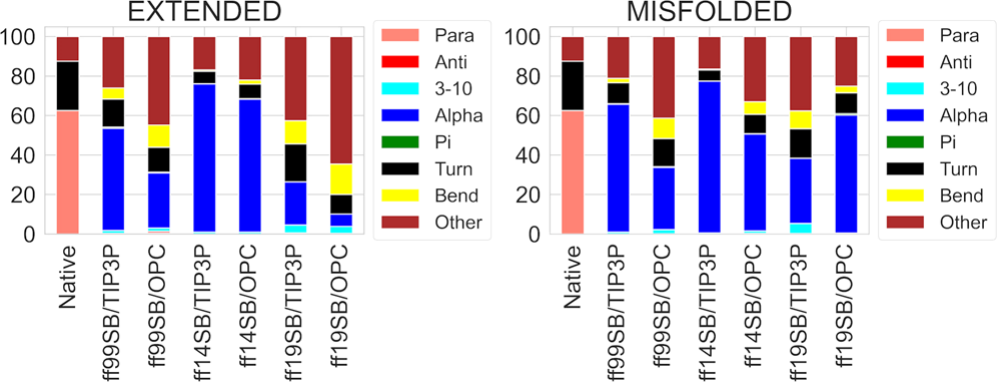
DSSP Analysis of B1 Trajectories from Extended and Misfolded Simulations. Values are expressed as a percentage of the distributions of all the residues considering the last 500 ns frames.

None of the combinations tested herein predicted the native conformation of B1, as previously observed for implicit solvent simulations where only the ff96/GB-HCT method predicted a correct folding for this peptide.^34^ Indeed, none of the calculated DSSP distributions reproduced the native one (Figure 6). The ff99SB/OPC and ff19SB/TIP3P combinations showed the lowest percentage of helical components and the highest para, anti, and turn conformations. Conversely, the ff99SB/TIP3P and ff14SB/TIP3P combinations, together with the ff19SB/OPC misfolded simulation, gave the highest helical components. In sharp contrast to the misfolded simulation, the ff19SB/OPC extended simulation provided the lowest α-helical component among all the simulations (Figure 6).

**Figure 7:**
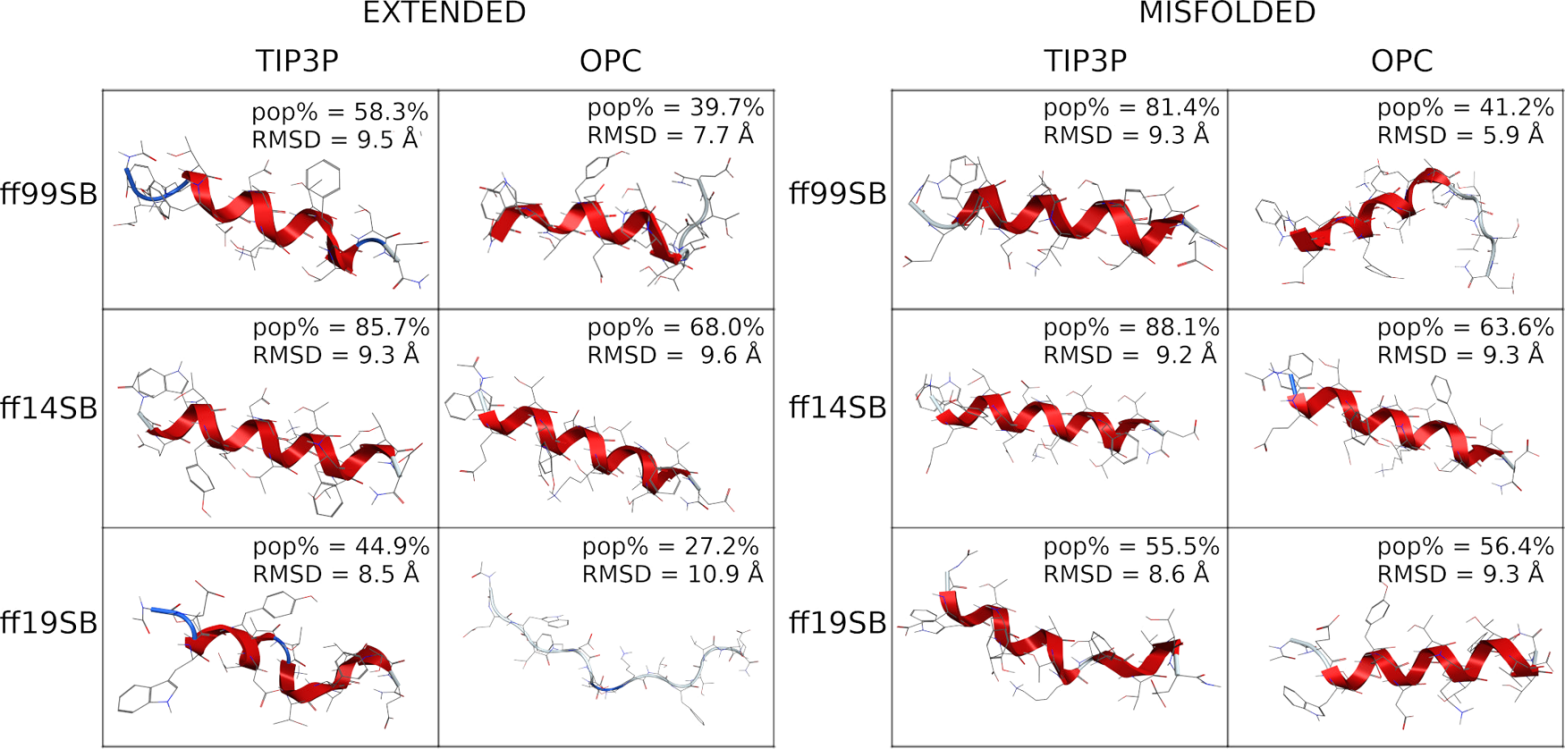
Representative conformation of the main cluster of B1. (Left) Clusters obtained from the extended simulation; (right) clusters obtained from the misfolded simulation. The percentage of frames (pop%) comprising the cluster and the backbone RMSD between the representative conformation and the native conformation are also shown.

PMFs obtained from φ and ψ dihedral distributions (Figures S42–S53) confirm the DSSP results. Only Thr10 had the global minimum at the native dihedrals in all combinations (Figures S42–S53). Among the TIP3P combinations, only ff19SB sampled all the correct dihedrals. On the other hand, all three force fields were able to explore the native dihedrals with the OPC model. Surprisingly, higher energy minima were found in the β-region by ff19SB, compared to the ff99SB and ff14SB combinations. This last aspect is better evidenced by cluster analyses (Figures 7 and S54). None of the main clusters reproduced the native structure (the lowest RMSD between the representative conformation of the main clusters and the native conformation is 5.9 Å, from the ff99SB/OPC misfolded run, as shown by Figure 7) and all the combinations predicted a helical secondary structure as the main conformation. A partial loss of helicity can only be seen in the second cluster of ff99SB/TIP3P, ff14SB/OPC, and ff19SB/TIP3P runs (Figure S54). The ff19SB/TIP3P combination provided the highest number of native H-bonds (Table S5), but none of the combinations reproduced the folding of this peptide overall.

At least for this example, although the ff19SB force field and the OPC solvent sometimes led to an improvement, the well-known helical bias affecting modern Amber force fields still appears to be an issue a quindecinnial after Best et al.’s article.^60^

To evaluate the effect of N- and C-terminus capping on the folding predictions, a second set of aMD simulations was performed on the uncapped peptide, starting from the extended conformation. The results were like those discussed above (Figures S55–S60), except for the ff99SB/TIP3P combination, that surprisingly provided the highest para component in DSSP calculation (15%, Figure S55). Additionally, most of the native dihedral angle and H-bonds were correctly sampled (Table S6 and Figures S56–S59) and a β-sheet-like structure was obtained as the most representative geometry of the main cluster (pop% = 53.5%, Figure S60), coherently with a 40% of β-hairpin structure detected by CD experiments.^46^

#### B2

**Figure 8:**
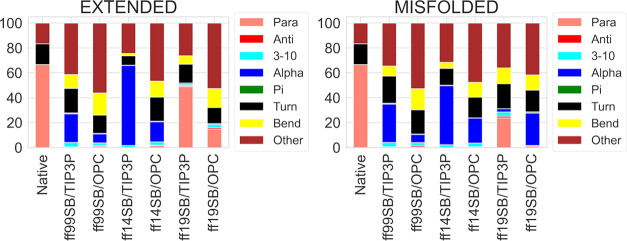
DSSP analysis of B2 trajectories from extended and misfolded simulations. Values are expressed as a percentage of the distributions of all of the residues considering the last 500 ns frames.

The folding behavior of the B2 β-hairpin peptide, rather stable in water as shown by NMR,^47^ was investigated. The ff19SB/TIP3P combination best reproduced the DSSP distribution of the native structure (Figure 8). The ff19SB/OPC extended simulation (Figure 8) partially reproduced the native DSSP distribution, but a lower similarity was observed compared to the TIP3P one. Among the other combinations, ff99SB/OPC is the only one that reduces the helical components, in favor of a disordered structure, while ff14SB/TIP3P is the one giving the highest helicity.

Compared to implicit solvation, where only ff96/GB-HTC was able to obtain a β-score higher than 11%,^34^ explicit solvents seem to perform better.

PMFs obtained from φ and ψ dihedral distributions show that all the combinations, except for ff14SB/TIP3P, explored the region of native φ and ψ (Figures S61–S66). However, ff19SB/TIP3P was the only one to recognize the native values as a global minimum. OPC runs show improved sampling with all the force fields, except the ff19SB.

**Figure 9:**
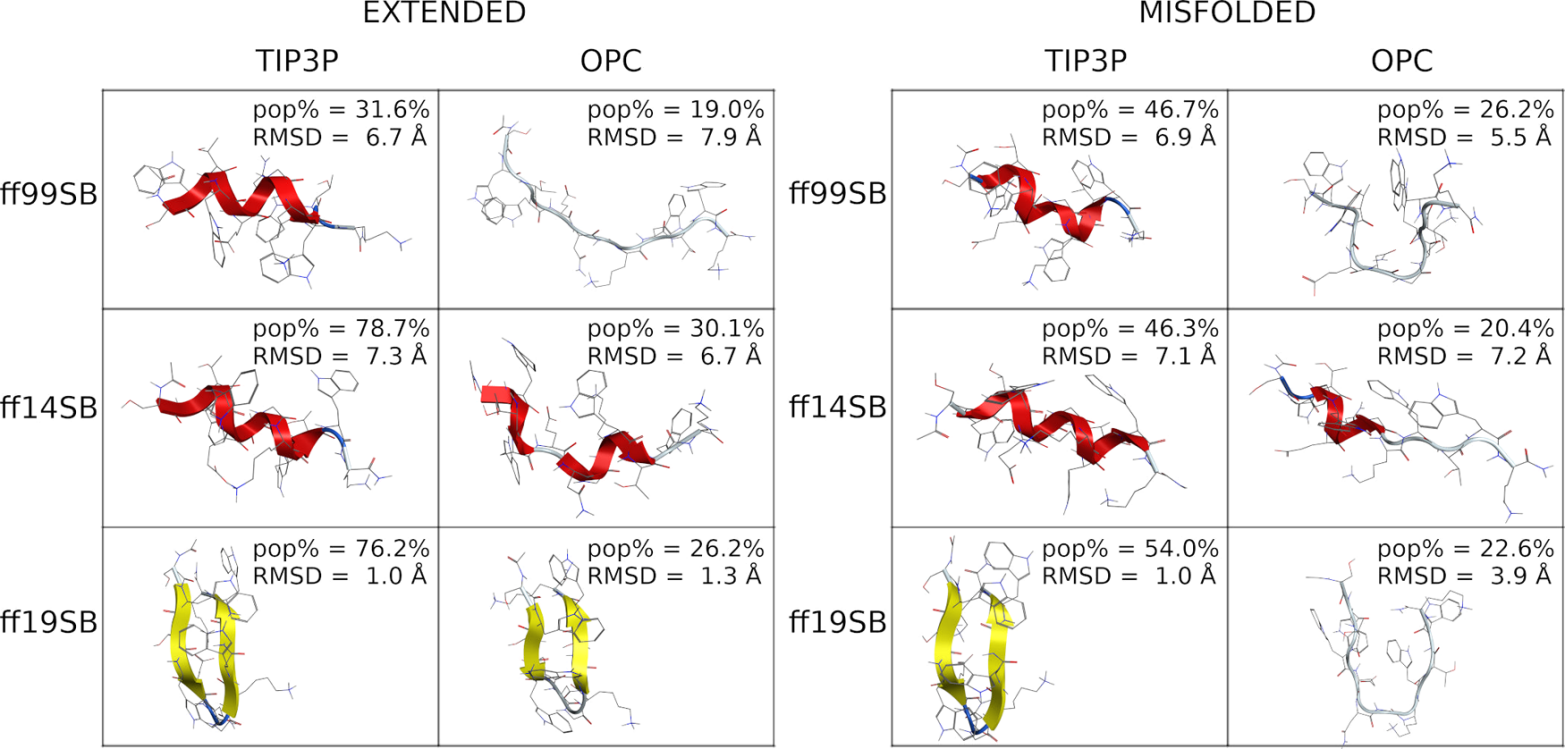
Representative conformation of the main cluster of B2. (Left) Clusters obtained from extended simulations; (right) clusters obtained from misfolded simulations. The percentage of frames (pop%) comprising the cluster and the backbone RMSD between the representative conformation and the native conformation are also shown.

Cluster analyses confirm the above results (Figures 9 and S67), since the ff19SB/TIP3P main cluster reproduces the native folding of the peptide in both the extended and misfolded simulations (the RMSD between the representative conformation of the main cluster and the native conformation is 1.0 Å for both runs), with pop% above 50% (76.2 and 54.0% for the extended and misfolded simulations, respectively). When using OPC solvation, only the extended ff19SB/OPC simulation was able to form the β-hairpin, with a low pop% (26.2%, Figure 9). Additionally, even if the ff99SB/OPC simulations were able to escape the helical conformation, their sampling concentrated away from the native conformation (RMSDs of 7.7 and 5.5 Å for extended and misfolded simulations, respectively). These results suggest that the use of OPC solvent coupled to ff19SB might slow down convergence for some systems. Finally, the H-bond analyses also showed that ff19SB/TIP3P is the best combination for reproducing the native contacts (Table S7). Once again, these data show a clear improvement over implicit solvent simulations, where only the combinations of ff96 and GB-HCT or GB-OBC(II) succeeded. However, ff96 predicted a hairpin or an extended conformation for helical peptides also.^34^

#### B3

**Figure 10:**
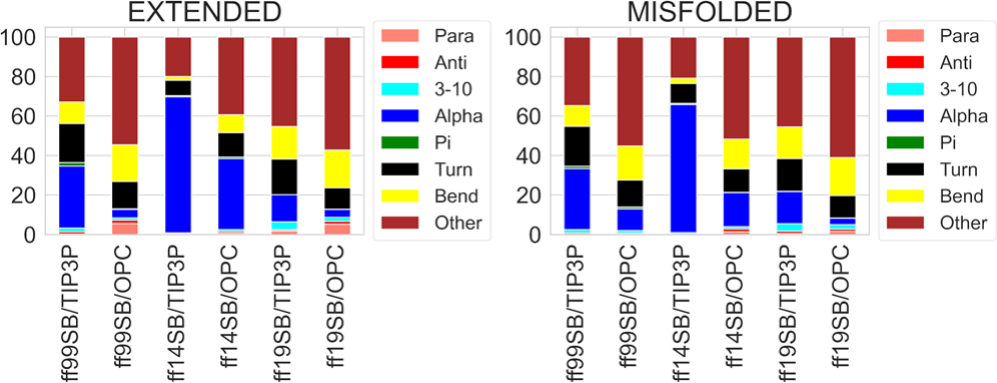
DSSP analysis of B3 trajectories from extended and misfolded simulations. Values are expressed as a percentage of the distributions of all the residues considering the last 500 ns frames.

The B3 β-hairpin is the N-terminal sequence of ubiquitin.^48^ The DSSP analysis shows that none of the combinations was able to match the native secondary structure (Figure 10). However, helicity was almost completely lost using the ff99SB/OPC (1.4 and 11.0% for 3-10 and α helices, respectively) and ff19SB/OPC (3-10 = 2.1% and α = 3.3%) combinations. Conversely, ff14SB with both TIP3P and OPC shows the highest values of helicity, as for the previous peptides. Implicit solvent ff99SB/GB-Neck2 simulations reproduced the native folding of this peptide better.^34^

PMFs obtained from φ and ψ dihedral distributions (Figures S68–S79) show that ff99SB and ff19SB sample a wide dihedral space (Figures S68–S71 and S76–S79), while ff14SB only explores the helical φ and ψ dihedrals (Figures S71–S75). The φ and ψ dihedrals sampled in the global minimum region match the native ones for Leu8 and Thr9 only (Figures S68–S79), while they generally fall in the α-helix region for the other residues. Once again, only ff99SB and ff19SB combined with TIP3P were able to sample the β-regions, but local minima close to the native conformation can only be observed for ff19SB. In this case, the OPC solvation method improves the sampling of the native dihedrals. However, the global minima fall within the right-handed α-helix region for all methods. These data underline once again the difficulty of these force fields in correctly folding the β secondary structures, even if improvements are observed for ff19SB and OPC over ff14SB and TIP3P, respectively.

**Figure 11:**
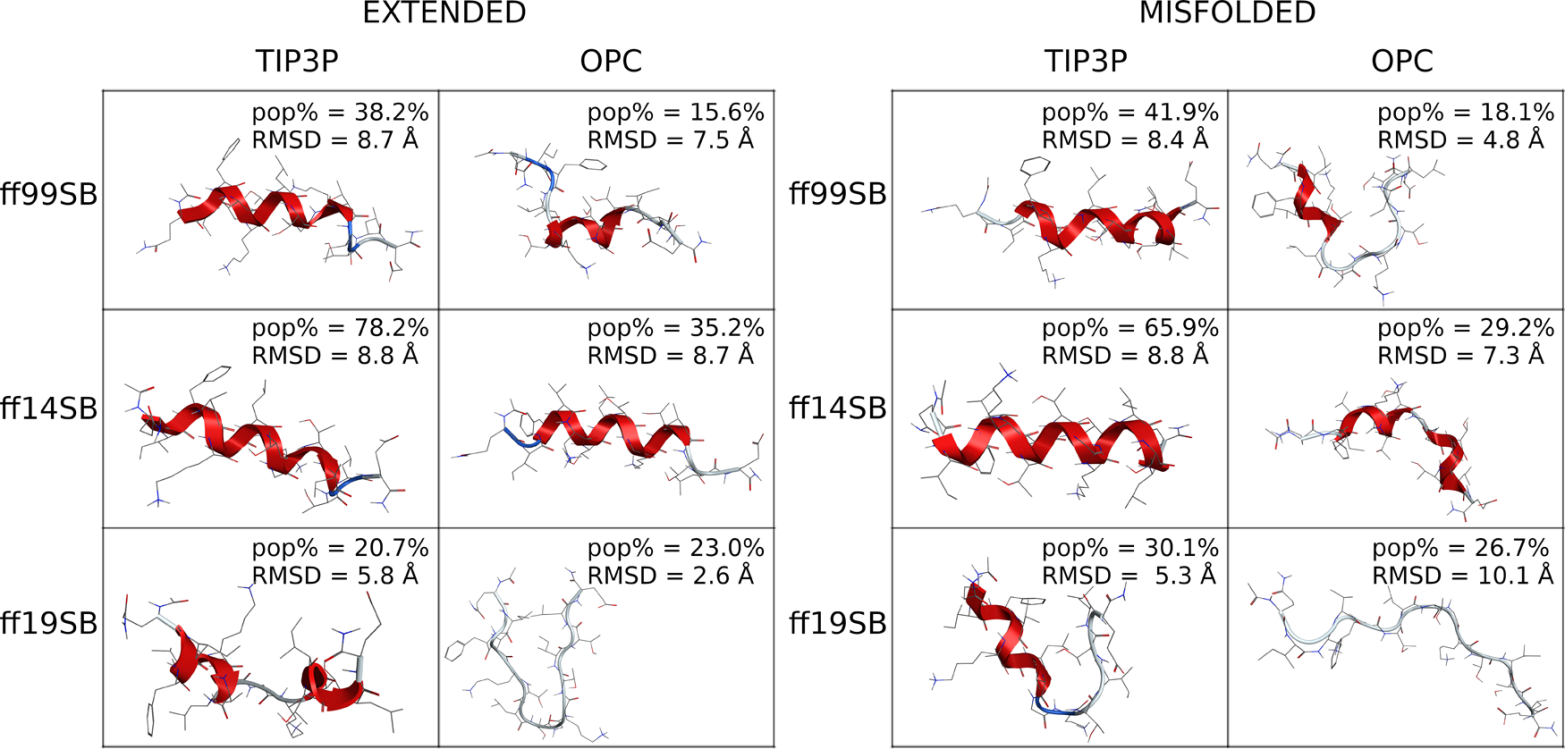
Representative conformation of the main cluster of B3. (Left) Clusters obtained from extended simulations; (right) clusters obtained from misfolded simulations. The percentage of frames (pop%) comprising the clusters and the backbone RMSD between the representative conformation and the native conformation are also shown.

This statement is strengthened by looking at the results of cluster analysis (Figures 11 and S80). None of the combinations was able to reproduce the native conformation within the most populated cluster. However, ff19SB/OPC provided the cluster representative closest to the native conformation (RMSD 2.6 Å), although with a rather low pop% (23.0%). Other combinations, except for ff99SB/OPC and ff19SB/TIP3P, provided α-helical structures (Figure 11) as confirmed by PMFs (Figures S68–S79). Surprisingly, the only native H-bond to be reproduced with a relevant occupancy was found between Thr7 and Lys11 by ff14SB/TIP3P simulations (occ% = 44.3 and 35.9 for extended and misfolded simulations, respectively; Table S8).

### Intrinsically Disordered Peptides

ID peptides are important in several biological processes, but predicting their structure is challenged by their flexibility.^65^ Thus, predicting the folding of ID peptides by MD simulations can lead to a better understanding of the biases affecting the evaluated force fields, as well as how their combination with explicit solvation models might reduce or worsen them.

#### ID1

**Figure 12:**
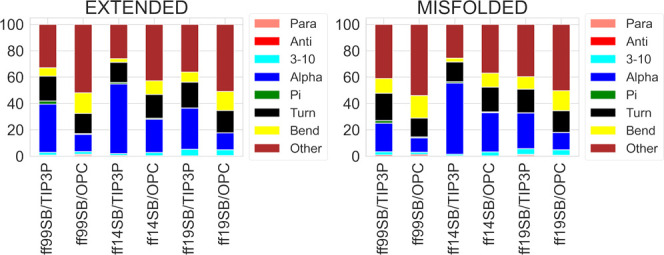
DSSP analysis of ID1 trajectories from extended and misfolded simulations. Values are expressed as a percentage of the distributions of all the residues considering the last 500 ns frames.

DSSP analyses of the ID1 trajectories show a tendency of the ff14SB/TIP3P combination to sample helical structures preferentially (54.0 and 52.9% of α-component for misfolded and extended simulations, respectively, Figure 12). The percentages of nonstructured portions of the peptides (“other” in Figure 12) are higher for the OPC than for the TIP3P model, at the expense of the helical content (Figure 12), as already observed for ff99SB.^31^

All the TIP3P runs, together with the ff14SB/OPC simulations, provided a disordered component like those obtained by implicit solvent simulations previously.^34^ However, ff99SB and ff19SB combined with OPC yielded a disordered component of at least 20% higher than those in implicit solvent, except for ff96/GB-OBC(II) that behaved similarly. Thus, the OPC solvent seems to model ID peptides better than both TIP3P and implicit solvent, especially when ff99SB or ff19SB are used.

PMFs obtained from φ and ψ dihedral distributions (Figures S81–S92) show that conformational space was explored by the different force fields and solvation methods very differently. In general, the PMFs showed that a slightly higher sampling is observed with OPC for the β-region, but global minima are always found in the α-region. Besides the effects of the OPC solvent, these data confirm that ff19SB is better than ff14SB in sampling conformations other than helical, and that OPC reduces the helical bias.

**Figure 13:**
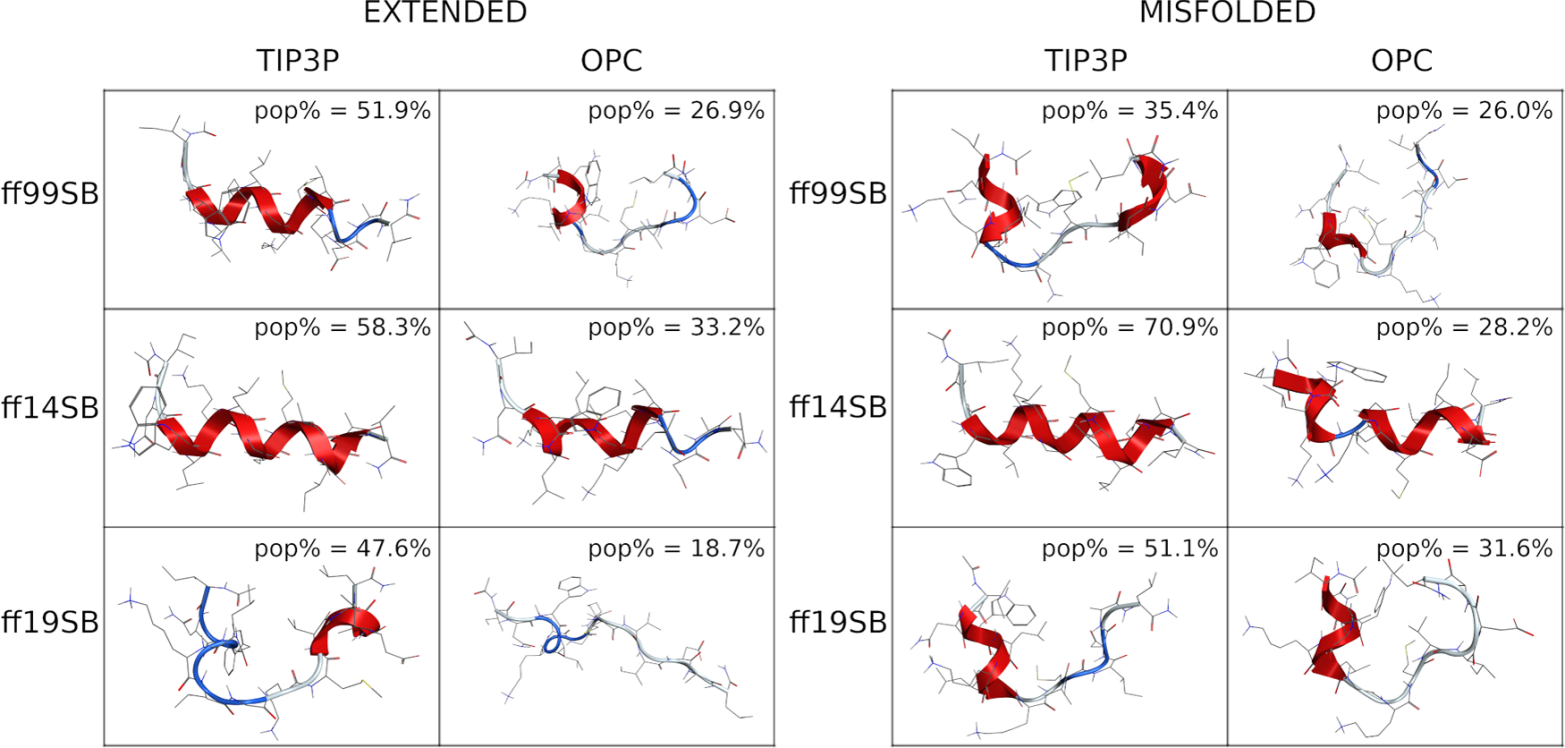
Representative conformation of the main cluster of ID1. (Left) Clusters obtained from extended simulations; (right) clusters obtained from misfolded simulations. The percentage of frames (pop%) comprising the clusters is also shown.

Cluster analyses (Figures 13 and S93) confirm the data reported above, as a well-structured helix was found as the representative conformation of the main cluster (pop% = 58.3 and 70.9 from extended and misfolded simulations, respectively) in both ff14SB/TIP3P simulations. The OPC model slightly improved this behavior, even if the representative conformation is still largely helical, especially for the extended simulation. A helical preference was also found for the ff99SB/TIP3P combination (pop% = 51.9%) in the extended simulation, while a less structured geometry was obtained from the misfolded simulation, as also observed in both ff99SB/OPC simulations. Excellent results were instead obtained with the ff19SB force field, regardless of the solvent model or the starting conformation.

Finally, H-bond analyses showed that the highest occupancies are obtained by the ff14SB/TIP3P simulation (Table S9), suggesting that more structured conformations are provided by this method. When the radius of gyration vs time was evaluated (Figure S94), the ff99SB/OPC combination and the ff19SB force field (with both the TIP3P and OPC solvents) showed a flatter distribution resembling the one typically seen for ID proteins.^66^ Taken together, these data suggest that ff99SB/OPC and ff19SB with both solvent models are probably the best choice, among those here evaluated, to simulate ID sequences.

**Figure 14:**
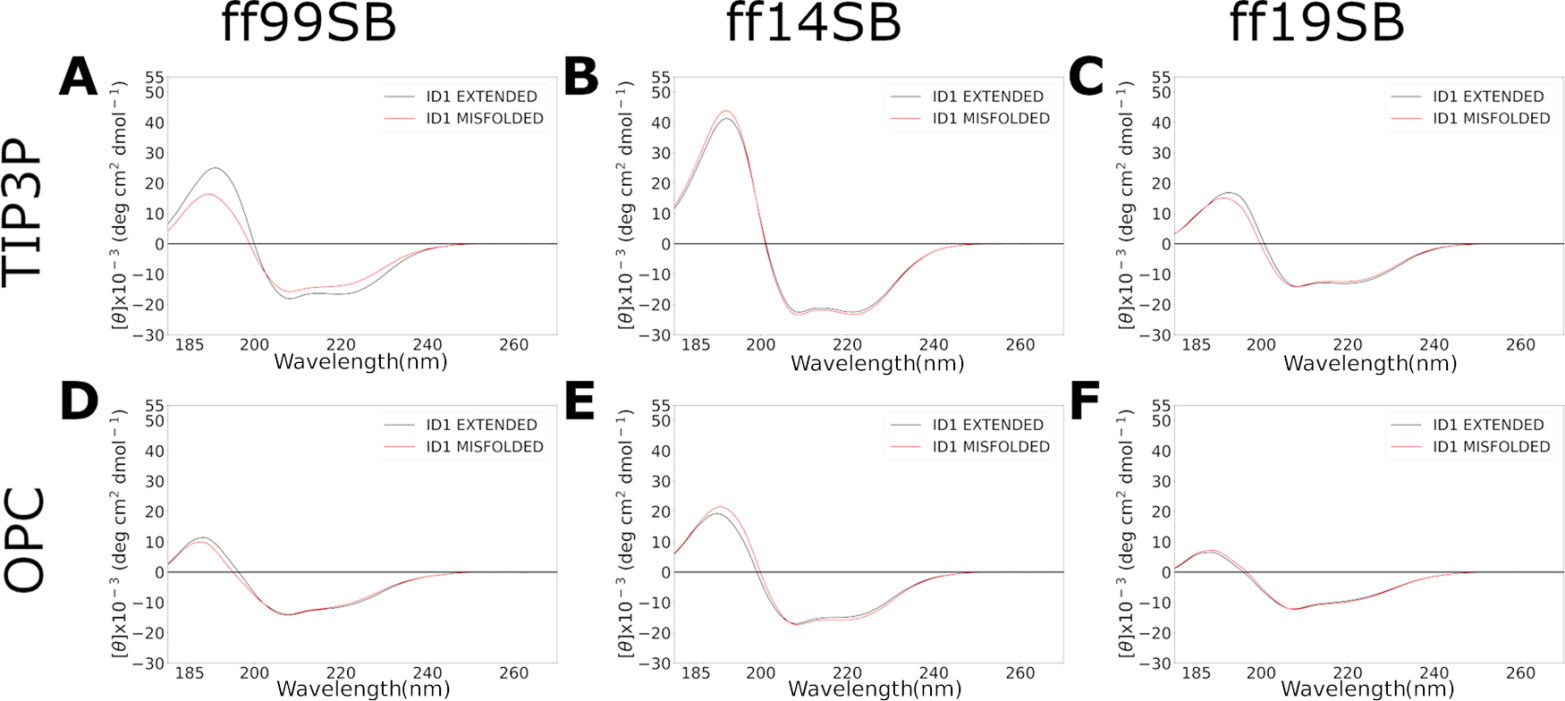
CD spectra of ID1 simulations calculated on the last 500 ns frames for each combination using SESCA software. Results from extended and misfolded simulations are reported in black and red lines, respectively.

CD spectra were calculated on the last 500 ns (Figure 14) and were similar to those observed for α-helix structures. On the other hand, when the main cluster is used for the calculation, ff19SB/OPC shows a spectrum like the experimental one^49^ when starting from the extended conformation (Figure S95). Thus, once again ff19SB, in this case, combined with OPC, provided the best results.

#### ID2 and ID3

The ID2 and ID3 peptides are sequences belonging to the TRTK-12 CapZ (PDB entry 1MWN) and p53 (1DT7) proteins, respectively. They are known to be helical in the protein but become disordered when isolated.^50−52^ Compared to ID1, similar conclusions can be drawn for both peptides. As shown by DSSP analysis, the ff14SB/TIP3P combination produced the highest helical content while the OPC model reduced it in all simulations (Figures 15 and 16). The PMFs obtained for the φ and ψ dihedral distributions of ID2 and ID3 (Figures S96–S113, respectively) in the ff14SB/TIP3P simulations are slightly different than those of ID1, as the left-handed helix and β-regions were also explored. As noted previously, the OPC solvent model improved sampling in all simulations (Figures S98–S109).

**Figure 15:**
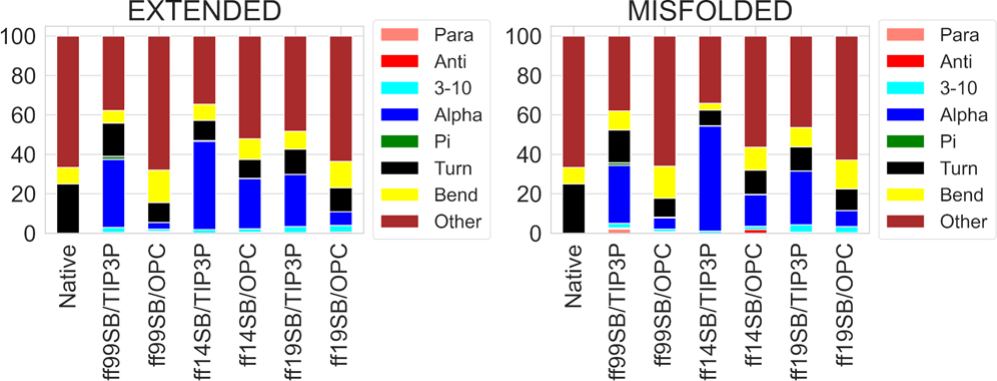
DSSP analysis of ID2 trajectories from extended and misfolded simulations. Values are expressed as a percentage of the distributions of all the residues considering the last 500 ns frames. The native structure derived from the NMR structure of TRTK-12 CapZ is also shown (PDB entry 1MWN).

**Figure 16:**
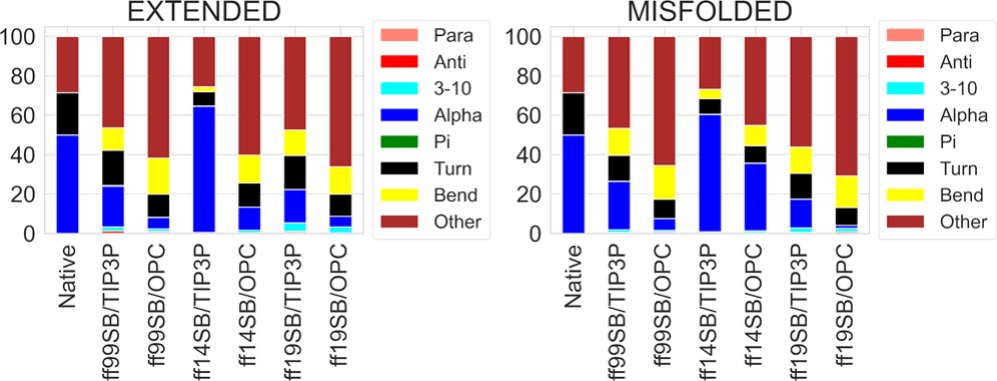
DSSP analysis of ID3 trajectories from extended and misfolded simulations. Values are expressed as a percentage of the distributions of all of the residues considering the last 500 ns frames. The native structure derived from the NMR structure of p53 is also shown (PDB entry 1DT7).

**Figure 17:**
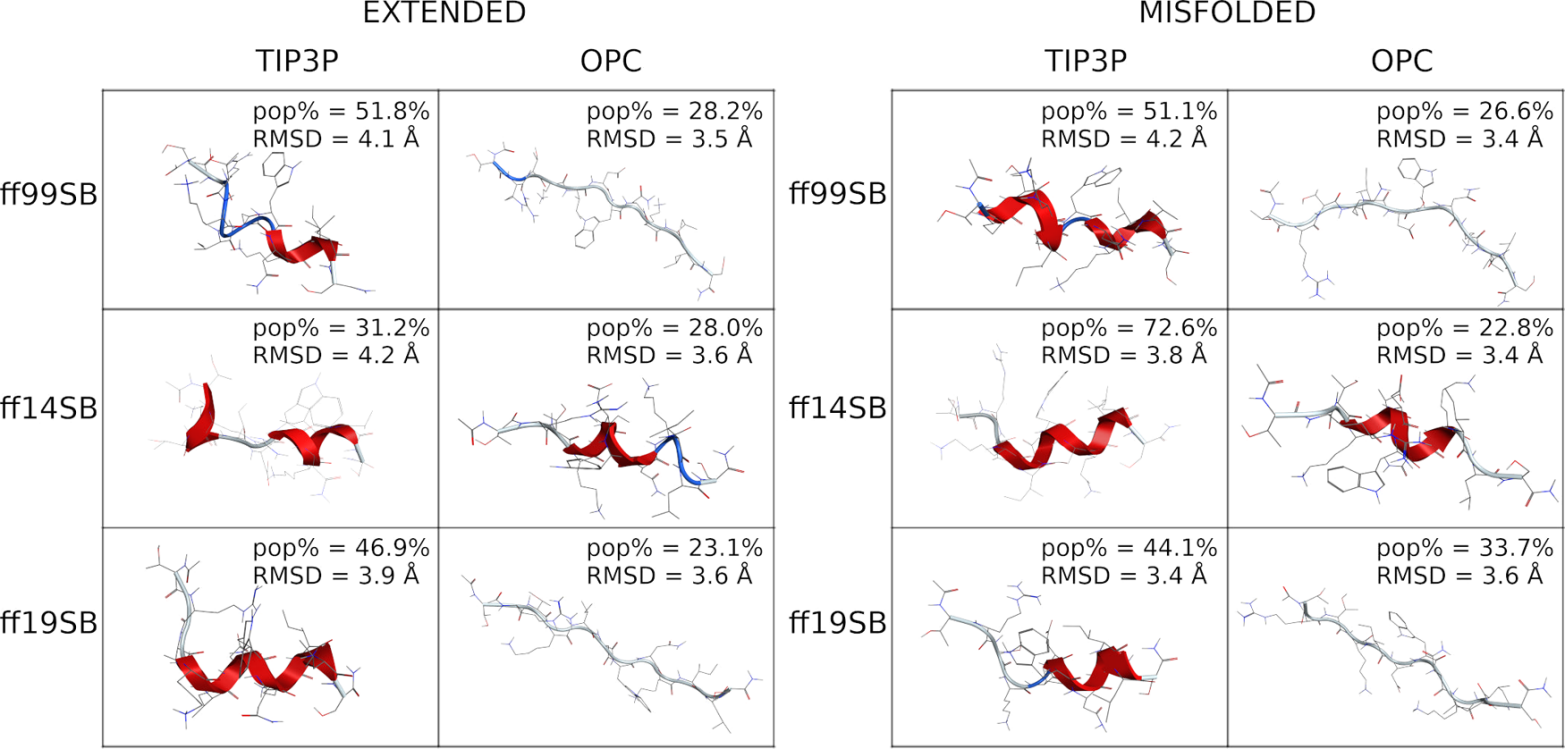
Representative conformation of the main cluster of ID2. (Left) Clusters obtained from extended simulations; (right) clusters obtained from misfolded simulations. The percentage of frames (pop%) comprising the clusters and the backbone RMSD between the representative conformation and the native conformation are also shown.

**Figure 18:**
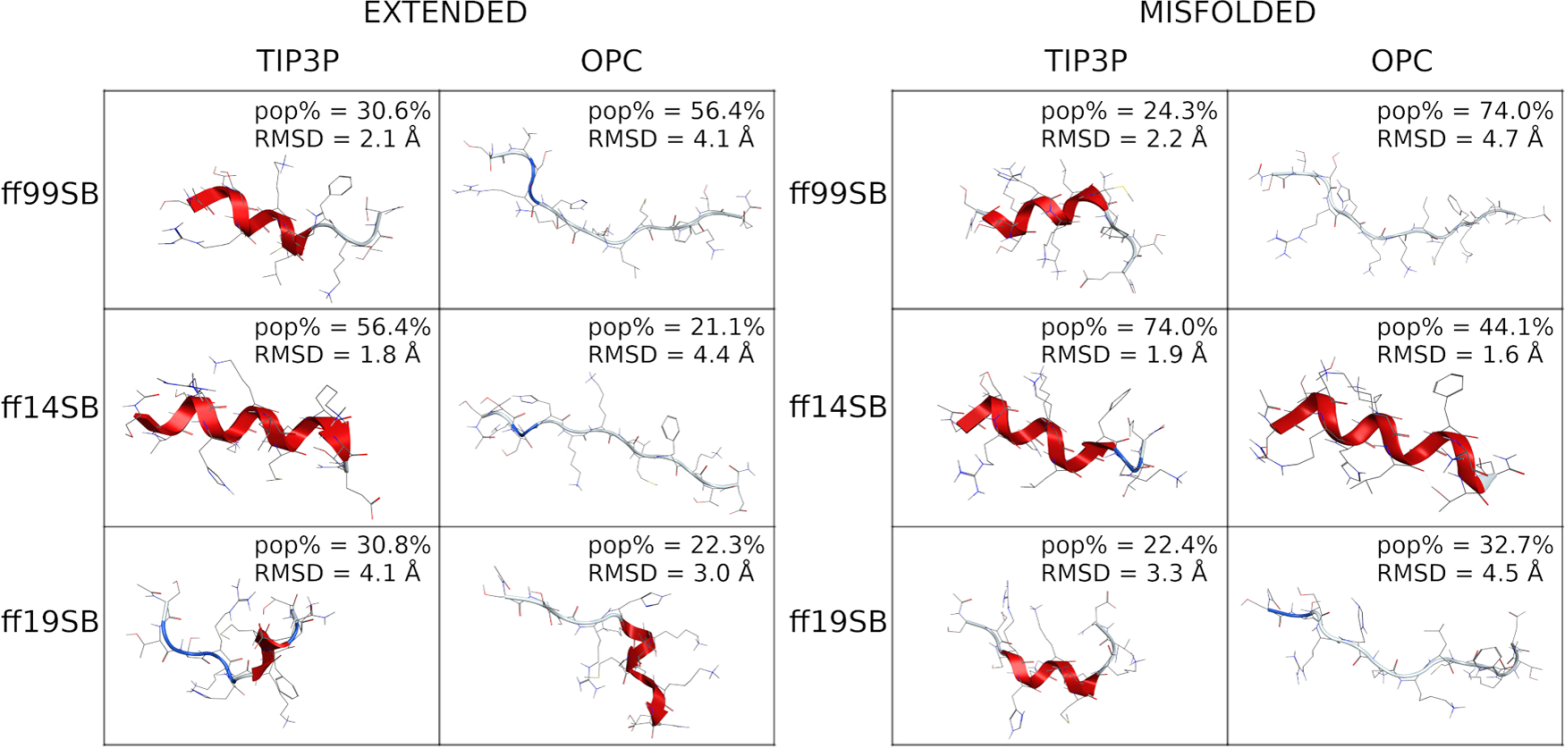
Representative conformation of the main cluster of ID3. (Left) Clusters obtained from extended simulations; (right) clusters obtained from misfolded simulations. The percentage of frames (pop%) comprising the clusters and the backbone RMSD between the representative conformation and the native conformation are also shown.

Cluster analyses (Figures 17, 18, S114, and S115) show that the representative conformations of the main clusters of the ff99SB/OPC and ff19SB/OPC combinations are disordered for both ID2 and ID3, with RMSDs ≥ 3.0 Å from the NMR native conformations in the full proteins (1MWN and 1DT7 for ID2 and ID3, respectively), where both sequences are structured. The representative conformation of the main cluster of ID3 from the ff14SB/OPC extended simulation also shows a disordered structure with a high RMSD (4.4 Å), compared to the structured native geometry. However, considering the low RMSD calculated from the superposition of the main cluster to the helical native geometry (1.6 Å), the peptide seems to fold into an α-helix in the ff14SB/OPC misfolded run.

The H-bond analyses show that several native interactions are formed for both peptides (Tables S10 and S11). The ff19SB/OPC misfolded simulation of ID3 resulted in the least structuration. Finally, the analyses of the radius of gyration (Figures S116 and S117) confirmed that ff14SB/TIP3P and ff99SB/TIP3P are the least indicated to simulate the ID peptides considered here.

## Discussion and Conclusions

Accelerated MD simulations were performed to determine which combination of AMBER force field and solvation model best reproduces the native conformation of two α-helices, three β-hairpins, and three ID peptides. This sampling method was chosen to ensure the exploration of a large portion of the potential energy surface with a relatively low computational effort.^18^ Three different Amber force fields were evaluated, namely, ff99SB,^38,39^ ff14SB,^40^ and ff19SB,^41^ each one coupled with either the TIP3P^42^ or the OPC^43^ explicit solvation model. For each system, two independent 1.5 μs aMD simulations were done, starting from an extended and misfolded conformation, respectively.

None of the combinations was able to reproduce all the native conformations. However, all the combinations could form stable helical conformations for peptides H1 and H2. The best combination for the helical peptides seems to be ff14SB/TIP3P; nonetheless, this combination folded most of the tested sequences into helices. All of the force field and solvent combinations used herein showed their limits in folding the three β-hairpin peptides. Indeed, a decent β-hairpin was only obtained by ff19SB for the B2 sequence. While both ff19SB and ff99SB sufficiently explored the β-region of the Ramachandran plot using both TIP3P and OPC solvation, a beneficial contribution of the OPC solvent was observed on β-hairpins only when using the ff99SB force field. In fact, the ff19SB/OPC combination led to a reduced exploration of the β-region, compared to ff19SB/TIP3P, for the B1 and B2. However, acceptable results were obtained for the uncapped B1 peptide, but by the ff99SB/TIP3P combination only. Conversely, low-energy conformations were mostly found in the β-region by ff19SB/OPC simulations for B3 only. Finally, all the combinations except those with ff14SB were able to well reproduce the folding behavior of ID peptides. However, the OPC solvation model was found to be better than TIP3P at limiting the helical bias of the evaluated force fields. Surprisingly, the ff19SB/OPC method seems the most sensitive to the starting conformation. In fact, H1, B1, B2, B3, and ID1 showed difference in DSSP components and/or in the conformation of the main cluster when comparing extended and misfolded simulation run with ff19SB/OPC. Conversely, no relevant differences were found among the extended or misfolded simulations when using different force field and solvent combinations.

In summary, ff99SB is still a rather valuable force field for folding predictions, especially if combined with OPC. However, ff19SB seems to be an improvement. Finding a preference for the solvation model is, on the other hand, less trivial. OPC seems to limit the helical bias, especially for ff99SB and ff14SB with β-hairpins and for ff19SB with ID peptides. However, TIP3P was found better in reproducing the H2 and B2 folding when the ff19SB force field was used. In conclusion, the OPC solvent seems to be more efficient when using older force fields, while TIP3P seems to work better with the newer one, where a lower dependence from the starting structure was also found. This is rather evident for β-hairpins that remains the most difficult secondary structure to be reproduced by the methods compared herein.

## Data Availability

Coordinates of the starting conformations are provided in the Supporting Information. The AmberTools package can be obtained at https://ambermd.org/.

## Supporting Information Available

Input parameters used for aMD simulations, output of potential energy average, DSSP results from extended simulations, PMFs obtained from φ and ψ dihedral distributions of all residues of all peptides from each simulation, three-dimensional conformations of the second populated clusters, H-bond analysis of each simulation for all peptides, radius of gyration from ID peptides simulations, simulation data for the uncapped B1 peptide, PDB coordinate of the starting geometry of each peptide in the extended and misfolded conformation, and CD spectra of the main cluster of H1 and ID1 (PDF)

## Abbreviations

Aib: α-aminoisobutyric acid
aMD: accelerated molecular dynamics
CA: α carbon
cMD: classical molecular dynamics
DSSP: define secondary structure of proteins
ID: intrinsically disordered
PES: potential energy surfaces
pop%: population percentage
PMF: potential of the mean force
PPI: protein–protein interaction
T-REMD: temperature replica exchange molecular dynamic
RMSD: root-mean-square deviation
