# Supplementary material for: Accelerated Molecular Dynamics for Peptide Folding: Benchmarking Different Combinations of Force Fields and Explicit Solvent Models
Source: J Chem Inf Model. 2023 May 10;63(10):3030–42. doi: 10.1021/acs.jcim.3c00138 (PMC10207344; doi:10.1021/acs.jcim.3c00138)
Supplement: Supplementary file 1 — ci3c00138_si_001.pdf [file ci3c00138_si_001.pdf]

Supporting Information

Accelerated Molecular Dynamics for Peptide Folding: Benchmarking Different Combinations of Amber Force Fields and Explicit Solvent Models

*Crescenzo Coppa,‡ Andrea Bazzoli,‡ Maral Barkhordari, Alessandro Contini**

Dipartimento di Scienze Farmaceutiche − Sezione di Chimica Generale e Organica "Alessandro Marchesini", Università degli Studi di Milano, Via Venezian, 21 20133 Milano, Italy

Index

1. Total potential energy and dihedral energy obtained from cMD simulations S4

2. Input parameters used in aMD simulations S6

3. RMSD vs time plots S9

4. DSSP analyses S13

5. Results of H1 peptide trajectory analyses S18
   a) PMF S18
   b) Clusters S30
   c) Hbonds S30
   d) CD S32

6. Results of H2 peptide trajectory analyses S33
   a) PMF S33
   b) Clusters S39
   c) Hbonds S39
   d) PMF from ff15ipq-m S40

7. Results of B1 peptide trajectory analyses S42
   a) PMF S42
   b) Clusters S54
   c) Hbonds S54

8. Results of B1 uncapped peptide trajectory analyses S56
   a) DSSP S56
   b) PMF S57
   c) Clusters S61
   d) Hbonds S61

9. Results of B2 peptide trajectory analyses S62
   a) PMF S62
   b) Clusters S68
   c) Hbonds S68

10. Results of B3 peptide trajectory analyses S70
    a) PMF S70
    b) Clusters S82
    c) Hbonds S82

11. Results of ID1 peptide trajectory analyses S84
a) PMF S84
b) Clusters S96
c) Hbonds S96
d) Radius of gyration S98
e) CD S98

12. Results of ID2 peptide trajectory analyses S99
a) PMF S99
b) Clusters S117
c) Hbonds S118
d) Radius of gyration S121

13. Results of ID3 peptide trajectory analyses S105
a) PMF S105
b) Clusters S117
c) Hbonds S119
d) Radius of gyration S121

14. PDB coordinate of the starting structures. S122
a) H1 extended S122
b) H1 misfolded S123
c) H2 extended S124
d) H2 misfolded S124
e) B1 extended S125
f) B1 misfolded S126
g) B2 extended S127
h) B2 misfolded S128
i) B3 extended S129
j) B3 misfolded S130
k) ID1 extended S131
l) ID1 misfolded S132
m) ID2 extended S133
n) ID2 misfolded S134
o) ID3 extended S135
p) ID3 misfolded S136

Table S1. Total Potential Energy (EPTOT) Average and dihedral angle (DHIED) average values obtained by cMD

ff99SB TIP3P	Average EPTOT*	Average DHIED*
H1 EXTENDED	-24216.6	171.0
H1 MISFOLDED	-27050.1	172.3
H2 EXTENDED	-10078.0	58.3
H2 MISFOLDED	-10244.8	60.0
B1 EXTENDED	-30044.5	165.0
B1 MISFOLDED	-23632.6	168.4
B2 EXTENDED	-22522.8	135.8
B2 MISFOLDED	-22541.2	139.2
B3 EXTENDED	-34514.5	159.1
B3 MISFOLDED	-22223.9	162.8
ID1 EXTENDED	-15929.4	150.4
ID1 MISFOLDED	-20480.7	148.2
ID2 EXTENDED	-24007.8	137.4
ID2 MISFOLDED	-18957.1	140.8
ID3 EXTENDED	-21743.6	139.0
ID3 MISFOLDED	-17957.3	149.8
**ff99SB OPC**	**Average EPTOT***	**Average DHIED***
H1 EXTENDED	-39642.5	171.0
H1 MISFOLDED	-35971.0	171.1
H2 EXTENDED	-14029.0	58.1
H2 MISFOLDED	-13261.7	60.1
B1 EXTENDED	-37900.0	162.0
B1 MISFOLDED	-30039.4	162.8
B2 EXTENDED	-25786.0	134.5
B2 MISFOLDED	-28368.7	136.4
B3 EXTENDED	-39629.6	160.8
B3 MISFOLDED	-28127.9	163.6
ID1 EXTENDED	-20304.9	146.8
ID1 MISFOLDED	-26763.9	152.7
ID2 EXTENDED	-30045.8	135.1
ID2 MISFOLDED	-21756.8	142.4
ID3 EXTENDED	-29418.7	136.8
ID3 MISFOLDED	-24346.0	142.1
**ff14SB TIP3P**	**Average EPTOT***	**Average DHIED***
H1 EXTENDED	-40384.0	191.6
H1 MISFOLDED	-27024.7	191.5
H2 EXTENDED	-10100.9	55.8
H2 MISFOLDED	-10244.7	58.2
B1 EXTENDED	-25237.0	210.9
B1 MISFOLDED	-23582.6	215.6
B2 EXTENDED	-20087.7	163.6

B2 MISFOLDED	-22518.1	163.5
B3 EXTENDED	-25025.7	199.6
B3 MISFOLDED	-22192.7	202.4
ID1 EXTENDED	-24497.5	169.2
ID1 MISFOLDED	-20454.6	174.7
ID2 EXTENDED	-25311.8	161.4
ID2 MISFOLDED	-18934.8	166.9
ID3 EXTENDED	-24036.5	169.4
ID3 MISFOLDED	-17935.0	173.9
**ff14SB OPC**	**Average EPTOT***	**Average DHIED***
H1 EXTENDED	-41568.5	191.2
H1 MISFOLDED	-35941.6	198.5
H2 EXTENDED	-13718.6	53.5
H2 MISFOLDED	-13261.2	58.8
B1 EXTENDED	-41820.8	192.9
B1 MISFOLDED	-29989.0	216.8
B2 EXTENDED	-28981.6	162.4
B2 MISFOLDED	-28345.7	164.1
B3 EXTENDED	-41820.8	192.9
B3 MISFOLDED	-28091.5	203.0
ID1 EXTENDED	-28280.6	170.2
ID1 MISFOLDED	-26743.3	172.6
ID2 EXTENDED	-31638.8	160.8
ID2 MISFOLDED	-21731.1	167.0
ID3 EXTENDED	-3331.1	159.4
ID3 MISFOLDED	-24324.5	172.2
**ff19SB TIP3P**	**Average EPTOT***	**Average DHIED***
H1 EXTENDED	-21846.2	93.3
H1 MISFOLDED	-27115.7	95.0
H2 EXTENDED	-95440.8	43.0
H2 MISFOLDED	-10259.3	45.5
B1 EXTENDED	-33799.7	96.9
B1 MISFOLDED	-23683.7	97.1
B2 EXTENDED	-19732.3	79.5
B2 MISFOLDED	-22595.7	79.4
B3 EXTENDED	-23016.5	88.2
B3 MISFOLDED	-22285.6	90.8
ID1 EXTENDED	-17731.1	74.5
ID1 MISFOLDED	-20548.9	74.1
ID2 EXTENDED	-26174.8	77.2
ID2 MISFOLDED	-19015.7	77.6
ID3 EXTENDED	-19476.7	70.7
ID3 MISFOLDED	-18060.0	72.7
**ff19SB OPC**	**Average EPTOT***	**Average DHIED***
H1 EXTENDED	-38906.2	94.8
H1 MISFOLDED	-36042.9	97.6

H2 EXTENDED	-11618.7	43.5
H2 MISFOLDED	-13272.9	47.0
B1 EXTENDED	-38353.5	92.1
B1 MISFOLDED	-30120.1	97.4
B2 EXTENDED	-20528.5	81.2
B2 MISFOLDED	-28711.7	81.5
B3 EXTENDED	-29998.9	90.9
B3 MISFOLDED	-28189.3	90.2
ID1 EXTENDED	-19186.2	75.2
ID1 MISFOLDED	-26824.6	74.0
ID2 EXTENDED	-41710.2	78.8
ID2 MISFOLDED	-21815.0	78.6
ID3 EXTENDED	-25853.0	72.0
ID3 MISFOLDED	-24403.9	72.7
**ff15ipq-m TIP3P**	**Average EPTOT***	**Average DHIED***
H2 EXTENDED	-15450.5	-47.5
**ff15ipq-m OPC**	**Average EPTOT***	**Average DHIED***
H2 EXTENDED	-9985.6	-43.8
**ff15ipq-m SPC/E**	**Average EPTOT***	**Average DHIED***
H2 EXTENDED	-17038.3	-45.7

* values are reported in kcal/mol

Table S2. parameters used in aMDs simulations

ff99SB TIP3P	ethreshd *	alphad*	ethreshp*	alphap*
H1 EXTENDED	239.0	13.6	-22981.4	1235.2
H1 MISFOLDED	240.3	13.6	-25672.3	1377.8
H2 EXTENDED	86.3	5.6	-9561.2	516.8
H2 MISFOLDED	87.9	5.6	-9719.4	525.4
B1 EXTENDED	237.0	14.4	-28535.2	1509.3
B1 MISFOLDED	240.4	14.4	-22445.9	1186.7
B2 EXTENDED	191.8	11.2	-21372.3	1150.6
B2 MISFOLDED	195.2	11.2	-21389.7	1151.5
B3 EXTENDED	227.1	13.6	-32759.6	1754.9
B3 MISFOLDED	230.8	13.6	-21087.3	1136.6
ID1 EXTENDED	214.4	12.8	-15107.2	822.2
ID1 MISFOLDED	212.2	12.8	-19429.5	1051.2
ID2 EXTENDED	193.4	11.2	-22797.8	1210.1
ID2 MISFOLDED	196.8	11.2	-18000.9	956.2
ID3 EXTENDED	199.0	12.0	-20646.8	1096.8
ID3 MISFOLDED	209.8	12.0	-17054.1	903.2
**ff99SB OPC**	**ethreshd ***	**alphad***	**ethreshp***	**alphap***
H1 EXTENDED	239.0	13.6	-37555.3	2087.2
H1 MISFOLDED	239.0	13.6	-34075.8	1895.2
H2 EXTENDED	86.1	5.6	-13286.6	742.4
H2 MISFOLDED	88.1	5.6	-12559.0	702.7

B1 EXTENDED	234.0	14.4	-35925.0	1975.0
B1 MISFOLDED	234.8	14.4	-28475.2	1564.2
B2 EXTENDED	190.5	11.2	-24422.0	1364.0
B2 MISFOLDED	192.4	11.2	-26869.7	1499.0
B3 EXTENDED	228.8	13.6	-37541.8	2087.8
B3 MISFOLDED	231.6	13.6	-26641.0	1486.9
ID1 EXTENDED	210.8	12.8	-19224.4	1080.5
ID1 MISFOLDED	216.7	12.8	-25345.5	1418.4
ID2 EXTENDED	191.1	11.2	-28475.0	1570.7
ID2 MISFOLDED	198.4	11.2	-20618.7	1138.1
ID3 EXTENDED	196.8	12.0	-27879.5	1539.2
ID3 MISFOLDED	202.1	12.0	-23074.7	1271.4
**ff14SB TIP3P**	**ethreshd ***	**alphad***	**ethreshp***	**alphap***
H1 EXTENDED	259.6	13.6	-38334.2	2049.8
H1 MISFOLDED	259.5	13.6	-25646.9	1377.8
H2 EXTENDED	83.8	5.6	-9583.2	517.8
H2 MISFOLDED	86.2	5.6	-9719.3	525.4
B1 EXTENDED	282.9	14.4	-23967.3	1269.7
B1 MISFOLDED	287.6	14.4	-22395.9	1186.7
B2 EXTENDED	219.6	11.2	-19058.6	1029.1
B2 MISFOLDED	219.6	11.2	-21366.6	1151.5
B3 EXTENDED	267.6	13.6	-23746.5	1279.2
B3 MISFOLDED	270.4	13.6	-21056.1	1136.6
ID1 EXTENDED	233.2	12.8	-23242.8	1254.7
ID1 MISFOLDED	238.7	12.8	-19403.4	1051.2
ID2 EXTENDED	217.4	11.2	-24034.5	1277.3
ID2 MISFOLDED	222.9	11.2	-17978.6	956.2
ID3 EXTENDED	229.5	12.0	-22823.1	1213.4
ID3 MISFOLDED	233.9	12.0	-17031.8	903.2
**ff14SB OPC**	**ethreshd ***	**alphad***	**ethreshp***	**alphap***
H1 EXTENDED	259.2	13.6	-39379.5	2189.0
H1 MISFOLDED	266.5	13.6	-34046.4	1895.2
H2 EXTENDED	84.7	5.6	-11814.3	661.1
H2 MISFOLDED	86.8	5.6	-12558.5	702.7
B1 EXTENDED	284.2	14.4	-38756.7	2133.8
B1 MISFOLDED	288.8	14.4	-28424.8	1564.2
B2 EXTENDED	218.4	11.2	-27449.3	1532.3
B2 MISFOLDED	220.1	11.2	-26846.7	1499.0
B3 EXTENDED	260.9	13.6	-39616.5	2204.3
B3 MISFOLDED	271.0	13.6	-26604.6	1486.9
ID1 EXTENDED	234.2	12.8	-26782.2	1498.4
ID1 MISFOLDED	236.6	12.8	-25324.9	1418.4
ID2 EXTENDED	216.8	11.2	-29983.0	1655.8
ID2 MISFOLDED	223.0	11.2	-20593.0	1138.1
ID3 EXTENDED	219.4	12.0	-28743.3	1587.8
ID3 MISFOLDED	232.2	12.0	-23053.2	1271.4

**ff19SB TIP3P**	**ethreshd ***	**alphad***	**ethreshp***	**alphap***
H1 EXTENDED	161.3	13.6	-20733.9	1112.3
H1 MISFOLDED	163.0	13.6	-25737.9	1377.8
H2 EXTENDED	71.0	5.6	-9055.1	489.0
H2 MISFOLDED	73.5	5.6	-9733.9	525.4
B1 EXTENDED	168.9	14.4	-32104.1	1695.5
B1 MISFOLDED	169.1	14.4	-22497.0	1186.7
B2 EXTENDED	135.5	11.2	-18724.8	1007.5
B2 MISFOLDED	135.4	11.2	-21444.2	1151.5
B3 EXTENDED	156.2	13.6	-21842.9	1173.6
B3 MISFOLDED	158.7	13.6	-21149.0	1136.6
ID1 EXTENDED	138.5	12.8	-16821.5	909.6
ID1 MISFOLDED	138.1	12.8	-19497.7	1051.2
ID2 EXTENDED	133.2	11.2	-24858.1	1316.6
ID2 MISFOLDED	133.6	11.2	-18059.5	956.2
ID3 EXTENDED	130.8	12.0	-18497.0	979.7
ID3 MISFOLDED	132.7	12.0	-17154.8	905.1
**ff19SB OPC**	**ethreshd ***	**alphad***	**ethreshp***	**alphap***
H1 EXTENDED	162.8	13.6	-36861.2	2045.0
H1 MISFOLDED	165.6	13.6	-34147.7	1895.2
H2 EXTENDED	71.5	5.6	-11003.0	615.7
H2 MISFOLDED	75.0	5.6	-12570.2	702.7
B1 EXTENDED	164.1	14.4	-36357.3	1996.2
B1 MISFOLDED	169.4	14.4	-28555.9	1564.2
B2 EXTENDED	137.2	11.2	-19441.6	1086.9
B2 MISFOLDED	137.5	11.2	-27197.3	1514.4
B3 EXTENDED	158.9	13.6	-28417.3	1581.6
B3 MISFOLDED	158.2	13.6	-26702.4	1486.9
ID1 EXTENDED	139.2	12.8	-18167.1	1019.0
ID1 MISFOLDED	138.0	12.8	-25406.8	1417.8
ID2 EXTENDED	134.8	11.2	-39532.1	2178.1
ID2 MISFOLDED	134.6	11.2	-20676.9	1138.1
ID3 EXTENDED	131.9	12.0	-24503.2	1349.8
ID3 MISFOLDED	132.7	12.0	-23133.2	1270.7
**ff15ipq-m TIP3P**	**ethreshd ***	**alphad***	**ethreshp***	**alphap***
H2 EXTENDED	-19.5	5.6	-14669.2	781.3
**ff15ipq-m OPC**	**ethreshd ***	**alphad***	**ethreshp***	**alphap***
H2 EXTENDED	-15.8	5.6	-9204.3	781.3
**ff15ipq-m SPC/E**	**ethreshd ***	**alphad***	**ethreshp***	**alphap***
H2 EXTENDED	-17.7	5.6	-16257.0	781.3

* values are reported in kcal/mol

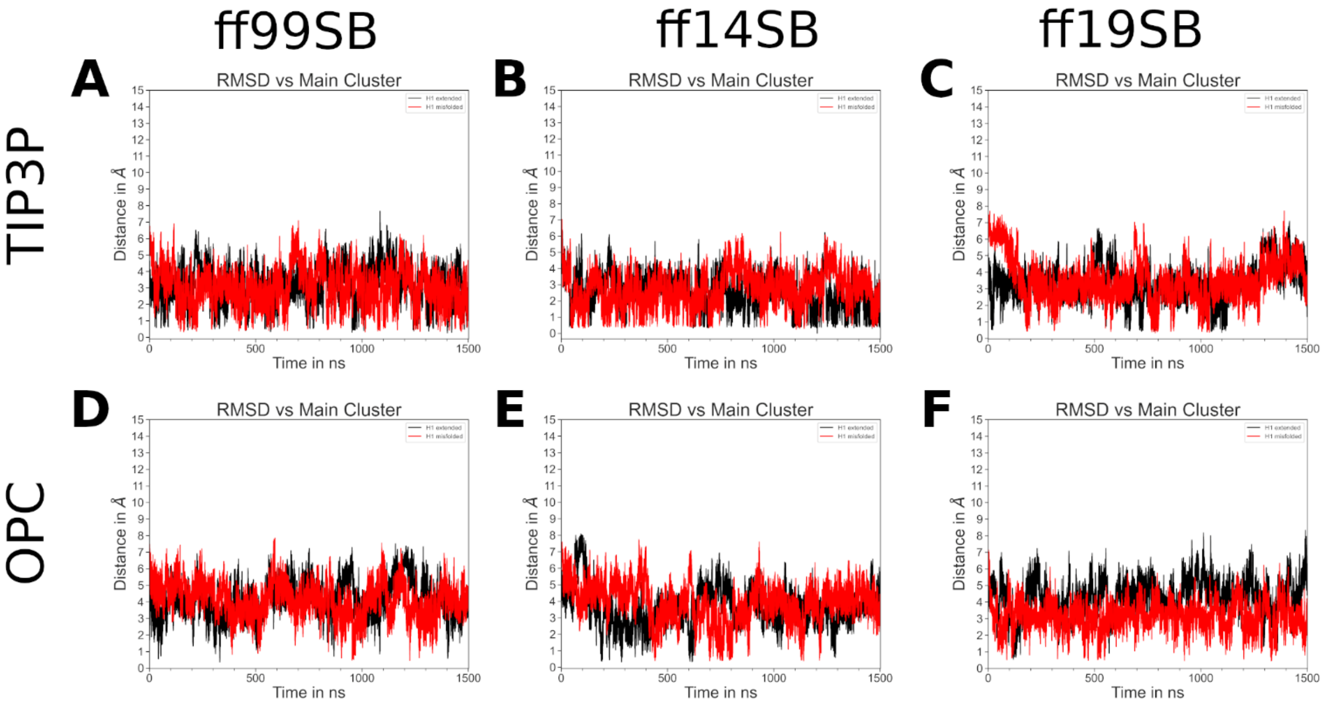

Figure S1. RMSD of H1 for extended (black) and misfolded (red) aMDs. The main cluster obtained from the last 500 ns of ff14SB/TIP3P simulation starting from extended conformation was used as the reference.

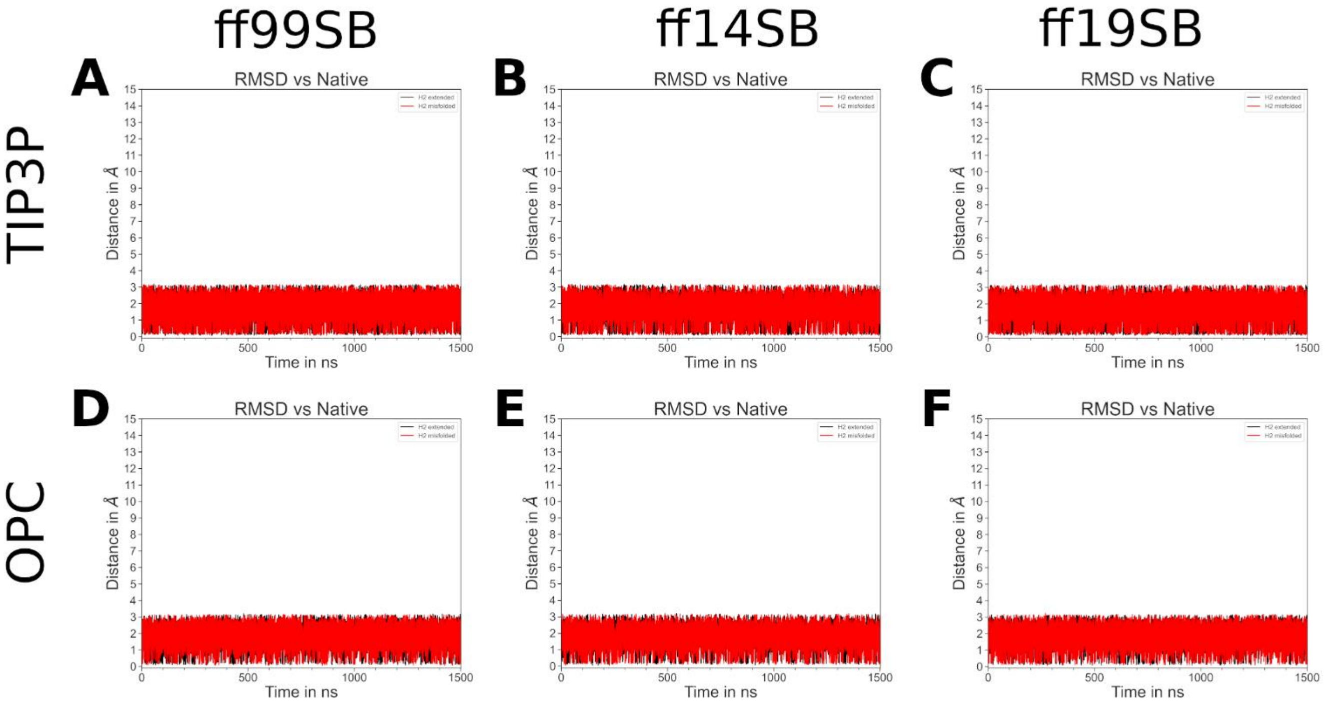

Figure S2. RMSD of H2 for extended (black) and misfolded (red) aMDs. The native structure was used as the reference.

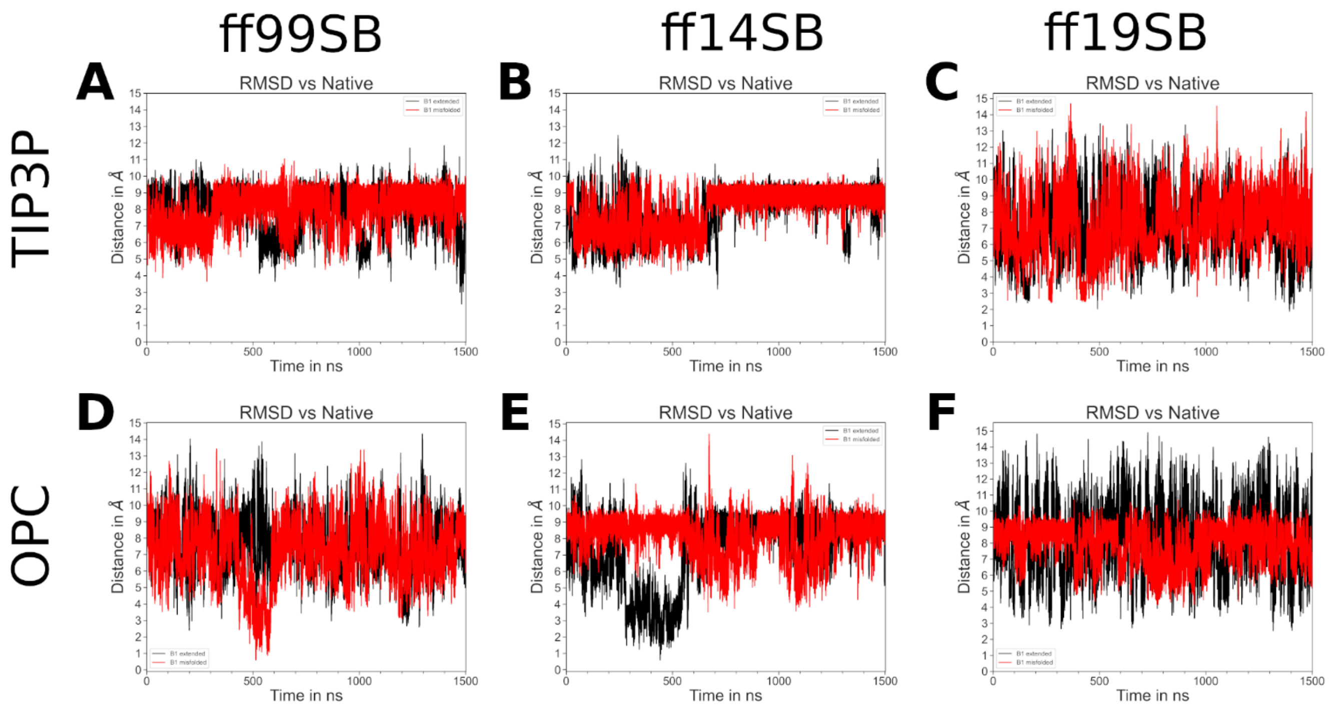

Figure S3. RMSD of B1 for extended (black) and misfolded (red) aMDs. The native structure was used as the reference.

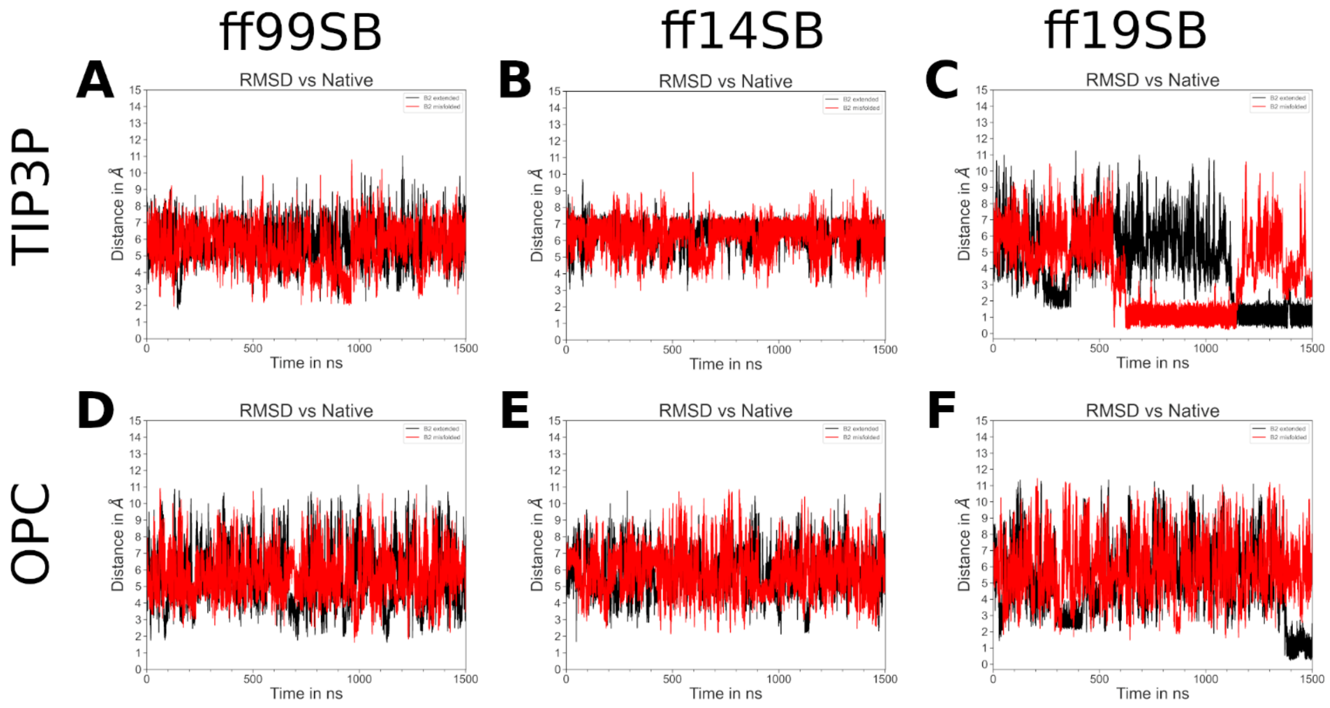

Figure S4. RMSD of B2 for extended (black) and misfolded (red) aMDs. The native structure was used as the reference.

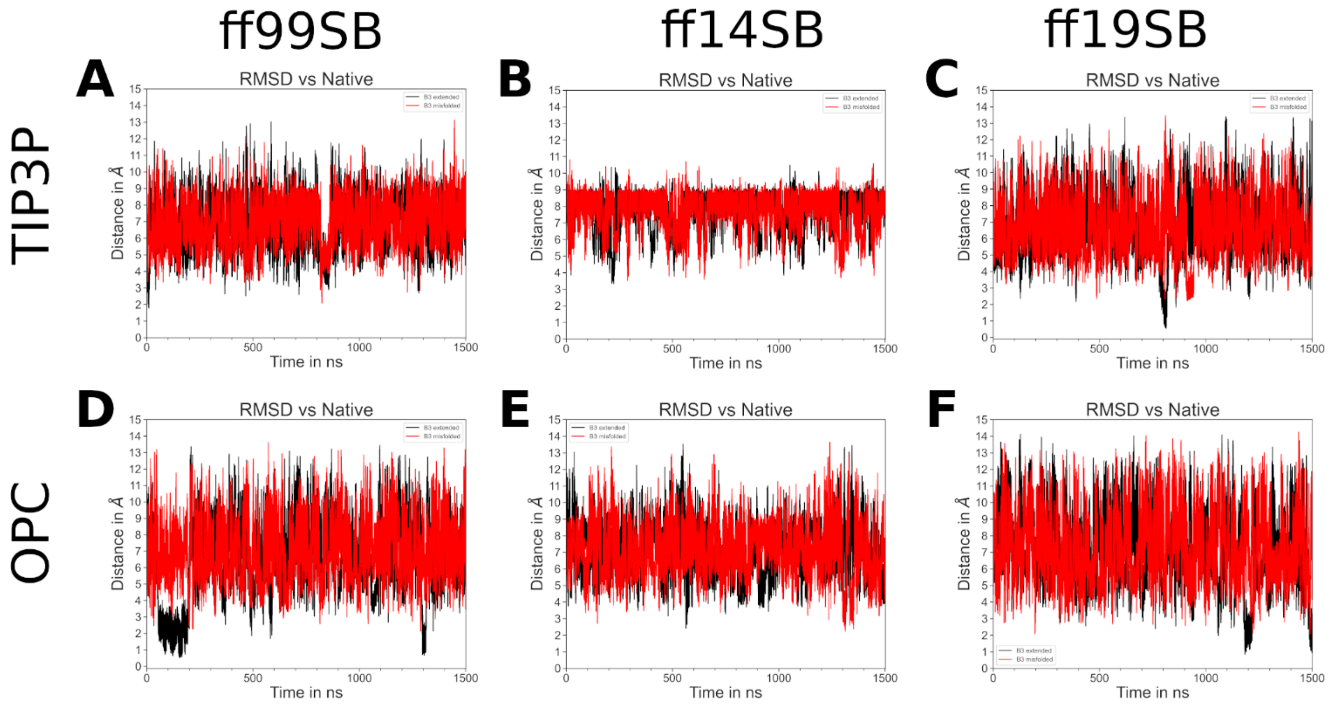

Figure S5. RMSD of B3 for extended (black) and misfolded (red) aMDs. The native structure was used as the reference.

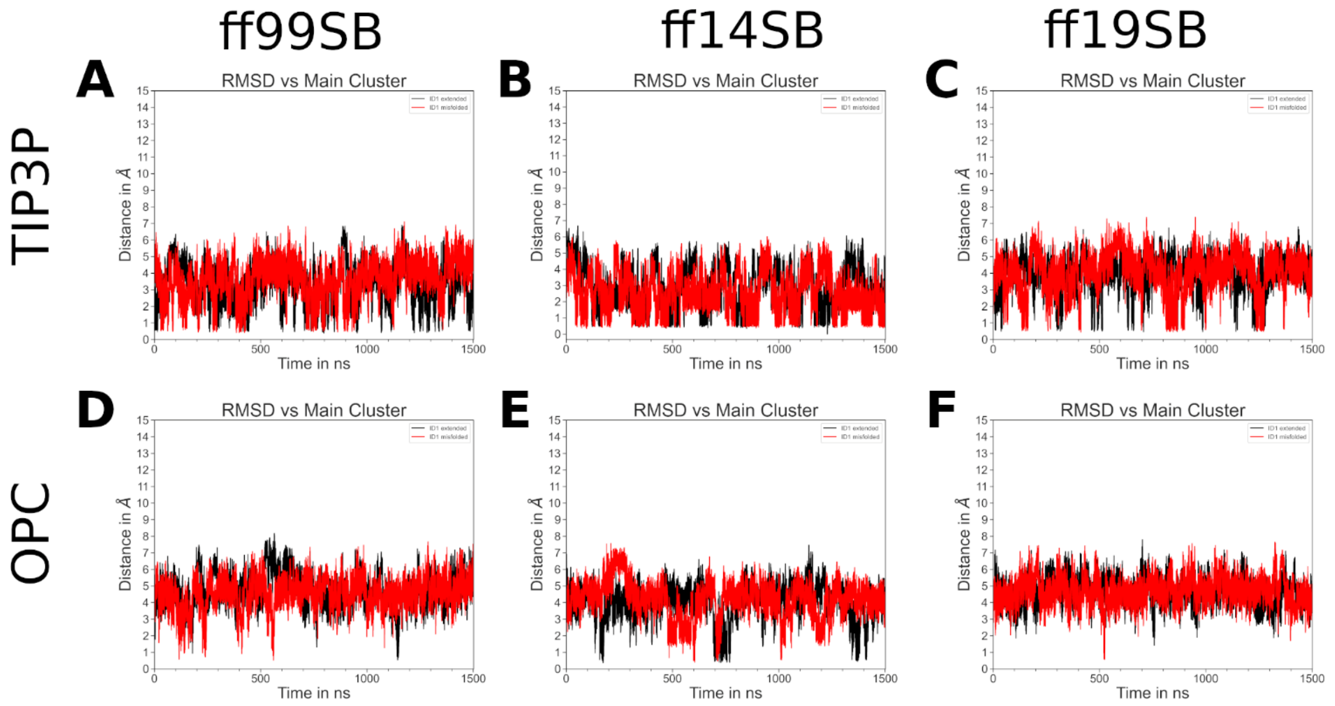

Figure S6. RMSD of ID1 for extended (black) and misfolded (red) aMDs. The main cluster obtained from the last 500 ns of the extended ff14SB/TIP3P simulation was used as the reference.

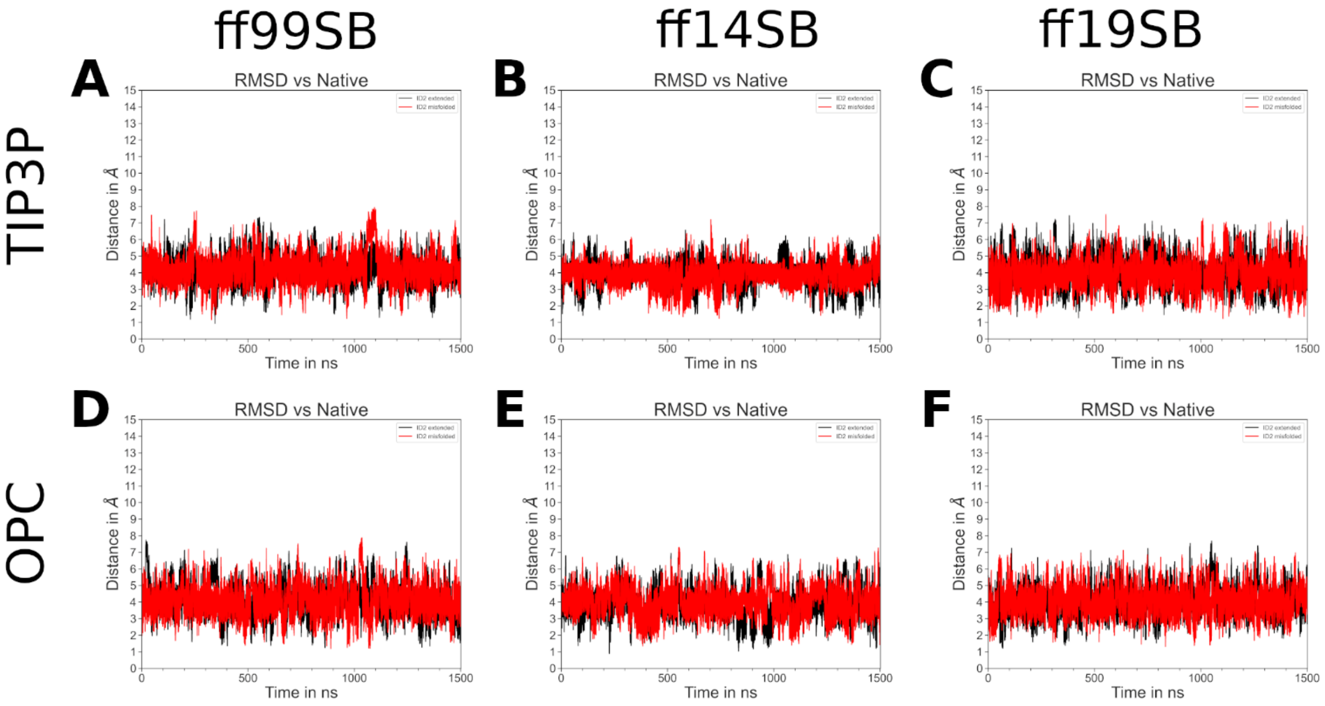

Figure S7. RMSD of ID2 for extended (black) and misfolded (red) aMD. The native structure was used as the reference.

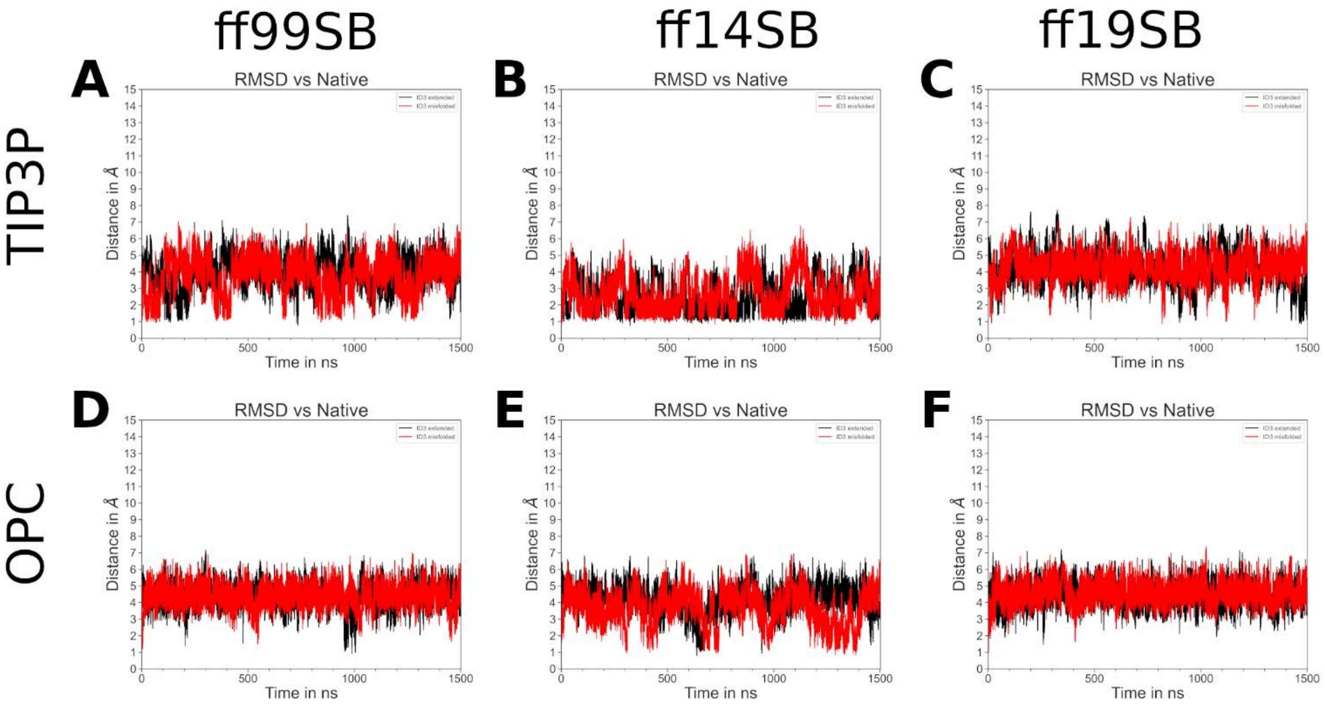

Figure S8. RMSD of ID3 for extended (black) and misfolded (red) aMDs. The native structure was used as the reference.

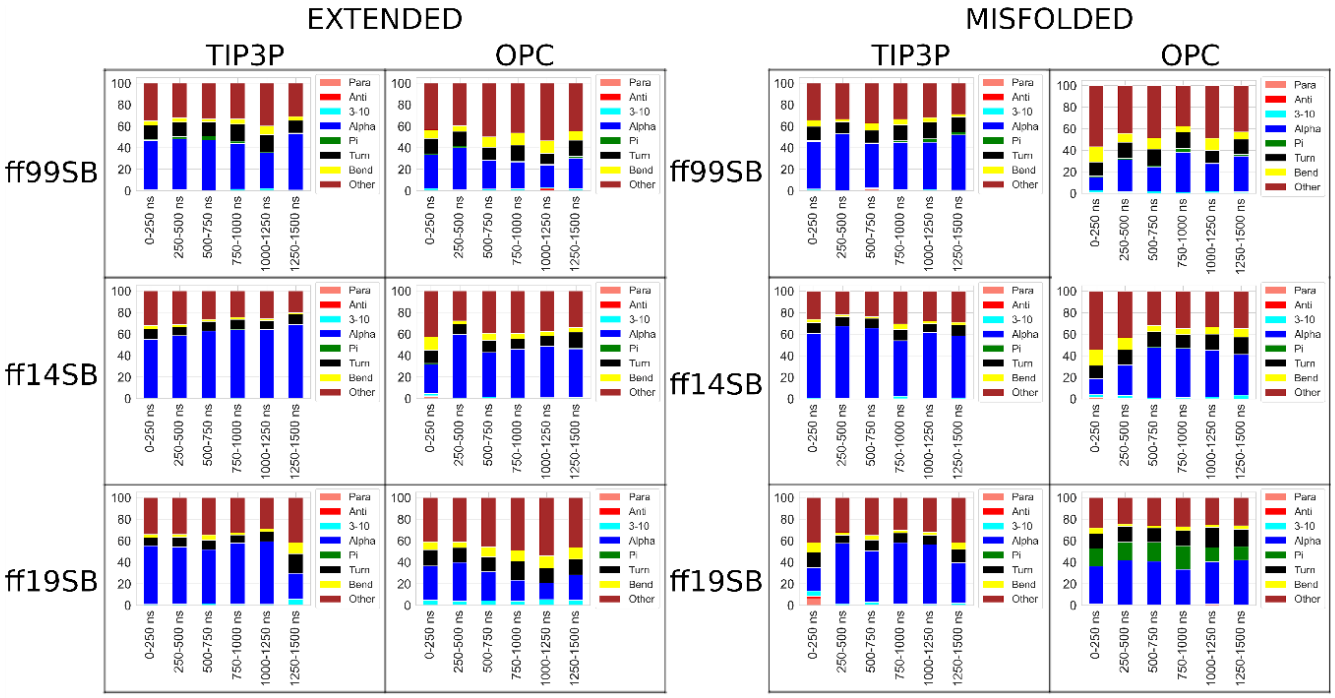

Figure S9. H1 DSSP for extended (left) and misfolded (right) aMDs, calculated at 250 ns intervals.

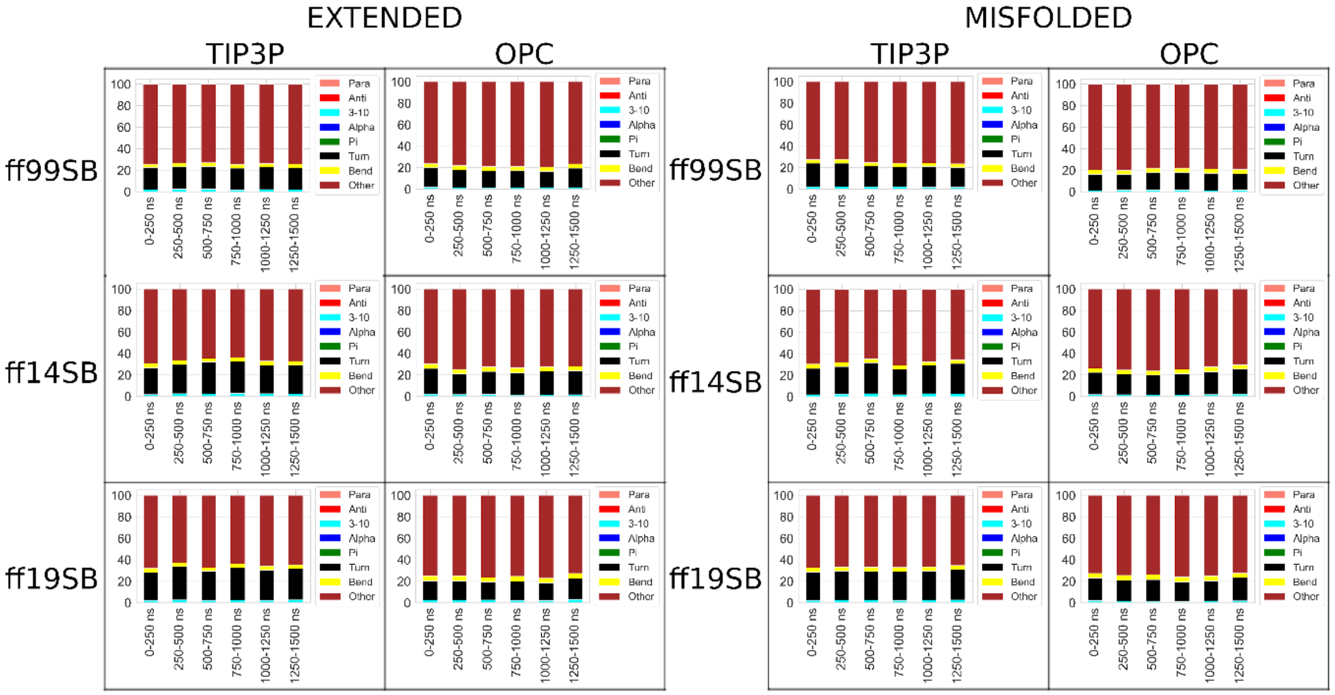

Figure S10. H2 DSSP for extended (left) and misfolded (right) aMDs, calculated at 250 ns intervals.

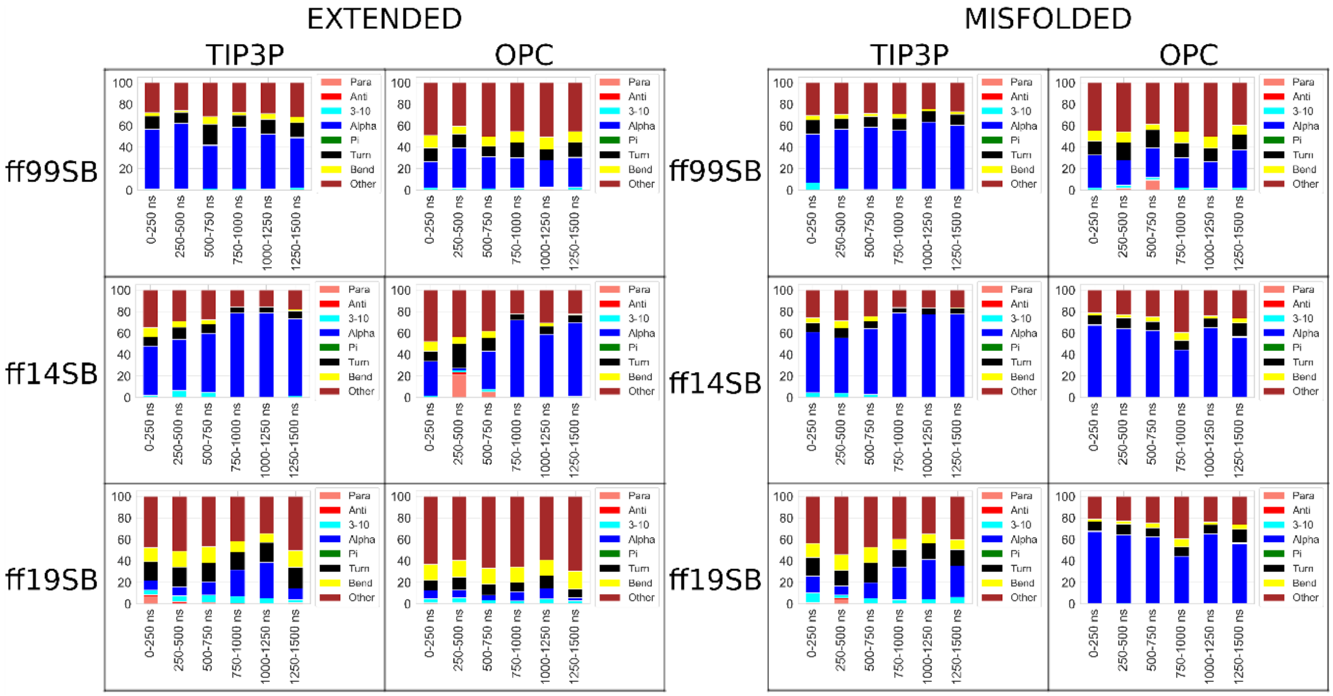

Figure S11. B1 DSSP for extended (left) and misfolded (right) aMDs, calculated at 250 ns intervals.

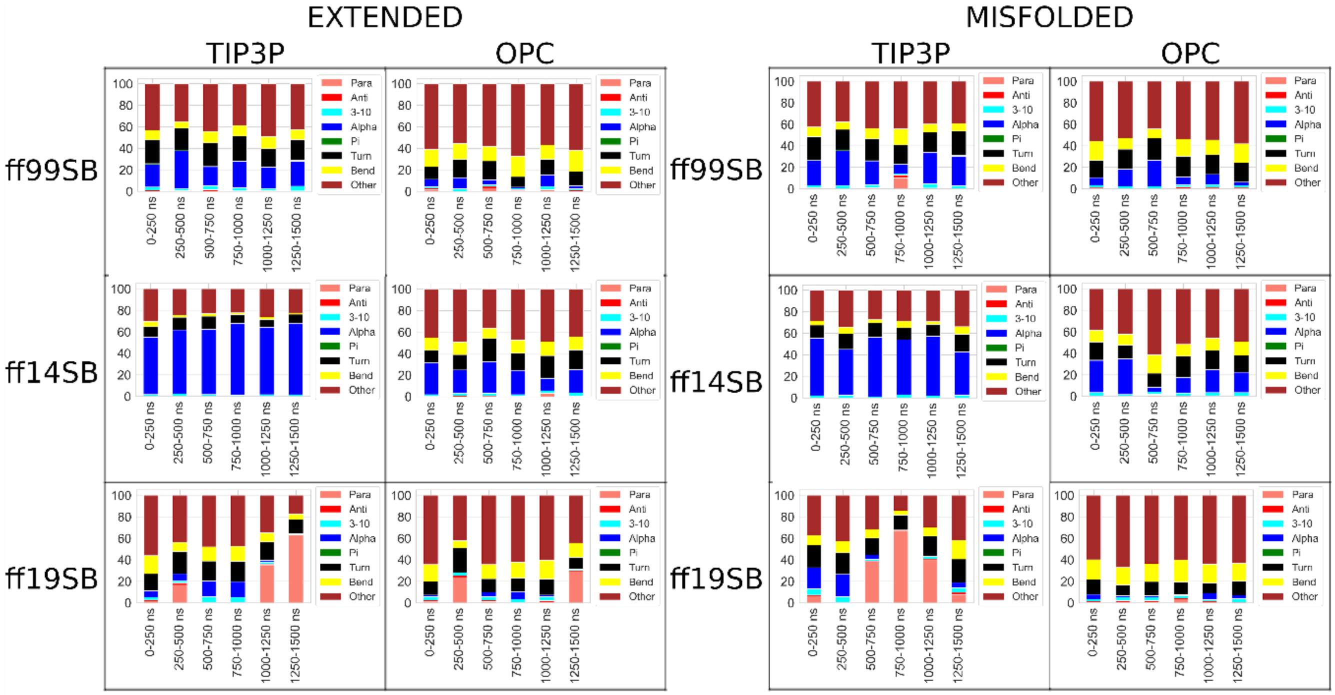

Figure S12. B2 DSSP for extended (left) and misfolded (right) aMDs, calculated at 250 ns intervals.

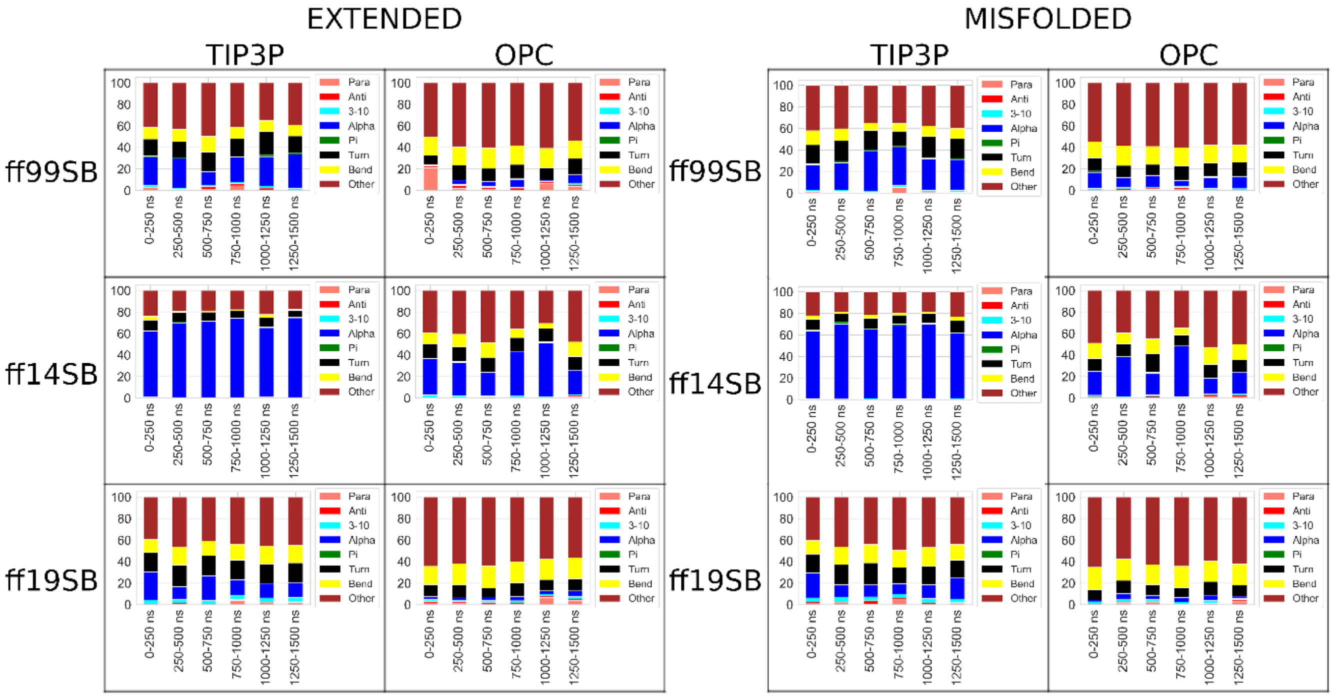

Figure S13. B3 DSSP for extended (left) and misfolded (right) aMDs, calculated at 250 ns intervals.

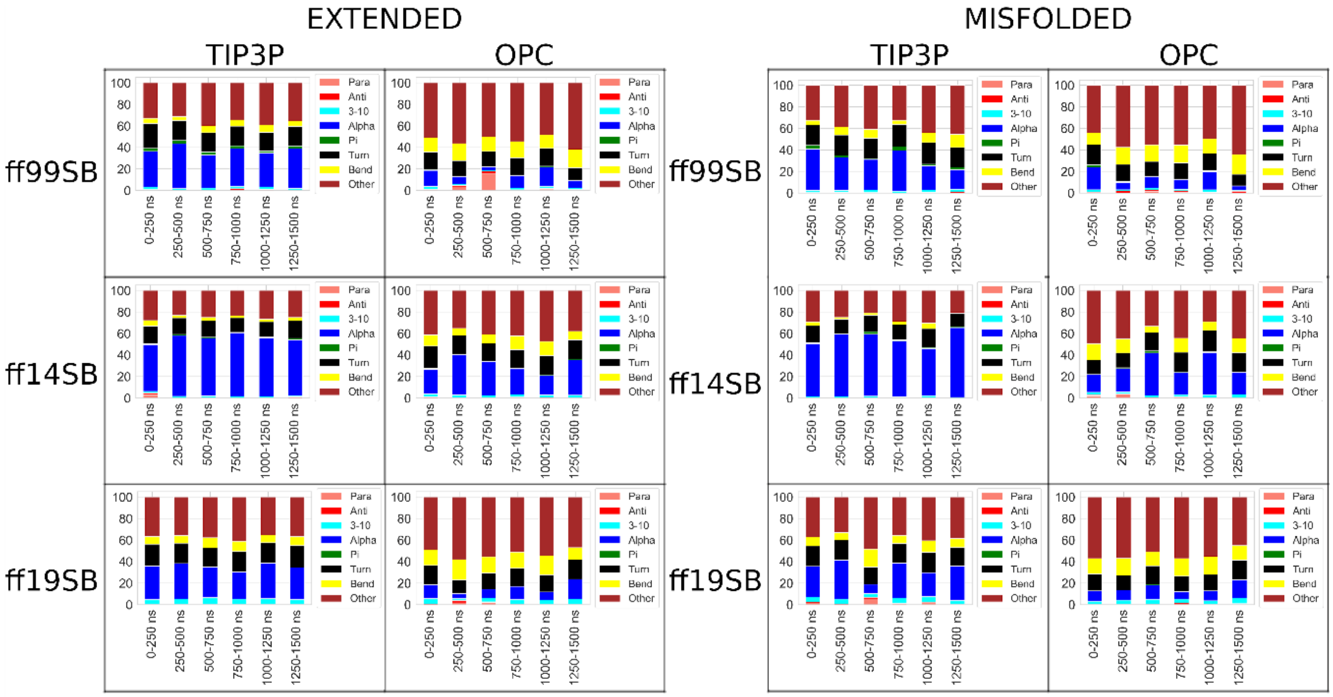

Figure S14. ID1 DSSP for extended (left) and misfolded (right) aMDs, calculated at 250 ns intervals.

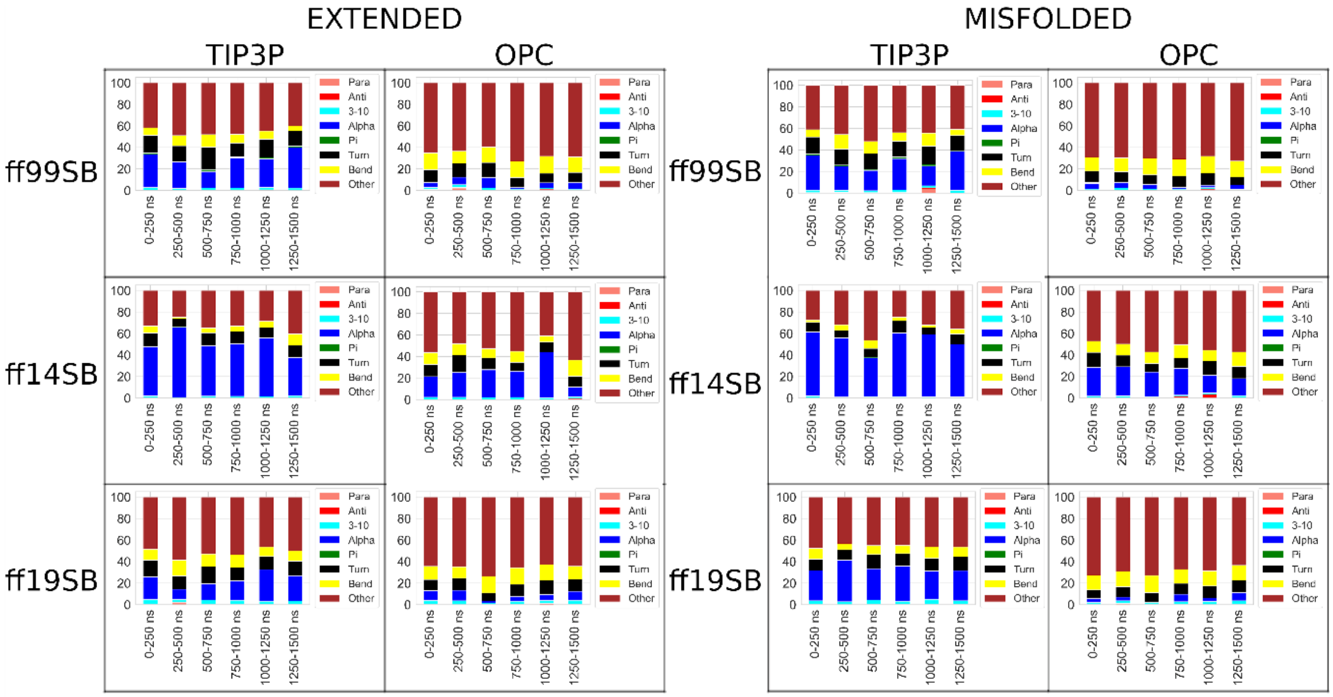

Figure S15. ID2 DSSP for extended (left) and misfolded (right) aMDs, calculated at 250 ns intervals.

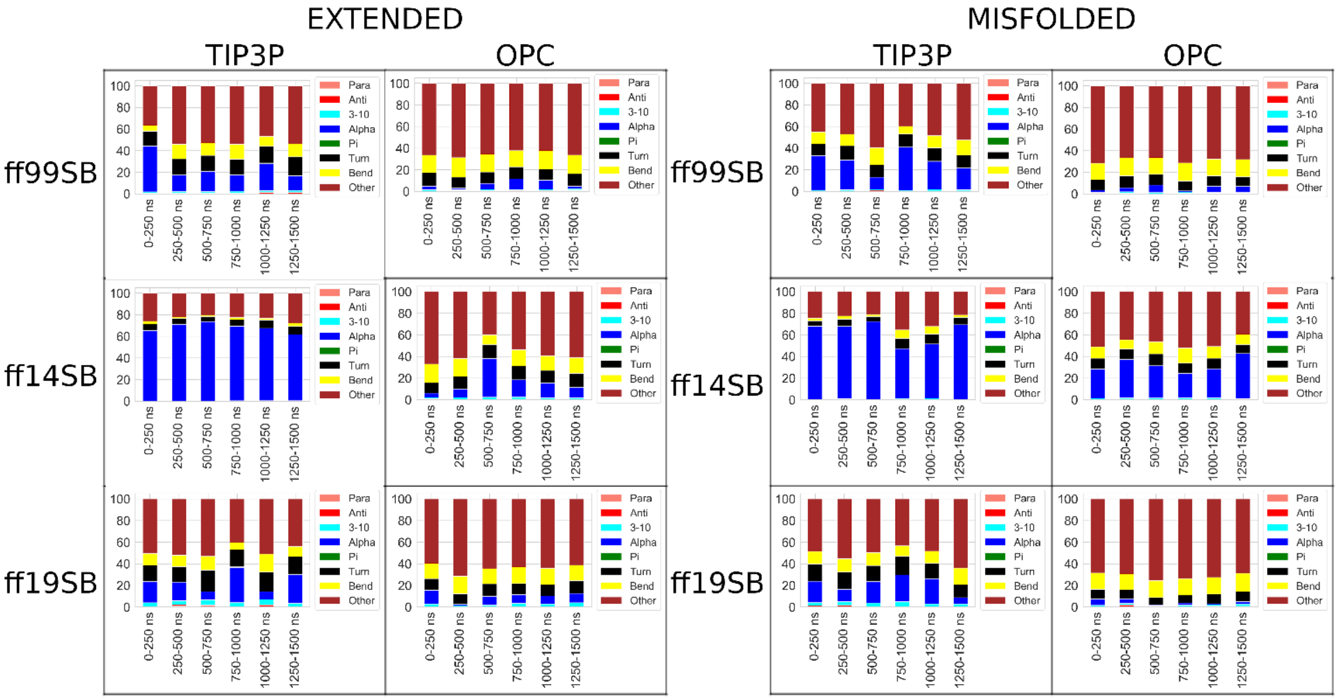

Figure S16. ID3 DSSP for extended (left) and misfolded (right) aMDs, calculated at 250 ns intervals.

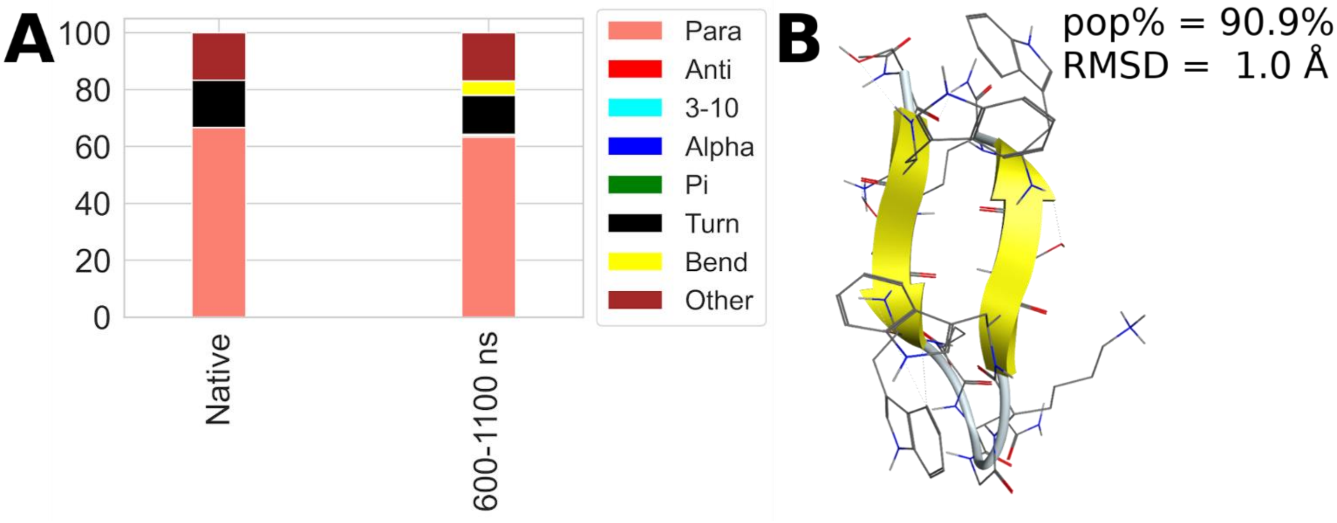

Figure S17. DSSP and main cluster of ff19SB/TIP3P B2 simulation starting from misfolded conformation. The 600 – 1100 ns time interval was considered for the analysis. Population (pop%) and RMSD compared to native structure are also shown.

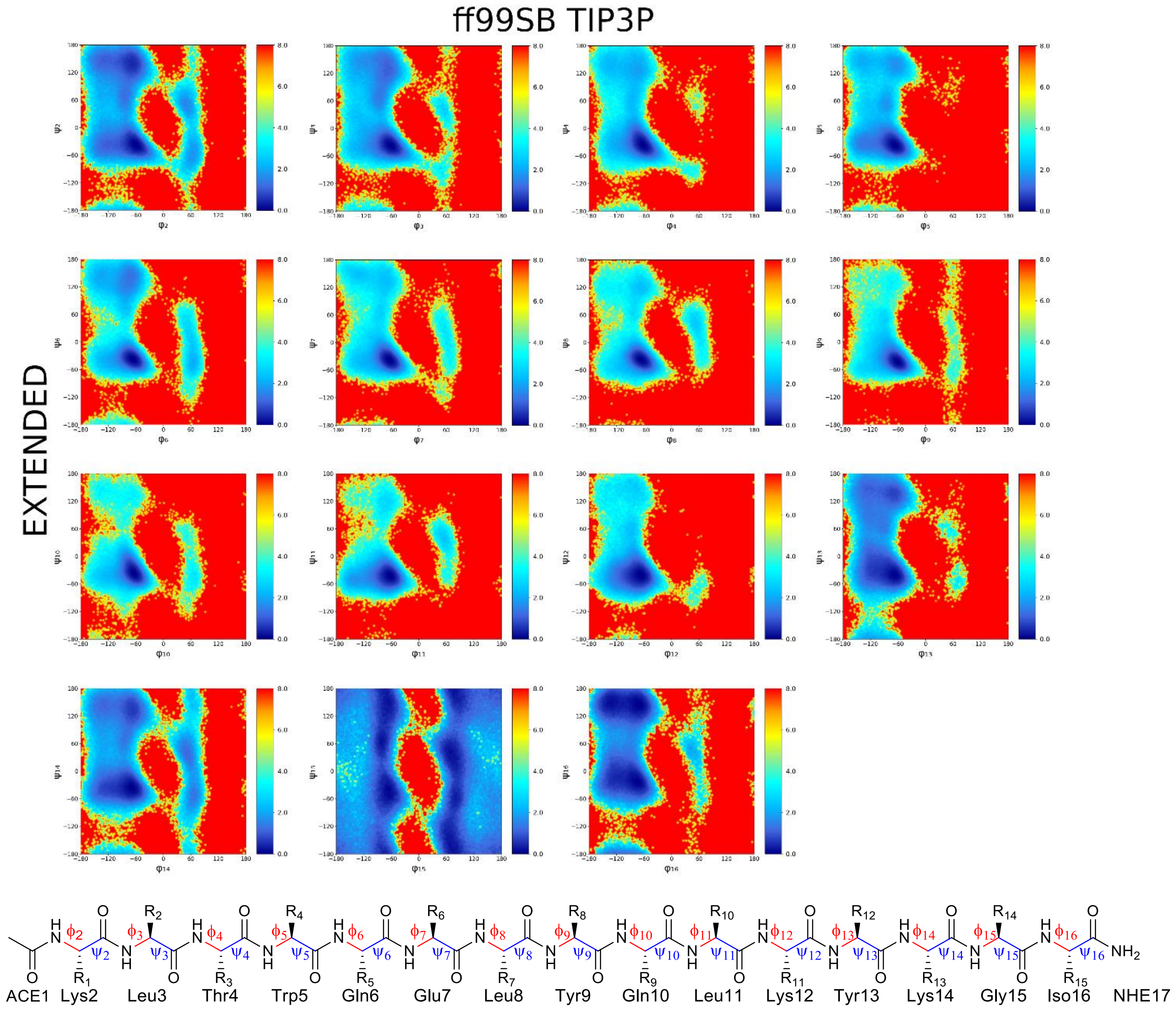

Figure S18. PMFs obtained from φ and ψ dihedral distributions of H1 residues for the ff99SB/TIP3P combination from an extended conformation. PMFs are shown as heat maps (0.0 – 8.0 kcal/mol range) from dark blue to red. H1 sequence and representation of φ and ψ angles numbering are also shown.

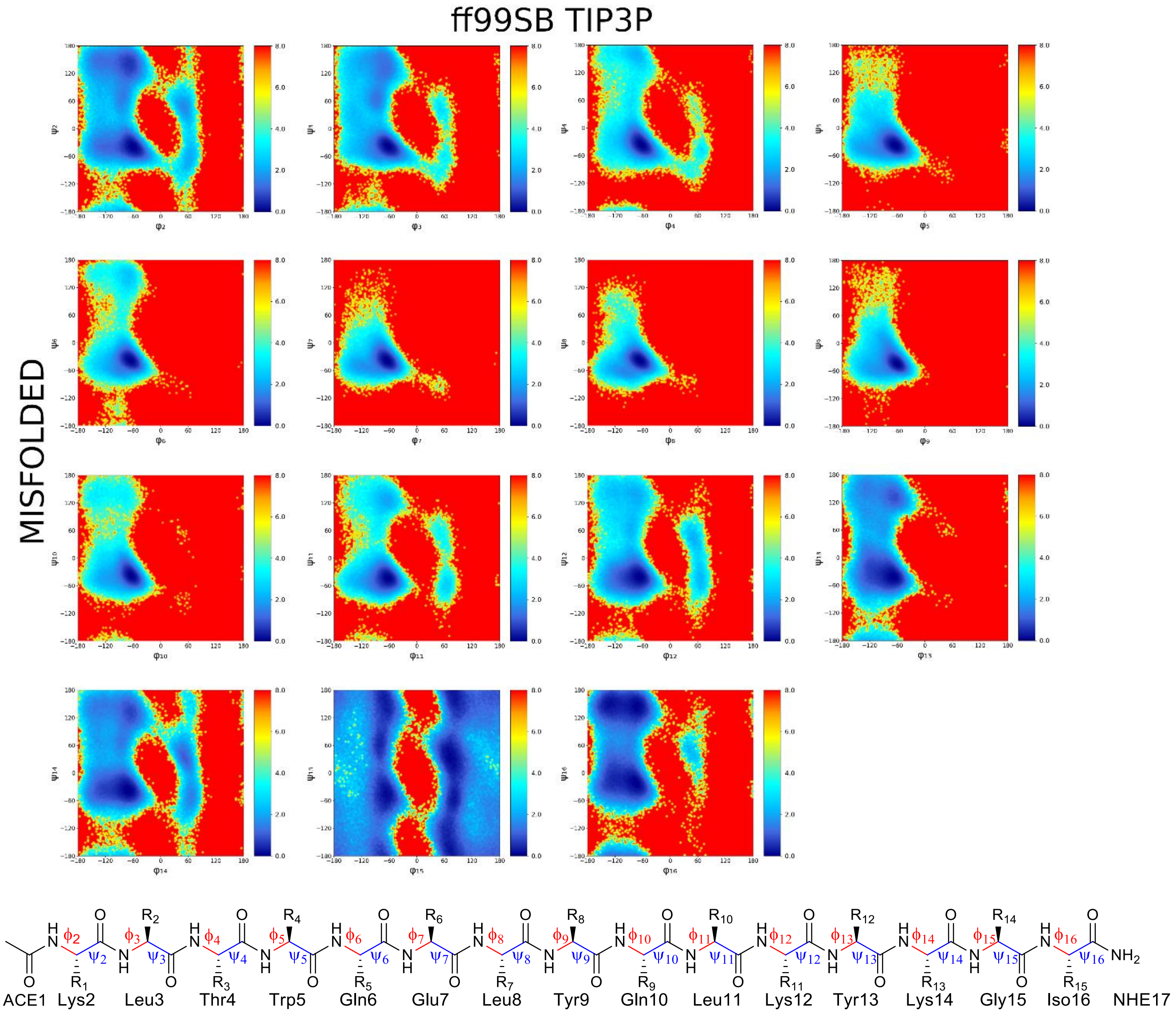

Figure S19. PMFs obtained from φ and ψ dihedral distributions of H1 residues for the ff99SB/TIP3P combination from a misfolded conformation. PMFs are shown as heat maps (0.0 – 8.0 kcal/mol range) from dark blue to red. H1 sequence and representation of φ and ψ angles numbering are also shown.

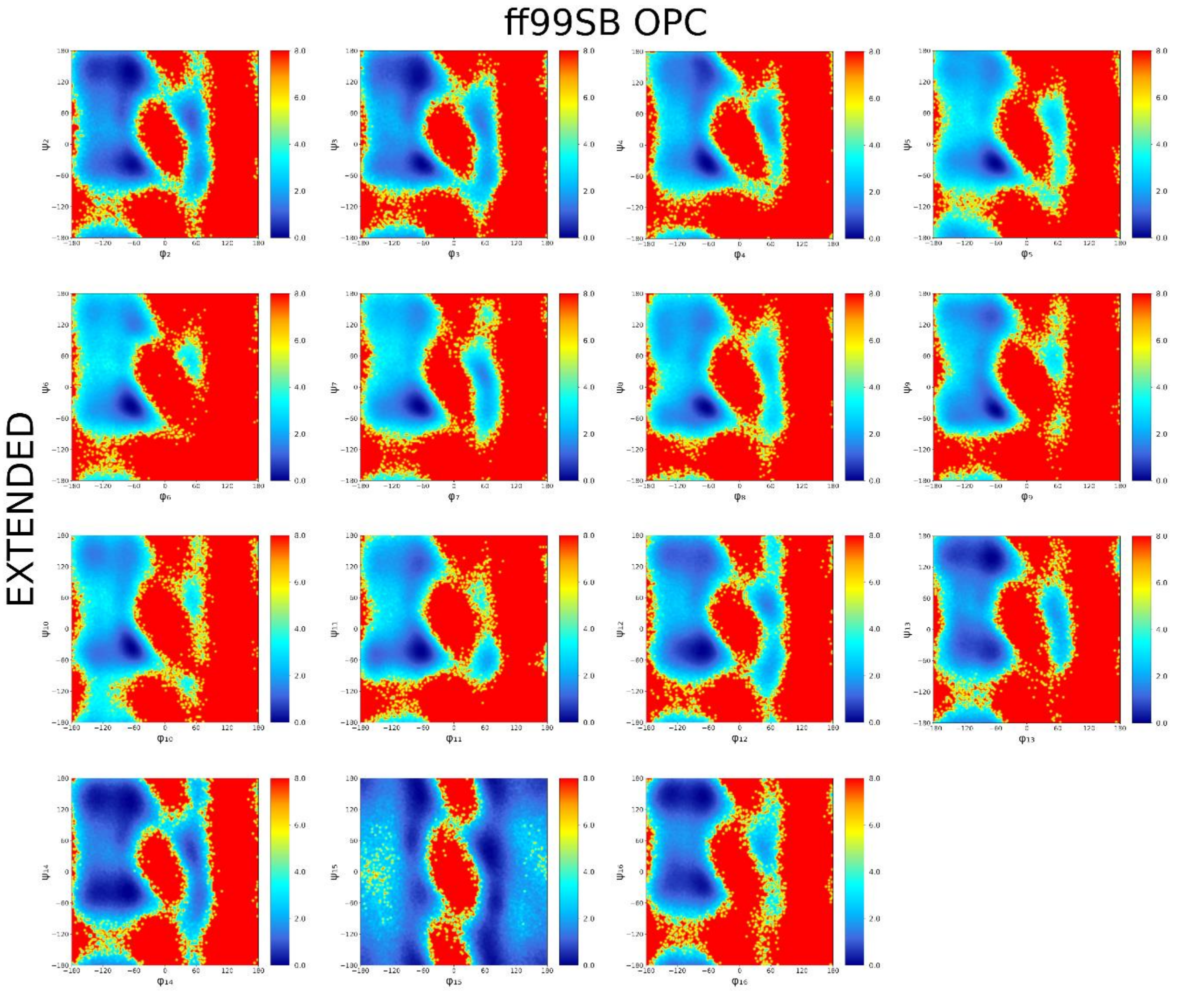

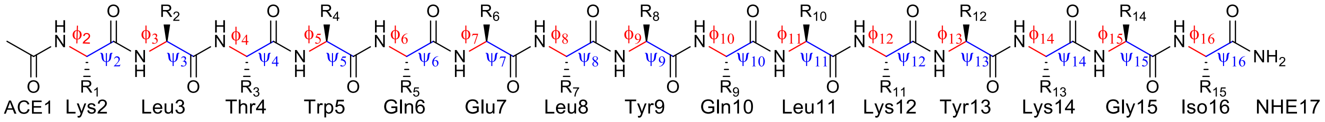

Figure S20. PMFs obtained from φ and ψ dihedral distributions of H1 residues for the ff99SB/OPC combination from an extended conformation. PMFs are shown as heat maps (0.0 – 8.0 kcal/mol range) from dark blue to red. H1 sequence and representation of φ and ψ angles numbering are also shown.

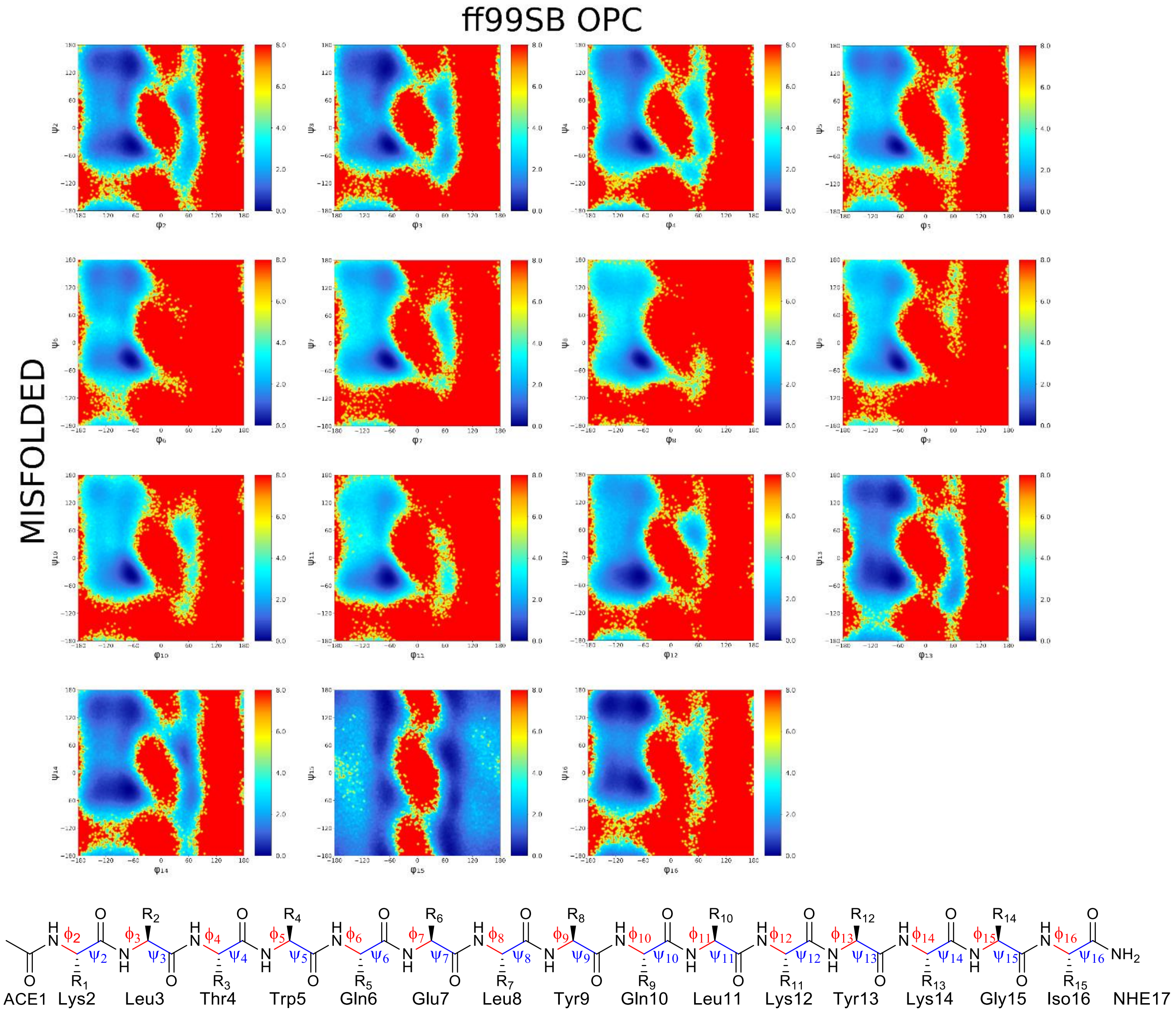

Figure S21. PMFs obtained from φ and ψ dihedral distributions of H1 residues for the ff99SB/OPC combination from a misfolded conformation. PMFs are shown as heat maps (0.0 – 8.0 kcal/mol range) from dark blue to red. H1 sequence and representation of φ and ψ angles numbering are also shown.

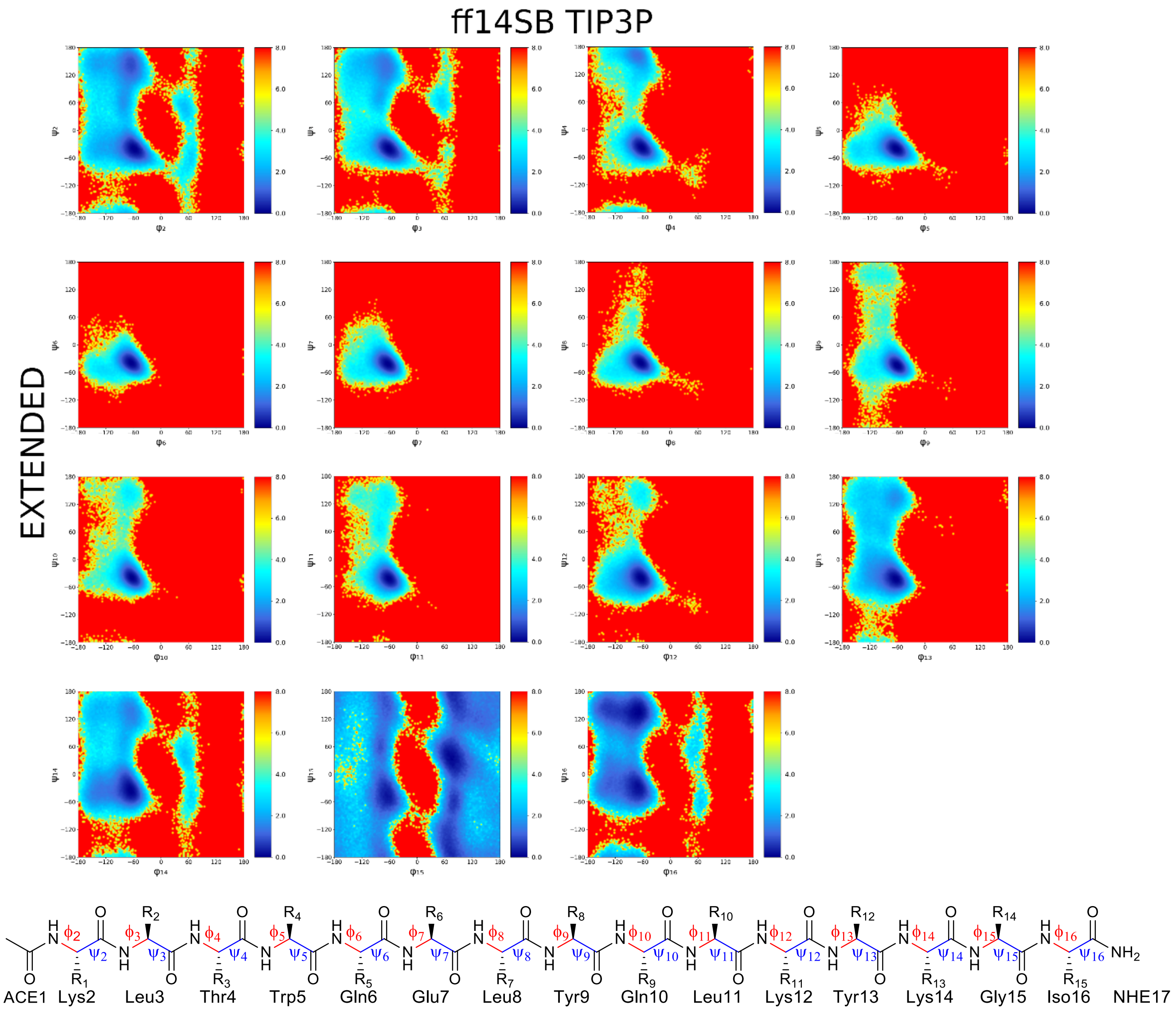

Figure S22. PMFs obtained from φ and ψ dihedral distributions of H1 residues for the ff14SB/TIP3P combination from an extended conformation. PMFs are shown as heat maps (0.0 – 8.0 kcal/mol range) from dark blue to red. H1 sequence and representation of φ and ψ angles numbering are also shown.

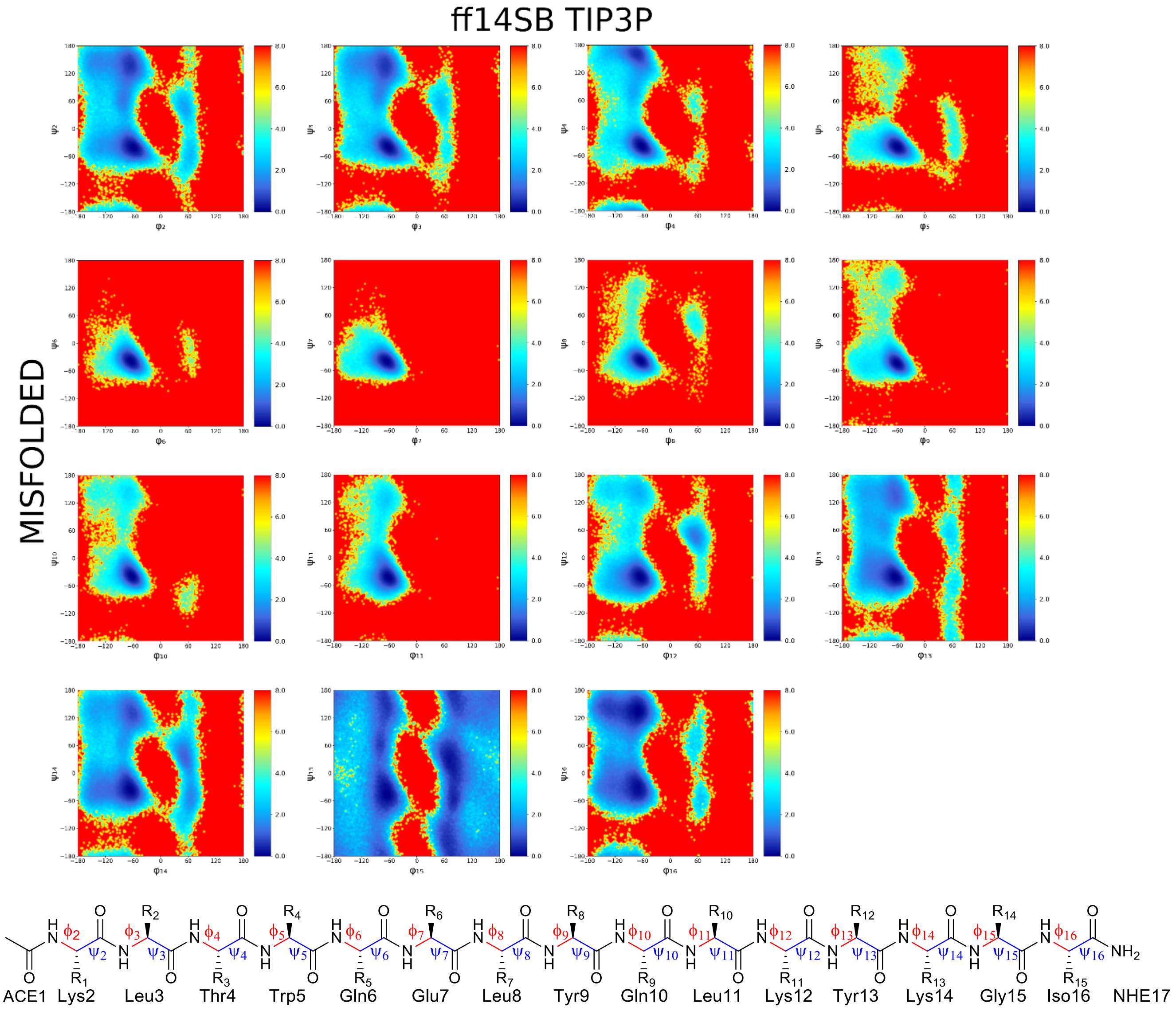

Figure S23. PMFs obtained from φ and ψ dihedral distributions of H1 residues for the ff14SB/TIP3P combination from a misfolded conformation. PMFs are shown as heat maps (0.0 – 8.0 kcal/mol range) from dark blue to red. H1 sequence and representation of φ and ψ angles numbering are also shown.

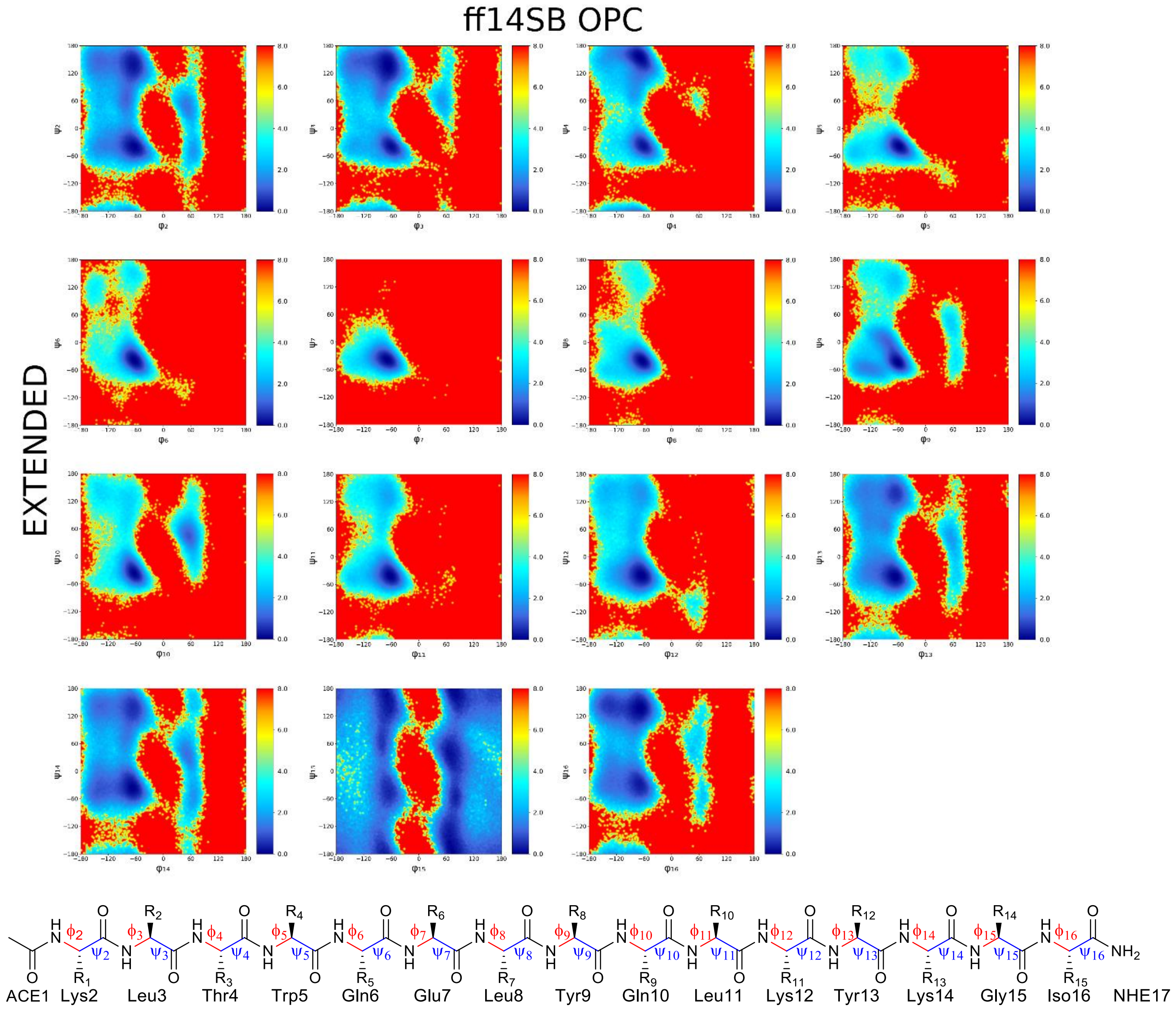

Figure S24. PMFs obtained from φ and ψ dihedral distributions of H1 residues for the ff14SB/OPC combination from an extended conformation. PMFs are shown as heat maps (0.0 – 8.0 kcal/mol range) from dark blue to red. H1 sequence and representation of φ and ψ angles numbering are also shown.

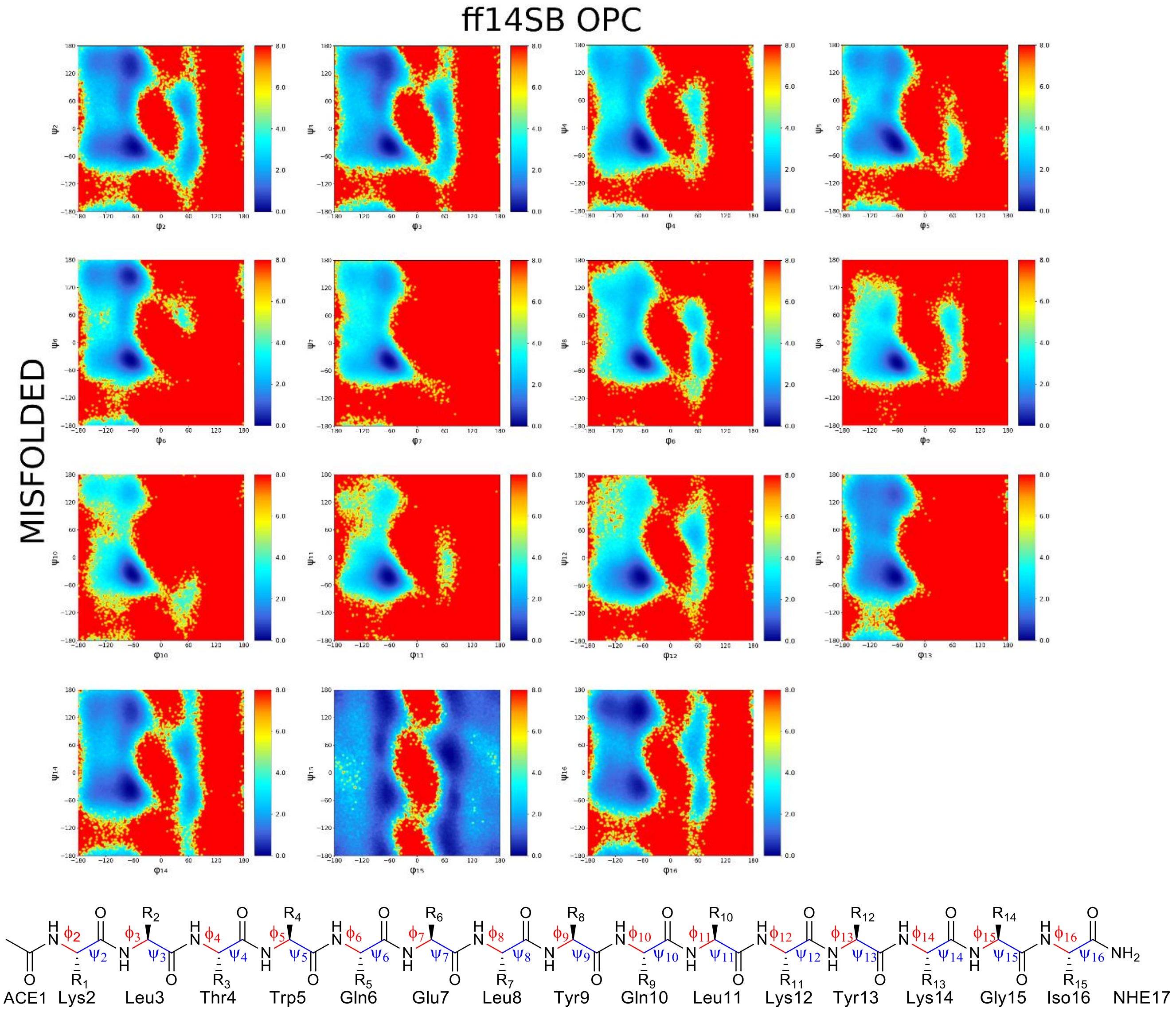

Figure S25. PMFs obtained from φ and ψ dihedral distributions of H1 residues for the ff14SB/OPC combination from a misfolded conformation. PMFs are shown as heat maps (0.0 – 8.0 kcal/mol range) from dark blue to red. H1 sequence and representation of φ and ψ angles numbering are also shown.

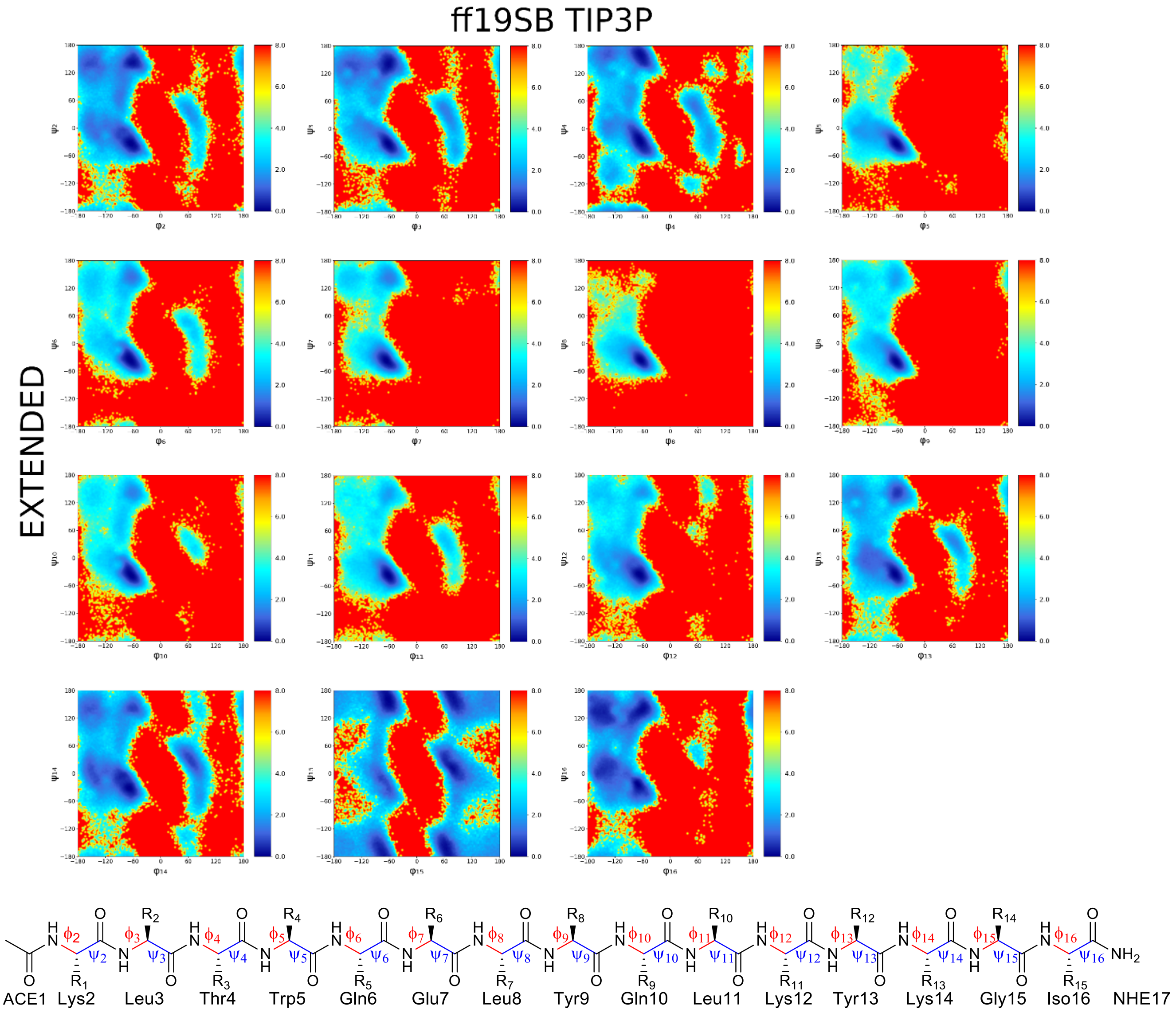

Figure S26. PMFs obtained from φ and ψ dihedral distributions of H1 residues for the ff19SB/TIP3P combination from an extended conformation. PMFs are shown as heat maps (0.0 – 8.0 kcal/mol range) from dark blue to red. H1 sequence and representation of φ and ψ angles numbering are also shown.

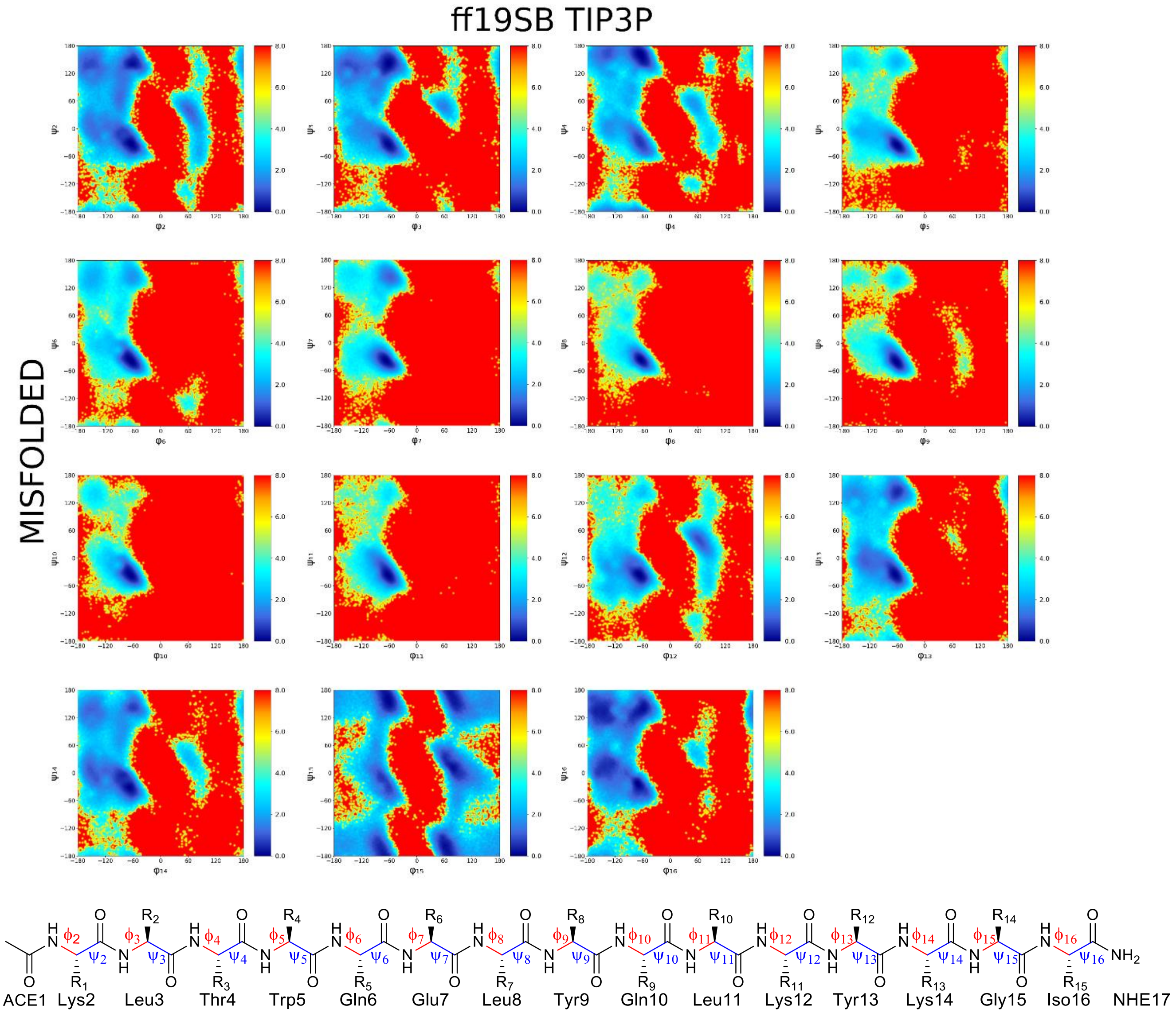

Figure S27. PMFs obtained from φ and ψ dihedral distributions of H1 residues for the ff19SB/TIP3P combination from a misfolded conformation. PMFs are shown as heat maps (0.0 – 8.0 kcal/mol range) from dark blue to red. H1 sequence and representation of φ and ψ angles numbering are also shown.

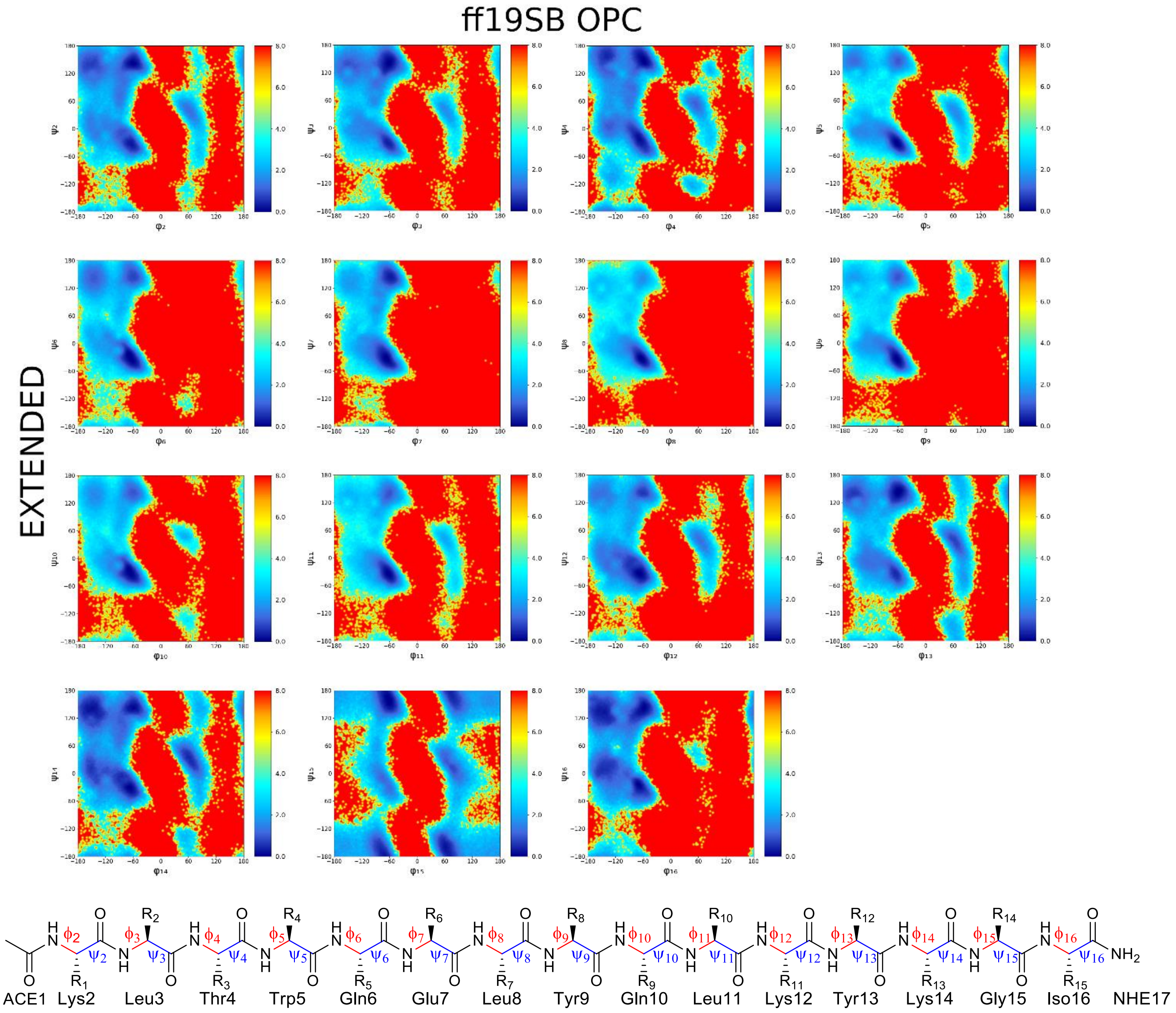

Figure S28. PMFs obtained from φ and ψ dihedral distributions of H1 residues for the ff19SB/OPC combination from an extended conformation. PMFs are shown as heat maps (0.0 – 8.0 kcal/mol range) from dark blue to red. H1 sequence and representation of φ and ψ angles numbering are also shown.

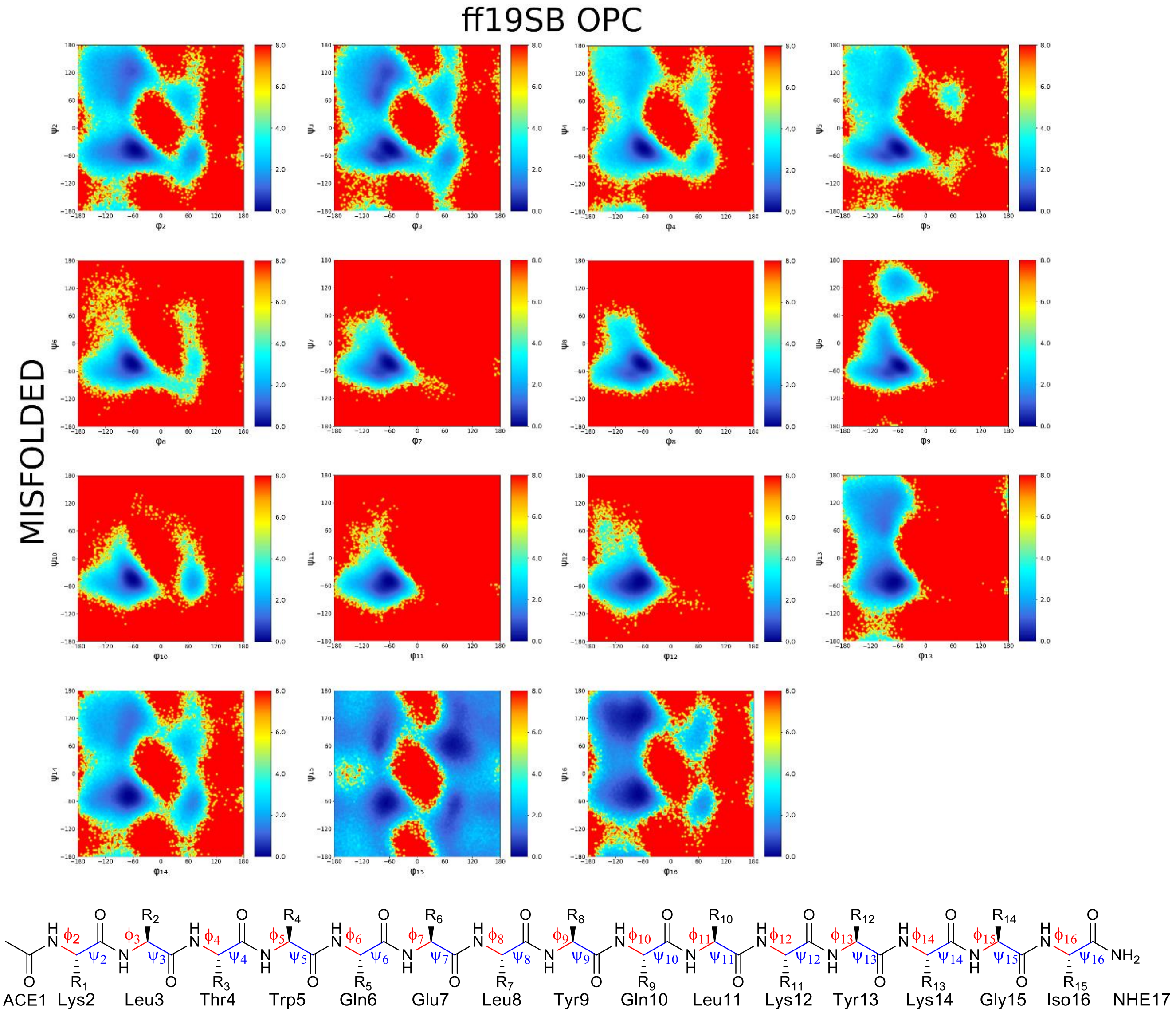

Figure S29. PMFs obtained from φ and ψ dihedral distributions of H1 residues for the ff19SB/OPC combination from a misfolded conformation. PMFs are shown as heat maps (0.0 – 8.0 kcal/mol range) from dark blue to red. H1 sequence and representation of φ and ψ angles numbering are also shown.

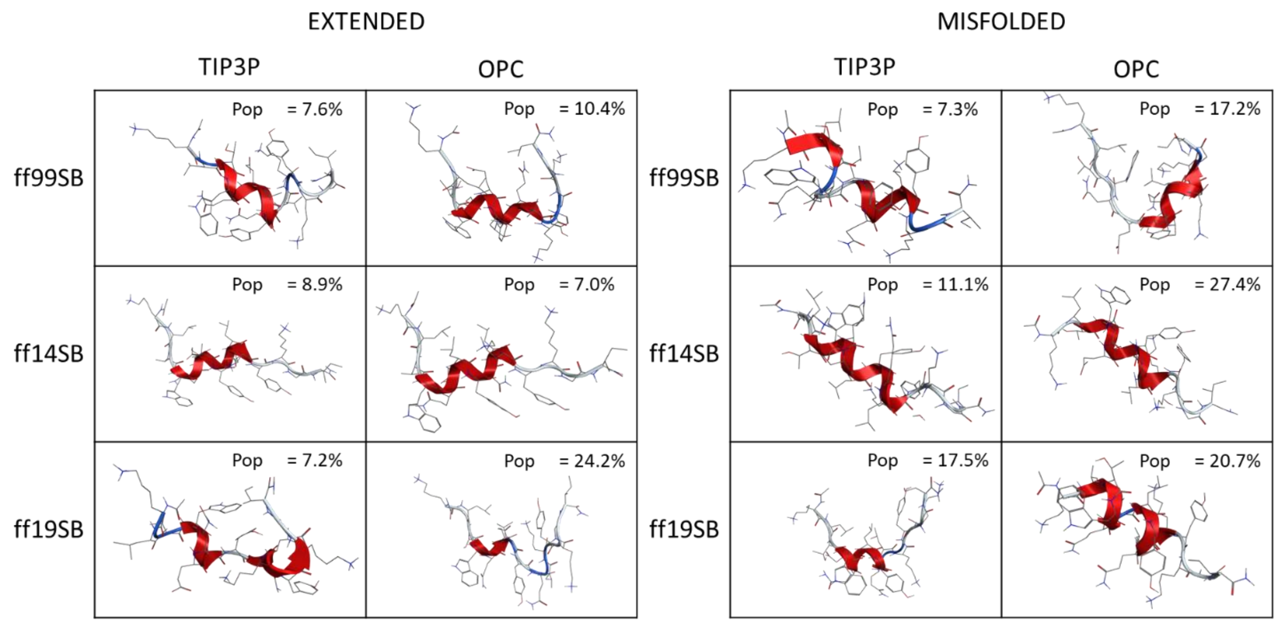

Figure S30. Representative conformation and population (pop%) of the second clusters from H1 trajectories.

Table S3. H-bond analyses from H1 trajectories. Only H-bonds involving the backbone are included in the table (donor N-H, acceptor C=O).

ff99SB OPC

EXTENDED			MISFOLDED		
acceptor	donor	occ%	acceptor	donor	occ%
TRP5	TYR9	23.59	LEU8	LYS12	25.94
THR4	LEU8	17.75	GLU7	LEU11	23.66
LEU8	LYS12	16.61	TYR9	TYR13	21.97
TYR9	TYR13	15.98	TRP5	TYR9	19.9
GLN6	GLN10	15.12	GLN6	GLN10	18.21
GLU7	LEU11	13.93	THR4	LEU8	12.88
LEU3	GLU7	11.74	GLN10	LYS14	12.72
THR4	GLU7	7.01	LEU3	GLU7	8.93
GLN10	LYS14	6.55	TYR9	LYS14	7.53
LYS2	GLN6	6.45	GLU7	GLN10	7.44
LEU3	GLN6	5.73	LYS2	GLN6	7.37
			GLN10	GLY15	6.51

ff99SB TIP3P

EXTENDED			MISFOLDED		
acceptor	donor	occ%	acceptor	donor	occ%
TYR9	TYR13	35.57	TRP5	TYR9	35.42
LEU8	LYS12	33.16	LEU8	LYS12	35.15
GLU7	LEU11	28.72	LEU3	GLU7	31.74
GLN6	GLN10	27.68	TYR9	TYR13	31.54
TRP5	TYR9	23.62	GLU7	LEU11	29.96
LEU3	GLU7	21.77	GLN6	GLN10	28.9
GLN10	LYS14	17.63	THR4	LEU8	23.33
LYS2	GLN6	16.01	GLN10	LYS14	19.72
THR4	LEU8	15.36	LYS2	GLN6	18.57
LEU3	GLN6	9.2	TYR9	LYS14	13.64
TYR9	LYS14	7.89	GLN10	GLY15	10.84
LEU11	GLY15	7.52	ACE1	TRP5	10.64
GLN10	GLY15	7.52	LEU3	GLN6	10.44
ACE1	TRP5	6.78	LEU11	GLY15	8.18
LYS2	TRP5	6.53	GLN6	LEU11	6.47
			LYS2	TRP5	5.57
			TRP5	GLN10	5.28
			LEU8	TYR13	5.13

ff14SB OPC

ff14SB TIP3P

EXTENDED		
acceptor	donor	occ%
TRP5	TYR9	46.2
THR4	LEU8	37.06
TYR9	TYR13	30.25
GLN10	LYS14	21.33
LEU8	LYS12	20.8
GLU7	LEU11	19.11
GLN6	GLN10	19.06
GLN6	LEU11	12.41
THR4	GLU7	11.72
LEU3	GLU7	8.91
LYS2	GLN6	7.74
LEU11	GLY15	7.65
GLN10	GLY15	6.36

MISFOLDED		
acceptor	donor	occ%
TYR9	TYR13	37.81
LEU8	LYS12	29.34
GLU7	LEU11	26.09
GLN6	GLN10	23.74
GLN10	LYS14	20.66
TRP5	TYR9	12.72
LEU3	GLN6	10.95
LEU3	GLU7	10.51
THR4	LEU8	10.07
LEU11	GLY15	9.34
GLN6	TYR9	8.11
LYS2	GLN6	7.7
TYR9	LYS14	5.53
TYR9	LYS12	5.5
GLN10	GLY15	5.37
ACE1	TRP5	5.33

EXTENDED		
acceptor	donor	occ%
TRP5	TYR9	46.13
TYR9	TYR13	45.18
GLU7	LEU11	39.23
LEU8	LYS12	38.88
GLN10	LYS14	35.65
GLN6	GLN10	35.29
THR4	LEU8	31.24
LEU3	GLU7	31.11
LYS2	GLN6	25.18
LEU11	GLY15	18.67
ACE1	TRP5	15.82
GLN10	GLY15	7.97
LEU11	ILE16	7.1

MISFOLDED		
acceptor	donor	occ%
TRP5	TYR9	49.83
TYR9	TYR13	40.46
GLU7	LEU11	39.56
LEU8	LYS12	37.93
THR4	LEU8	34.15
GLN6	GLN10	33.25
GLN10	LYS14	26.96
LEU3	GLU7	19.75
LYS2	GLN6	15.38
LEU11	GLY15	10.46
ACE1	TRP5	10.39
GLN10	GLY15	7.91
THR4	GLU7	7.42
TYR9	LYS14	6

ff19SB OPC					
EXTENDED			MISFOLDED		
acceptor	donor	occ%	acceptor	donor	occ%
LEU8	LYS12	14.25	LEU8	LYS12	34.48
TYR9	TYR13	14.02	TRP5	TYR9	32.19
GLU7	LEU11	13.2	TYR9	LYS14	26.75
TRP5	TYR9	12.92	LEU3	GLU7	25.95
GLU7	GLN10	11.43	TYR9	TYR13	25.33
THR4	LEU8	9.88	GLU7	LEU11	24.86
TYR9	LYS12	8.26	GLN6	GLN10	23.14
GLN6	GLN10	8.07	THR4	LEU8	22.81
THR4	GLU7	7.32	TRP5	GLN10	17.07
GLN10	LYS14	7.11	LEU8	TYR13	16.75
GLN10	TYR13	5.5	LYS2	GLN6	15.89
			GLN10	GLY15	15.7
			ACE1	TRP5	14.54
			GLN10	LYS14	13.21
			GLU7	LYS12	11.39
			GLN6	LEU11	11.02
			LEU3	LEU8	10.15
			ACE1	GLN6	8
			LEU11	ILE16	7.72
			THR4	TYR9	7.66
			LEU11	GLY15	7.02
			GLN10	ILE16	5.63
			LEU3	GLN6	5.45

ff19SB TIP3P					
EXTENDED			MISFOLDED		
acceptor	donor	occ%	acceptor	donor	occ%
TRP5	TYR9	31.45	GLU7	LEU11	39.46
GLU7	LEU11	29.39	TRP5	TYR9	37.55
TYR9	TYR13	26.5	LEU8	LYS12	30.56
LEU8	LYS12	24.05	TYR9	TYR13	27.23
THR4	LEU8	21.95	THR4	LEU8	27
GLN6	GLN10	21.9	GLN10	LYS14	21.8
GLN10	LYS14	20.12	GLN6	GLN10	20.3
LEU11	GLY15	9.3	GLU7	GLN10	9.53
THR4	GLU7	7.78	LEU11	GLY15	9.03
LEU3	GLU7	7.56	THR4	GLU7	8.81
LYS2	GLN6	6.9	TYR9	LYS12	6.21
LEU3	GLN6	6.75	LYS2	GLN6	5.35
GLN10	TYR13	5.99			
GLU7	GLN10	5.82			
TYR9	LYS12	5.74			

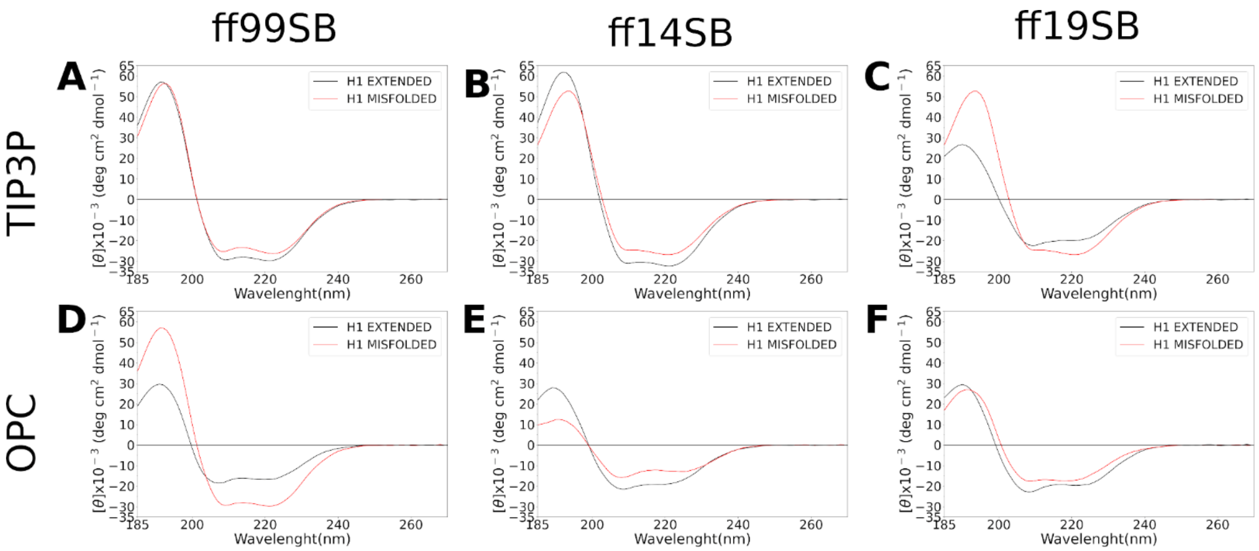

Figure 31. SESCA CD spectra of H1 calculated using the main cluster obtained from the last 500 ns of aMD trajectory, for each combination. Results from extended and misfolded simulations are reported in black and red lines, respectively.

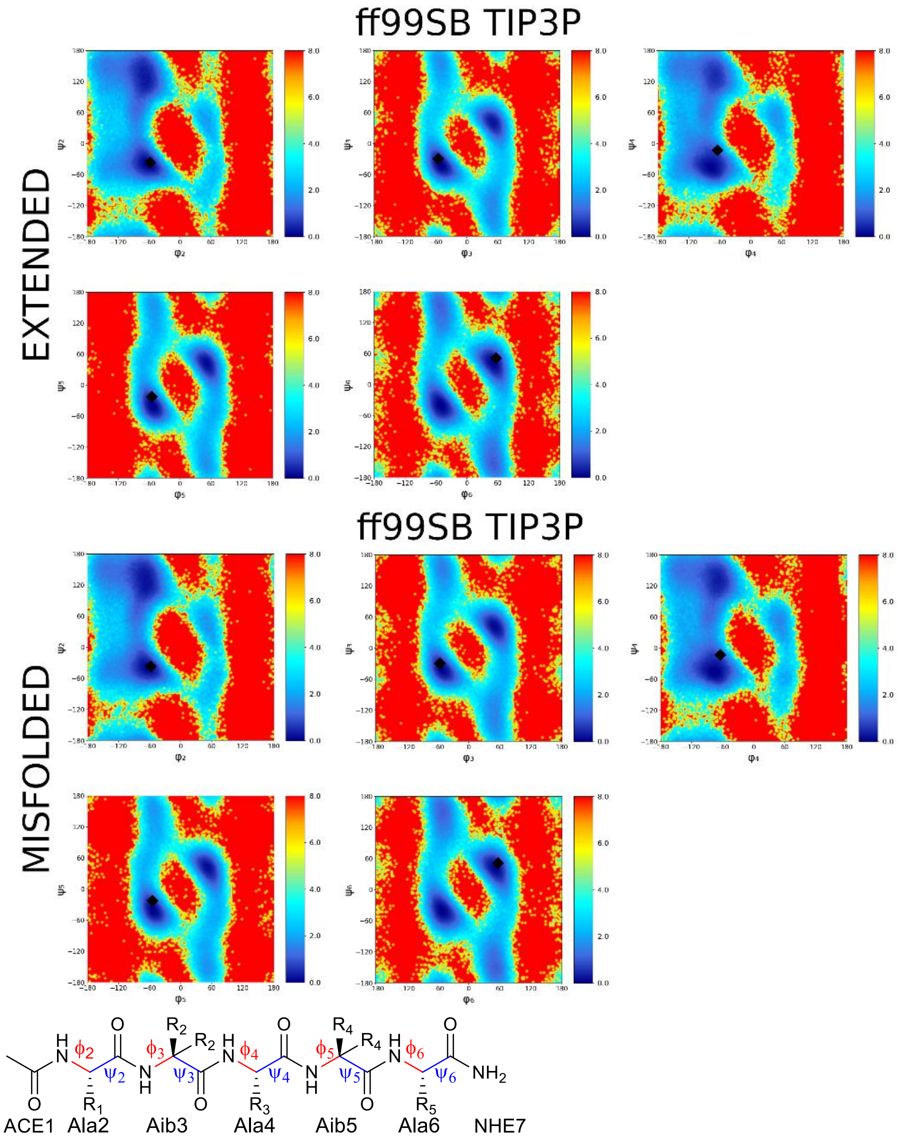

Figure S32. PMFs obtained from φ and ψ dihedral distributions of H2 residues for the ff99SB/TIP3P combination from extended (top panels) and misfolded (bottom panels) conformations. PMFs are shown as heat maps (0.0 – 8.0 kcal/mol range) from dark blue to red. The native conformation's φ and ψ angles are reported as black diamonds. H2 sequence and representation of φ and ψ angles numbering are also shown.

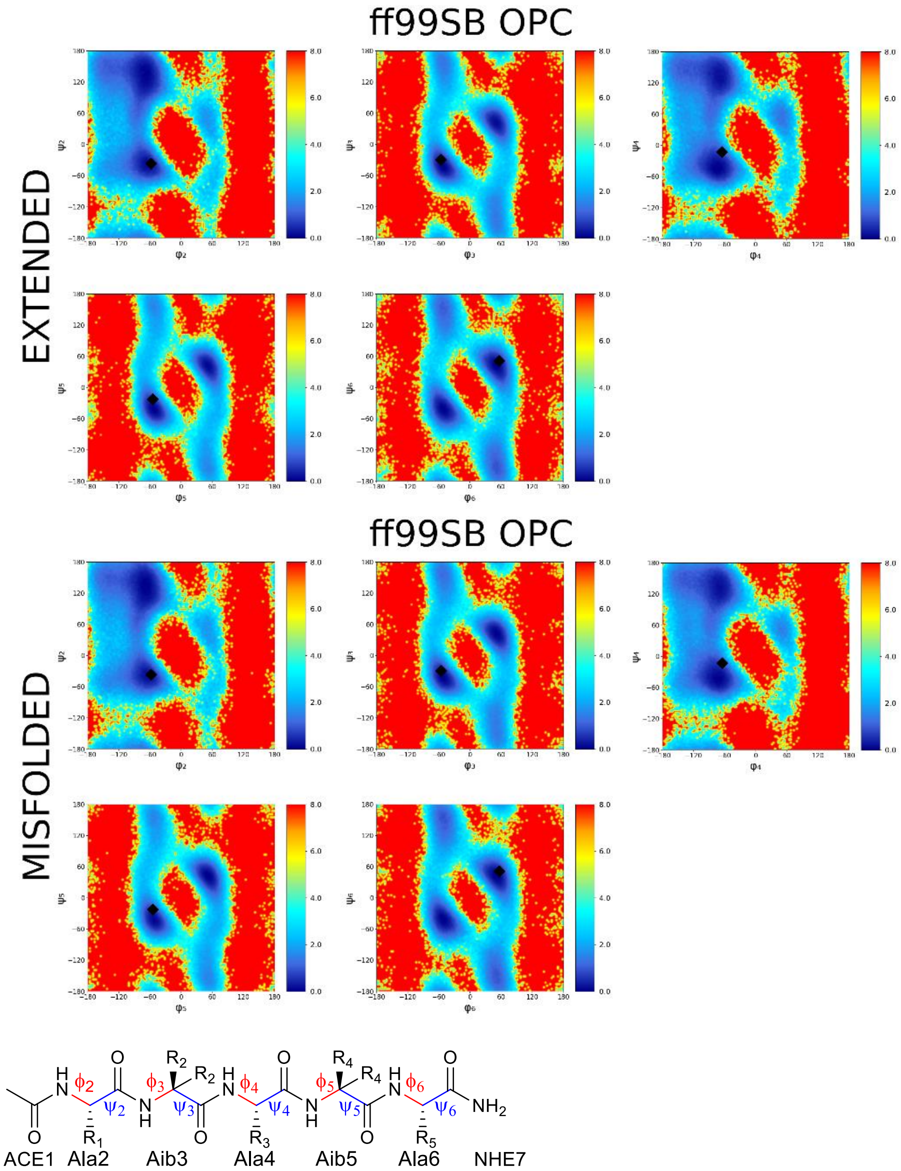

Figure S33. PMFs obtained from φ and ψ dihedral distributions of H2 residues for the ff99SB/OPC combination from extended (top panels) and misfolded (bottom panels) conformations. PMFs are shown as heat maps (0.0 – 8.0 kcal/mol range) from dark blue to red. The native conformation's φ and ψ angles are reported as black diamonds. H2 sequence and representation of φ and ψ angles numbering are also shown.

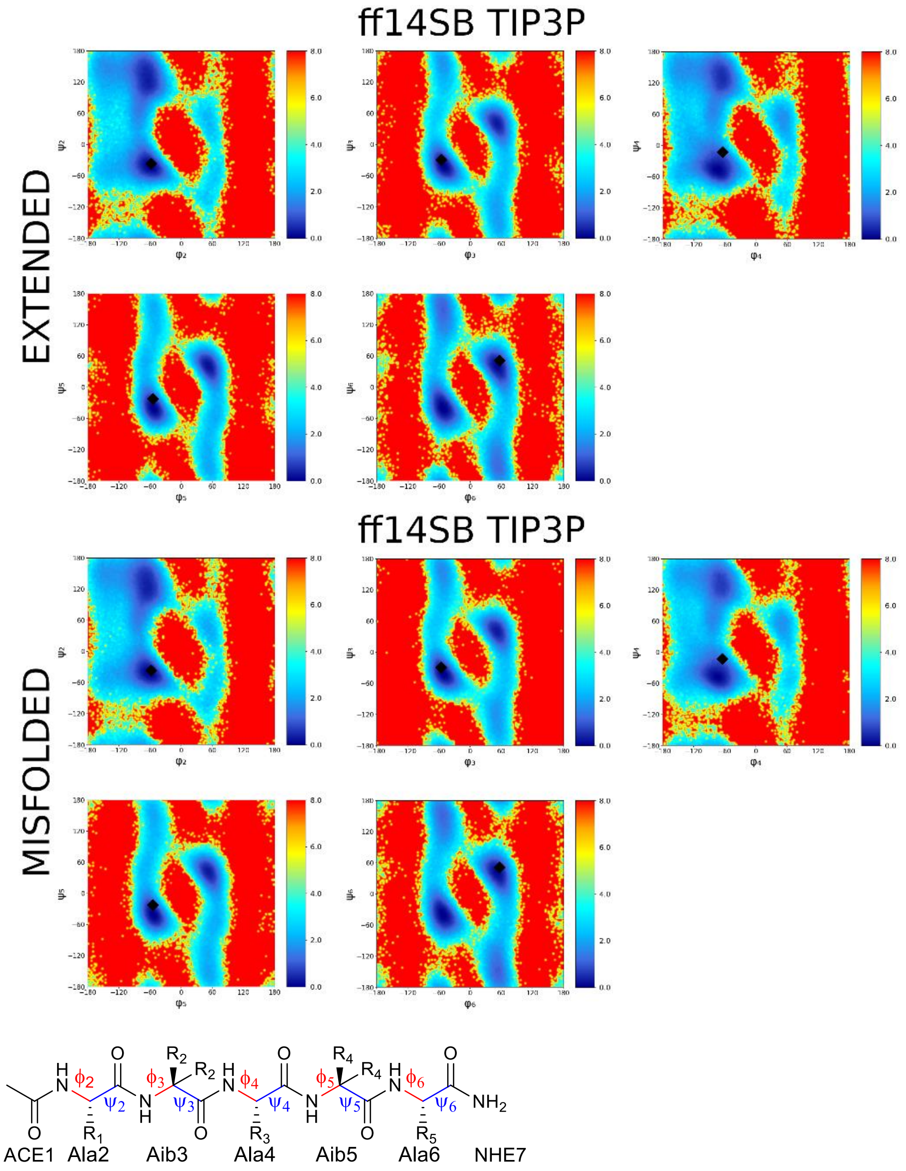

Figure S34. PMFs obtained from φ and ψ dihedral distributions of H2 residues for the ff14SB/TIP3P combination from extended (top panels) and misfolded (bottom panels) conformations. PMFs are shown as heat maps (0.0 – 8.0 kcal/mol range) from dark blue to red. The native conformation's φ and ψ angles are reported as black diamonds. H2 sequence and representation of φ and ψ angles numbering are also shown.

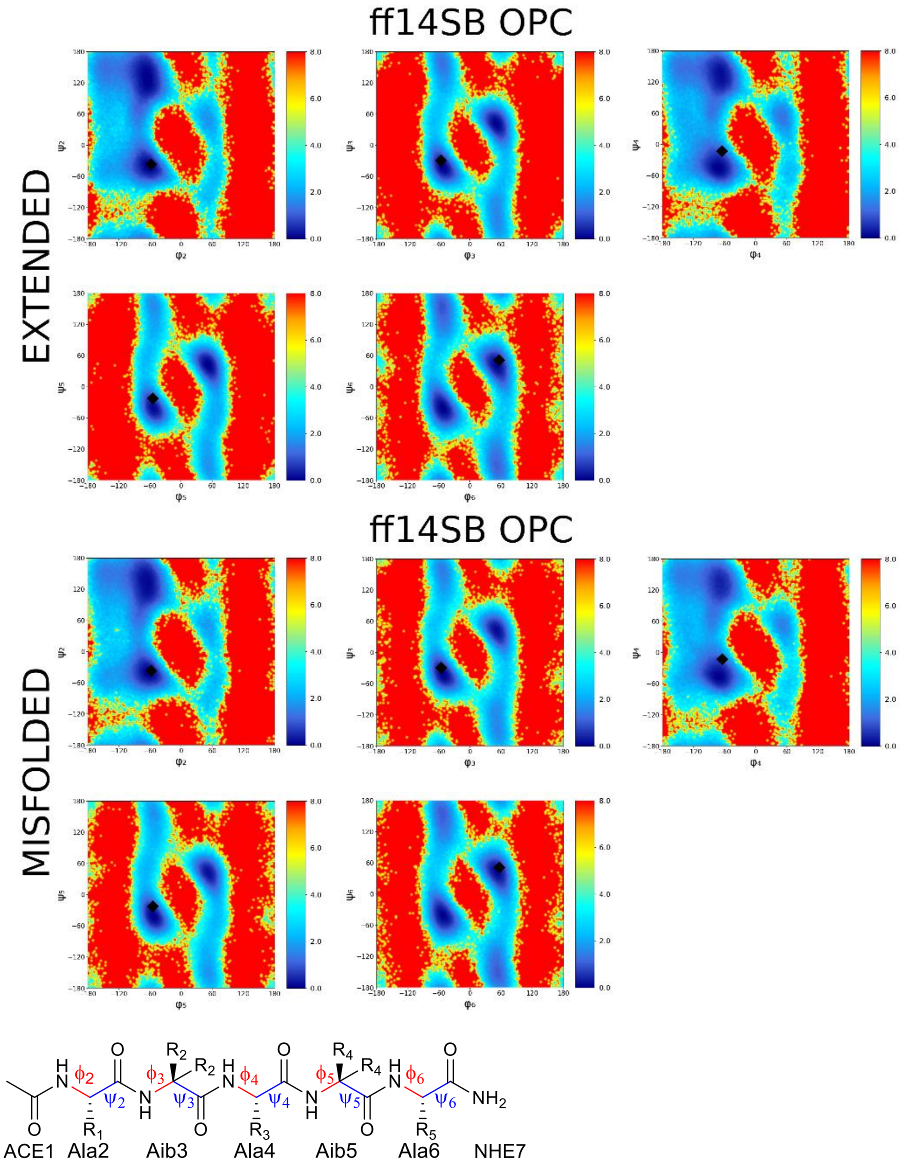

Figure S35. PMFs obtained from φ and ψ dihedral distributions of H2 residues for the ff14SB/OPC combination from extended (top panels) and misfolded (bottom panels) conformations. PMFs are shown as heat maps (0.0 – 8.0 kcal/mol range) from dark blue to red. The native conformation's φ and ψ angles are reported as black diamonds. H2 sequence and representation of φ and ψ angles numbering are also shown.

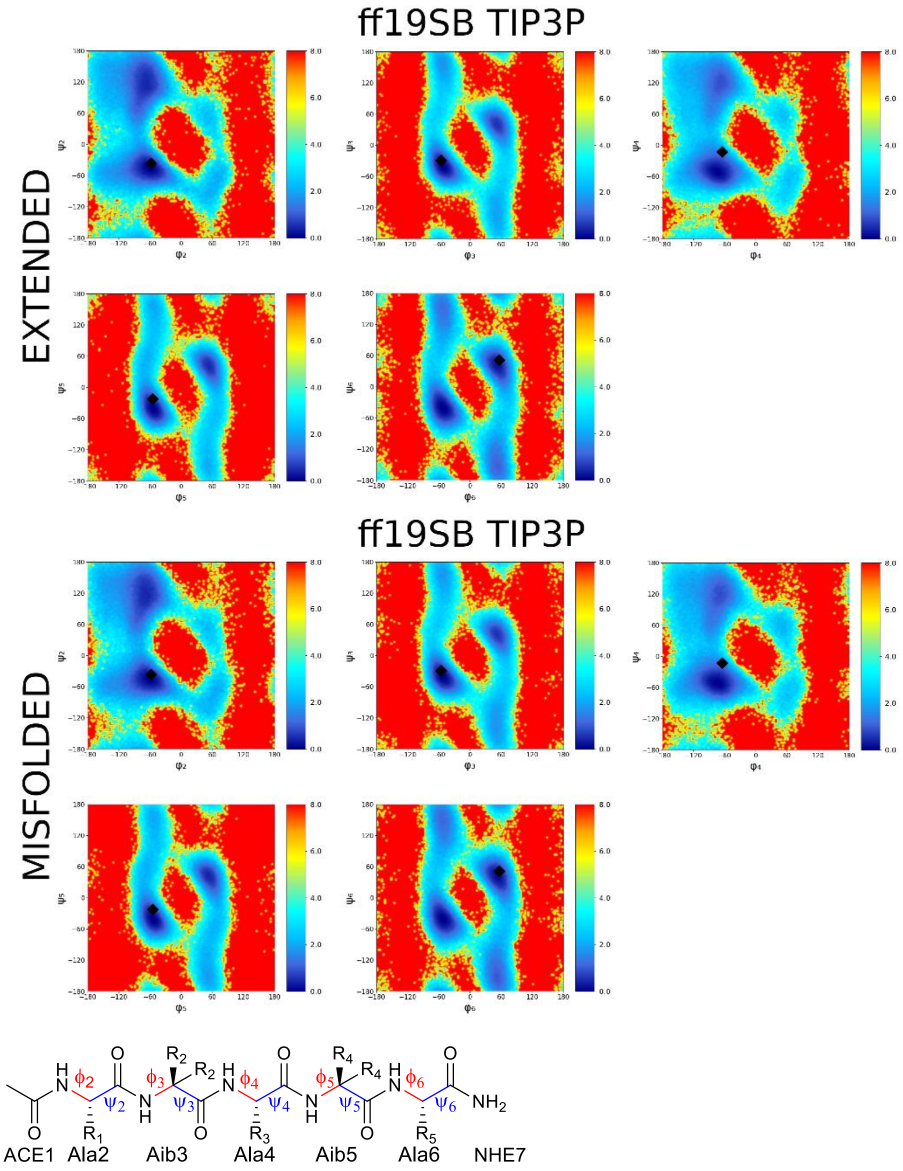

Figure S36. PMFs obtained from φ and ψ dihedral distributions of H2 residues for the ff19SB/TIP3P combination from extended (top panels) and misfolded (bottom panels) conformations. PMFs are shown as heat maps (0.0 – 8.0 kcal/mol range) from dark blue to red. The native conformation's φ and ψ angles are reported as black diamonds. H2 sequence and representation of φ and ψ angles numbering are also shown.

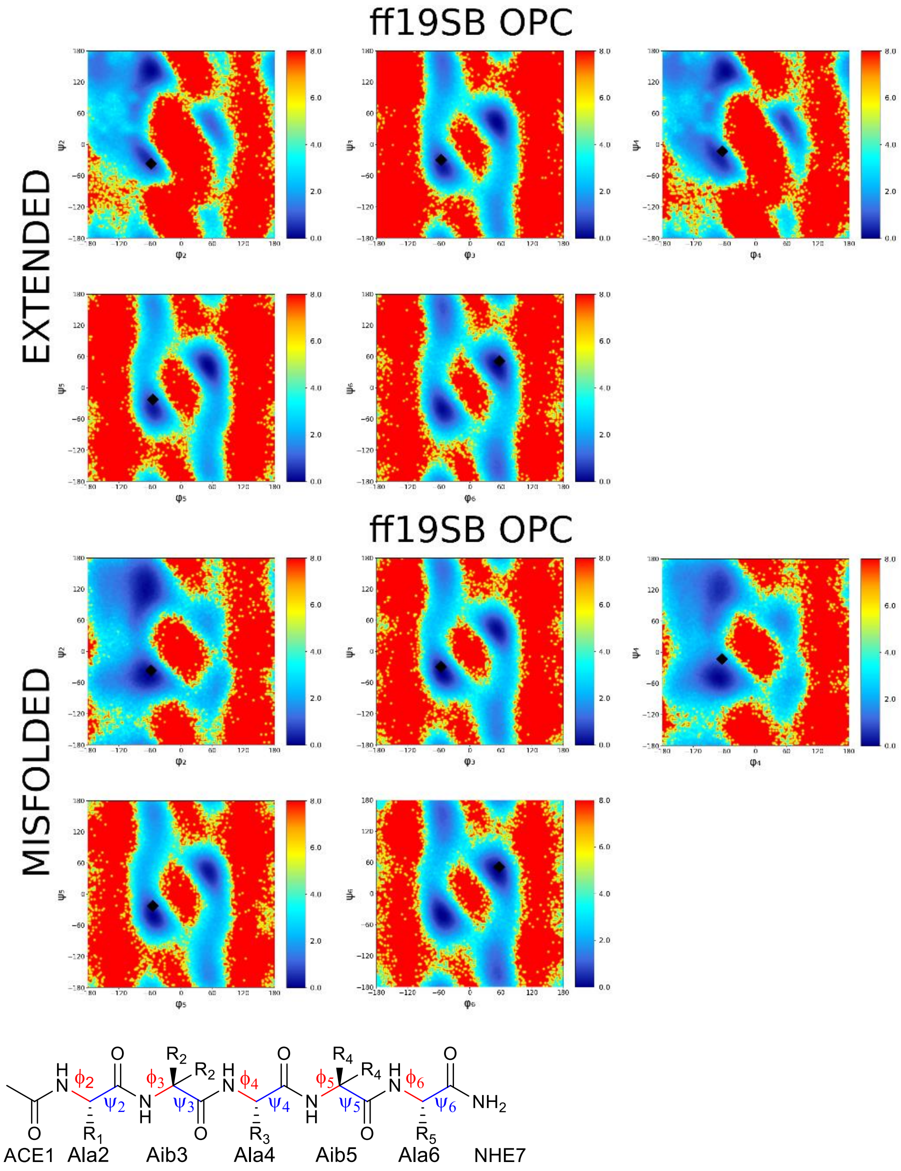

Figure S37. PMFs obtained from φ and ψ dihedral distributions of H2 residues for the ff19SB/OPC combination from extended (top panels) and misfolded (bottom panels) conformations. PMFs are shown as heat maps (0.0 – 8.0 kcal/mol range) from dark blue to red. The native conformation's φ and ψ angles are reported as black diamonds. H2 sequence and representation of φ and ψ angles numbering are also shown.

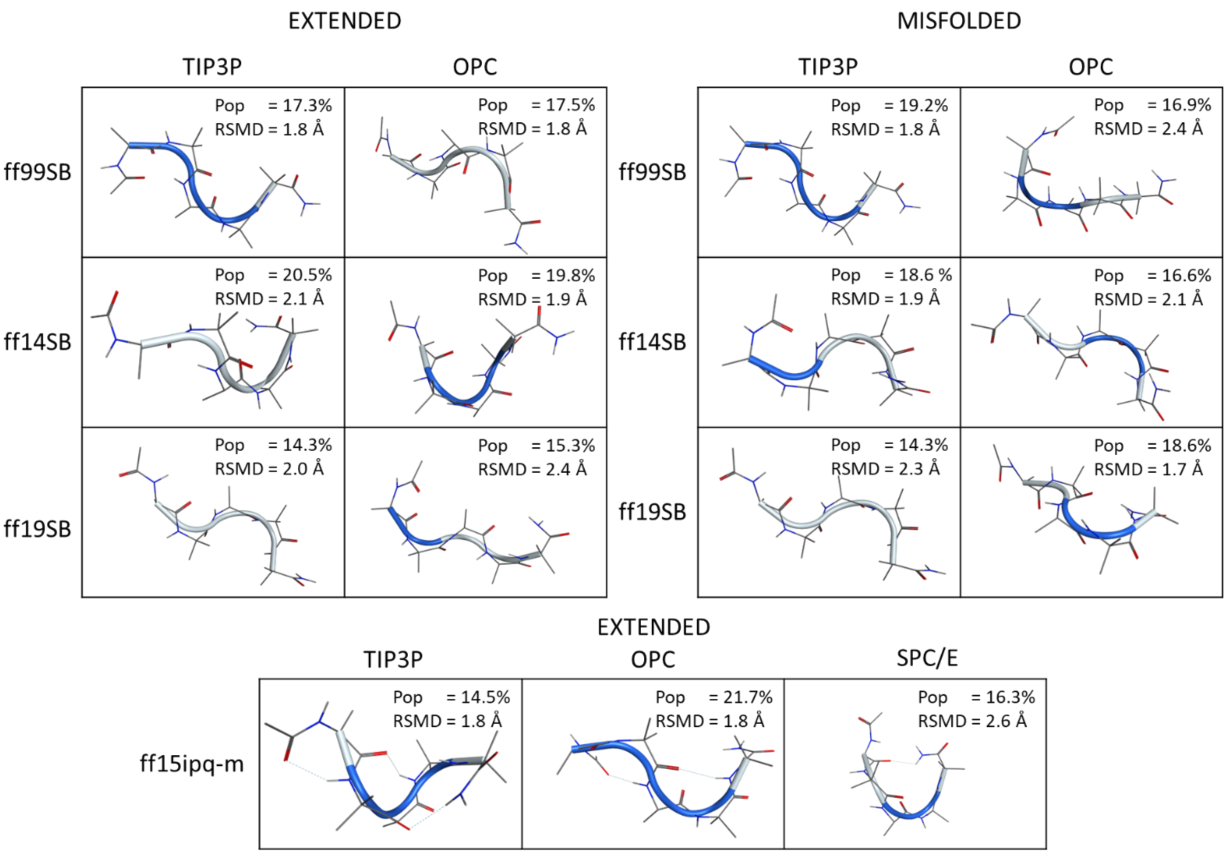

Figure S38. Representative conformation of the second clusters from H2 trajectories. Population (pop%) and RMSD vs native structure are also shown in the figure.

Table S4. H-bond analyses from H2 trajectories. Only H-bonds involving the backbone are included in the table (donor N-H, acceptor C=O). Native H-bonds are highlighted in bold.

NATIVE		
acceptor	donor	Distance
**AIB3**	**AIB6**	2.91
**AIB3**	**AIB5**	2.94
**ALA2**	**AIB5**	2.97
**ALA2**	**ALA4**	3.02
**ALA4**	**AIB6**	3.04
**ACE1**	**ALA4**	3.13
**ACE1**	**AIB3**	3.22
**AIB5**	**NHE7**	3.34
**ACE1**	**AIB5**	4.43

ff99SB OPC					
EXTENDED			MISFOLDED		
acceptor	donor	occ%	acceptor	donor	occ%
ALA2	AIB6	5.34	**ACE1**	**ALA4**	5.22

ff99SB TIP3P					
EXTENDED			MISFOLDED		
acceptor	donor	occ%	acceptor	donor	occ%
**ACE1**	**ALA4**	8.63	**ACE1**	**ALA4**	7.75
ALA2	AIB6	8.01	ALA2	AIB6	6.82

**ACE1**	**AIB5**	5.30	**ALA4**	**AIB6**	5.02

ff14SB OPC					
EXTENDED			MISFOLDED		
acceptor	donor	occ%	acceptor	donor	occ%
**ACE1**	**ALA4**	5.90	ALA2	AIB6	6.90
ALA2	AIB6	5.86	**ACE1**	**ALA4**	5.82

ff14SB TIP3P					
EXTENDED			MISFOLDED		
acceptor	donor	occ%	acceptor	donor	occ%
ALA2	AIB6	8.68	ALA2	AIB6	9.88
**ACE1**	**ALA4**	8.58	**ACE1**	**ALA4**	8.25
**ACE1**	**AIB5**	6.74	**ACE1**	**AIB5**	6.63

ff19SB OPC					
EXTENDED			MISFOLDED		
acceptor	donor	occ%	acceptor	donor	occ%
			ALA2	AIB6	6.06
			**ACE1**	**ALA4**	5.30

ff19SB TIP3P					
EXTENDED			MISFOLDED		
acceptor	donor	occ%	acceptor	donor	occ%
ALA2	AIB6	11.35	ALA2	AIB6	10.86
**ACE1**	**ALA4**	8.56	**ACE1**	**ALA4**	8.16
**ACE1**	**AIB5**	7.12	**ACE1**	**AIB5**	6.66
			**ALA4**	**AIB6**	5.54

ff15ipq-m OPC		
acceptor	donor	occ%
ALA4	NHE7	12.43
**ACE1**	**ALA4**	8.05
**AIB3**	**AIB5**	7.62
**ACE1**	**AIB3**	7.38

ff15ipq-m TIP3P		
acceptor	donor	occ%
ALA4	NHE7	10.13
**ACE1**	**ALA4**	6.00
**ACE1**	**AIB3**	5.11

ff15ipq-m SPC/E		
acceptor	donor	occ%
ALA4	NHE7	8.50
**ACE1**	**ALA4**	6.30

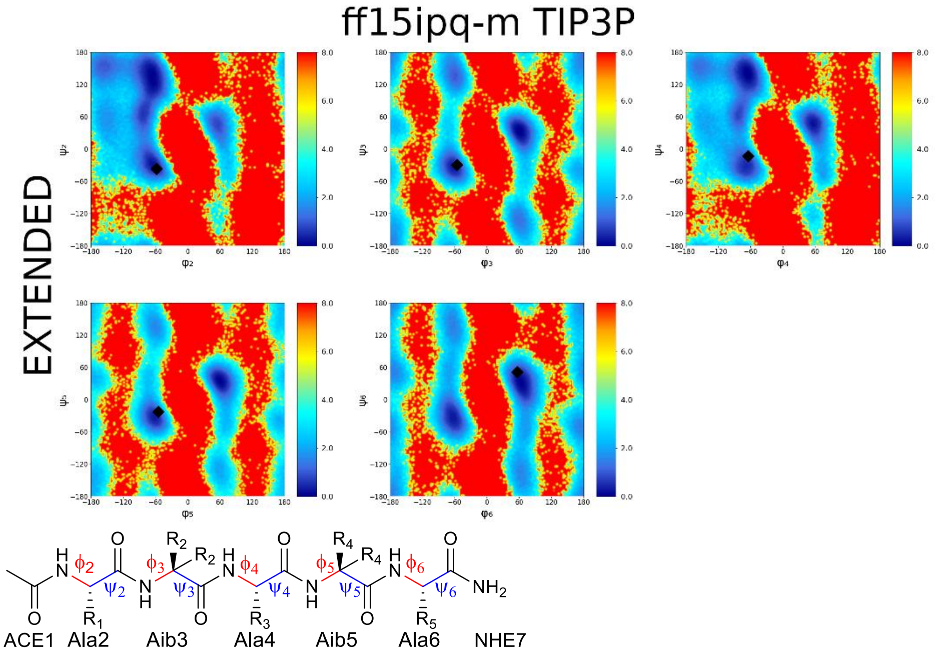

Figure S39. PMFs obtained from φ and ψ dihedral distributions of H2 residues for the ff115ipq-m/TIP3P combination from an extended conformation. PMFs are shown as heat maps (0.0 – 8.0 kcal/mol range) from dark blue to red. The native conformation's φ and ψ angles are reported as black diamonds. H2 sequence and representation of φ and ψ angles numbering are also shown.

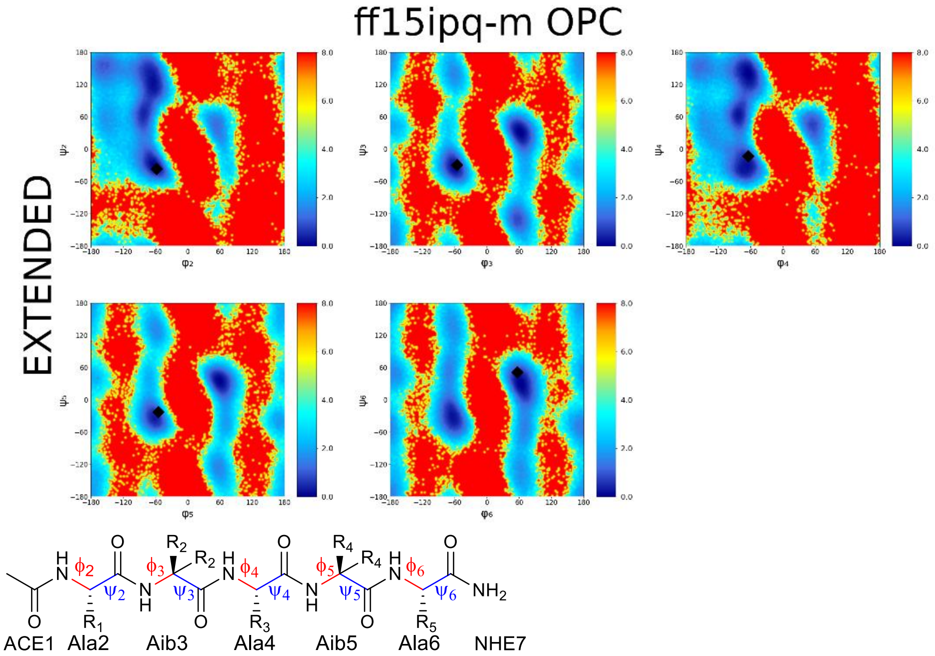

Figure S40. PMFs obtained from φ and ψ dihedral distributions of H2 residues for the ff115ipq-m/OPC combination from an extended conformation. PMFs are shown as heat maps (0.0 – 8.0 kcal/mol range) from dark blue to red. The native conformation's φ and ψ angles are reported as black diamonds. H2 sequence and representation of φ and ψ angles numbering are also shown.

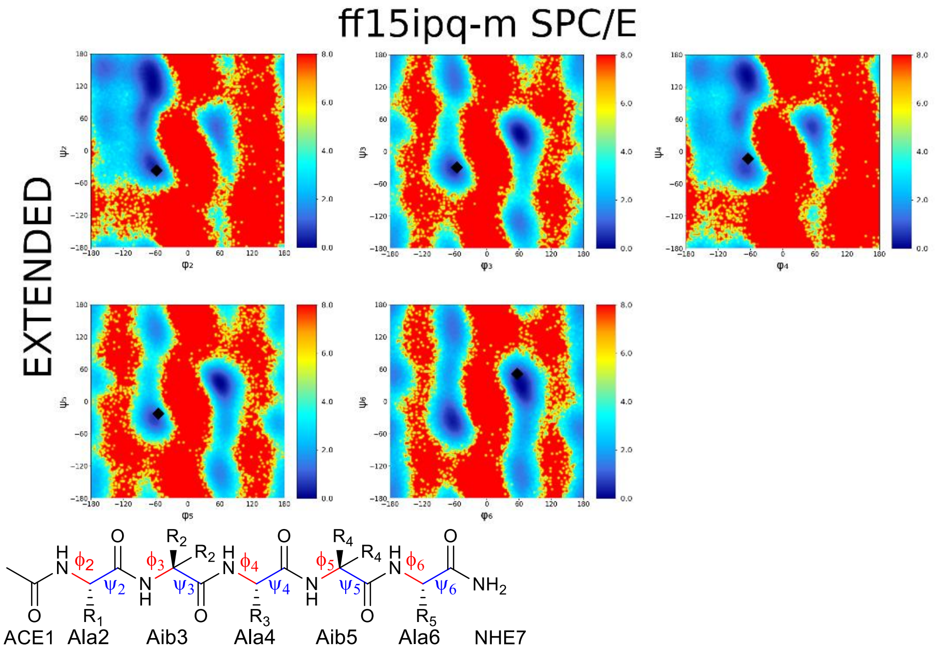

Figure S41. PMFs obtained from φ and ψ dihedral distributions of H2 residues for the ff115ipq-m/SPC/E combination from an extended conformation. PMFs are shown as heat maps (0.0 – 8.0 kcal/mol range) from dark blue to red. The native conformation's φ and ψ angles are reported as black diamonds. H2 sequence and representation of φ and ψ angles numbering are also shown.

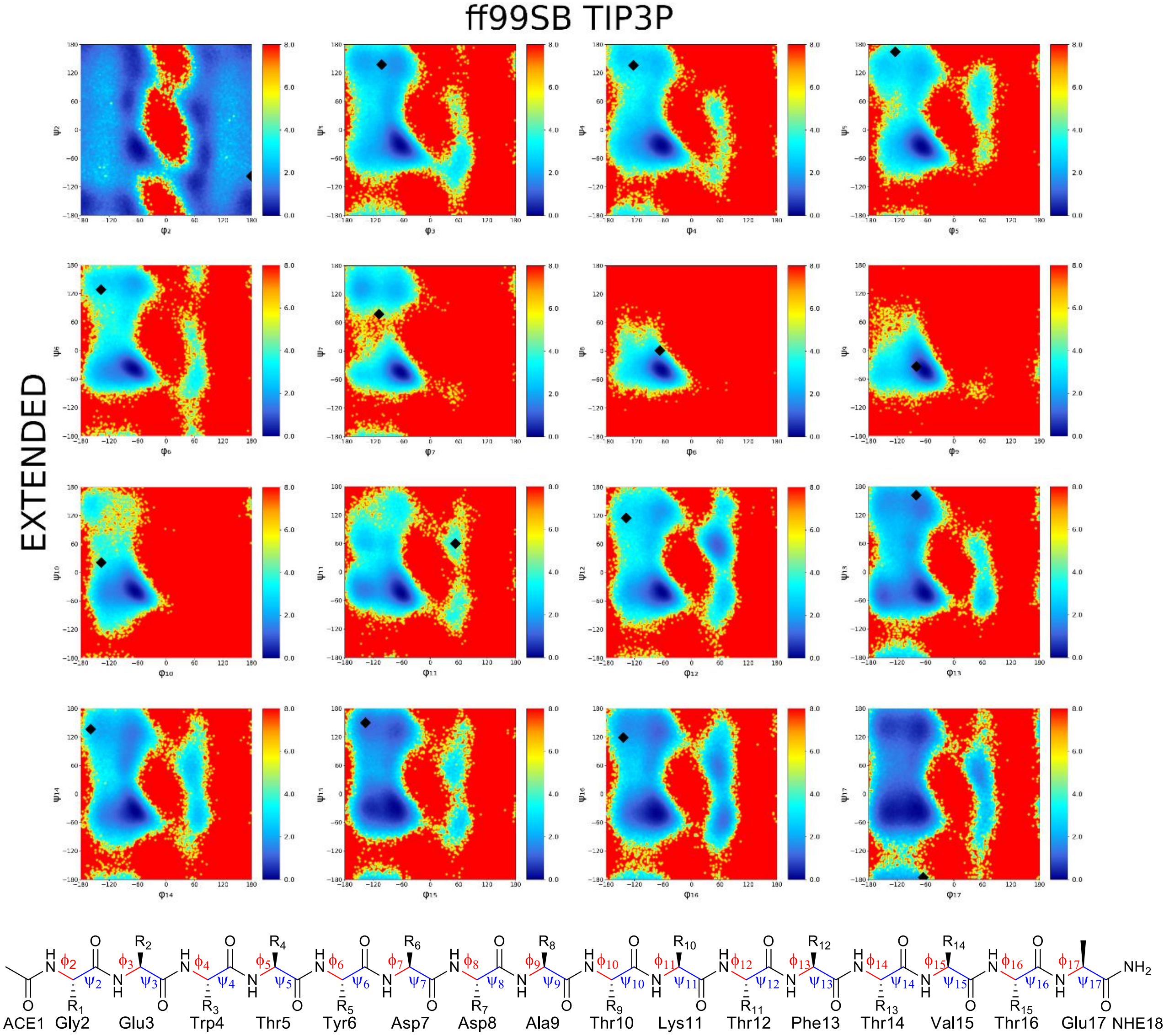

Figure S42. PMFs obtained from φ and ψ dihedral distributions of B1 residues for the ff99SB/TIP3P combination from an extended conformation. PMFs are shown as heat maps (0.0 – 8.0 kcal/mol range) from dark blue to red. The native conformation's φ and ψ angles are reported as black diamonds. B1 sequence and representation of φ and ψ angles numbering are also shown.

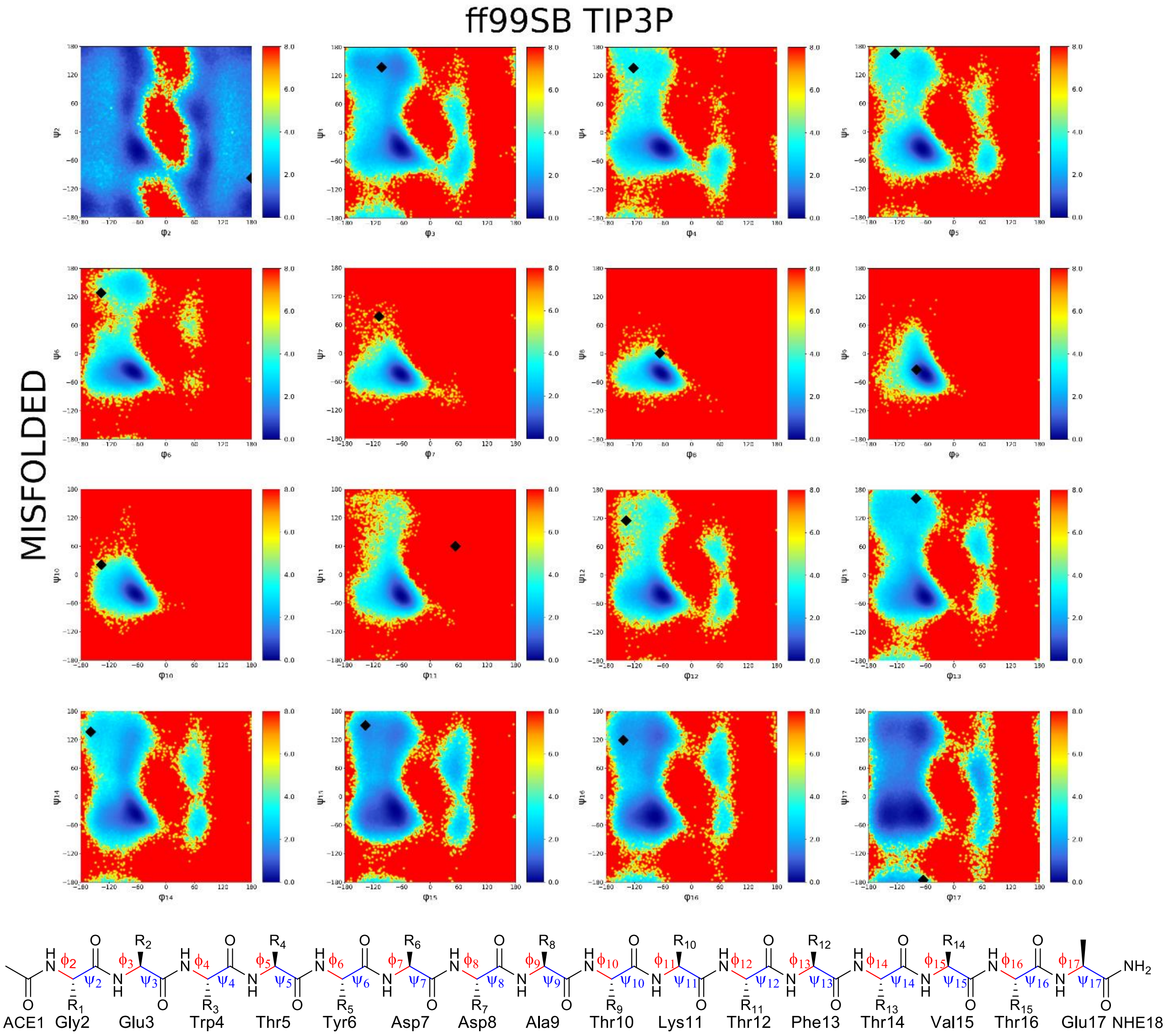

Figure S43. PMFs obtained from φ and ψ dihedral distributions of B1 residues for the ff99SB/TIP3P combination from a misfolded conformation. PMFs are shown as heat maps (0.0 – 8.0 kcal/mol range) from dark blue to red. The native conformation's φ and ψ angles are reported as black diamonds. B1 sequence and representation of φ and ψ angles numbering are also shown.

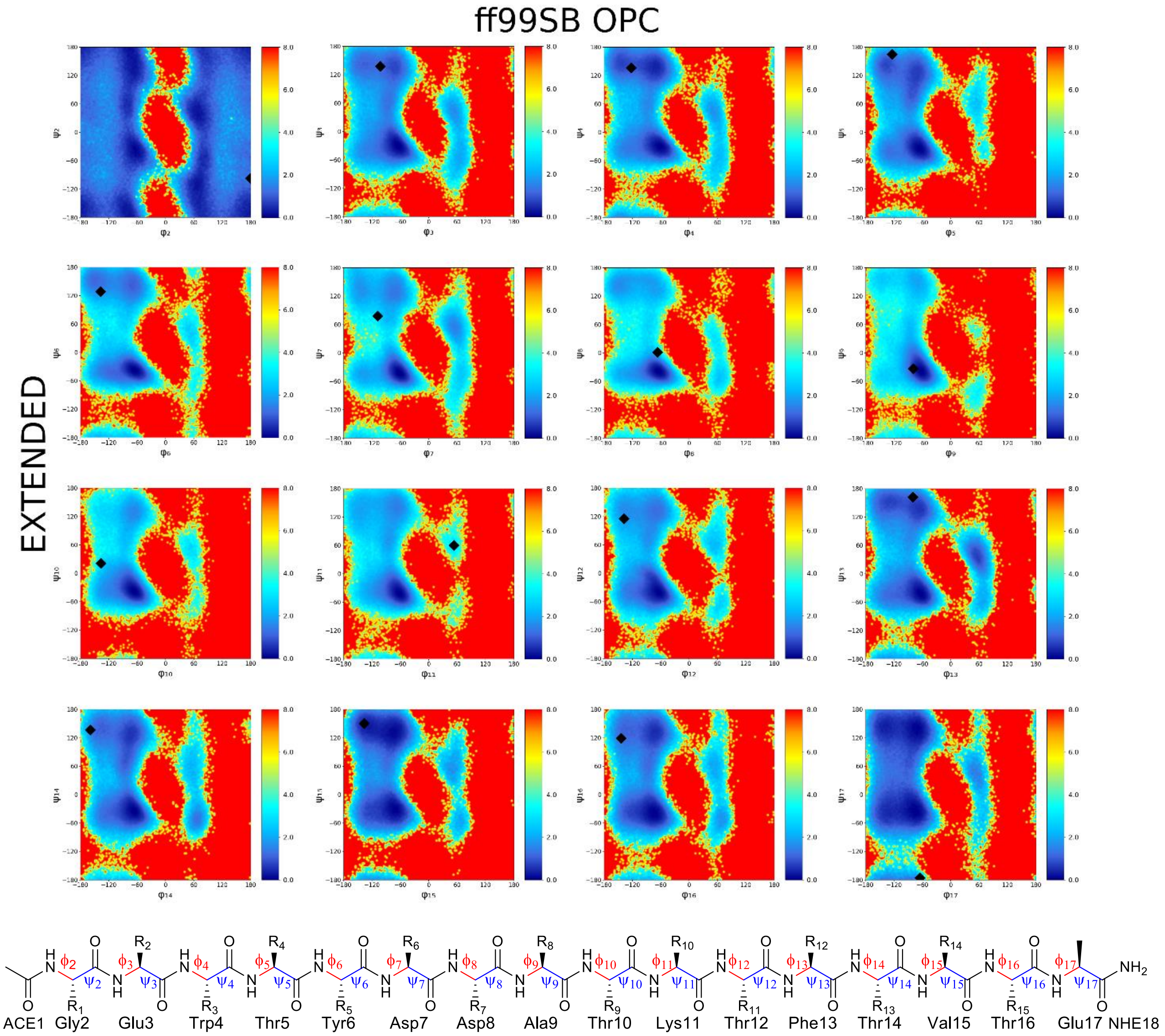

Figure S44. PMFs obtained from φ and ψ dihedral distributions of B1 residues for the ff99SB/OPC combination from an extended conformation. PMFs are shown as heat maps (0.0 – 8.0 kcal/mol range) from dark blue to red. The native conformation's φ and ψ angles are reported as black diamonds. B1 sequence and representation of φ and ψ angles numbering are also shown.

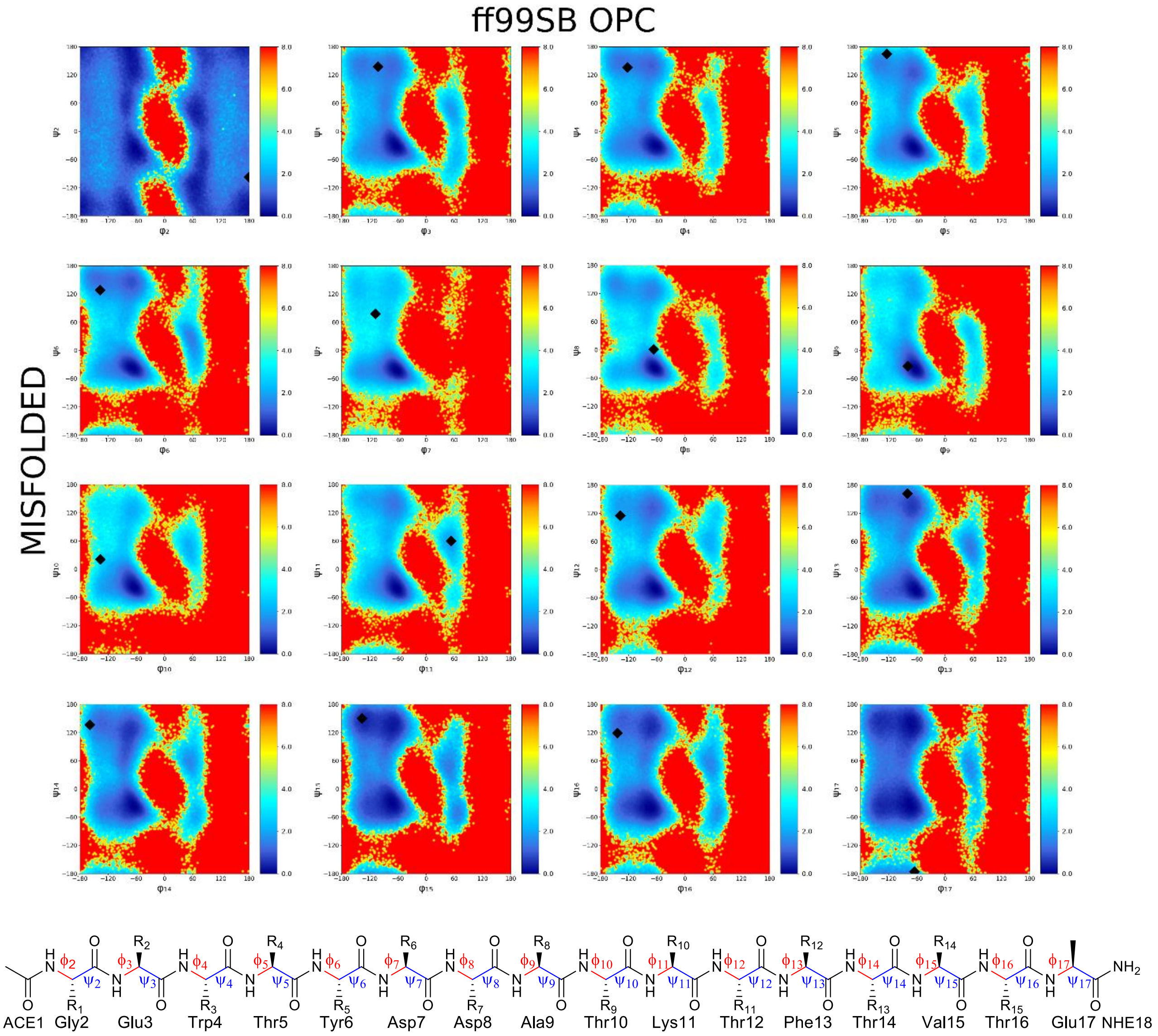

Figure S45. PMFs obtained from φ and ψ dihedral distributions of B1 residues for the ff99SB/OPC combination from a misfolded conformation. PMFs are shown as heat maps (0.0 – 8.0 kcal/mol range) from dark blue to red. The native conformation's φ and ψ angles are reported as black diamonds. B1 sequence and representation of φ and ψ angles numbering are also shown.

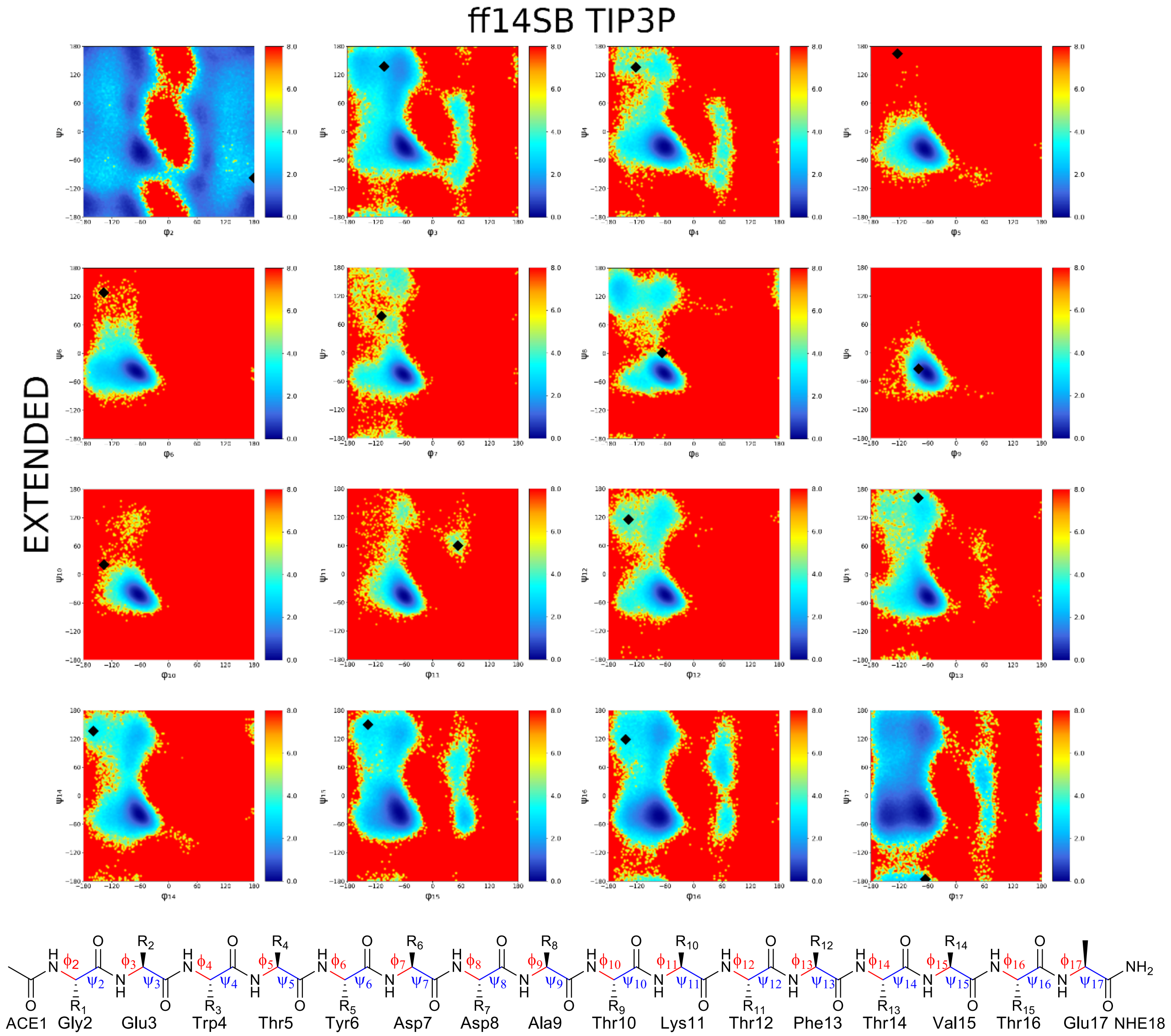

Figure S46. PMFs obtained from φ and ψ dihedral distributions of B1 residues for the ff14SB/TIP3P combination from an extended conformation. PMFs are shown as heat maps (0.0 – 8.0 kcal/mol range) from dark blue to red. The native conformation's φ and ψ angles are reported as black diamonds. B1 sequence and representation of φ and ψ angles numbering are also shown.

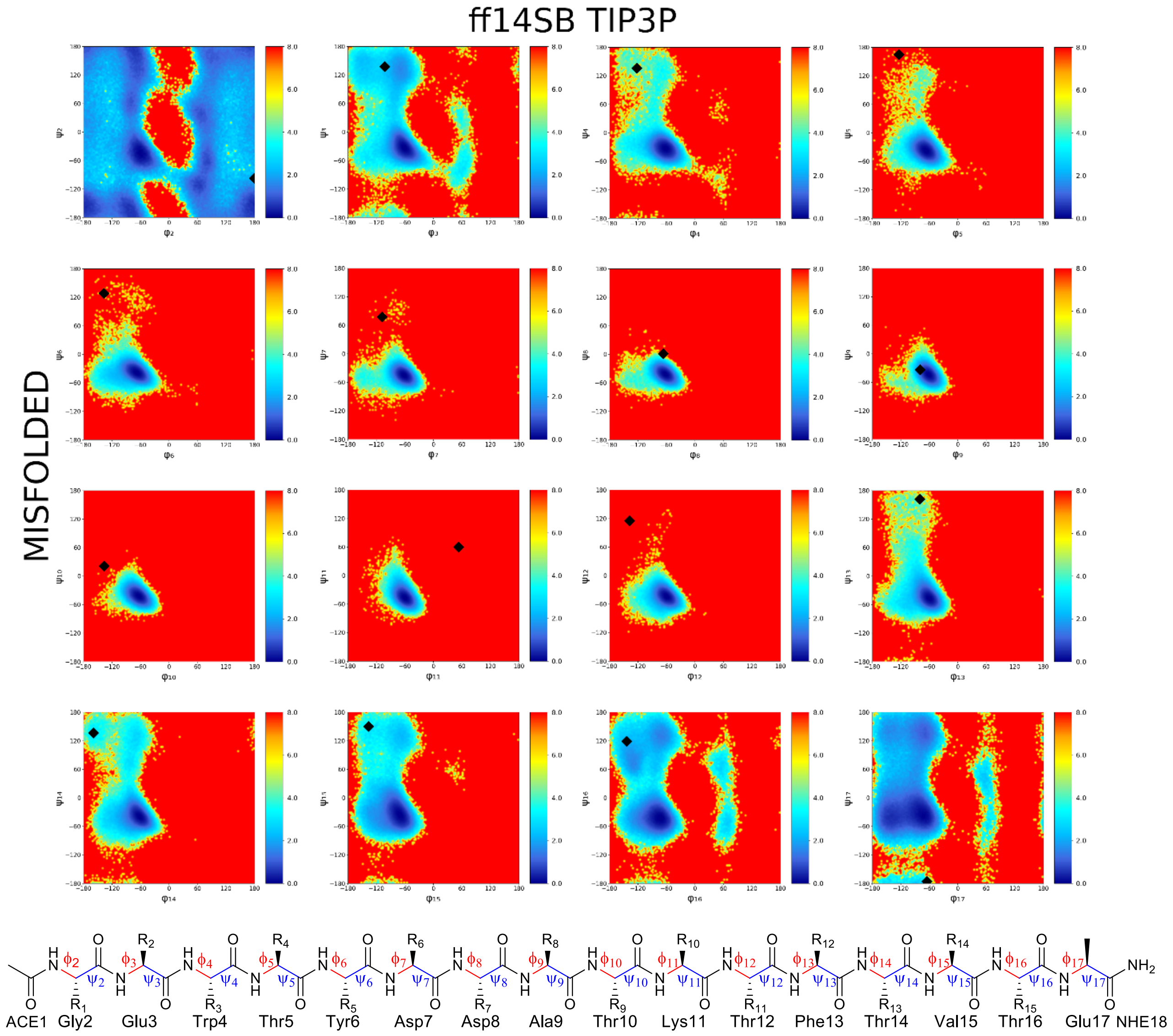

Figure S47. PMFs obtained from φ and ψ dihedral distributions of B1 residues for the ff14SB/TIP3P combination from a misfolded conformation. PMFs are shown as heat maps (0.0 – 8.0 kcal/mol range) from dark blue to red. The native conformation's φ and ψ angles are reported as black diamonds. B1 sequence and representation of φ and ψ angles numbering are also shown.

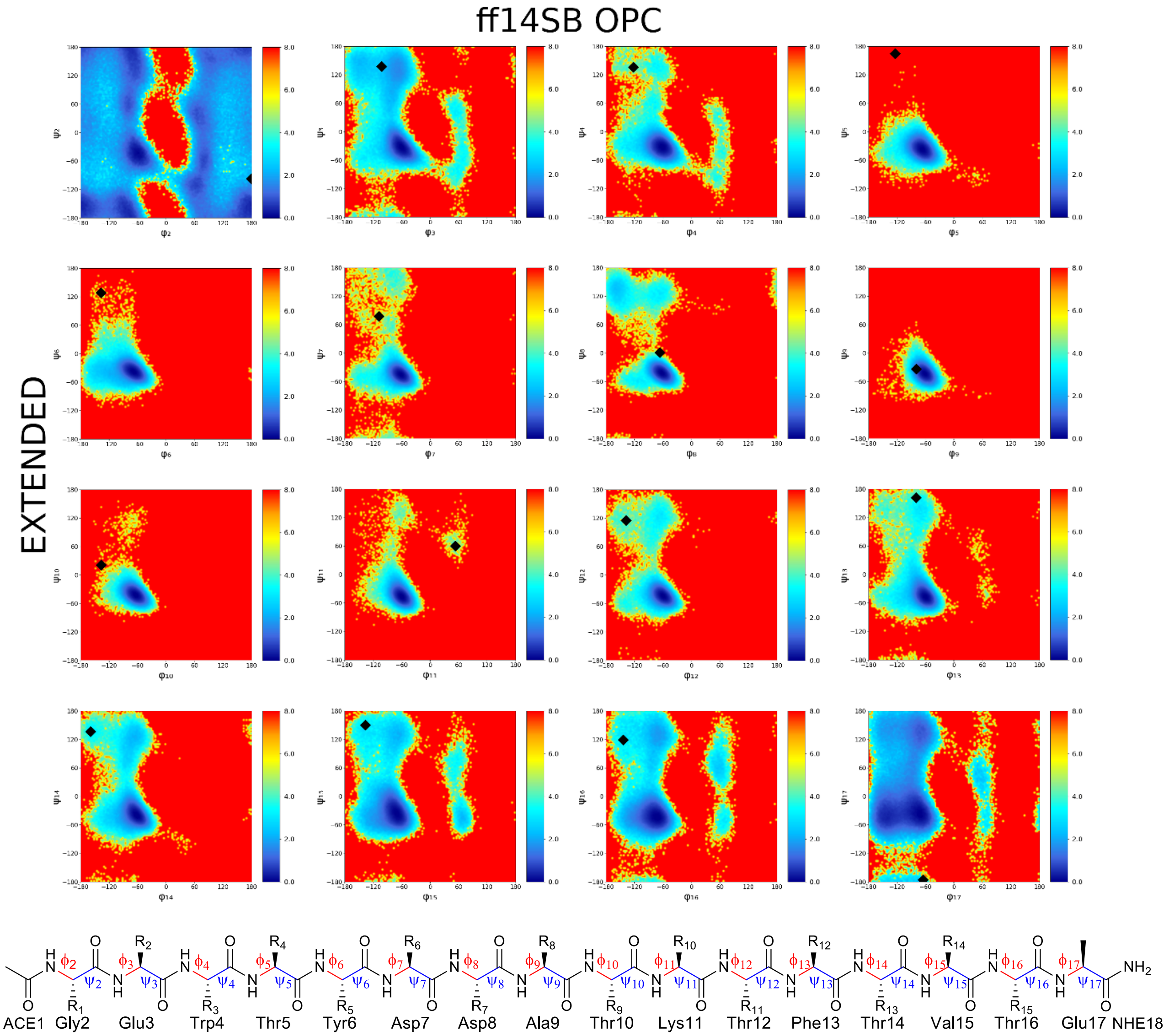

Figure S48. PMFs obtained from φ and ψ dihedral distributions of B1 residues for the ff14SB/OPC combination from an extended conformation. PMFs are shown as heat maps (0.0 – 8.0 kcal/mol range) from dark blue to red. The native conformation's φ and ψ angles are reported as black diamonds. B1 sequence and representation of φ and ψ angles numbering are also shown.

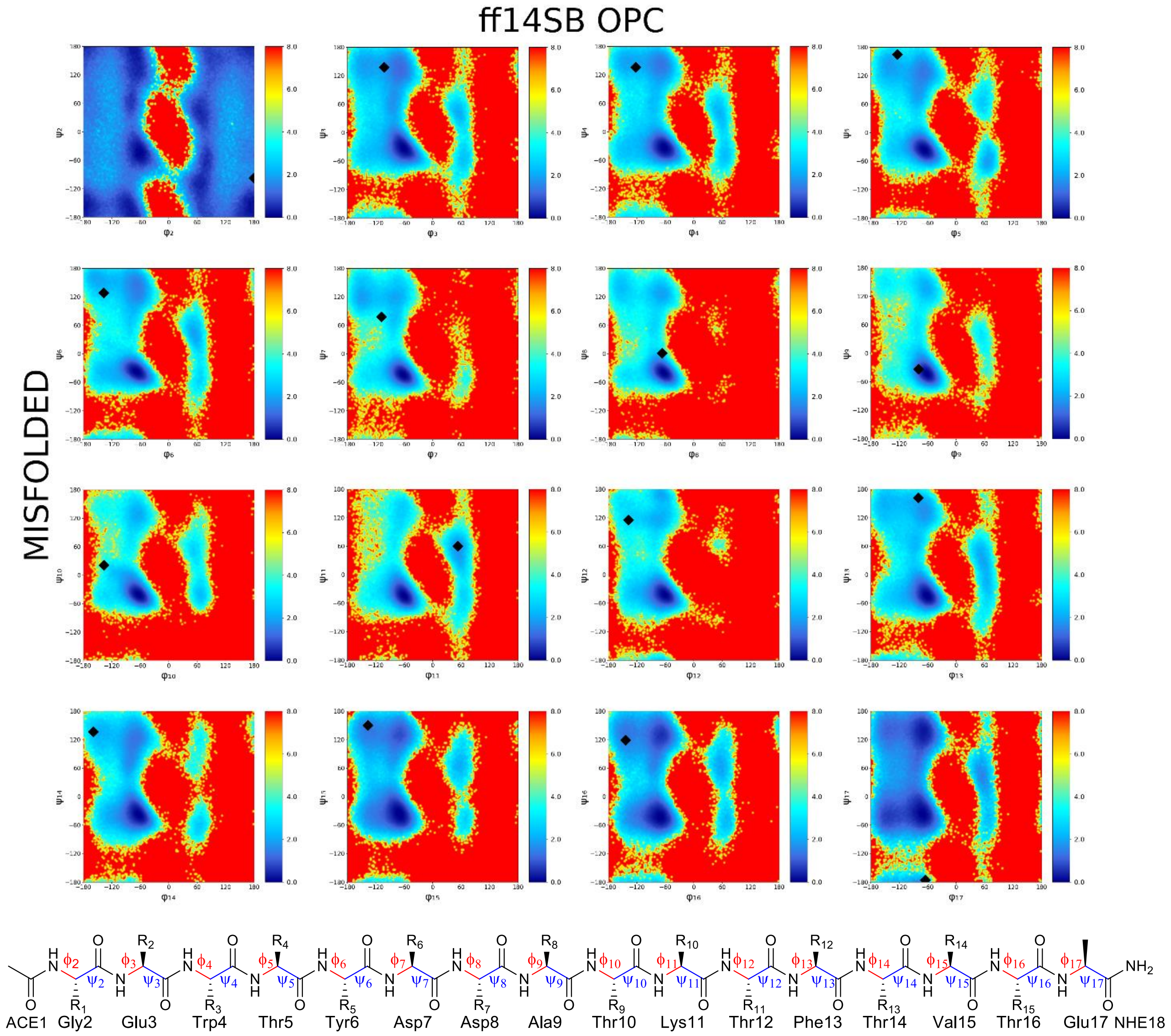

Figure S49. PMFs obtained from φ and ψ dihedral distributions of B1 residues for the ff14SB/OPC combination from a misfolded conformation. PMFs are shown as heat maps (0.0 – 8.0 kcal/mol range) from dark blue to red. The native conformation's φ and ψ angles are reported as black diamonds. B1 sequence and representation of φ and ψ angles numbering are also shown.

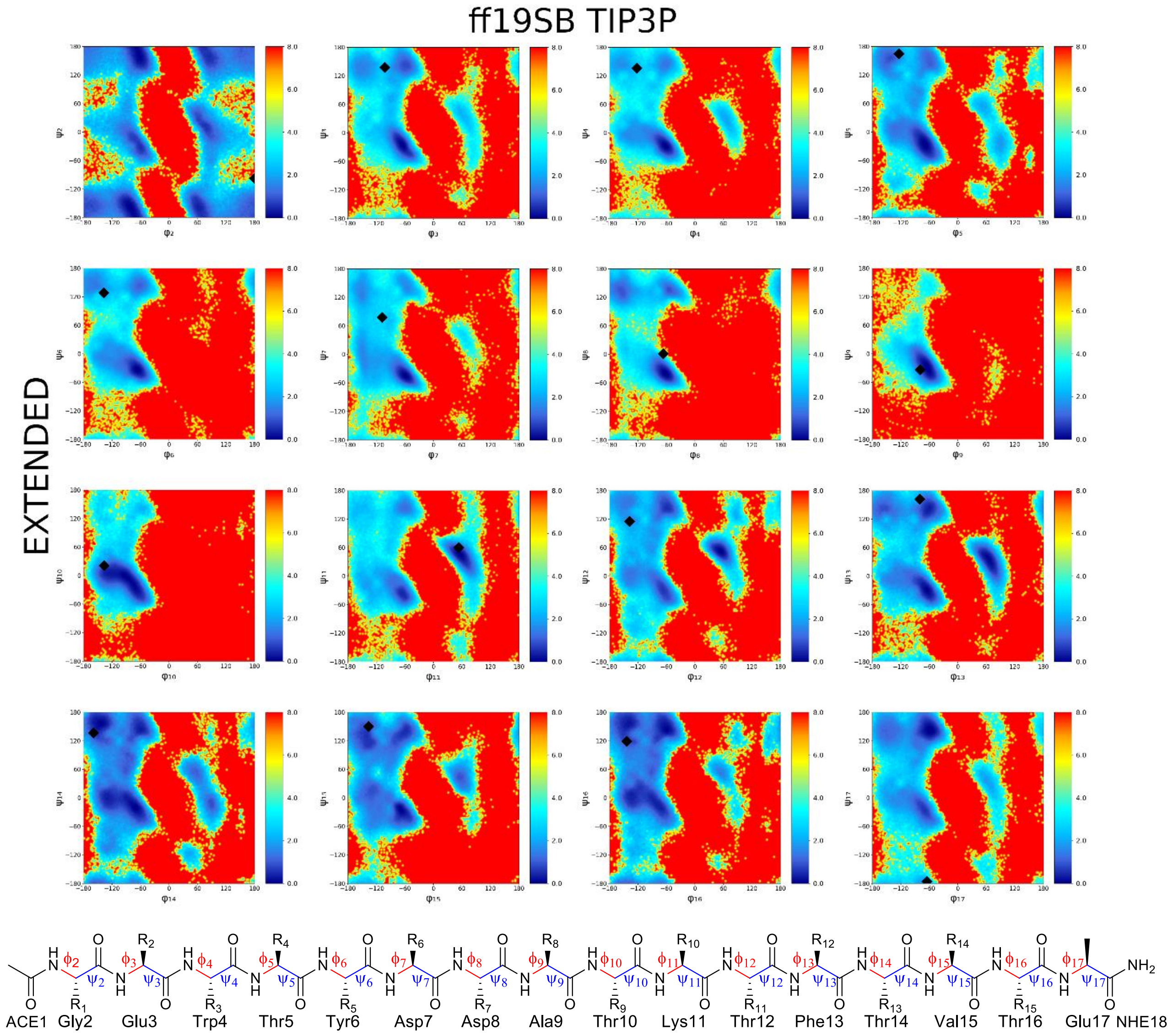

Figure S50. PMFs obtained from φ and ψ dihedral distributions of B1 residues for the ff19SB/TIP3P combination from an extended conformation. PMFs are shown as heat maps (0.0 – 8.0 kcal/mol range) from dark blue to red. The native conformation's φ and ψ angles are reported as black diamonds. B1 sequence and representation of φ and ψ angles numbering are also shown.

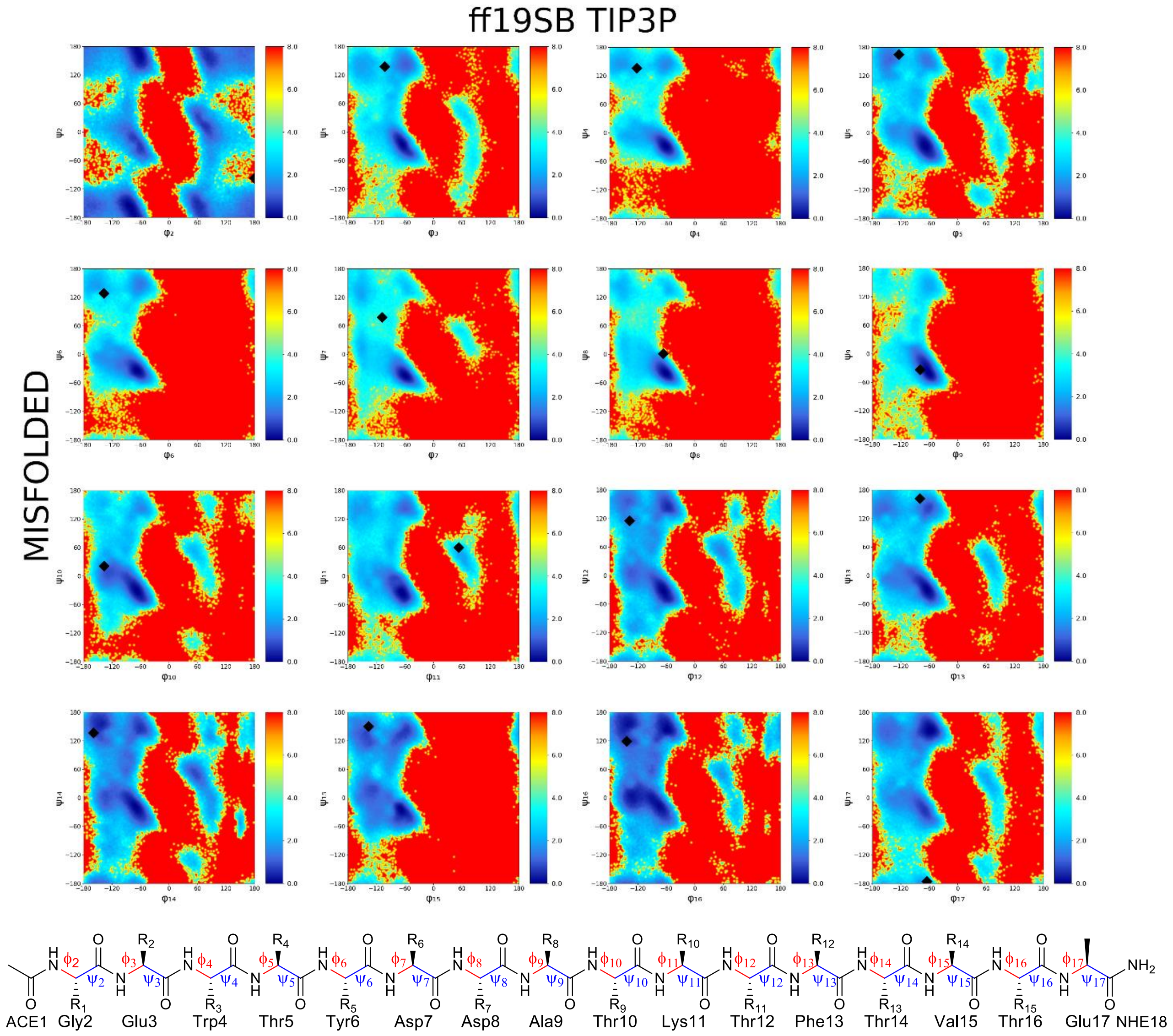

Figure S51. PMFs obtained from φ and ψ dihedral distributions of B1 residues for the ff19SB/TIP3P combination from a misfolded conformation. PMFs are shown as heat maps (0.0 – 8.0 kcal/mol range) from dark blue to red. The native conformation's φ and ψ angles are reported as black diamonds. B1 sequence and representation of φ and ψ angles numbering are also shown.

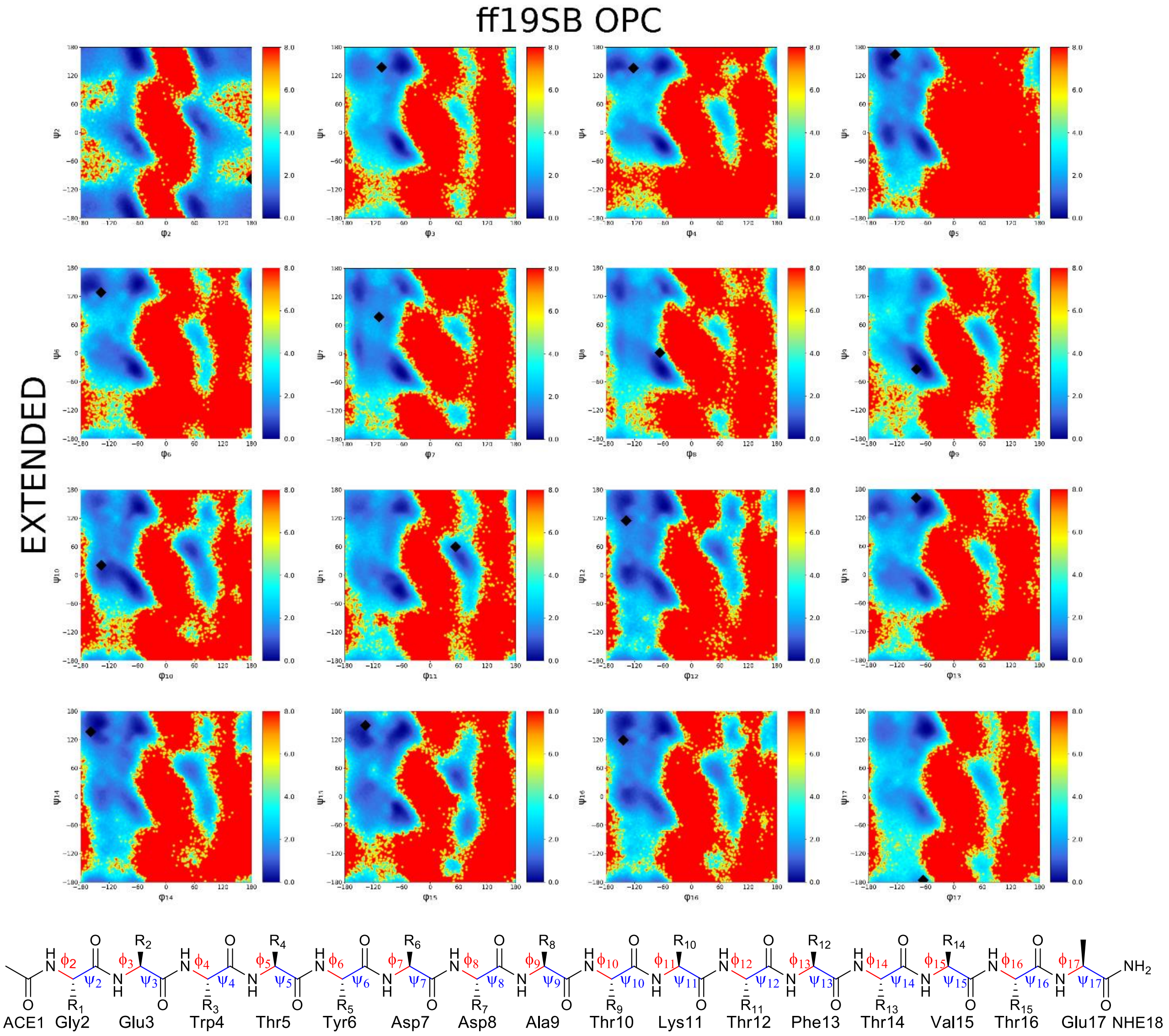

Figure S52. PMFs obtained from φ and ψ dihedral distributions of B1 residues for the ff19SB/OPC combination from an extended conformation. PMFs are shown as heat maps (0.0 – 8.0 kcal/mol range) from dark blue to red. The native conformation's φ and ψ angles are reported as black diamonds. B1 sequence and representation of φ and ψ angles numbering are also shown.

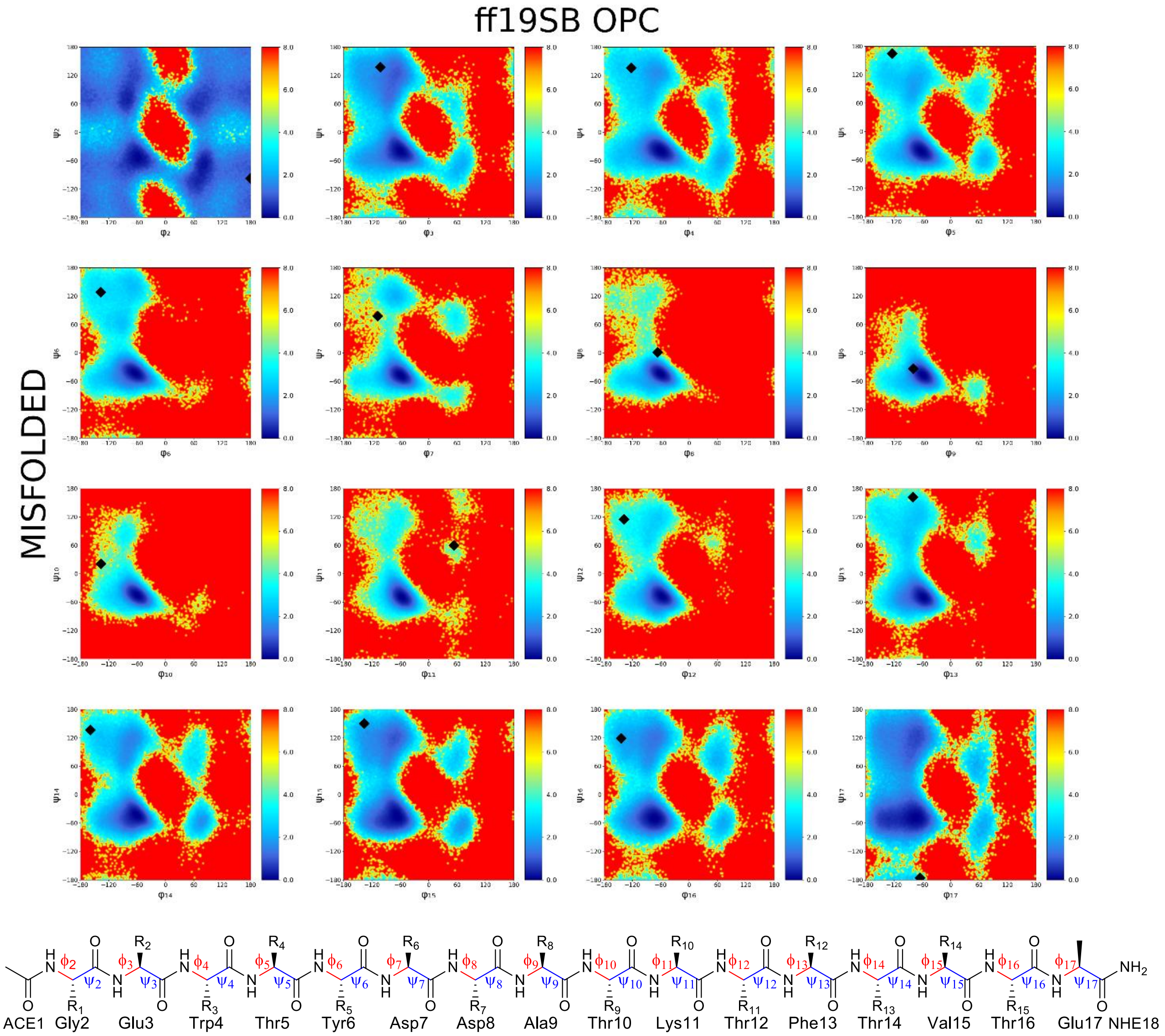

Figure S53. PMFs obtained from φ and ψ dihedral distributions of B1 residues for the ff19SB/OPC combination from a misfolded conformation. PMFs are shown as heat maps (0.0 – 8.0 kcal/mol range) from dark blue to red. The native conformation's φ and ψ angles are reported as black diamonds. B1 sequence and representation of φ and ψ angles numbering are also shown.

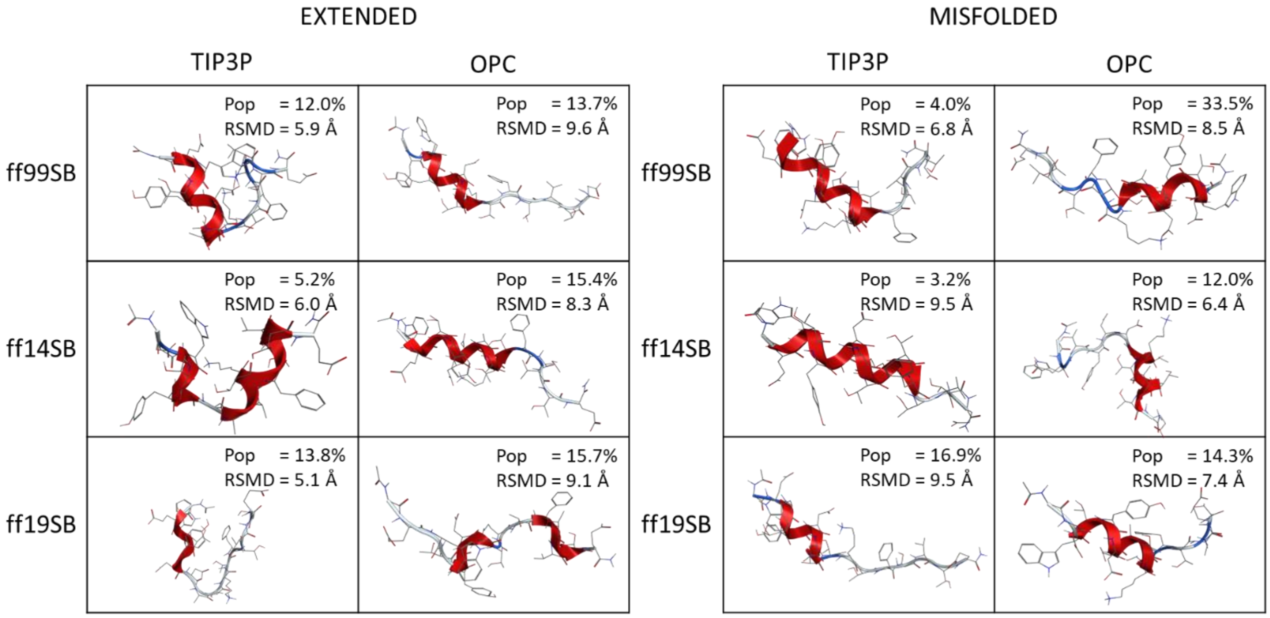

Figure S54. Representative conformation and population (pop%) of the second clusters from B1 trajectories. RMSD vs native structure are also shown.

Table S5. H-bond analyses from B1 trajectories. Only H-bonds involving the backbone are included in the table (donor N-H, acceptor C=O). Native H-bonds are highlighted in bold.

NATIVE		
acceptor	donor	Distance
**ASP7**	**ALA9**	**2.78**
**ASP7**	**LYS11**	**2.98**
**GLU3**	**THR16**	**3.06**
**THR12**	**ASP7**	**3.08**
**ASP7**	**THR10**	**3.18**
**ASP7**	**THR12**	**3.22**
**THR5**	**THR14**	**3.33**
**THR16**	**GLU3**	**3.37**
**THR10**	**THR12**	**3.38**
**TYR6**	**ASP8**	**3.59**
**ASP8**	**THR10**	**3.68**
**THR12**	**THR14**	**3.95**
**GLY2**	**TRP4**	**4.04**
**ASP8**	**LYS11**	**4.29**
**ALA9**	**LYS11**	**4.32**
**GLU3**	**THR5**	**4.35**
**LYS11**	**PHE13**	**4.46**

ff99SB OPC					
EXTENDED			MISFOLDED		
acceptor	donor	occ%	acceptor	donor	occ%
ASP8	THR12	23.70	ALA9	PHE13	24.34

ff99SB TIP3P					
EXTENDED			MISFOLDED		
acceptor	donor	occ%	acceptor	donor	occ%
**ASP7**	**LYS11**	43.99	**ASP7**	**LYS11**	48.48

TYR6	THR10	21.02
**ASP7**	**LYS11**	20.94
ALA9	PHE13	18.57
THR5	ALA9	13.73
THR10	THR14	10.03
GLU3	ASP7	9.80
TRP4	ASP8	8.05
PHE13	GLU17	6.38
LYS11	VAL15	6.28
ALA9	THR14	5.52
GLU3	TYR6	5.51
**ASP8**	**LYS11**	5.31
GLY2	THR5	5.04

TYR6	THR10	22.35
ASP8	THR12	21.02
**ASP7**	**LYS11**	19.94
TRP4	ASP8	13.16
THR5	ALA9	12.28
THR10	THR14	12.16
GLU3	ASP7	10.75
LYS11	VAL15	9.02
PHE13	GLU17	8.40
GLY2	THR5	7.70
GLY2	TYR6	5.49
ALA9	THR12	5.23

TYR6	THR10	34.59
ASP8	THR12	33.73
ALA9	PHE13	32.19
GLU3	ASP7	27.93
TRP4	ASP8	25.80
THR5	ALA9	24.74
THR10	THR14	18.67
PHE13	GLU17	17.70
LYS11	VAL15	15.99
GLY2	TYR6	14.25
GLY2	THR5	12.01
**ASP7**	**THR12**	8.92
GLU3	TYR6	8.41
THR12	THR16	6.61

ALA9	PHE13	46.09
ASP8	THR12	45.21
TYR6	THR10	45.01
GLU3	ASP7	31.71
TRP4	ASP8	31.07
THR5	ALA9	30.43
THR10	THR14	29.85
LYS11	VAL15	24.85
PHE13	GLU17	24.25
GLY2	THR5	12.05
GLY2	TYR6	11.81
GLU3	TYR6	10.96
THR12	THR16	10.90
THR12	GLU17	5.61
PHE13	THR16	5.08

ff14SB OPC					
EXTENDED			MISFOLDED		
acceptor	donor	occ%	acceptor	donor	occ%
**ASP7**	**LYS11**	50.91	ALA9	PHE13	36.34
ALA9	PHE13	48.16	**ASP7**	**LYS11**	35.76
TYR6	THR10	46.74	ASP8	THR12	33.62
ASP8	THR12	46.41	TYR6	THR10	32.01
THR5	ALA9	33.42	THR10	THR14	25.41
THR10	THR14	33.24	THR5	ALA9	21.26
TRP4	ASP8	28.98	LYS11	VAL15	20.82
GLU3	ASP7	26.93	PHE13	GLU17	18.83
LYS11	VAL15	24.34	TRP4	ASP8	18.30
PHE13	GLU17	23.66	GLU3	ASP7	17.30
GLY2	THR5	20.68	THR12	THR16	11.90
THR12	THR16	14.24	GLY2	THR5	10.97
GLY2	TYR6	11.73	GLY2	TYR6	9.29
GLU3	TYR6	7.91	GLU3	TYR6	5.30

ff14SB TIP3P					
EXTENDED			MISFOLDED		
acceptor	donor	occ%	acceptor	donor	occ%
**ASP7**	**LYS11**	54.92	**ASP7**	**LYS11**	58.56
ALA9	PHE13	54.72	ALA9	PHE13	57.31
ASP8	THR12	54.03	ASP8	THR12	55.66
TYR6	THR10	46.93	TYR6	THR10	51.25
PHE13	GLU17	43.61	THR10	THR14	41.59
THR10	THR14	41.88	PHE13	GLU17	39.88
LYS11	VAL15	36.09	THR5	ALA9	38.29
THR5	ALA9	34.81	LYS11	VAL15	37.43
TRP4	ASP8	34.22	TRP4	ASP8	35.49
GLU3	ASP7	32.97	GLU3	ASP7	34.04
THR12	THR16	22.82	THR12	THR16	22.97
GLY2	THR5	17.65	GLY2	TYR6	16.03
GLY2	TYR6	14.09	GLY2	THR5	16.00
GLU3	TYR6	9.99	GLU3	TYR6	8.67
PHE13	THR16	5.75	ACE1	THR5	5.71
			PHE13	THR16	5.66

ff19SB OPC					
EXTENDED			MISFOLDED		
acceptor	donor	occ%	acceptor	donor	occ%
GLY2	THR5	6.51	ALA9	PHE13	47.92
**ASP7**	**THR10**	5.01	**ASP7**	**LYS11**	46.64
			ASP8	THR12	45.65
			TYR6	THR10	41.59
			THR10	THR14	35.62
			LYS11	VAL15	28.43
			THR5	ALA9	27.90
			TRP4	ASP8	26.64
			GLU3	ASP7	23.23

ff19SB TIP3P					
EXTENDED			MISFOLDED		
acceptor	donor	occ%	acceptor	donor	occ%
GLY2	THR5	15.31	**ASP7**	**LYS11**	25.16
TYR6	THR10	15.07	TYR6	THR10	24.81
THR5	ALA9	14.54	GLY2	THR5	19.04
**ASP7**	**LYS11**	14.16	THR5	ALA9	18.78
**ASP7**	**THR12**	12.26	ASP8	THR12	18.51
ASP8	LYS11	12.23	GLU3	ASP7	16.70
GLU3	ASP7	11.55	ALA9	PHE13	15.98
**ASP8**	**THR12**	11.12	TRP4	ASP8	15.57
THR10	THR14	10.58	GLU3	TYR6	10.86

PHE13	GLU17	21.77
THR12	THR16	13.94
GLY2	TYR6	11.76
THR12	GLU17	10.28
GLU3	TYR6	7.11
GLY2	THR5	6.56
THR10	VAL15	5.76
TYR6	LYS11	5.57

TRP4	ASP8	9.63
GLU3	TYR6	7.71
ALA9	PHE13	5.96

**ASP7**	**THR10**	7.58
THR10	THR14	6.48

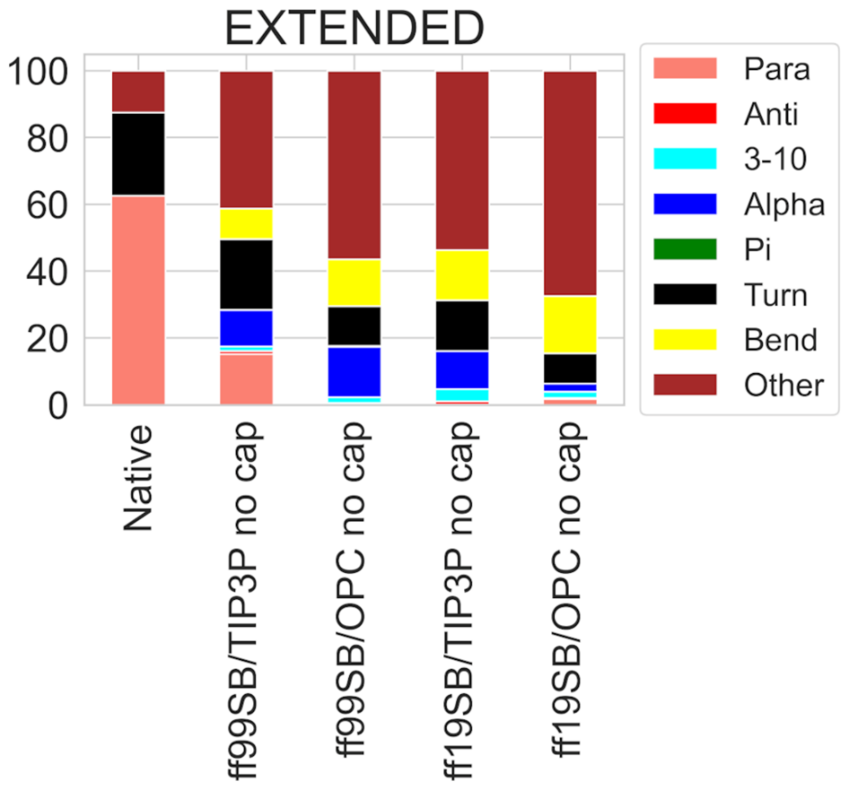

Figure S55. DSSP analysis of B1 uncapped trajectories from extended conformation. Data are reported as percentage of the distributions of all the residues considering the last 500 ns frames.

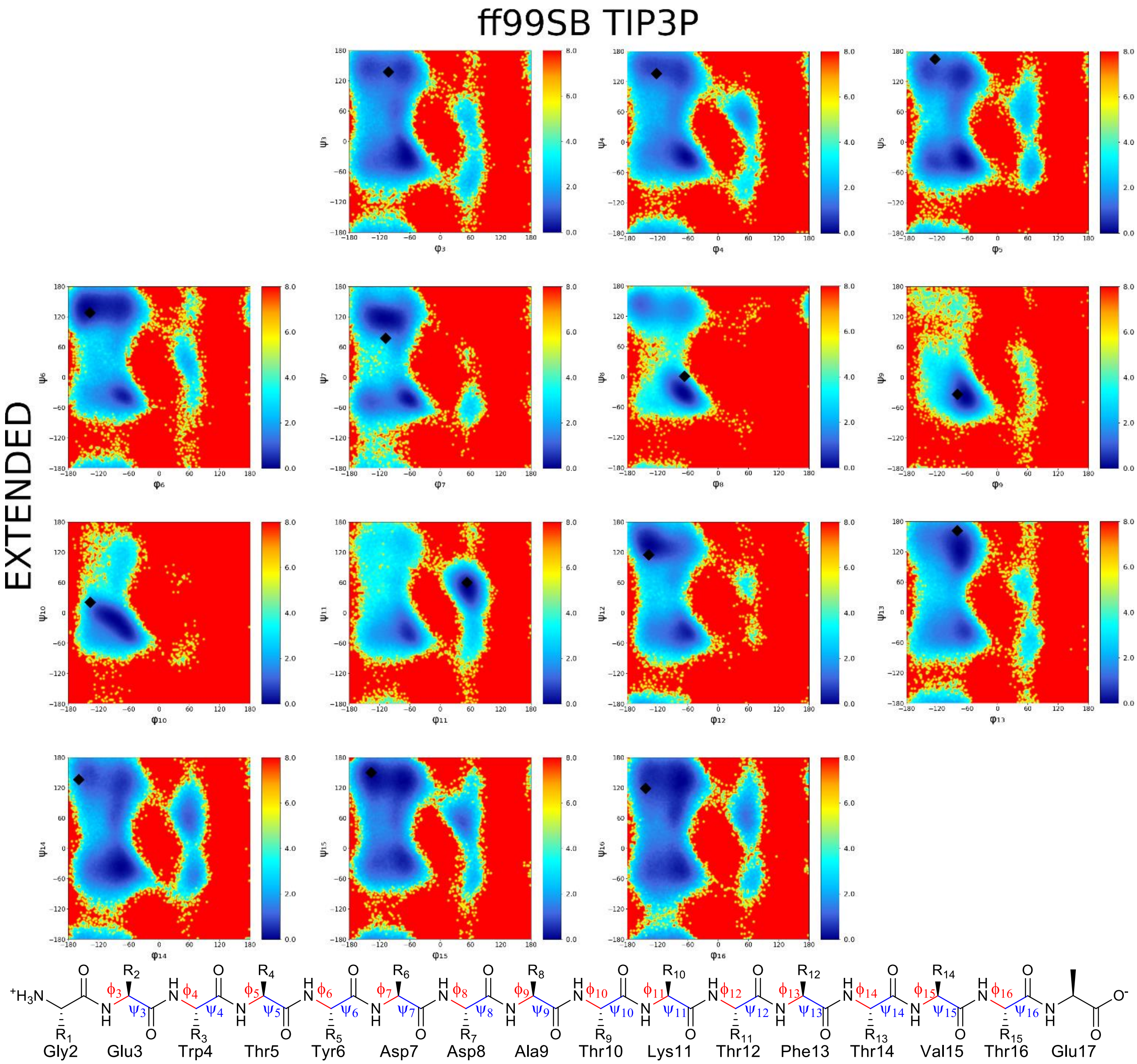

Figure S56. PMFs obtained from φ and ψ dihedral distributions of B1 uncapped residues for the ff99SB/TIP3P combination from extended (top panel) and misfolded (bottom panels) conformations. PMFs are shown as heat maps (0.0 – 8.0 kcal/mol range) from dark blue to red. The native conformation's φ and ψ angles are reported as black diamonds. B1 uncapped sequence and representation of φ and ψ angles numbering are also shown.

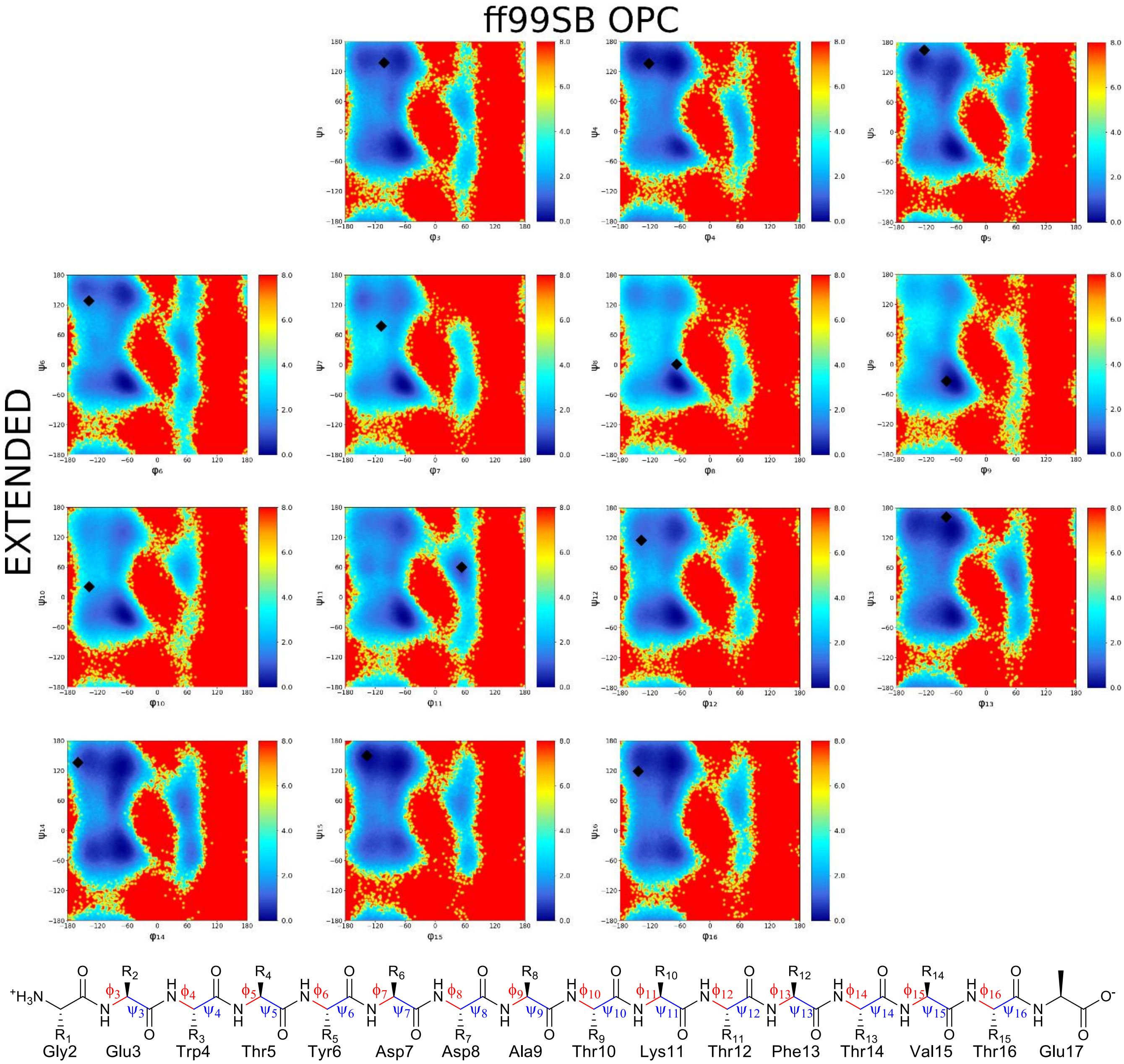

Figure S57. PMFs obtained from φ and ψ dihedral distributions of B1 uncapped residues for the ff99SB/OPC combination from extended (top panel) and misfolded (bottom panels) conformations. PMFs are shown as heat maps (0.0 – 8.0 kcal/mol range) from dark blue to red. The native conformation's φ and ψ angles are reported as black diamonds. B1 uncapped sequence and representation of φ and ψ angles numbering are also shown.

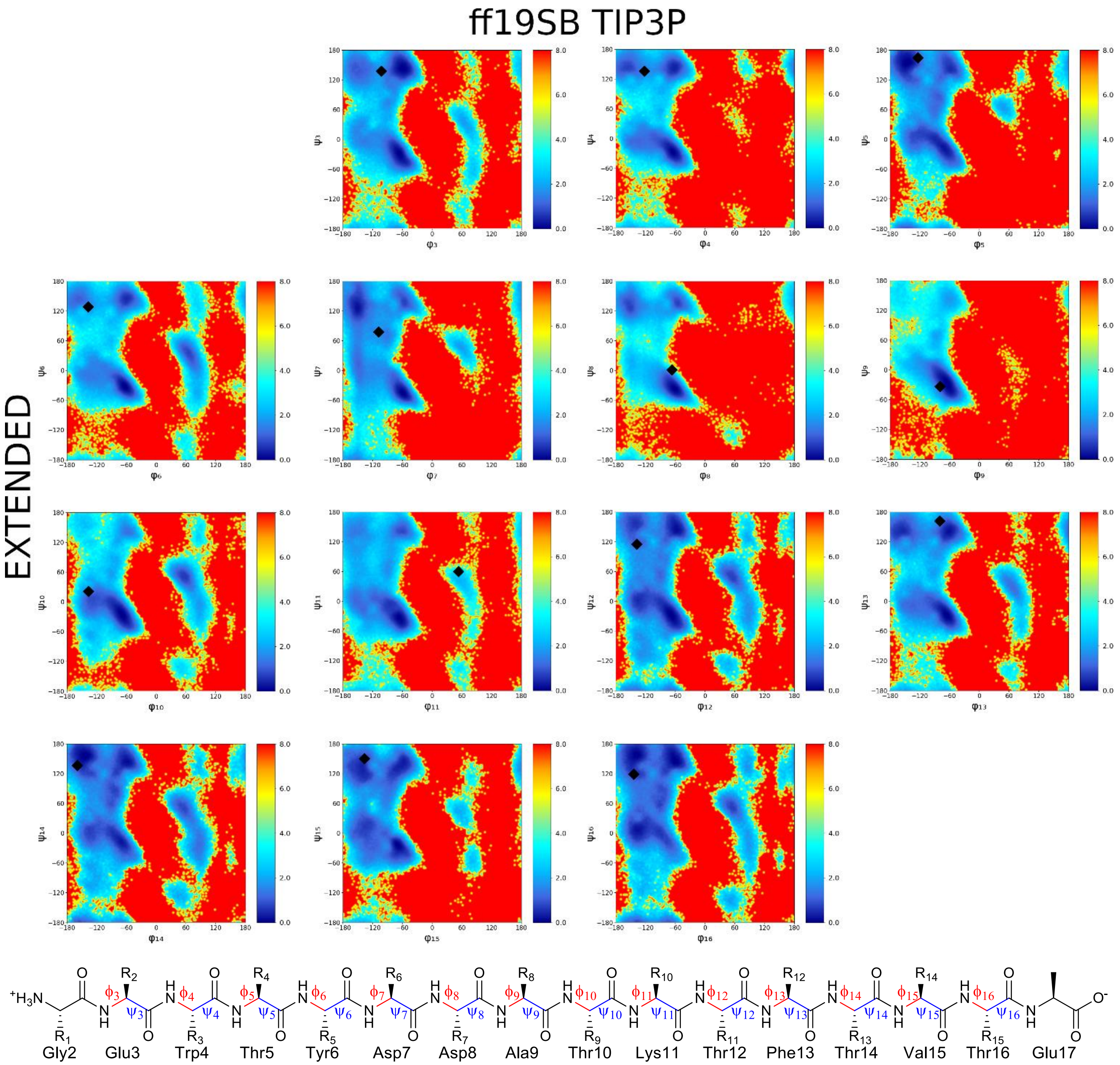

Figure S58. PMFs obtained from φ and ψ dihedral distributions of B1 uncapped residues for the ff99SB/TIP3P combination from extended (top panel) and misfolded (bottom panels) conformations. PMFs are shown as heat maps (0.0 – 8.0 kcal/mol range) from dark blue to red. The native conformation's φ and ψ angles are reported as black diamonds. B1 uncapped sequence and representation of φ and ψ angles numbering are also shown.

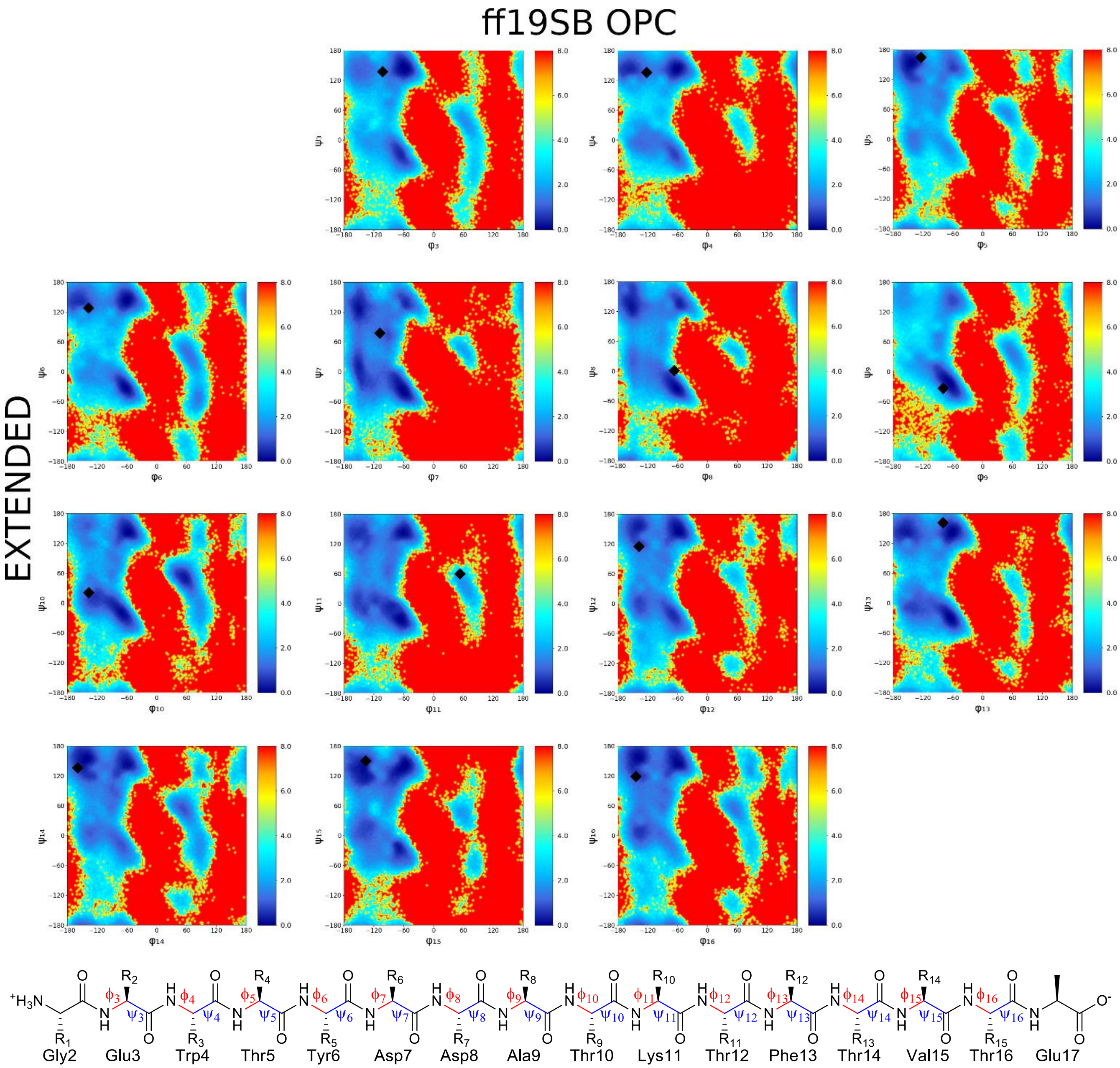

Figure S59. PMFs obtained from φ and ψ dihedral distributions of B1 uncapped residues for the ff19SB/OPC combination from extended (top panel) and misfolded (bottom panels) conformations. PMFs are shown as heat maps (0.0 – 8.0 kcal/mol range) from dark blue to red. The native conformation's φ and ψ angles are reported as black diamonds. B1 uncapped sequence and representation of φ and ψ angles numbering are also shown.

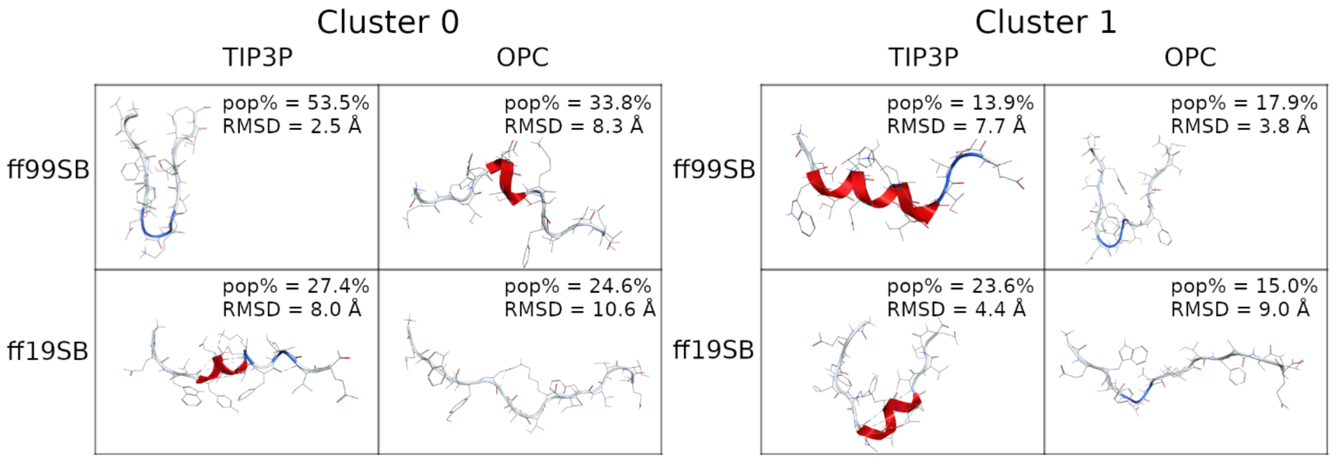

Figure S60. Representative conformation of the main and second clusters from B1 uncapped trajectories. Population (pop%) and RMSD vs native structure are also shown in the figure.

Table S6. H-bond analyses from B1 uncapped trajectories. Only H-bonds involving the backbone are included in the table (donor N-H, acceptor C=O). Native H-bonds are highlighted in bold.

ff99SB OPC		
EXTENDED		
acceptor	donor	occ%
**ASP7**	**LYS11**	15.63
TYR6	THR10	13.4
ASP8	THR12	11.24
ALA9	PHE13	11.05
THR5	ALA9	7.97
THR10	THR14	5.29
TRP4	ASP8	5

ff99SB TIP3P		
EXTENDED		
acceptor	donor	occ%
**THR12**	**ASP7**	33.31
**THR5**	**THR14**	25.12
**ASP7**	**THR12**	17.09
**ASP7**	**LYS11**	15.83
ASP8	THR12	12.2
ALA9	PHE13	10.78
**ASP8**	**LYS11**	8.42
TYR6	THR10	6.36
THR14	THR5	5.64
THR10	THR14	5.33
TRP4	ASP8	5

ff19SB OPC		
EXTENDED		
acceptor	donor	occ%
**ASP8**	**LYS11**	5

ff19SB TIP3P		
EXTENDED		
acceptor	donor	occ%
**ASP7**	**LYS11**	13.68
ASP8	THR12	12.69
ALA9	PHE13	8.22
**ASP8**	**LYS11**	7.85
TYR6	THR10	7.18
**ASP7**	**THR10**	6.5
THR5	ALA9	5.45

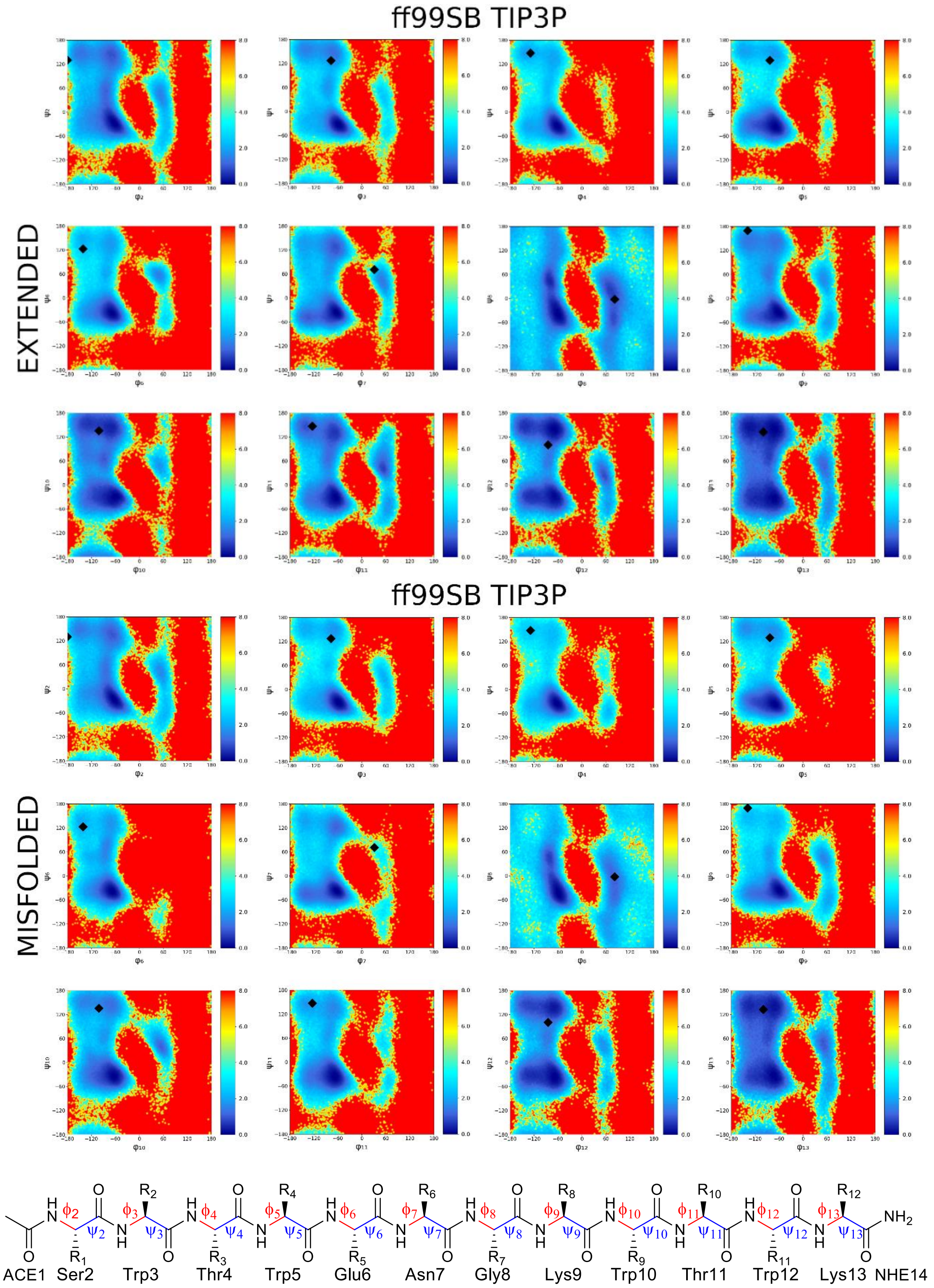

Figure S61. PMFs obtained from φ and ψ dihedral distributions of B2 residues for the ff99SB/TIP3P combination from extended (top panel) and misfolded (bottom panels) conformations. PMFs are shown as heat maps (0.0 – 8.0 kcal/mol range) from dark blue to red. The native conformation's φ and ψ angles are reported as black diamonds. B2 sequence and representation of φ and ψ angles numbering are also shown.

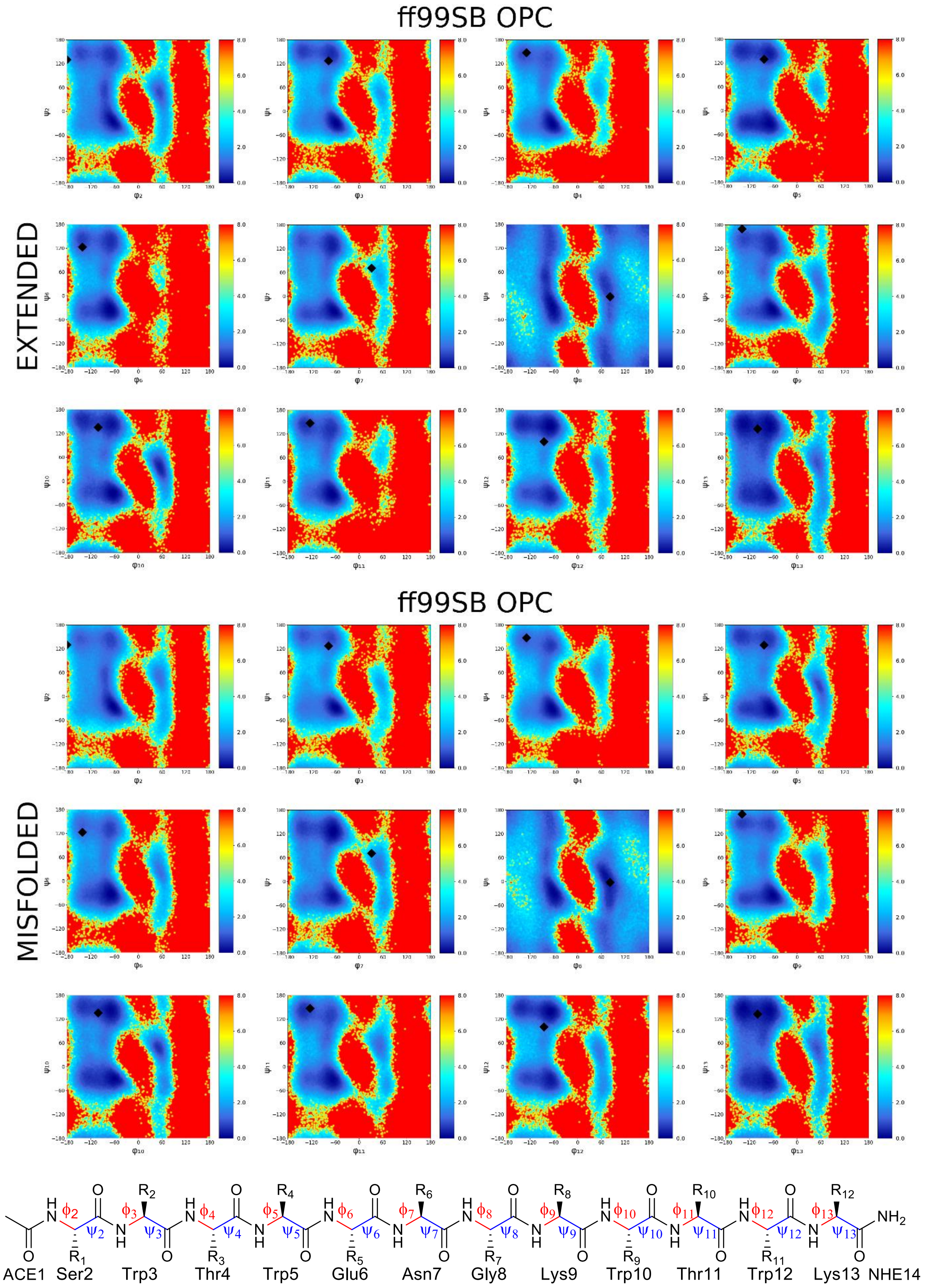

Figure S62. PMFs obtained from φ and ψ dihedral distributions of B2 residues for the ff99SB/OPC combination from extended (top panel) and misfolded (bottom panels) conformations. PMFs are shown as heat maps (0.0 – 8.0 kcal/mol range) from dark blue to red. The native conformation's φ and ψ angles are reported as black diamonds. B2 sequence and representation of φ and ψ angles numbering are also shown.

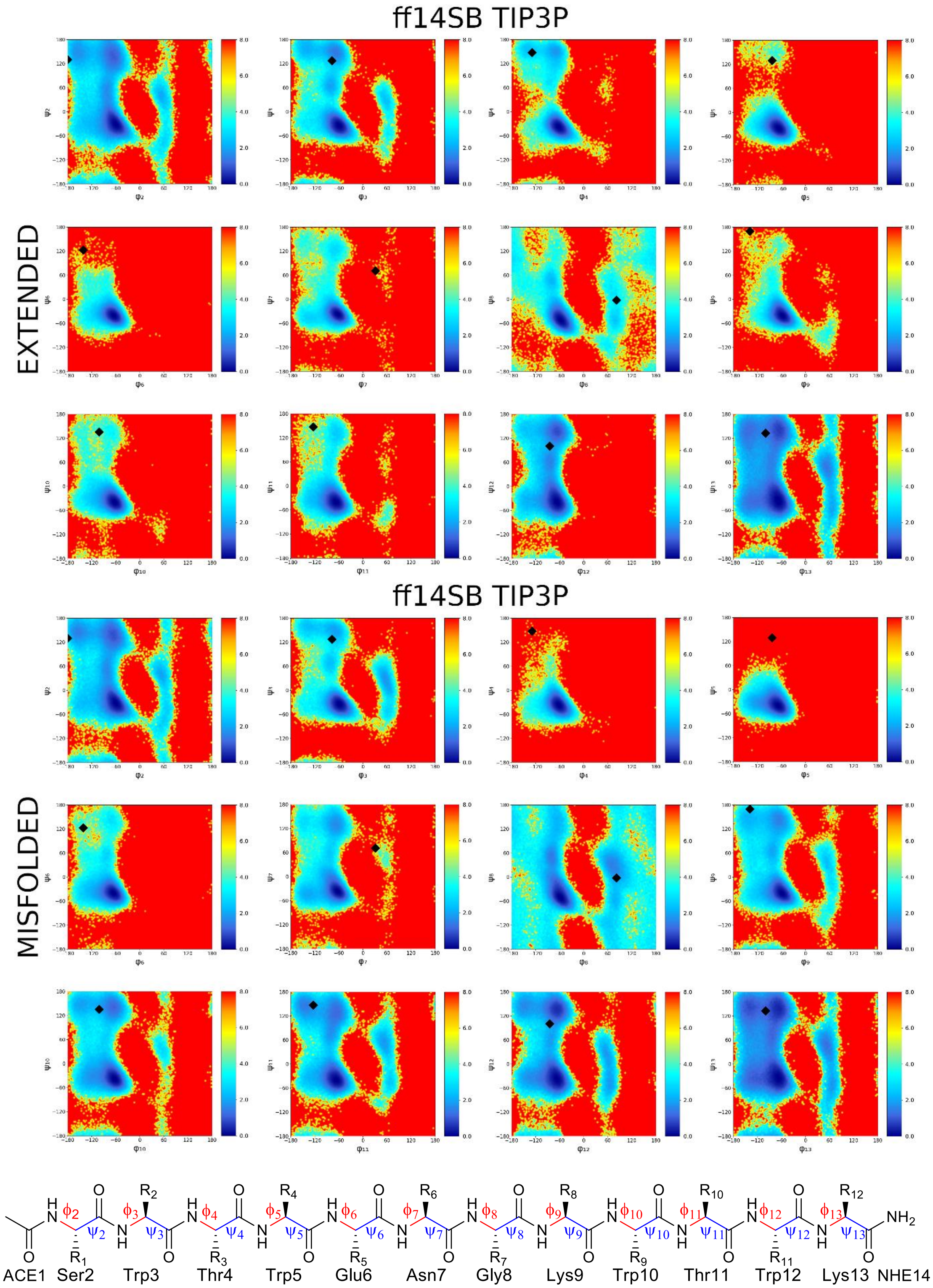

Figure S63. PMFs obtained from φ and ψ dihedral distributions of B2 residues for the ff14SB/TIP3P combination from extended (top panel) and misfolded (bottom panels) conformations. PMFs are shown as heat maps (0.0 – 8.0 kcal/mol range) from dark blue to red. The native conformation's φ and ψ angles are reported as black diamonds. B2 sequence and representation of φ and ψ angles numbering are also shown.

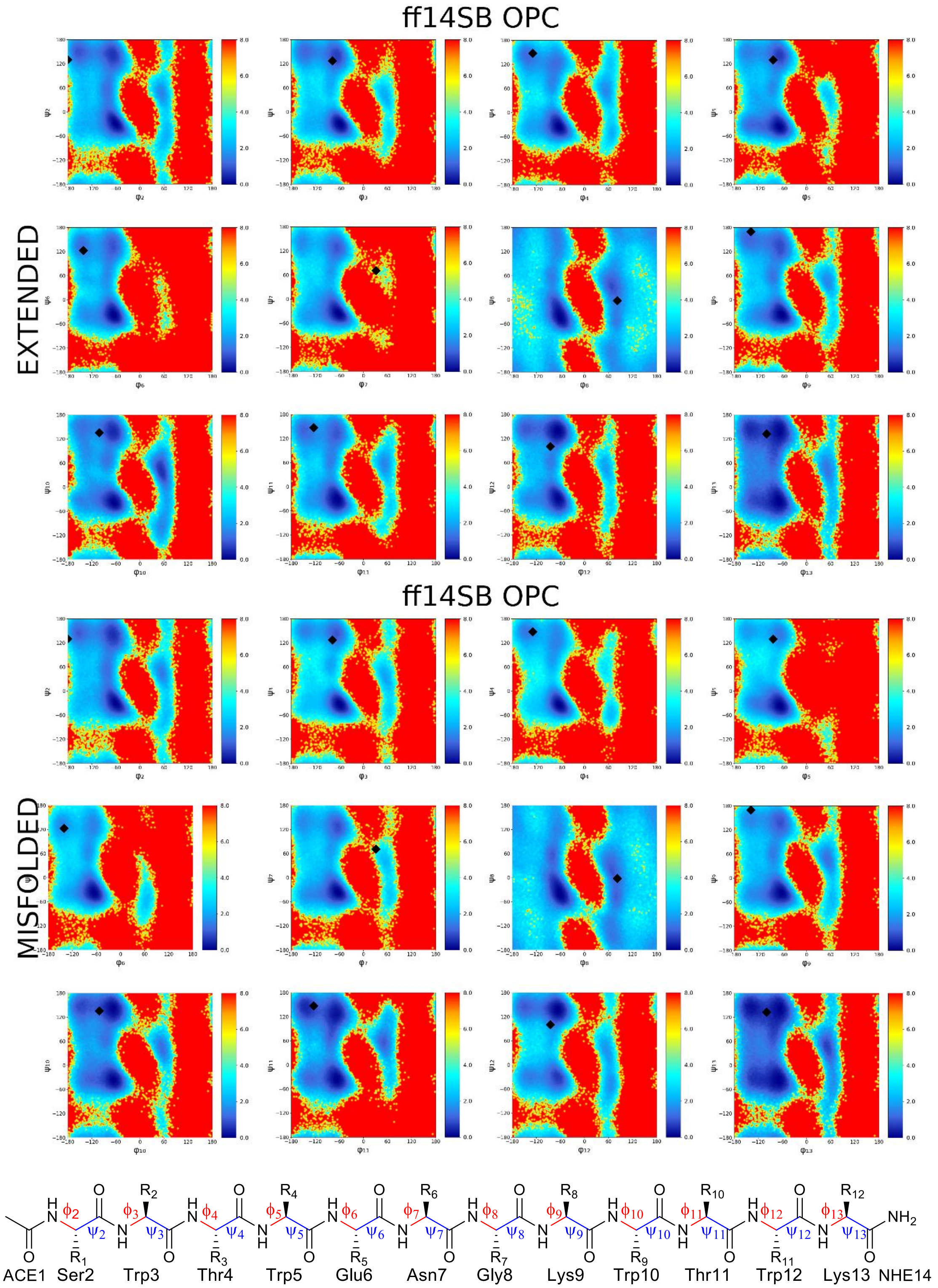

Figure S64. PMFs obtained from φ and ψ dihedral distributions of B2 residues for the ff14SB/OPC combination from extended (top panel) and misfolded (bottom panels) conformations. PMFs are shown as heat maps (0.0 – 8.0 kcal/mol range) from dark blue to red. The native conformation's φ and ψ angles are reported as black diamonds. B2 sequence and representation of φ and ψ angles numbering are also shown.

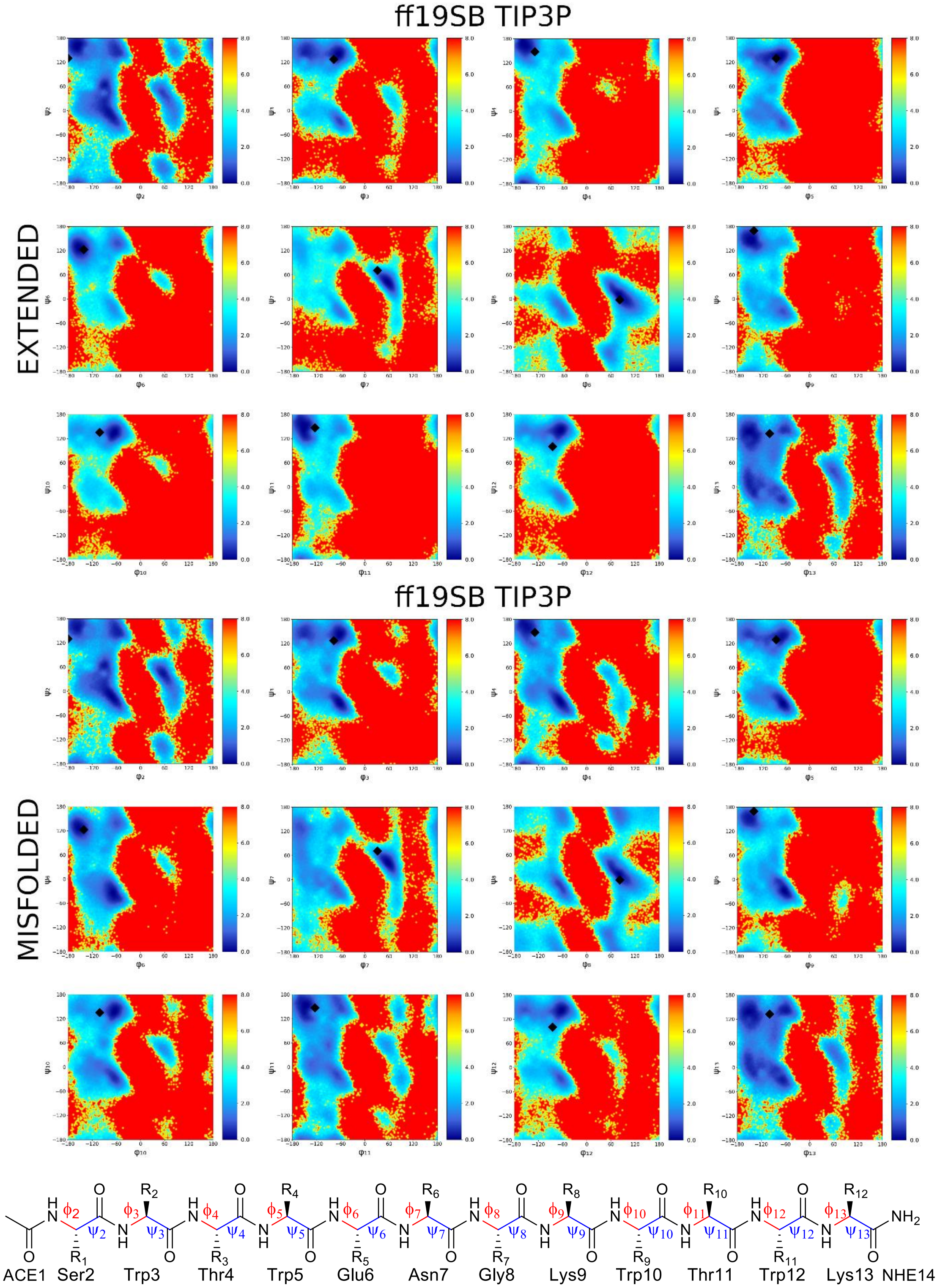

Figure S65. PMFs obtained from φ and ψ dihedral distributions of B2 residues for the ff19SB/TIP3P combination from extended (top panel) and misfolded (bottom panels) conformations. PMFs are shown as heat maps (0.0 – 8.0 kcal/mol range) from dark blue to red. The native conformation's φ and ψ angles are reported as black diamonds.

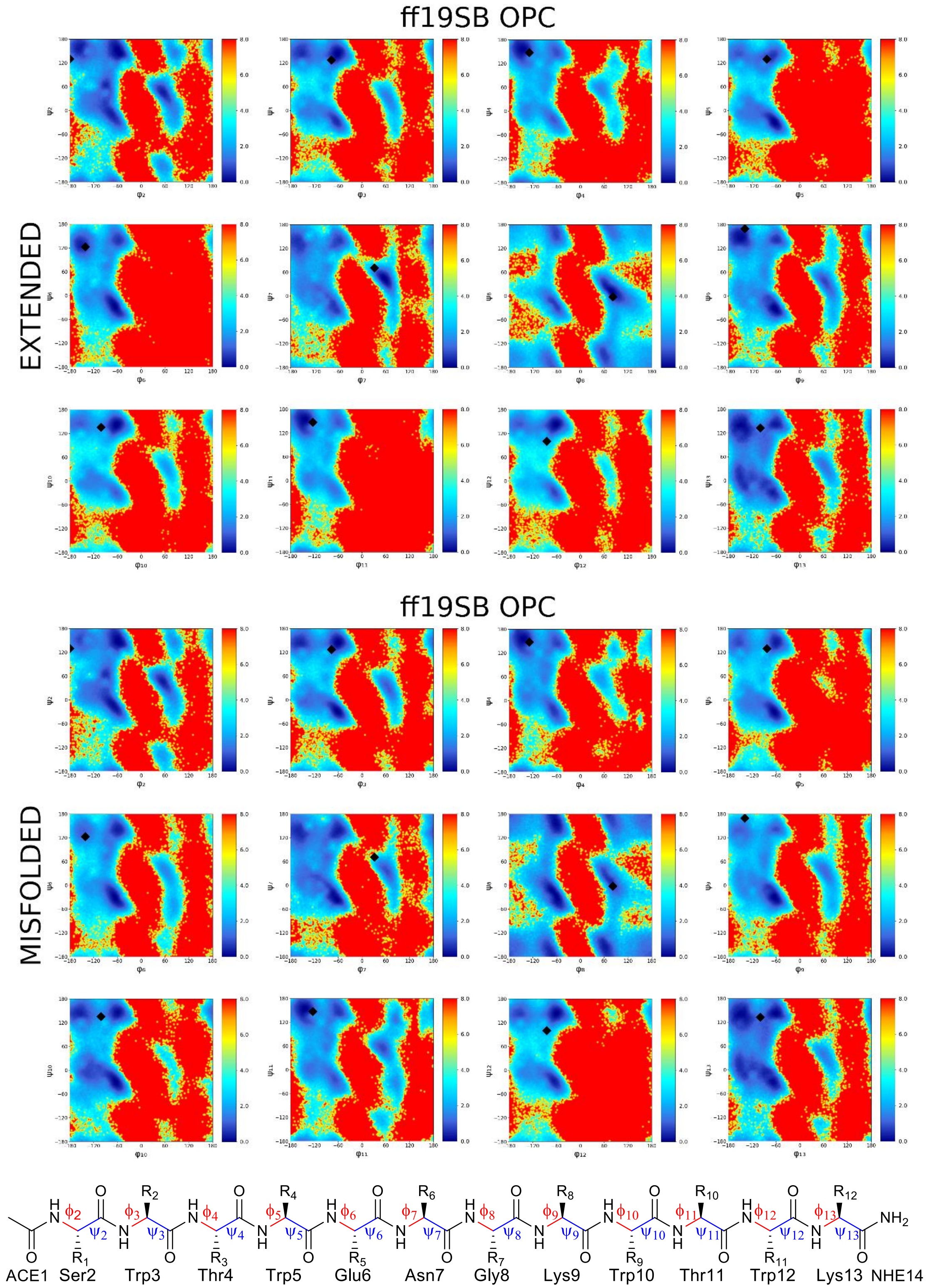

Figure S66. PMFs obtained from φ and ψ dihedral distributions of B2 residues for the ff19SB/OPC combination from extended (top panel) and misfolded (bottom panels) conformations. PMFs are shown as heat maps (0.0 – 8.0 kcal/mol range) from dark blue to red. The native conformation's φ and ψ angles are reported as black diamonds. B2 sequence and representation of φ and ψ angles numbering are also shown.

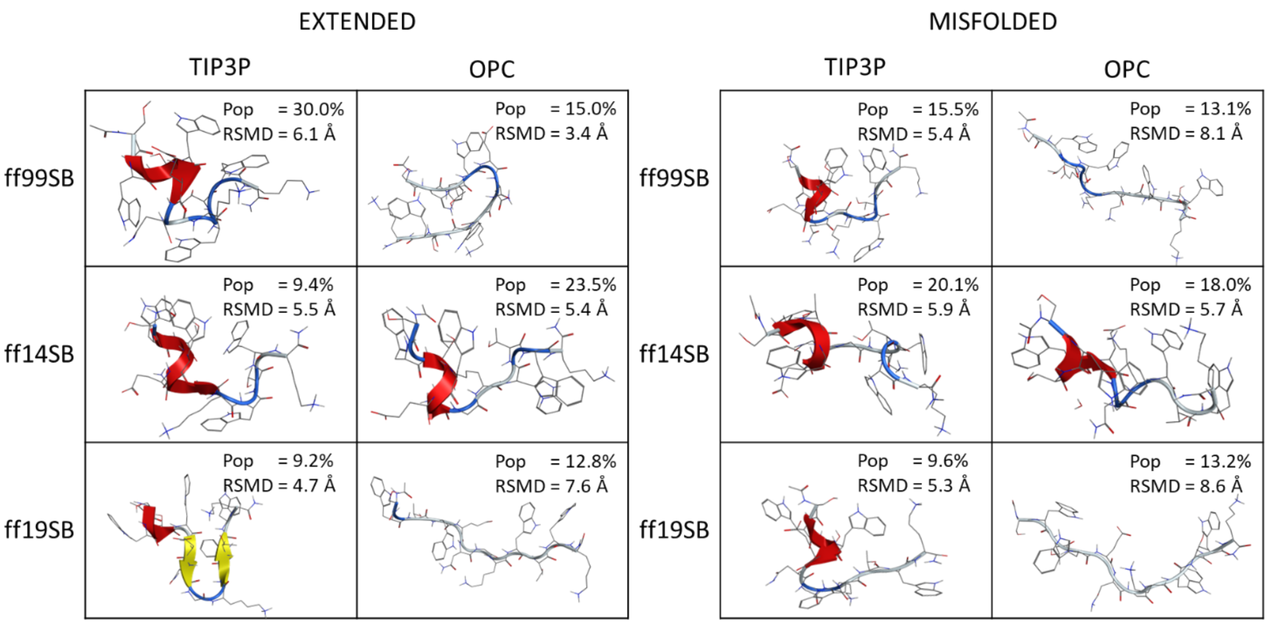

Figure S67. Representative conformation of the second cluster from B2 trajectories. Population (pop%) and RMSD vs native structure are also shown in the figure.

Table S7. H-bond analyses from B2 trajectories. Only H-bonds involving the backbone are included in the table (donor N-H, acceptor C=O). Native H-bonds are highlighted in bold.

NATIVE		
donor	acceptor	Distance
**SER2**	**LYS13**	**2.74**
**LYS9**	**GLU6**	**2.84**
**THR4**	**THR11**	**2.9**
**GLU6**	**LYS9**	**2.92**
**LYS13**	**SER2**	**2.96**
**THR11**	**THR4**	**2.99**
**GLU6**	**GLY8**	**3.31**
**THR11**	**LYS13**	**3.37**
**ASN7**	**LYS9**	**3.47**
**SER2**	**THR4**	**3.62**
**THR4**	**GLU6**	**3.78**
**TRP12**	**NME**	**4.15**
**LYS9**	**THR11**	**4.21**
**THR11**	**TRP3**	**4.47**
**SER2**	**TRP12**	**4.47**
**LYS9**	**TRP5**	**4.49**

ff99SB OPC						ff99SB TIP3P					
EXTENDED			MISFOLDED			EXTENDED			MISFOLDED		
donor	acceptor	occ%	donor	acceptor	occ%	donor	acceptor	occ%	donor	acceptor	occ%
SER2	GLU6	10.61	TRP3	ASN7	9.59	TRP3	ASN7	28.69	TRP3	ASN7	30.04

TRP3	ASN7	8.46	SER2	GLU6	9.54	SER2	GLU6	19.53	SER2	GLU6	24.18
SER2	TRP5	6.53	GLU6	TRP10	6.92	THR4	GLY8	11.81	GLU6	TRP10	15.15
LYS9	LYS13	5.14	ACE1	THR4	6.15	TRP5	LYS9	10.37	GLY8	TRP12	14.70
			SER2	TRP5	5.83	GLU6	TRP10	10.22	LYS9	LYS13	13.42
			**GLU6**	**LYS9**	5.25	SER2	TRP5	10.03	ASN7	THR11	12.85
			ACE1	TRP3	5.04	TRP3	GLY8	9.55	THR4	GLY8	12.51
			**ASN7**	**LYS9**	5.02	**ASN7**	**LYS9**	9.43	SER2	TRP5	12.14
						LYS9	LYS13	7.70	TRP3	GLY8	10.66
						ACE1	THR4	7.06	TRP5	LYS9	10.02
						ASN7	THR11	5.76	ACE1	THR4	9.21
						TRP3	GLU6	5.65	**ASN7**	**LYS9**	8.61
									GLY8	THR11	6.92
									**GLU6**	**LYS9**	6.57
									ACE1	TRP5	6.26
									GLY8	LYS13	5.13

ff14SB OPC					
EXTENDED			MISFOLDED		
donor	acceptor	occ%	donor	acceptor	occ%
GLU6	TRP10	12.89	TRP3	ASN7	18.92
SER2	GLU6	12.83	SER2	GLU6	15.66
TRP3	ASN7	10.45	THR4	GLY8	10.85
ASN7	THR11	10.33	GLU6	TRP10	9.50
THR4	GLY8	7.23	SER2	TRP5	8.25
GLY8	TRP12	7.20	ASN7	THR11	7.26
SER2	TRP5	6.86	ACE1	THR4	6.42
ACE1	THR4	6.65	TRP5	LYS9	5.89
**GLU6**	**LYS9**	5.15	**ASN7**	**LYS9**	5.33

ff14SB TIP3P					
EXTENDED			MISFOLDED		
donor	acceptor	occ%	donor	acceptor	occ%
TRP3	ASN7	44.90	TRP3	ASN7	46.71
GLY8	TRP12	42.56	SER2	GLU6	25.95
TRP5	LYS9	39.18	THR4	GLY8	22.48
ASN7	THR11	36.49	TRP5	LYS9	22.47
THR4	GLY8	33.59	GLY8	TRP12	22.33
GLU6	TRP10	32.53	ASN7	THR11	21.49
LYS9	LYS13	23.56	GLU6	TRP10	18.96
SER2	GLU6	20.88	LYS9	LYS13	15.37
ACE1	THR4	10.49	SER2	TRP5	9.74
GLY8	THR11	8.97	ACE1	THR4	9.21
ACE1	TRP5	6.69	ACE1	TRP5	7.78
SER2	TRP5	6.29	TRP3	GLU6	6.71
			GLY8	THR11	5.46

ff19SB OPC					
EXTENDED			MISFOLDED		
donor	acceptor	occ%	donor	acceptor	occ%
**THR4**	**THR11**	16.90	TRP3	ASN7	22.38
**THR11**	**THR4**	15.63	SER2	GLU6	21.11
**SER2**	**LYS13**	12.73	GLU6	TRP10	17.28
**LYS9**	**GLU6**	12.19	GLY8	TRP12	15.13
			ASN7	THR11	13.77
			TRP5	LYS9	12.00
			THR4	GLY8	11.87
			LYS9	LYS13	8.02
			ACE1	TRP5	7.77
			TRP3	GLY8	6.10
			ACE1	THR4	5.97
			SER2	TRP5	5.96

ff19SB TIP3P					
EXTENDED			MISFOLDED		
donor	acceptor	occ%	donor	acceptor	occ%
**THR4**	**THR11**	53.39	**THR4**	**THR11**	24.54
**THR11**	**THR4**	45.56	**LYS9**	**GLU6**	20.21
**SER2**	**LYS13**	45.01	**THR11**	**THR4**	17.66
**LYS9**	**GLU6**	40.83	**SER2**	**LYS13**	16.02
**GLU6**	**LYS9**	17.20	GLY8	THR11	8.47
**LYS13**	**SER2**	7.96	**GLU6**	**LYS9**	6.31
ACE1	TRP3	5.30	TRP3	GLU6	5.88
			TRP3	ASN7	5.41
			THR4	LYS13	5.03

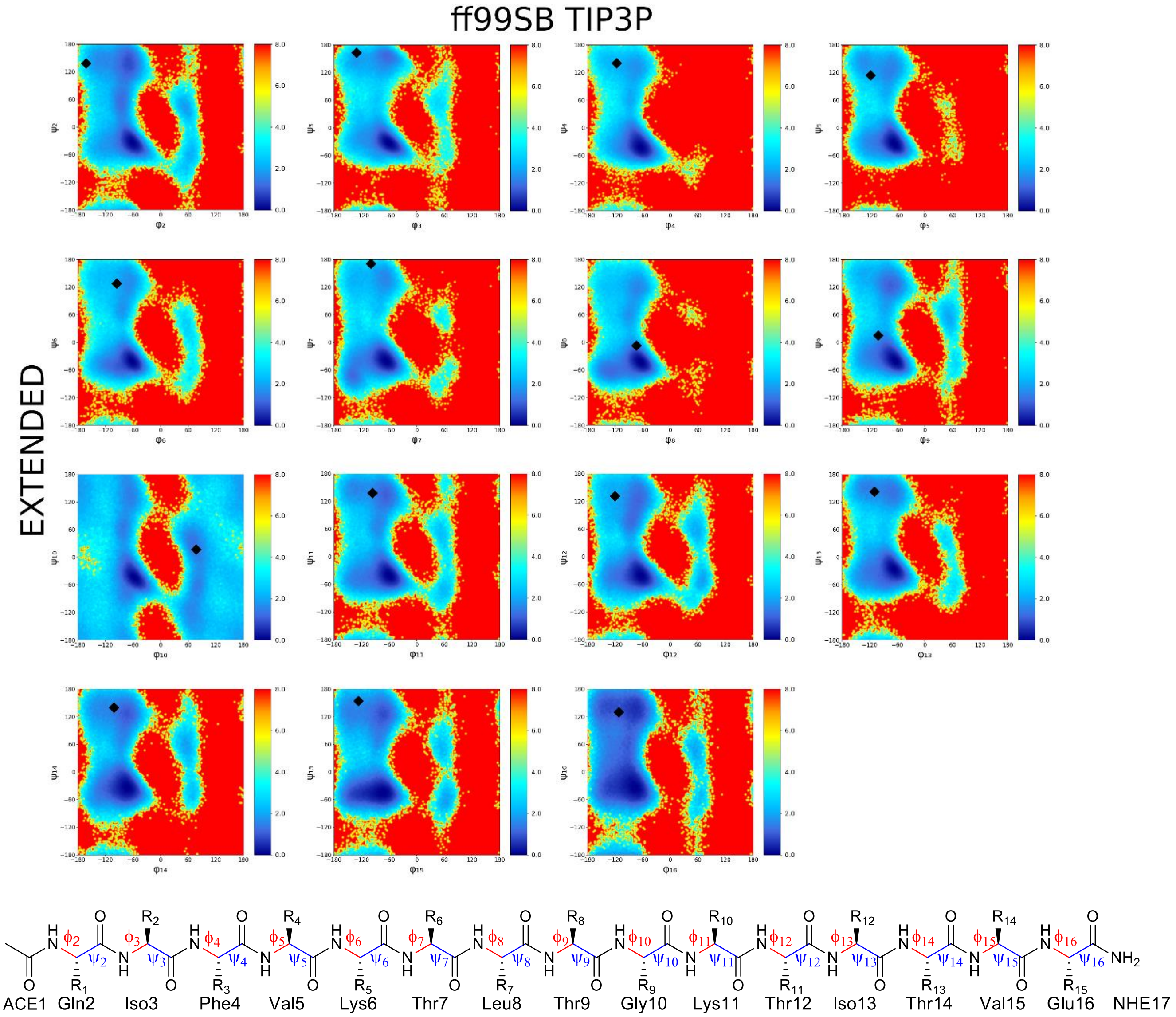

Figure S68. PMFs obtained from φ and ψ dihedral distributions of B3 residues for the ff99SB/TIP3P combination from an extended conformation. PMFs are shown as heat maps (0.0 – 8.0 kcal/mol range) from dark blue to red. The native conformation's φ and ψ angles are reported as black diamonds. B3 sequence and representation of φ and ψ angles numbering are also shown.

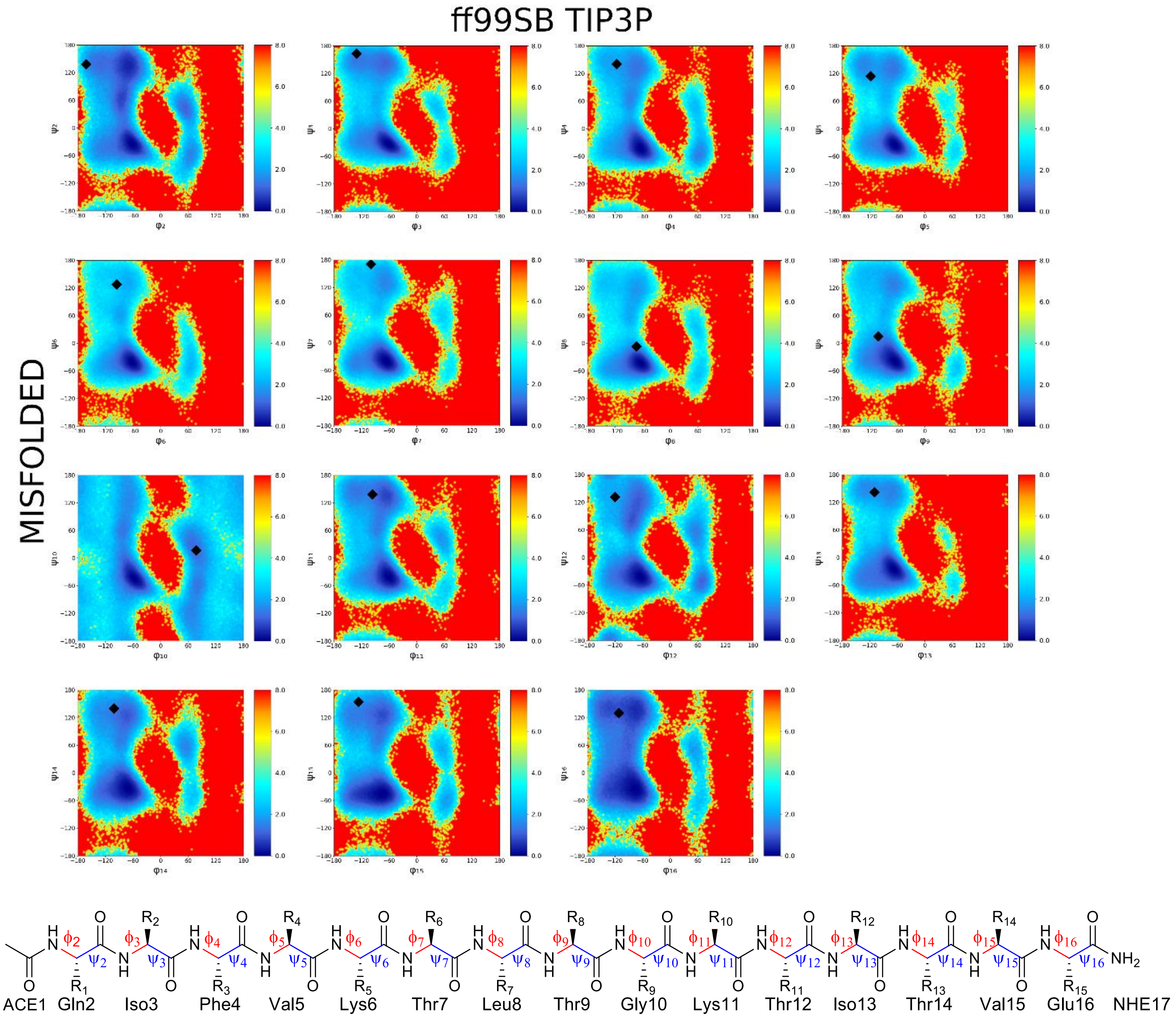

Figure S69. PMFs obtained from φ and ψ dihedral distributions of B3 residues for the ff99SB/TIP3P combination from a misfolded conformation. PMFs are shown as heat maps (0.0 – 8.0 kcal/mol range) from dark blue to red. The native conformation's φ and ψ angles are reported as black diamonds. B3 sequence and representation of φ and ψ angles numbering are also shown.

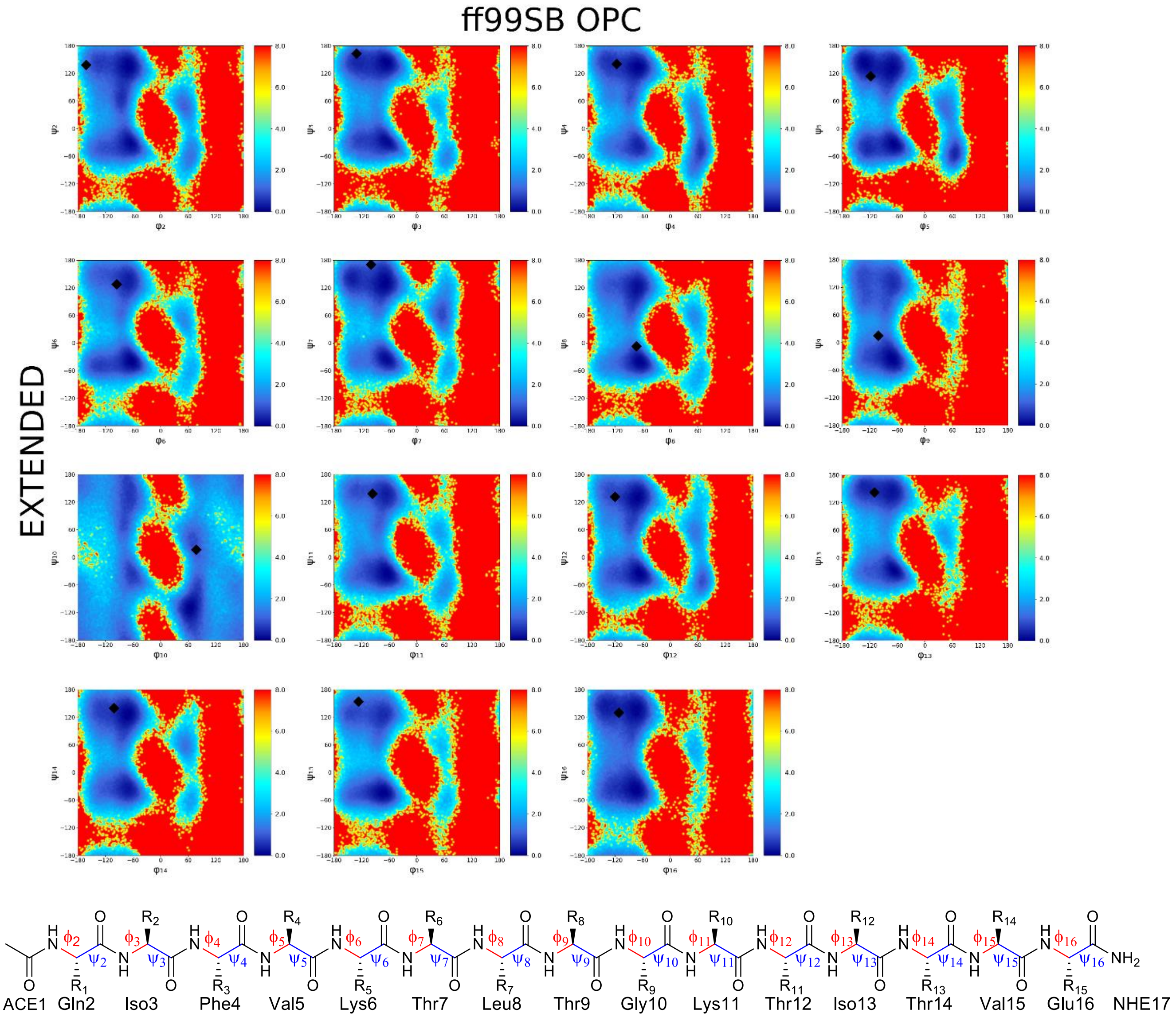

Figure S70. PMFs obtained from φ and ψ dihedral distributions of B3 residues for the ff99SB/OPC combination from an extended conformation. PMFs are shown as heat maps (0.0 – 8.0 kcal/mol range) from dark blue to red. The native conformation's φ and ψ angles are reported as black diamonds. B3 sequence and representation of φ and ψ angles numbering are also shown.

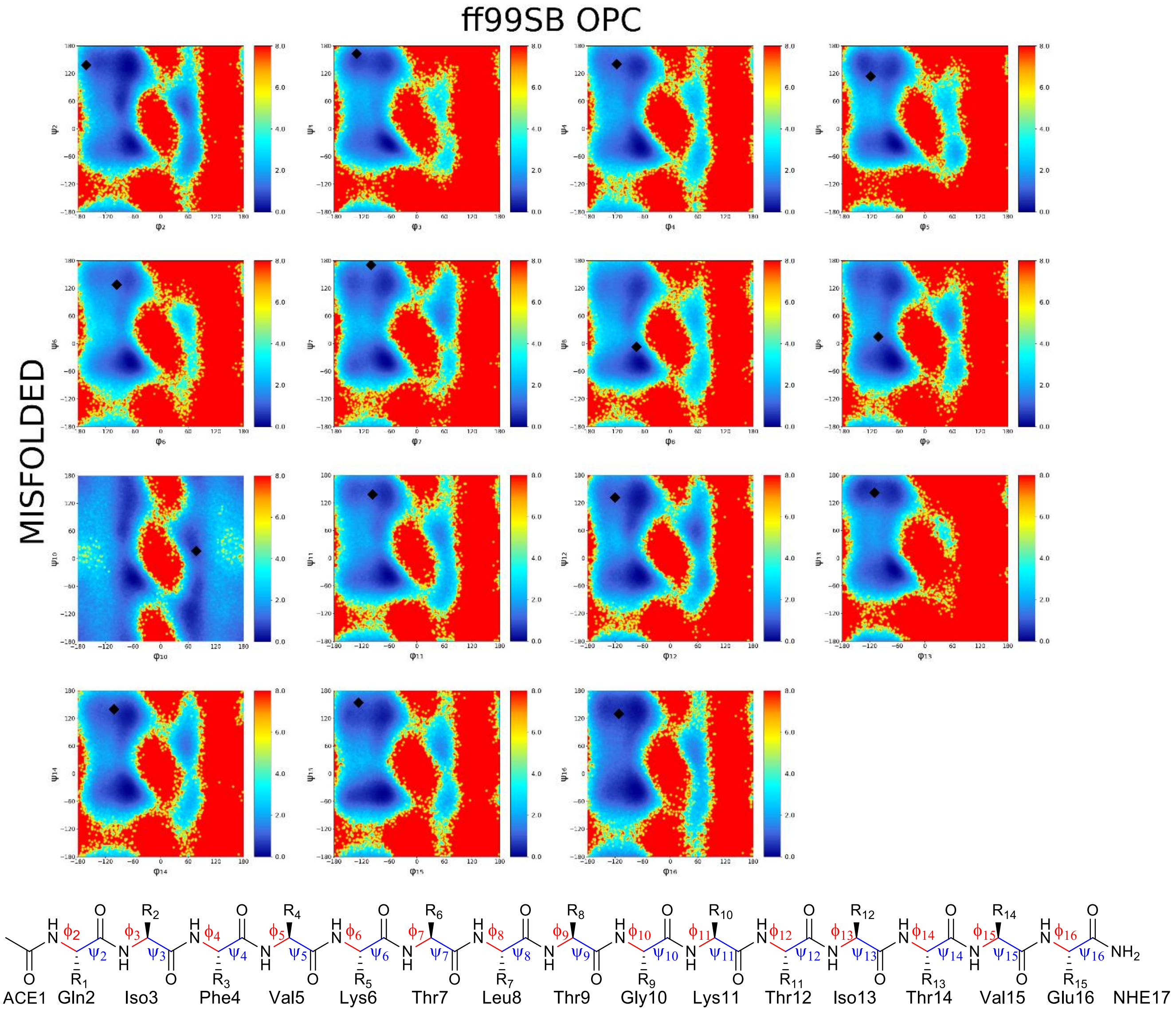

Figure S71. PMFs obtained from φ and ψ dihedral distributions of B3 residues for the ff99SB/OPC combination from a misfolded conformation. PMFs are shown as heat maps (0.0 – 8.0 kcal/mol range) from dark blue to red. The native conformation's φ and ψ angles are reported as black diamonds. B3 sequence and representation of φ and ψ angles numbering are also shown.

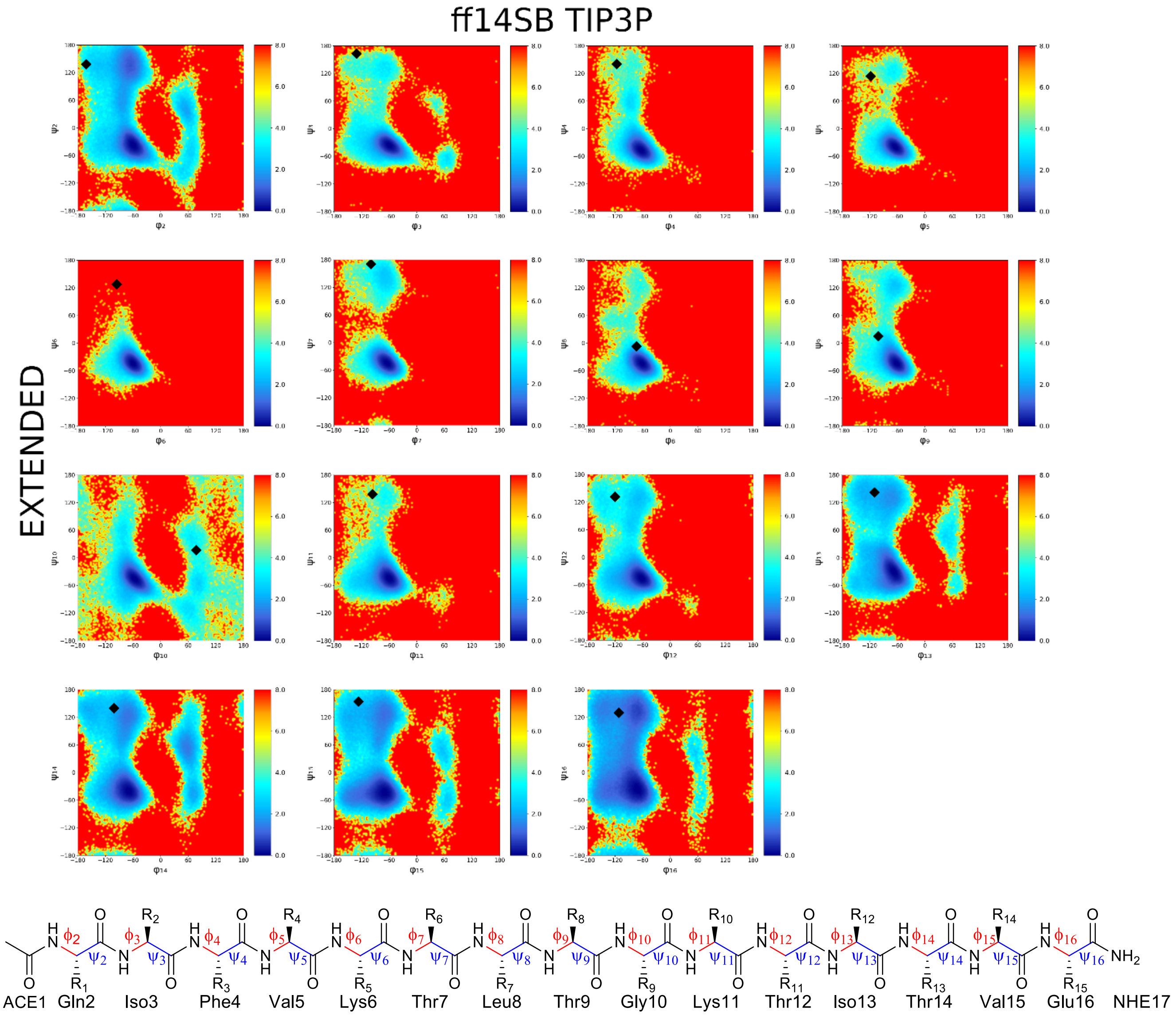

Figure S72. PMFs obtained from φ and ψ dihedral distributions of B3 residues for the ff14SB/TIP3P combination from an extended conformation. PMFs are shown as heat maps (0.0 – 8.0 kcal/mol range) from dark blue to red. The native conformation's φ and ψ angles are reported as black diamonds. B3 sequence and representation of φ and ψ angles numbering are also shown.

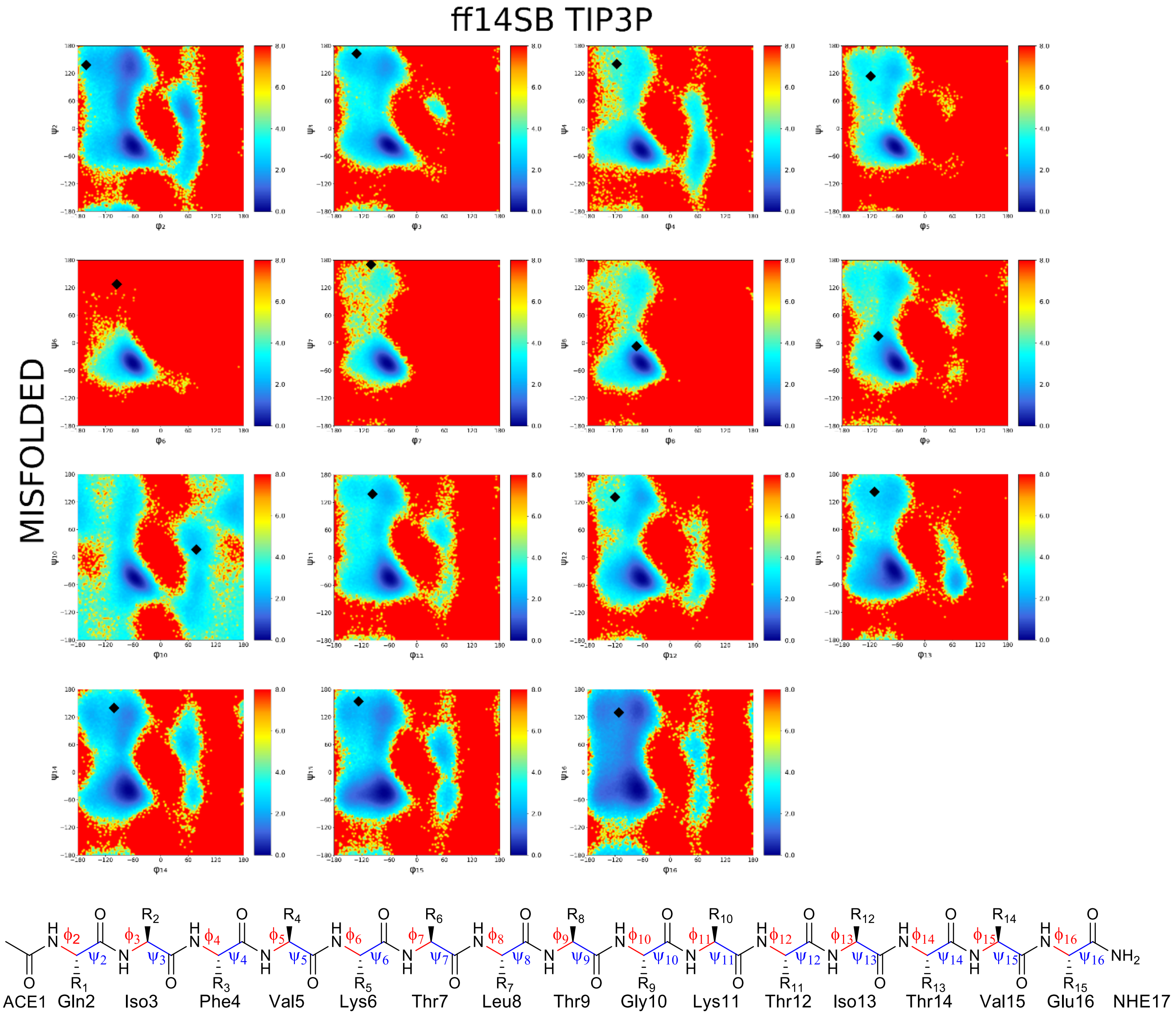

Figure S73. PMFs obtained from φ and ψ dihedral distributions of B3 residues for the ff14SB/TIP3P combination from a misfolded conformation. PMFs are shown as heat maps (0.0 – 8.0 kcal/mol range) from dark blue to red. The native conformation's φ and ψ angles are reported as black diamonds. B3 sequence and representation of φ and ψ angles numbering are also shown.

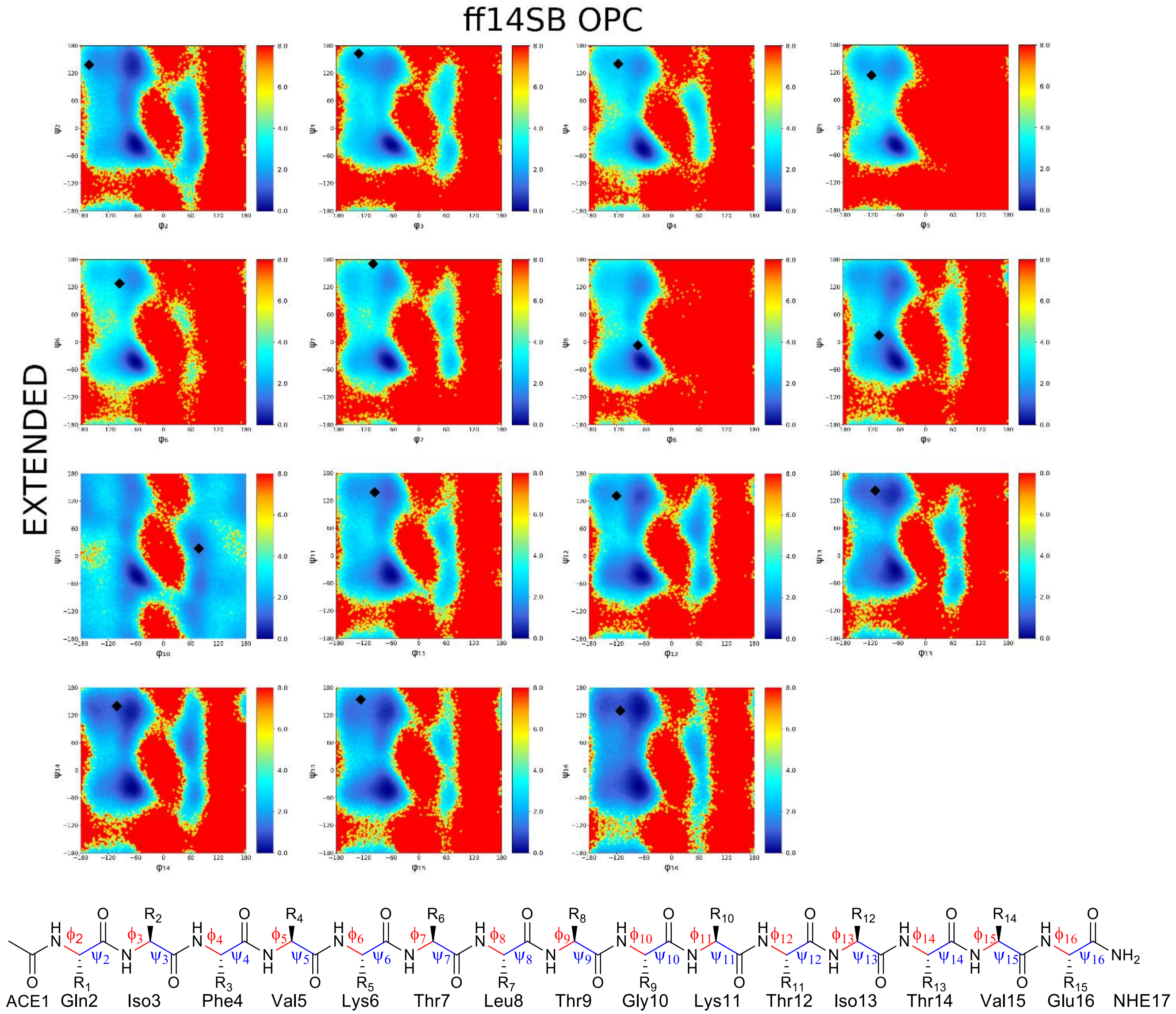

Figure S74. PMFs obtained from φ and ψ dihedral distributions of B3 residues for the ff14SB/OPC combination from an extended conformation. PMFs are shown as heat maps (0.0 – 8.0 kcal/mol range) from dark blue to red. The native conformation's φ and ψ angles are reported as black diamonds. B3 sequence and representation of φ and ψ angles numbering are also shown.

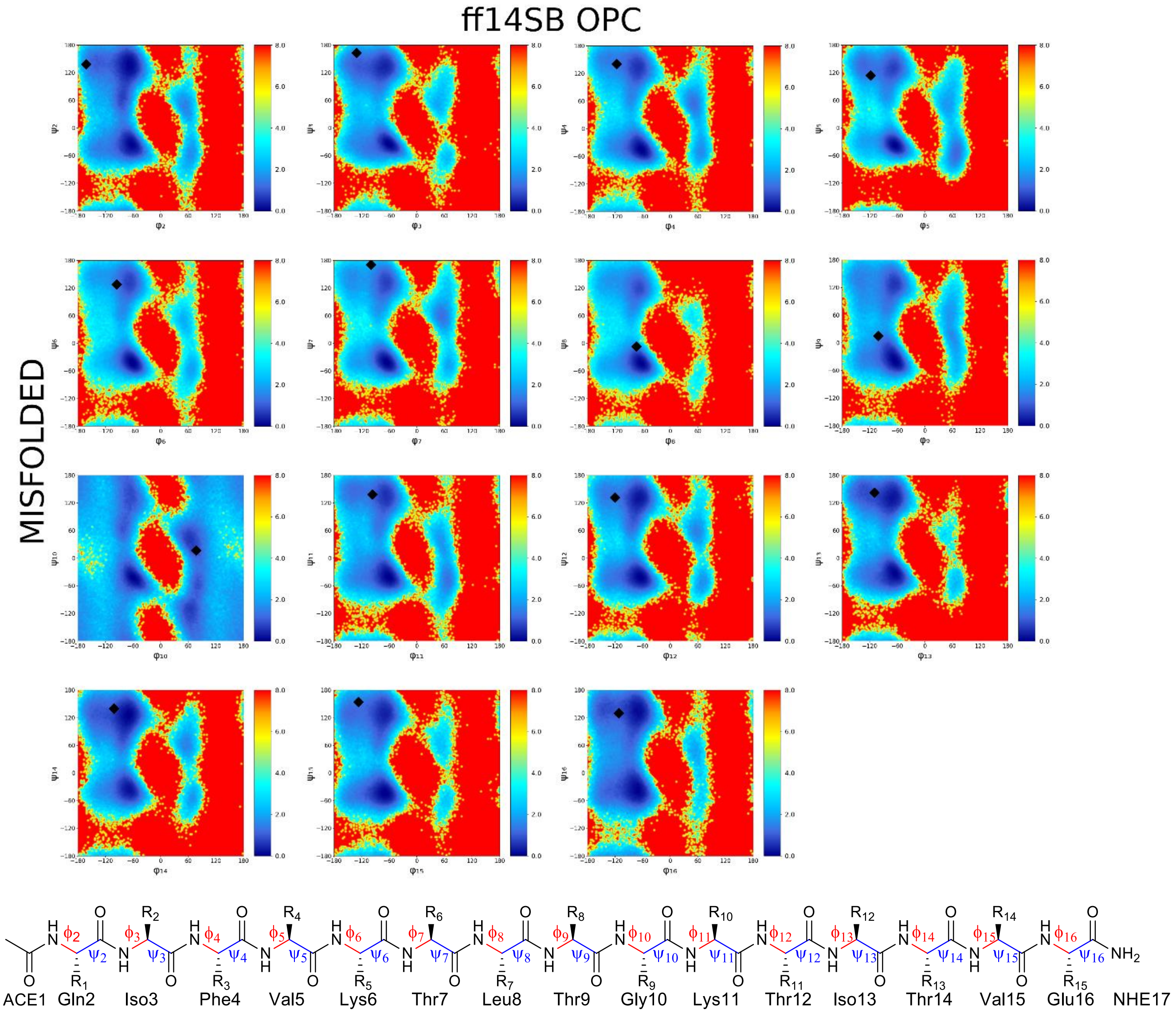

Figure S75. PMFs obtained from φ and ψ dihedral distributions of B3 residues for the ff14SB/OPC combination from a misfolded conformation. PMFs are shown as heat maps (0.0 – 8.0 kcal/mol range) from dark blue to red. The native conformation's φ and ψ angles are reported as black diamonds. B3 sequence and representation of φ and ψ angles numbering are also shown.

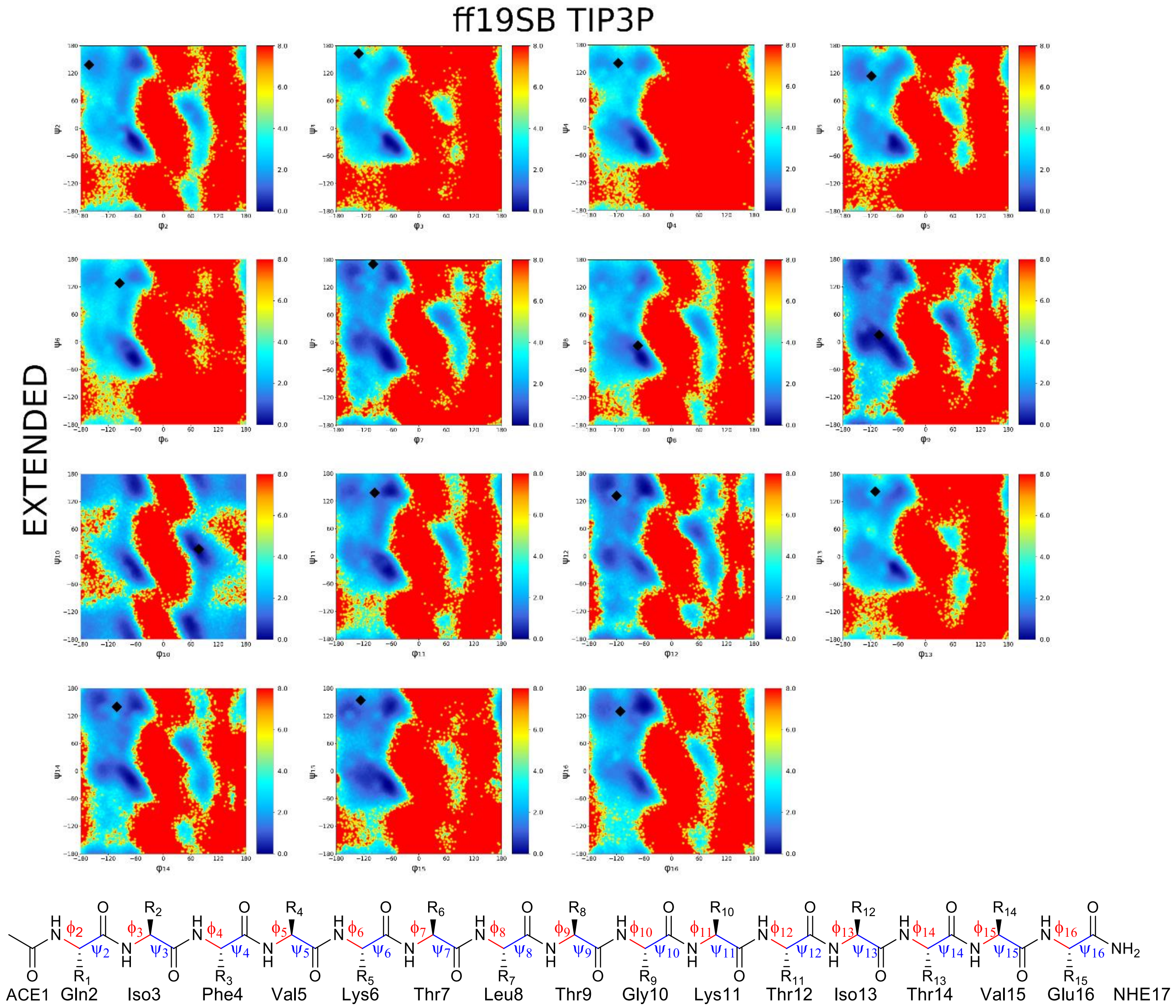

Figure S76. PMFs obtained from φ and ψ dihedral distributions of B3 residues for the ff19SB/TIP3P combination from an extended conformation. PMFs are shown as heat maps (0.0 – 8.0 kcal/mol range) from dark blue to red. The native conformation's φ and ψ angles are reported as black diamonds. B3 sequence and representation of φ and ψ angles numbering are also shown.

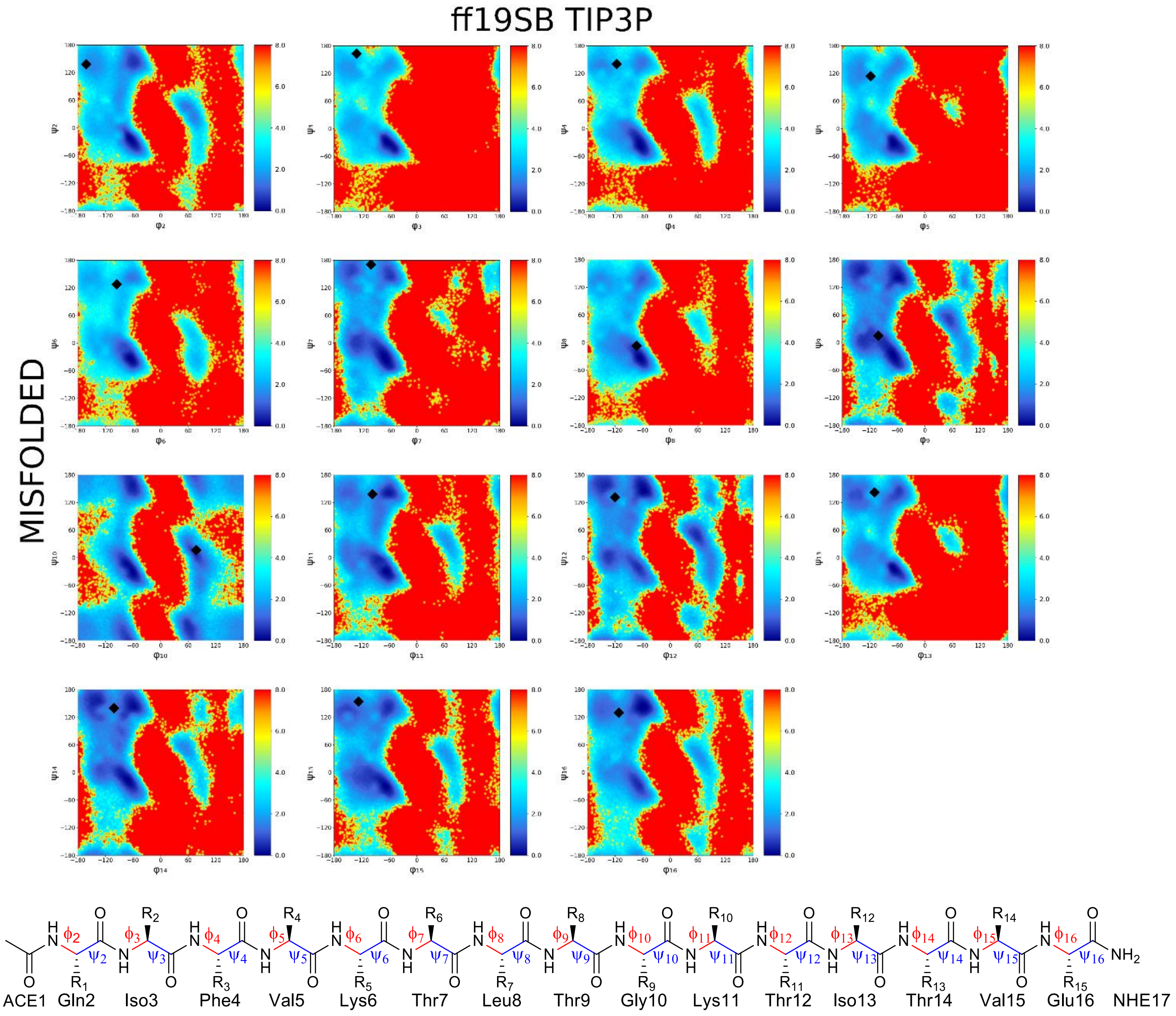

Figure S77. PMFs obtained from φ and ψ dihedral distributions of B3 residues for the ff19SB/TIP3P combination from a misfolded conformation. PMFs are shown as heat maps (0.0 – 8.0 kcal/mol range) from dark blue to red. The native conformation's φ and ψ angles are reported as black diamonds. B3 sequence and representation of φ and ψ angles numbering are also shown.

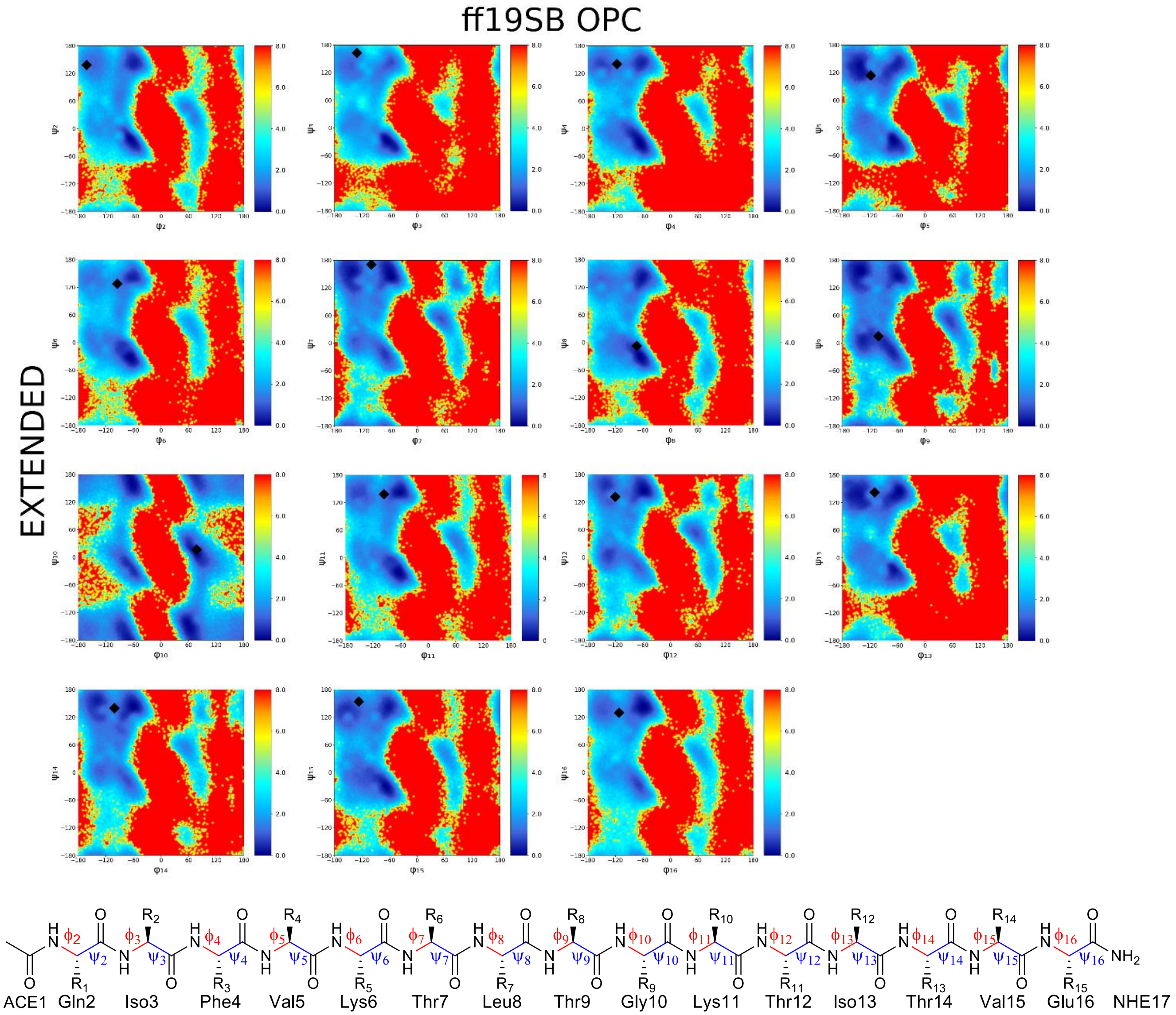

Figure S78. PMFs obtained from φ and ψ dihedral distributions of B3 residues for the ff19SB/OPC combination from an extended conformation. PMFs are shown as heat maps (0.0 – 8.0 kcal/mol range) from dark blue to red. The native conformation's φ and ψ angles are reported as black diamonds. B3 sequence and representation of φ and ψ angles numbering are also shown.

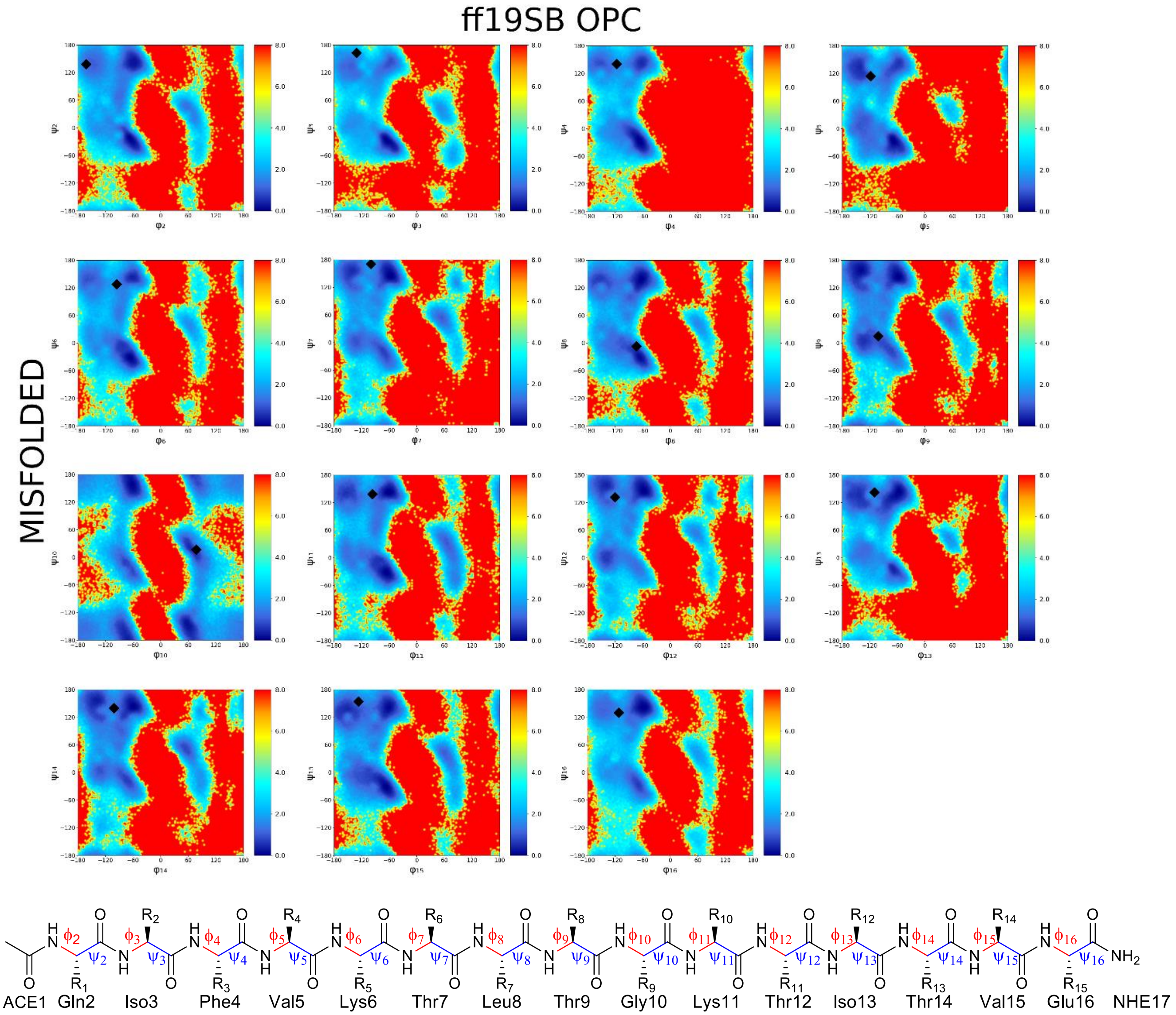

Figure S79. PMFs obtained from φ and ψ dihedral distributions of B3 residues for the ff19SB/OPC combination from a misfolded conformation. PMFs are shown as heat maps (0.0 – 8.0 kcal/mol range) from dark blue to red. The native conformation's φ and ψ angles are reported as black diamonds. B3 sequence and representation of φ and ψ angles numbering are also shown.

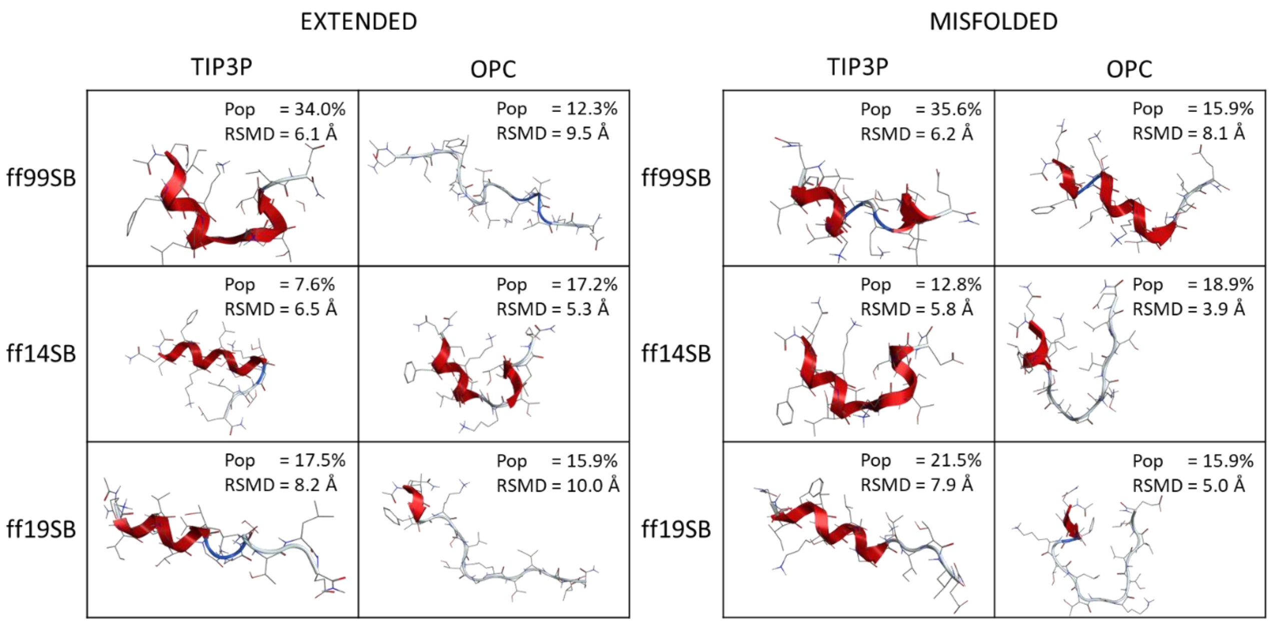

Figure S80. Representative conformation and population (pop%) of the second cluster from B3 trajectories. RMSD vs native structure are also shown.

Table S8. H-bond analyses from B3 trajectories. Only H-bonds involving the backbone are included in the table (donor N-H, acceptor C=O). Native H-bonds are highlighted in bold.

NATIVE		
acceptor	donor	Distance
VAL5	ILE13	2.72
ILE13	VAL5	2.79
LYS11	THR7	2.91
ILE3	LEU15	3.05
LEU15	ILE3	3.05
THR7	THR9	3.15
THR7	GLY10	3.37
THR9	LYS11	3.42
LEU8	GLY10	3.6
VAL5	THR7	3.9
GLY10	THR12	4.02
ILE13	LEU15	4.02
PHE4	LYS6	4.12
LEU15	NME	4.15
THR12	THR14	4.17
LYS11	ILE13	4.29
THR7	LYS11	4.3
LYS6	LEU8	4.43
ILE3	VAL5	4.45
LEU15	GLN2	4.47

ff99SB OPC	ff99SB TIP3P

EXTENDED		
acceptor	donor	occ%
**ILE3**	**VAL5**	7.07
GLN2	LYS6	6.25
**PHE4**	**LYS6**	5.83

MISFOLDED		
acceptor	donor	occ%
PHE4	LEU8	8.69
GLN2	LYS6	8.12
ILE3	THR7	8.02
VAL5	THR9	6.34
THR12	GLU16	5.88
ACE1	ILE3	5.16

EXTENDED		
acceptor	donor	occ%
GLN2	LYS6	22.07
ILE3	THR7	21.90
PHE4	LEU8	21.28
VAL5	THR9	17.91
THR12	GLU16	17.33
LYS6	GLY10	16.09
**THR7**	**LYS11**	13.77
THR9	ILE13	12.93
LYS11	LEU15	11.46
LEU8	THR12	10.18
GLY10	THR14	9.42
ILE3	LYS6	8.74
ACE1	PHE4	8.45
THR12	LEU15	7.05
ACE1	VAL5	6.97
GLN2	VAL5	6.50
LYS11	GLU16	5.73

MISFOLDED		
acceptor	donor	occ%
PHE4	LEU8	24.76
VAL5	THR9	22.40
GLN2	LYS6	19.08
LYS6	GLY10	18.29
ILE3	THR7	16.13
THR12	GLU16	15.92
**THR7**	**LYS11**	13.36
THR9	ILE13	11.74
LEU8	THR12	10.74
GLY10	THR14	8.63
LYS11	LEU15	8.39
THR12	LEU15	8.14
GLN2	VAL5	5.89
ACE1	ILE3	5.62
ILE3	LYS6	5.48
ACE1	PHE4	5.41
**ILE3**	**VAL5**	5.25
**LYS11**	**ILE13**	5.16

ff14SB OPC					
EXTENDED			MISFOLDED		
acceptor	donor	occ%	acceptor	donor	occ%
PHE4	LEU8	34.19	VAL5	THR9	14.89
VAL5	THR9	29.81	PHE4	LEU8	13.29
ILE3	THR7	27.15	ILE3	THR7	9.79
GLN2	LYS6	23.29	GLN2	LYS6	8.99
LYS6	GLY10	17.73	LYS6	GLY10	8.41
**THR7**	**LYS11**	12.25	**PHE4**	**LYS6**	6.13
THR9	ILE13	11.88	LEU8	THR12	6.02
LEU8	THR12	11.78	THR9	ILE13	5.94
GLY10	THR14	8.72	**THR7**	**LYS11**	5.93
THR12	GLU16	6.68	THR12	GLU16	5.52
ACE1	VAL5	5.89	GLY10	THR14	5.38
LYS11	LEU15	5.84			
GLN2	VAL5	5.31			

ff14SB TIP3P					
EXTENDED			MISFOLDED		
acceptor	donor	occ%	acceptor	donor	occ%
PHE4	LEU8	53.03	PHE4	LEU8	50.57
VAL5	THR9	51.95	VAL5	THR9	50.13
**THR7**	**LYS11**	44.33	LYS6	GLY10	38.09
GLN2	LYS6	43.51	GLN2	LYS6	36.04
THR9	ILE13	40.66	**THR7**	**LYS11**	35.90
ILE3	THR7	40.28	ILE3	THR7	35.51
LYS6	GLY10	37.95	THR9	ILE13	34.71
LEU8	THR12	35.48	LEU8	THR12	28.11
LYS11	LEU15	23.08	LYS11	LEU15	22.87
THR12	GLU16	20.48	THR12	GLU16	21.59
GLY10	THR14	19.79	GLY10	THR14	20.92
ACE1	VAL5	12.19	ACE1	VAL5	9.90
ACE1	PHE4	8.72	LYS11	GLU16	8.93
LYS11	GLU16	6.66	ACE1	PHE4	7.17
GLN2	VAL5	6.50	GLN2	VAL5	5.47
			THR12	LEU15	5.05

ff19SB OPC					
EXTENDED			MISFOLDED		
acceptor	donor	occ%	acceptor	donor	occ%
GLN2	LYS6	6.24	GLN2	LYS6	7.48
ACE1	PHE4	5.61	GLN2	VAL5	7.16
GLN2	VAL5	5.40			
**ILE13**	**VAL5**	5.24			

ff19SB TIP3P					
EXTENDED			MISFOLDED		
acceptor	donor	occ%	acceptor	donor	occ%
GLN2	LYS6	17.75	GLN2	LYS6	17.52
PHE4	LEU8	13.10	PHE4	LEU8	15.49
ILE3	THR7	12.21	ILE3	THR7	14.31
GLN2	VAL5	10.37	VAL5	THR9	11.04

**VAL5**	**ILE13**	5.10

VAL5	THR9	9.34
ACE1	PHE4	8.68
THR12	LEU15	8.61
THR12	GLU16	6.44

GLN2	VAL5	10.72
ACE1	PHE4	8.58
THR12	LEU15	6.64
PHE4	THR7	5.78
LYS6	GLY10	5.31
THR12	GLU16	5.10

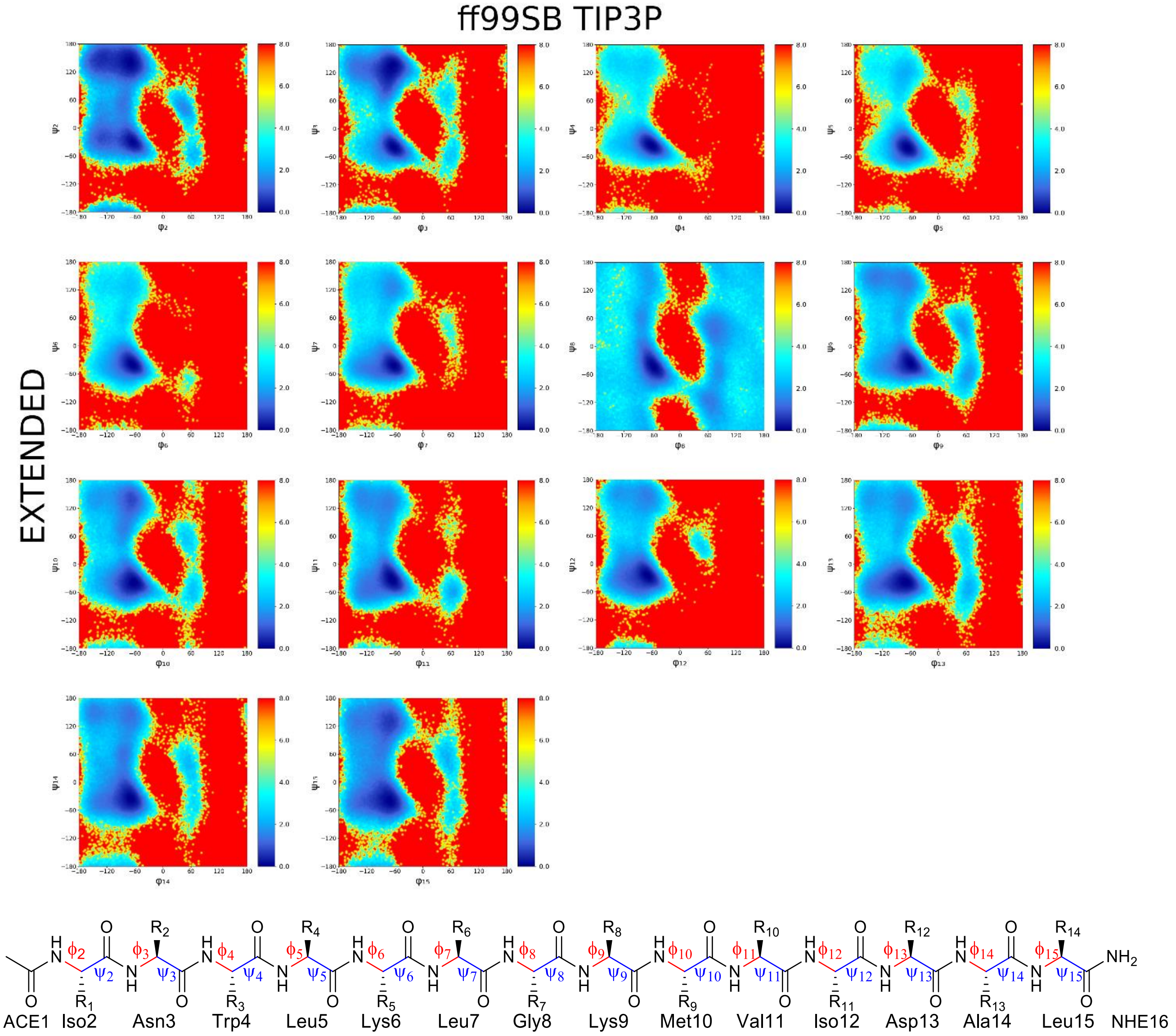

Figure S81. PMFs obtained from φ and ψ dihedral distributions of ID1 residues for the ff99SB/TIP3P combination from an extended conformation. PMFs are shown as heat maps (0.0 – 8.0 kcal/mol range) from dark blue to red. The native conformation's φ and ψ angles are reported as black diamonds. ID1 sequence and representation of φ and ψ angles numbering are also shown.

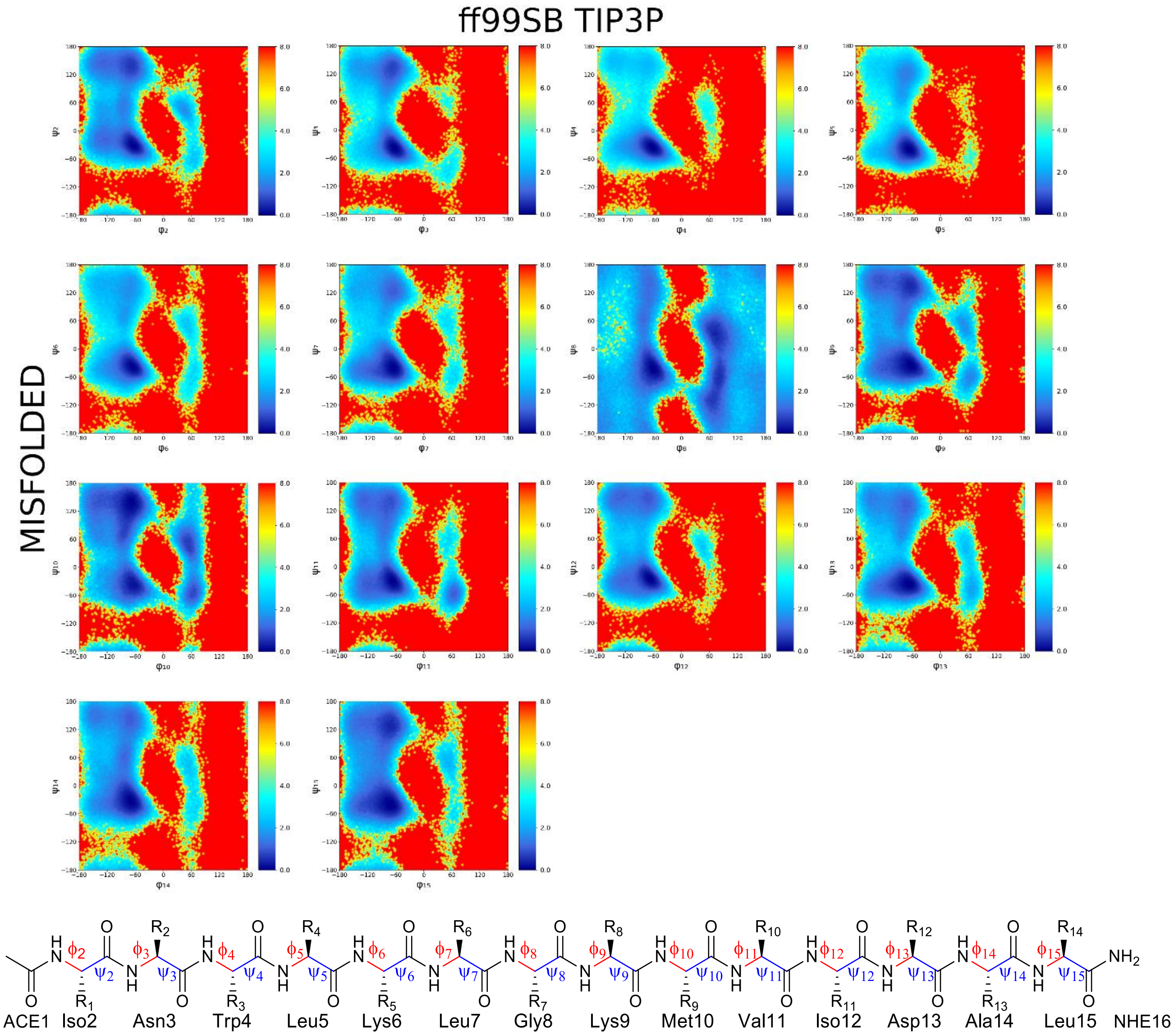

Figure S82. PMFs obtained from φ and ψ dihedral distributions of ID1 residues for the ff99SB/TIP3P combination from a misfolded conformation. PMFs are shown as heat maps (0.0 – 8.0 kcal/mol range) from dark blue to red. The native conformation's φ and ψ angles are reported as black diamonds. ID1 sequence and representation of φ and ψ angles numbering are also shown.

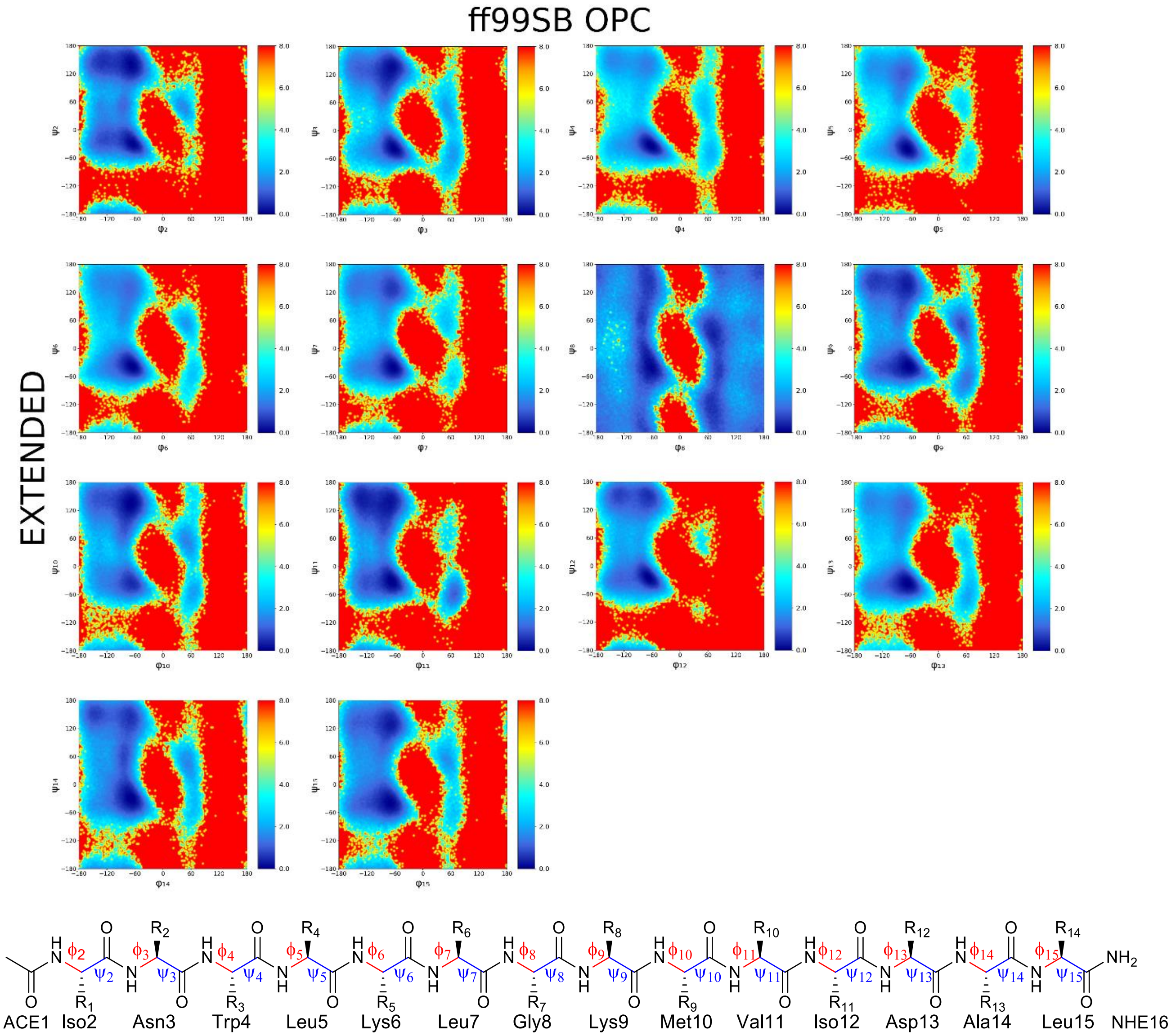

Figure S83. PMFs obtained from φ and ψ dihedral distributions of ID1 residues for the ff99SB/OPC combination from an extended conformation. PMFs are shown as heat maps (0.0 – 8.0 kcal/mol range) from dark blue to red. The native conformation's φ and ψ angles are reported as black diamonds. ID1 sequence and representation of φ and ψ angles numbering are also shown.

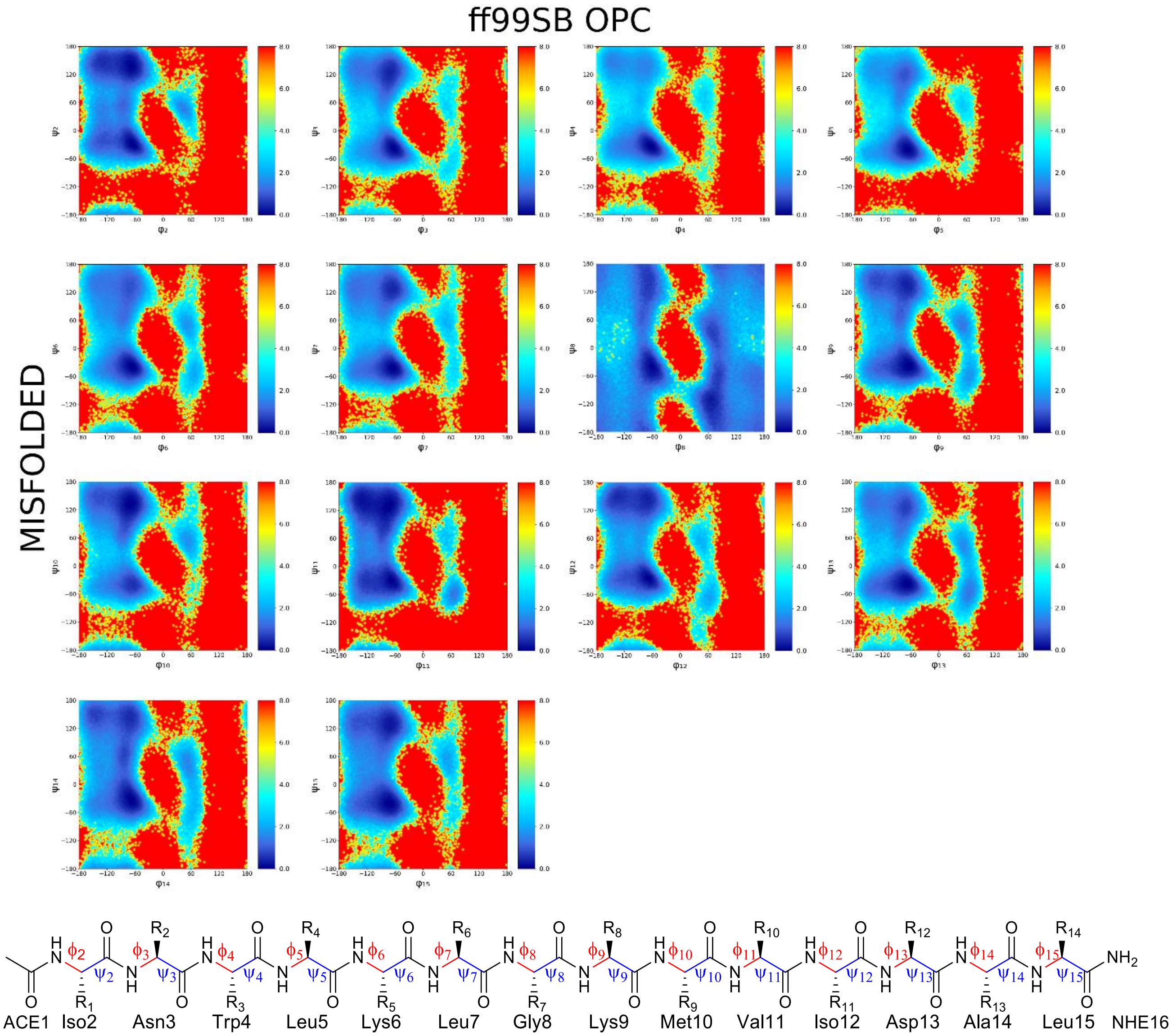

Figure S84. PMFs obtained from φ and ψ dihedral distributions of ID1 residues for the ff99SB/OPC combination from a misfolded conformation. PMFs are shown as heat maps (0.0 – 8.0 kcal/mol range) from dark blue to red. The native conformation's φ and ψ angles are reported as black diamonds. ID1 sequence and representation of φ and ψ angles numbering are also shown.

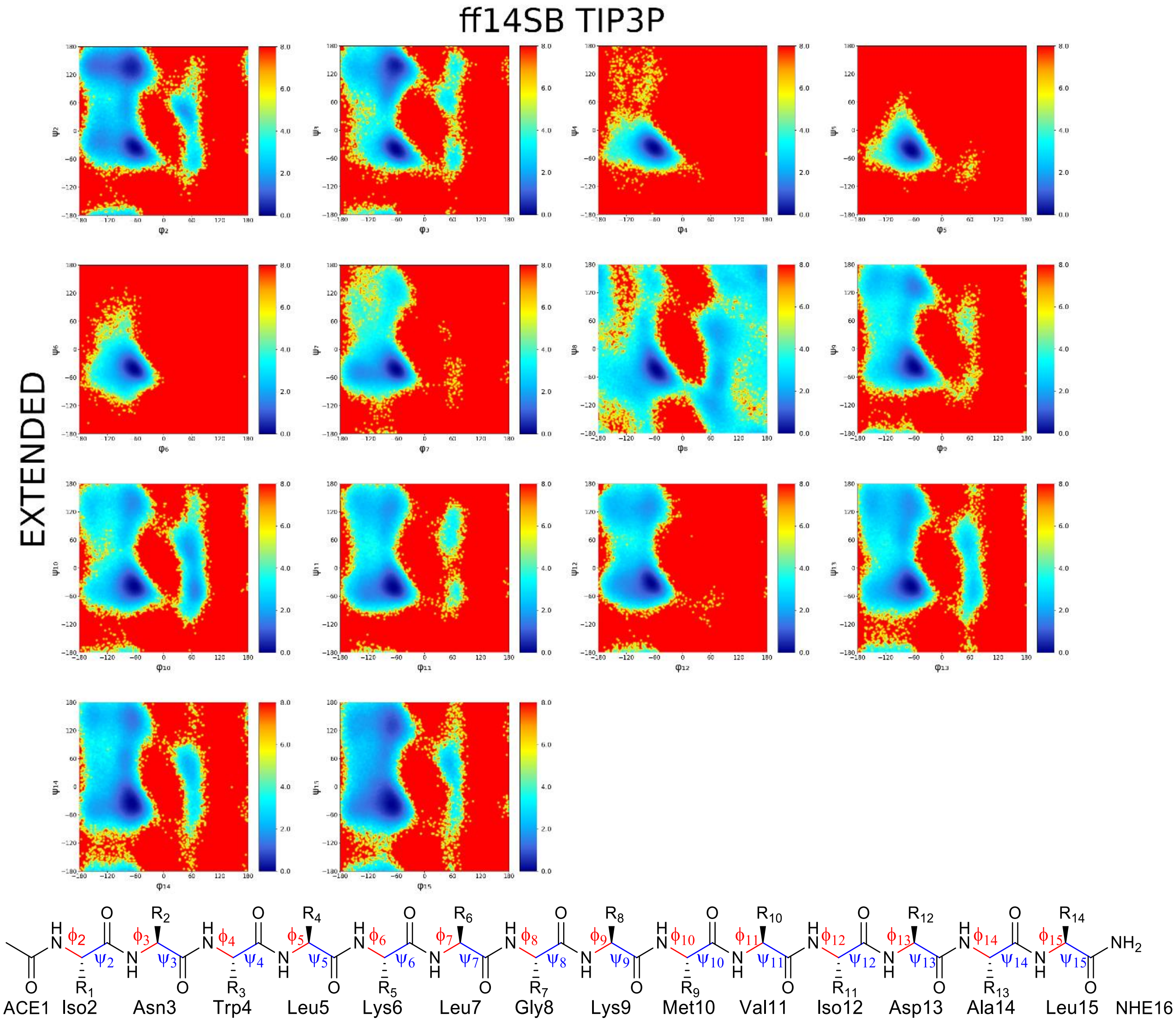

Figure S85. PMFs obtained from φ and ψ dihedral distributions of ID1 residues for the ff14SB/TIP3P combination from an extended conformation. PMFs are shown as heat maps (0.0 – 8.0 kcal/mol range) from dark blue to red. The native conformation's φ and ψ angles are reported as black diamonds. ID1 sequence and representation of φ and ψ angles numbering are also shown.

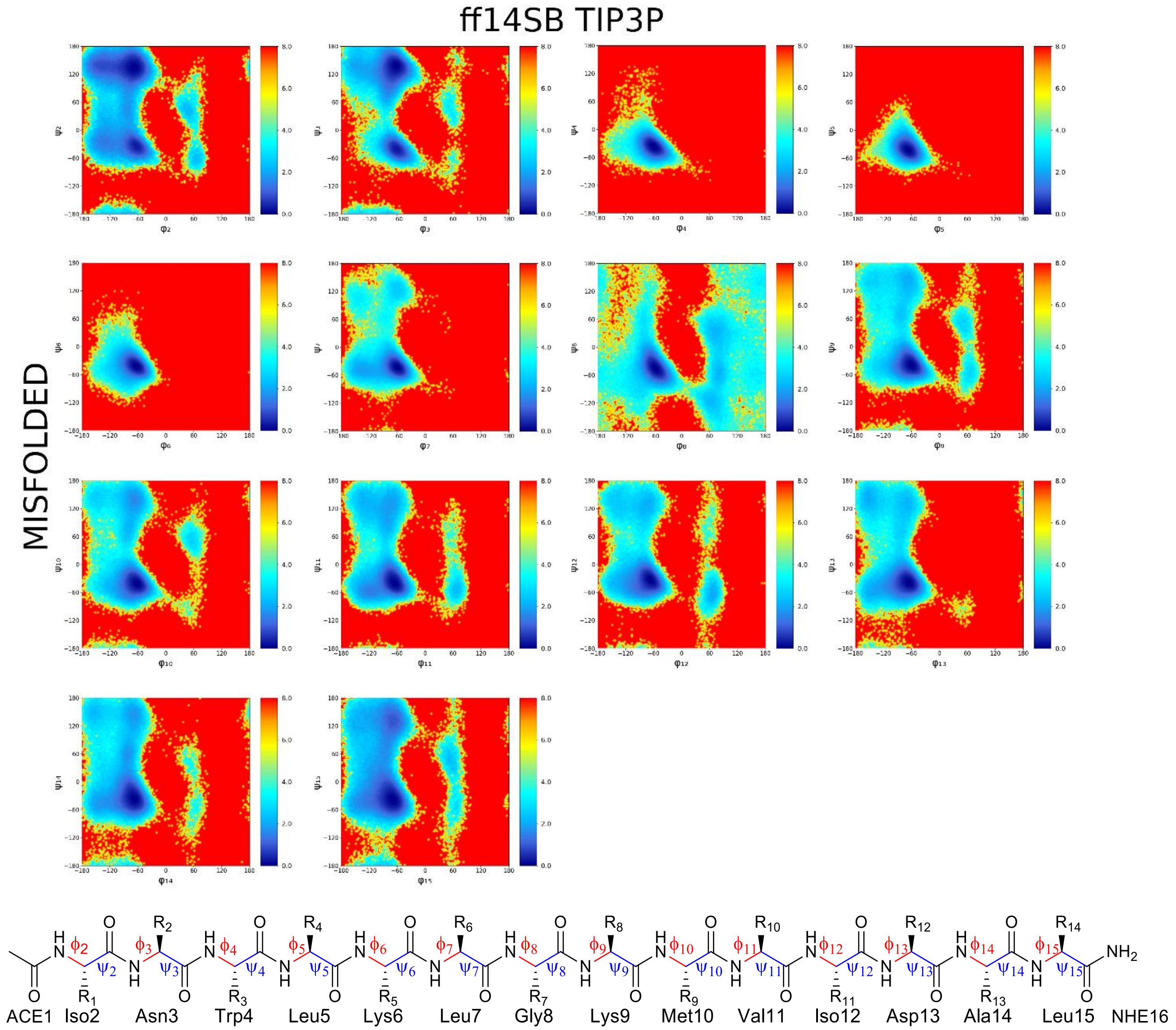

Figure S86. PMFs obtained from φ and ψ dihedral distributions of ID1 residues for the ff14SB/TIP3P combination from a misfolded conformation. PMFs are shown as heat maps (0.0 – 8.0 kcal/mol range) from dark blue to red. The native conformation's φ and ψ angles are reported as black diamonds. ID1 sequence and representation of φ and ψ angles numbering are also shown.

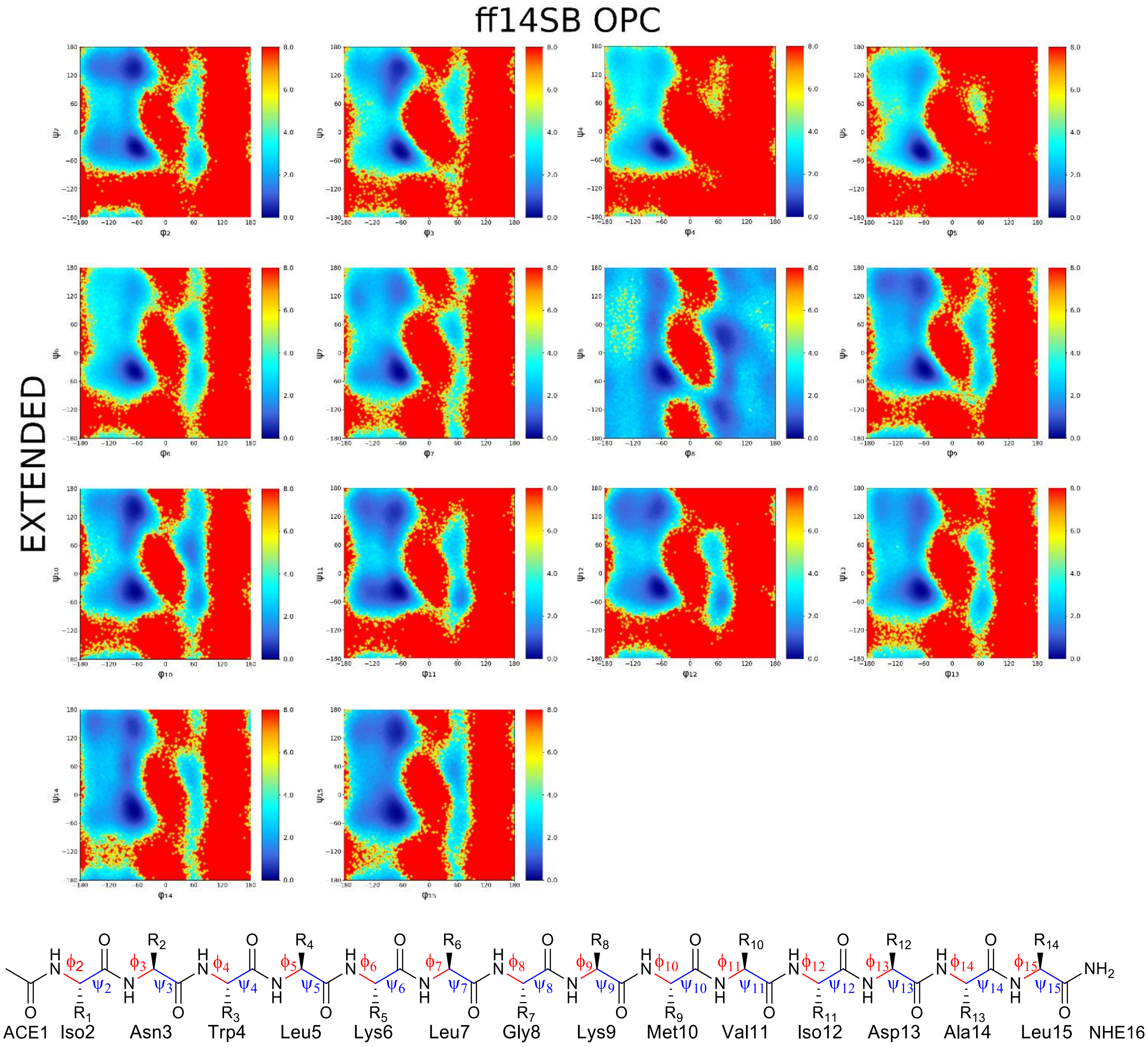

Figure S87. PMFs obtained from φ and ψ dihedral distributions of ID1 residues for the ff14SB/OPC combination from an extended conformation. PMFs are shown as heat maps (0.0 – 8.0 kcal/mol range) from dark blue to red. The native conformation's φ and ψ angles are reported as black diamonds. ID1 sequence and representation of φ and ψ angles numbering are also shown.

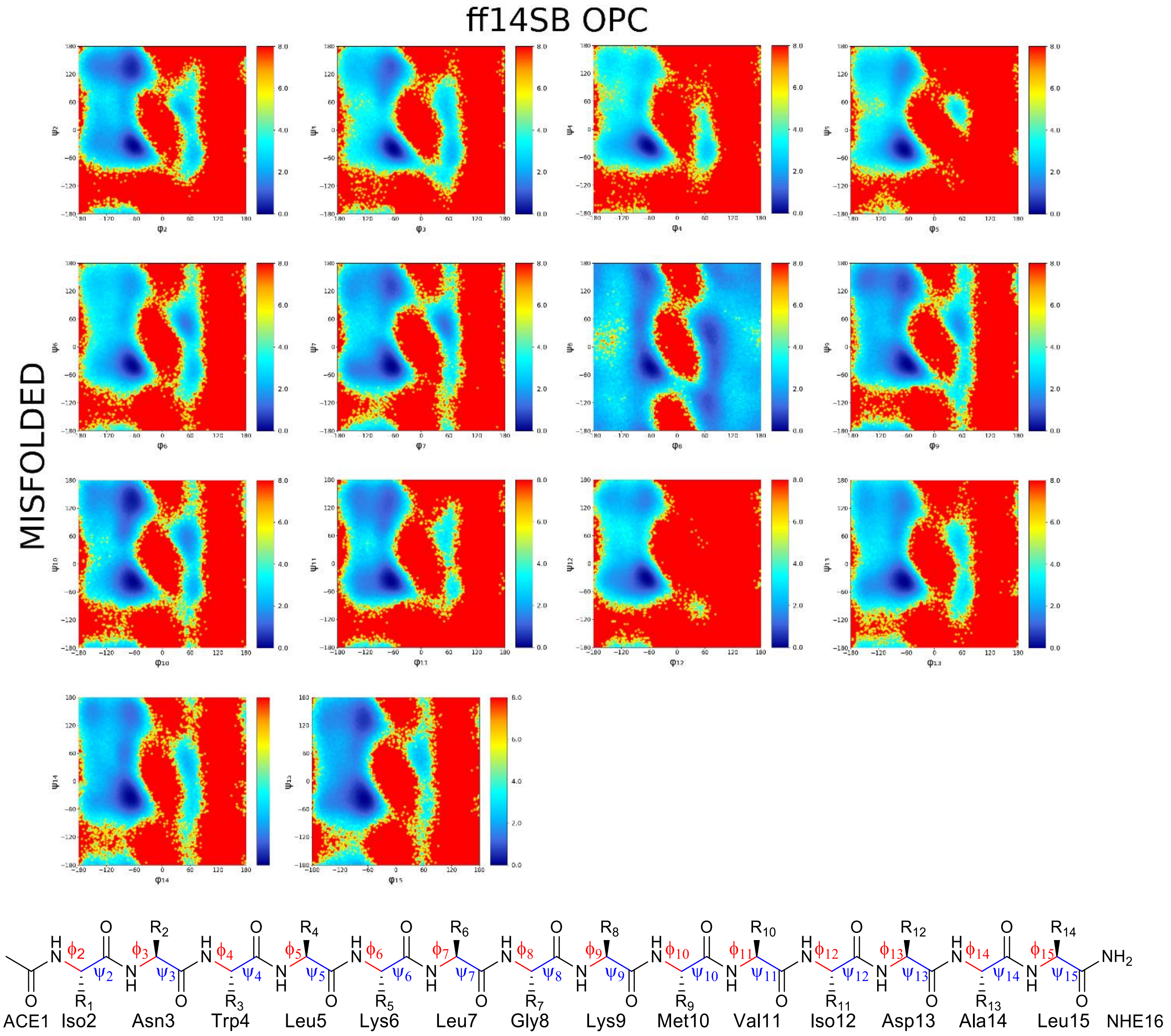

Figure S88. PMFs obtained from φ and ψ dihedral distributions of ID1 residues for the ff14SB/OPC combination from a misfolded conformation. PMFs are shown as heat maps (0.0 – 8.0 kcal/mol range) from dark blue to red. The native conformation's φ and ψ angles are reported as black diamonds. ID1 sequence and representation of φ and ψ angles numbering are also shown.

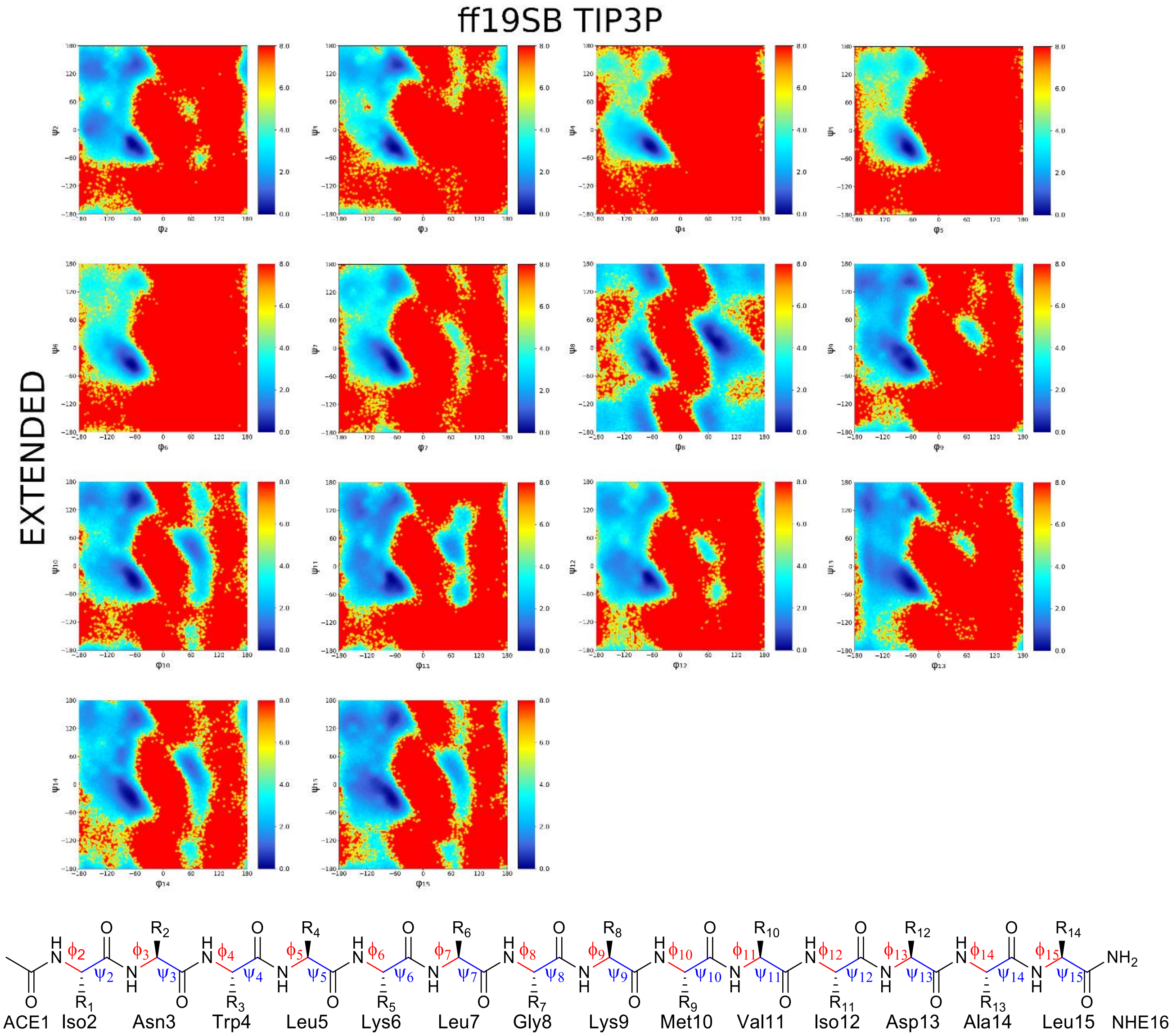

Figure S89. PMFs obtained from φ and ψ dihedral distributions of ID1 residues for the ff19SB/TIP3P combination from an extended conformation. PMFs are shown as heat maps (0.0 – 8.0 kcal/mol range) from dark blue to red. The native conformation's φ and ψ angles are reported as black diamonds. ID1 sequence and representation of φ and ψ angles numbering are also shown.

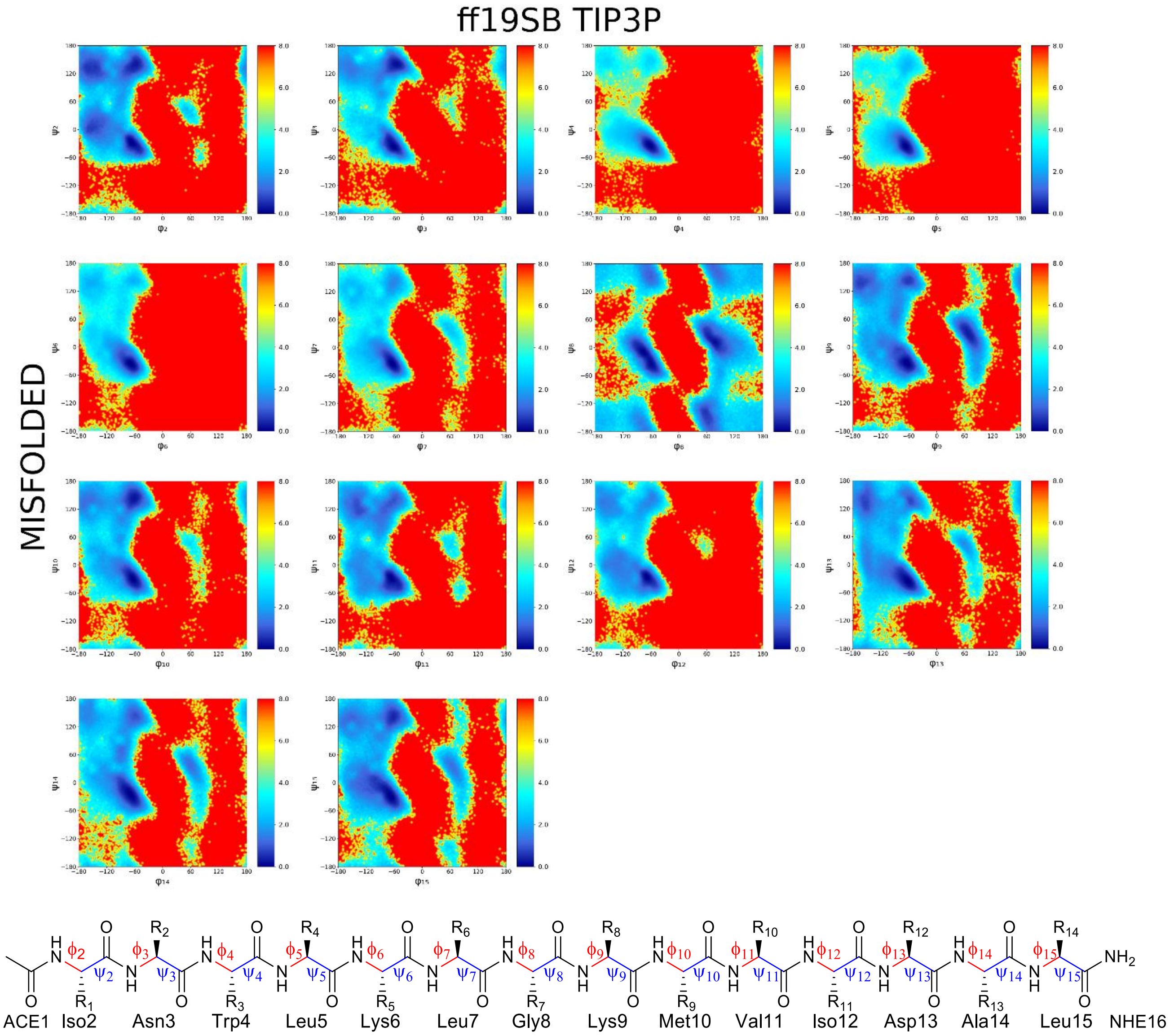

Figure S90. PMFs obtained from φ and ψ dihedral distributions of ID1 residues for the ff19SB/TIP3P combination from a misfolded conformation. PMFs are shown as heat maps (0.0 – 8.0 kcal/mol range) from dark blue to red. The native conformation's φ and ψ angles are reported as black diamonds. ID1 sequence and representation of φ and ψ angles numbering are also shown.

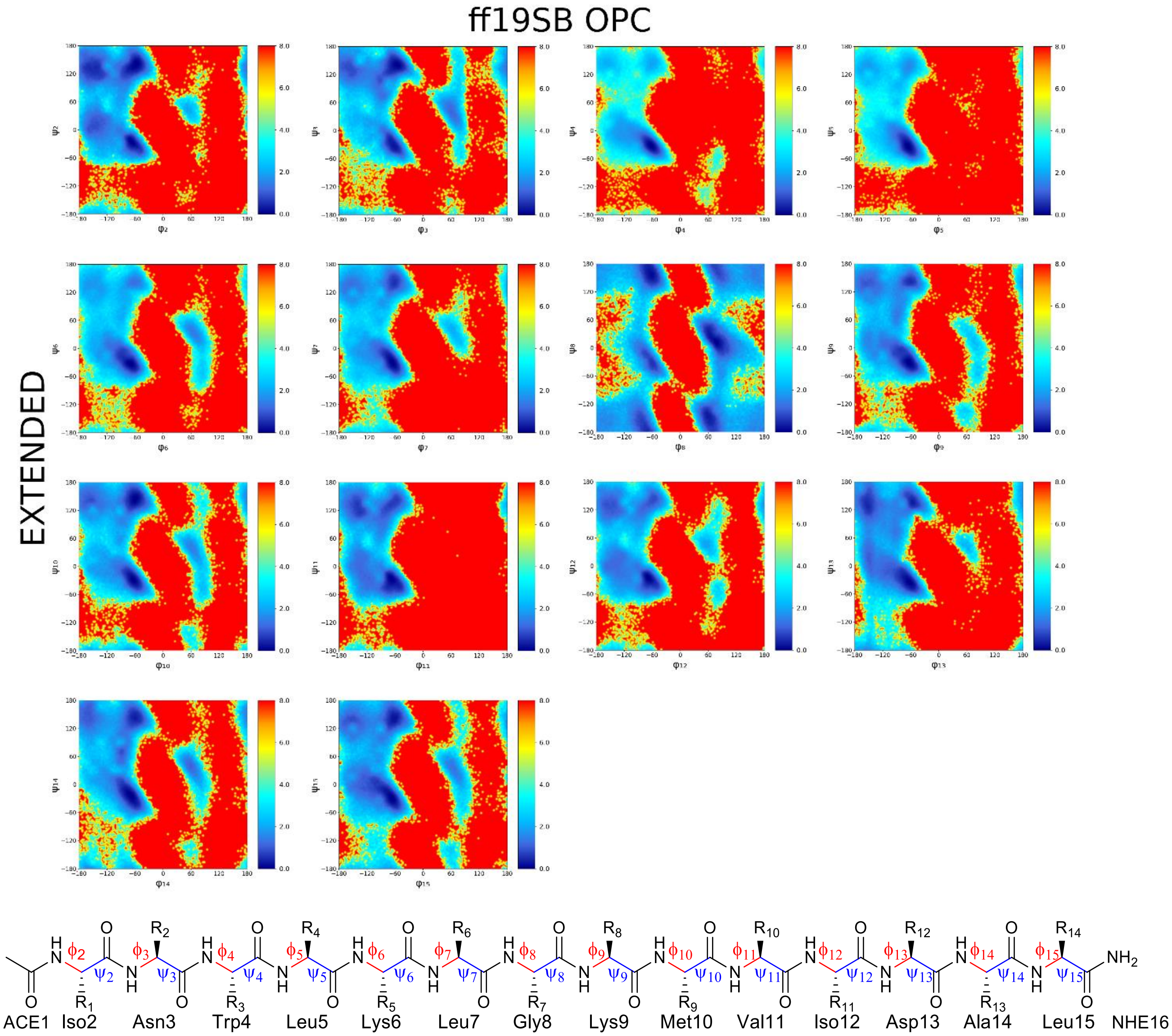

Figure S91. PMFs obtained from φ and ψ dihedral distributions of ID1 residues for the ff19SB/OPC combination from an extended conformation. PMFs are shown as heat maps (0.0 – 8.0 kcal/mol range) from dark blue to red. The native conformation's φ and ψ angles are reported as black diamonds. ID1 sequence and representation of φ and ψ angles numbering are also shown.

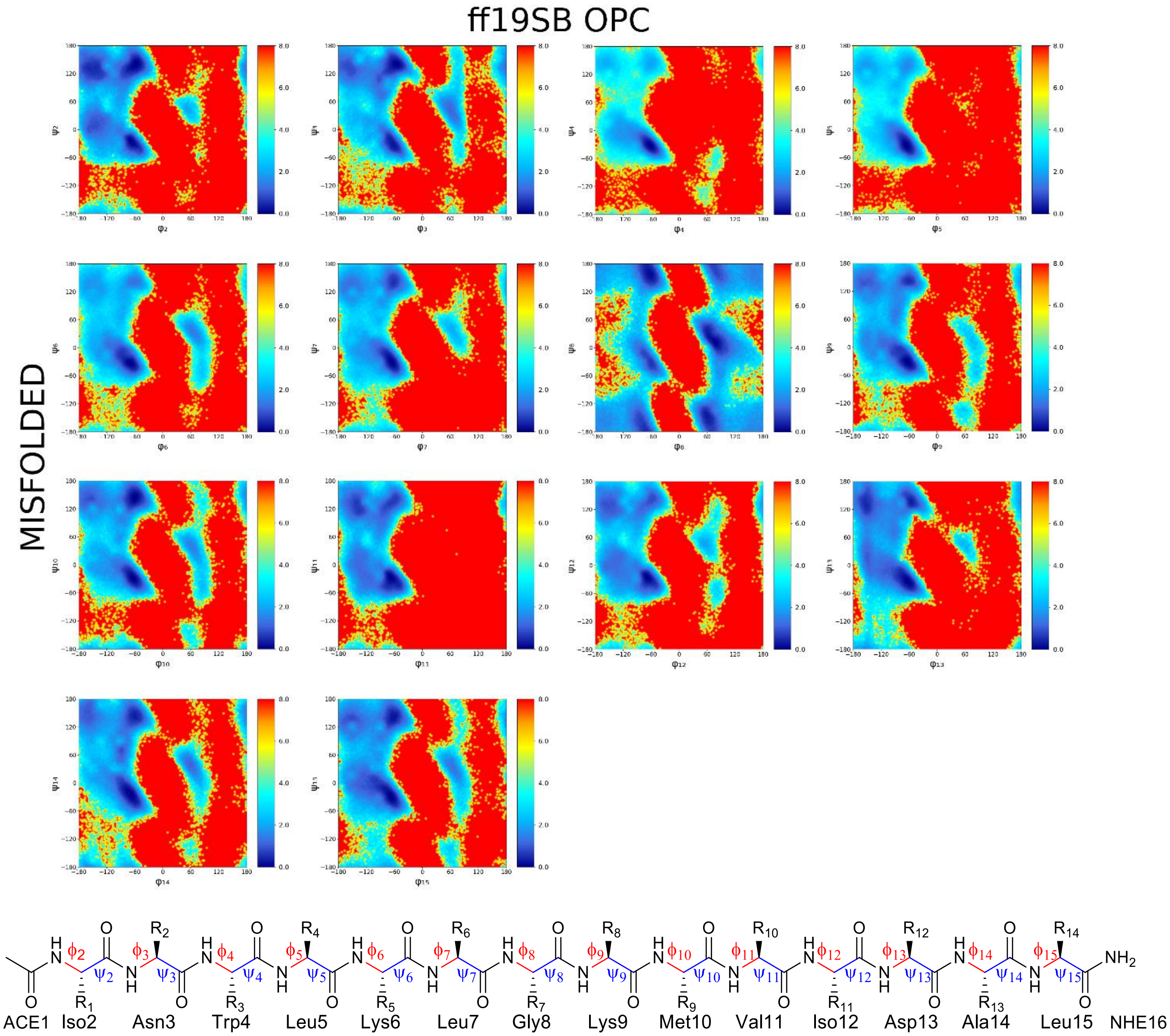

Figure S92. PMFs obtained from φ and ψ dihedral distributions of ID1 residues for the ff19SB/OPC combination from a misfolded conformation. PMFs are shown as heat maps (0.0 – 8.0 kcal/mol range) from dark blue to red. The native conformation's φ and ψ angles are reported as black diamonds. ID1 sequence and representation of φ and ψ angles numbering are also shown.

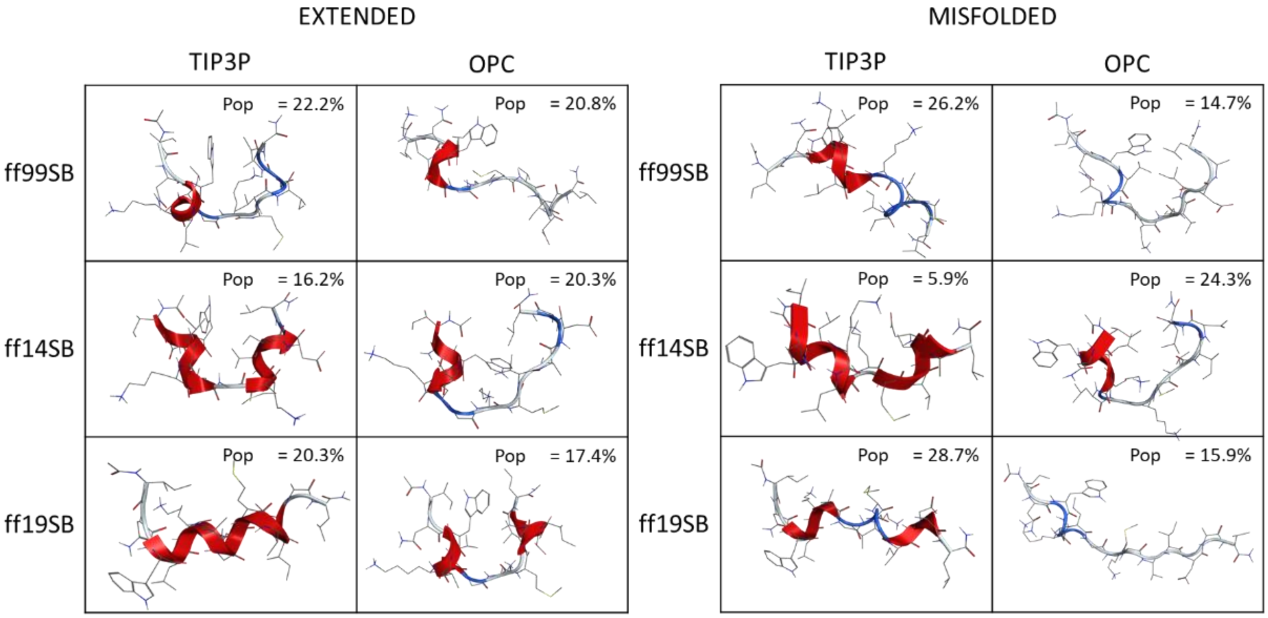

Figure S93. Representative conformation and population (pop%) of the second clusters from ID1 trajectories.

Table S9. H-bond analyses from ID1 trajectories. Only H-bonds involving the backbone are included in the table (donor N-H, acceptor C=O). Native H-bonds are highlighted in bold.

ff99SB OPC

EXTENDED			MISFOLDED		
acceptor	donor	occ%	acceptor	donor	occ%
ASN3	LEU7	19.47	ASN3	LEU7	15.24
TRP4	GLY8	13.95	TRP4	GLY8	10.11
VAL11	LEU15	6.93	ILE2	LYS6	7.64
ILE2	LYS6	6.57			

ff99SB TIP3P

EXTENDED			MISFOLDED		
acceptor	donor	occ%	acceptor	donor	occ%
ASN3	LEU7	34.89	ASN3	LEU7	28.27
TRP4	GLY8	28.97	TRP4	GLY8	17.39
MET10	ALA14	15.99	ILE2	LYS6	17.07
LEU7	VAL11	14.86	TRP4	LYS9	12.50
VAL11	LEU15	14.50	VAL11	LEU15	11.37
LYS9	ASP13	12.16	ACE1	LEU5	10.64
LEU5	LYS9	12.14	MET10	ALA14	8.04
TRP4	LYS9	11.69	ILE2	LEU5	7.02
LYS6	MET10	10.72	GLY8	ILE12	6.15
GLY8	ILE12	9.99	LEU7	LYS9	5.98
ILE2	LYS6	9.10	ASN3	GLY8	5.86
MET10	ASP13	7.25	LYS9	VAL11	5.53
ASN3	GLY8	6.49	MET10	ASP13	5.38
MET10	LEU15	6.36	MET10	ILE12	5.19
ASN3	LYS6	5.88	VAL11	ALA14	5.19
LEU7	MET10	5.81			

ff14SB OPC

EXTENDED			MISFOLDED		
acceptor	donor	occ%	acceptor	donor	occ%

ff14SB TIP3P

EXTENDED			MISFOLDED		
acceptor	donor	occ%	acceptor	donor	occ%

ASN3	LEU7	29.46
TRP4	GLY8	19.73
ILE2	LYS6	13.91
TRP4	LYS9	10.69
GLY8	ILE12	10.11
VAL11	LEU15	8.97
ACE1	LEU5	8.90
MET10	ALA14	7.05
GLY8	VAL11	6.12
ASN3	LYS6	5.58
LEU7	VAL11	5.52
LYS9	ASP13	5.47

ASN3	LEU7	27.03
ILE2	LYS6	20.96
TRP4	GLY8	16.92
VAL11	LEU15	14.70
MET10	ALA14	11.98
ACE1	LEU5	11.43
LYS9	ASP13	9.76
GLY8	ILE12	8.38
TRP4	LYS9	8.25
ILE2	LEU5	6.61
LEU7	VAL11	5.78
VAL11	ALA14	5.75
LEU5	LYS9	5.56
GLY8	VAL11	5.11
MET10	ASP13	5.04

ASN3	LEU7	44.94
TRP4	GLY8	34.08
LEU7	VAL11	21.72
GLY8	ILE12	20.38
LEU5	LYS9	20.37
VAL11	LEU15	19.04
ILE2	LYS6	18.77
LYS6	MET10	18.20
LYS9	ASP13	18.05
MET10	ALA14	15.95
TRP4	LYS9	12.66
ACE1	LEU5	10.67
GLY8	VAL11	7.00
LEU7	MET10	6.15
GLY8	ASP13	5.71

TRP5	TYR9	49.83
TYR9	TYR13	40.46
GLU7	LEU11	39.56
LEU8	LYS12	37.93
THR4	LEU8	34.15
GLN6	GLN10	33.25
GLN10	LYS14	26.96
LEU3	GLU7	19.75
LYS2	GLN6	15.38
LEU11	GLY15	10.46
ACE1	TRP5	10.39
GLN10	GLY15	7.91
THR4	GLU7	7.42
TYR9	LYS14	6.00

ff19SB OPC					
EXTENDED			MISFOLDED		
acceptor	donor	occ%	acceptor	donor	occ%
ASN3	LEU7	18.17	LEU8	LYS12	34.48
ASN3	LYS6	11.41	TRP5	TYR9	32.19
TRP4	GLY8	10.93	TYR9	LYS14	26.75
TRP4	LYS9	6.03	LEU3	GLU7	25.95
GLY8	VAL11	5.81	TYR9	TYR13	25.33
LYS9	ASP13	5.09	GLU7	LEU11	24.86
			GLN6	GLN10	23.14
			THR4	LEU8	22.81
			TRP5	GLN10	17.07
			LEU8	TYR13	16.75
			LYS2	GLN6	15.89
			GLN10	GLY15	15.70
			ACE1	TRP5	14.54
			GLN10	LYS14	13.21
			GLU7	LYS12	11.39
			GLN6	LEU11	11.02
			LEU3	LEU8	10.15
			ACE1	GLN6	8.00
			LEU11	ILE16	7.72
			THR4	TYR9	7.66
			LEU11	GLY15	7.02
			GLN10	ILE16	5.63
			LEU3	GLN6	5.45

ff19SB TIP3P					
EXTENDED			MISFOLDED		
acceptor	donor	occ%	acceptor	donor	occ%
ASN3	LEU7	35.90	GLU7	LEU11	39.46
TRP4	GLY8	20.21	TRP5	TYR9	37.55
ILE2	LYS6	19.84	LEU8	LYS12	30.56
TRP4	LYS9	14.58	TYR9	TYR13	27.23
ACE1	LEU5	12.13	THR4	LEU8	27.00
LEU5	GLY8	9.42	GLN10	LYS14	21.80
VAL11	LEU15	9.06	GLN6	GLN10	20.30
ASN3	LYS6	8.35	GLU7	GLN10	9.53
LYS9	ASP13	7.68	LEU11	GLY15	9.03
MET10	ALA14	7.08	THR4	GLU7	8.81
LEU5	LYS9	6.80	TYR9	LYS12	6.21
VAL11	ALA14	6.30	LYS2	GLN6	5.35
ACE1	TRP4	6.20			
GLY8	VAL11	6.13			
MET10	ASP13	6.07			
LYS6	MET10	5.88			
ILE2	LEU5	5.67			
GLY8	ILE12	5.06			

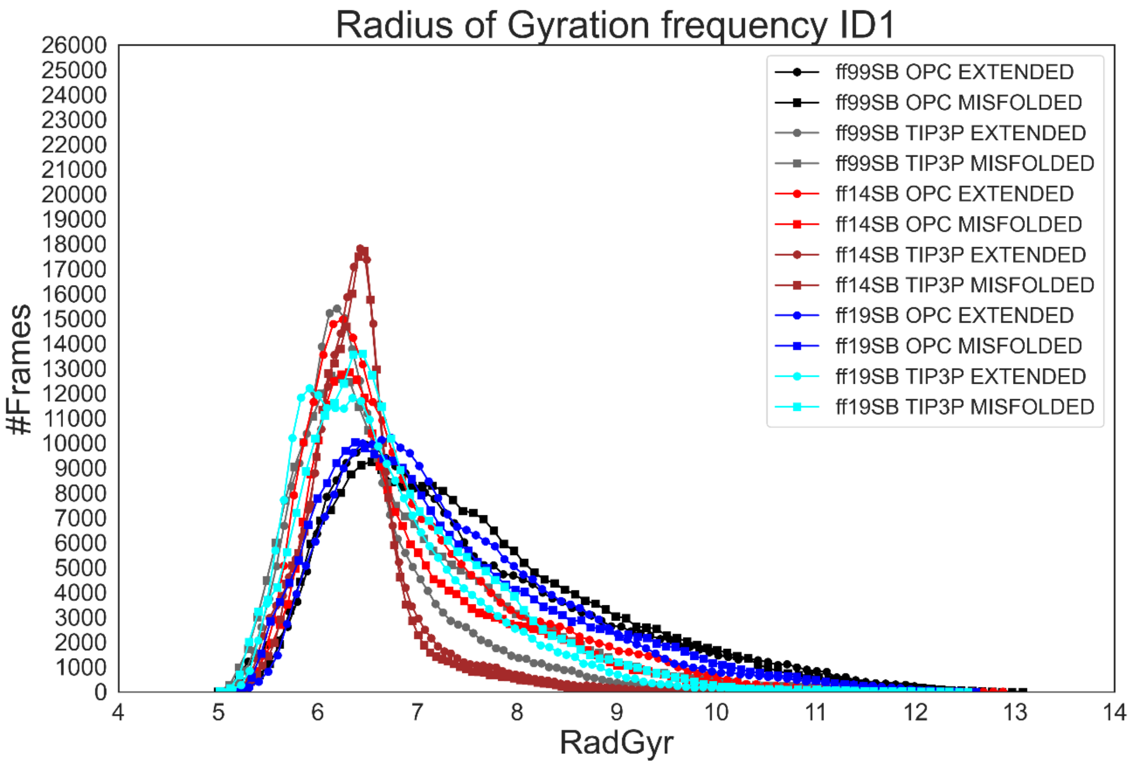

Figure S94. Radius of gyration frequencies calculated on the last 500ns of ID1 trajectories.

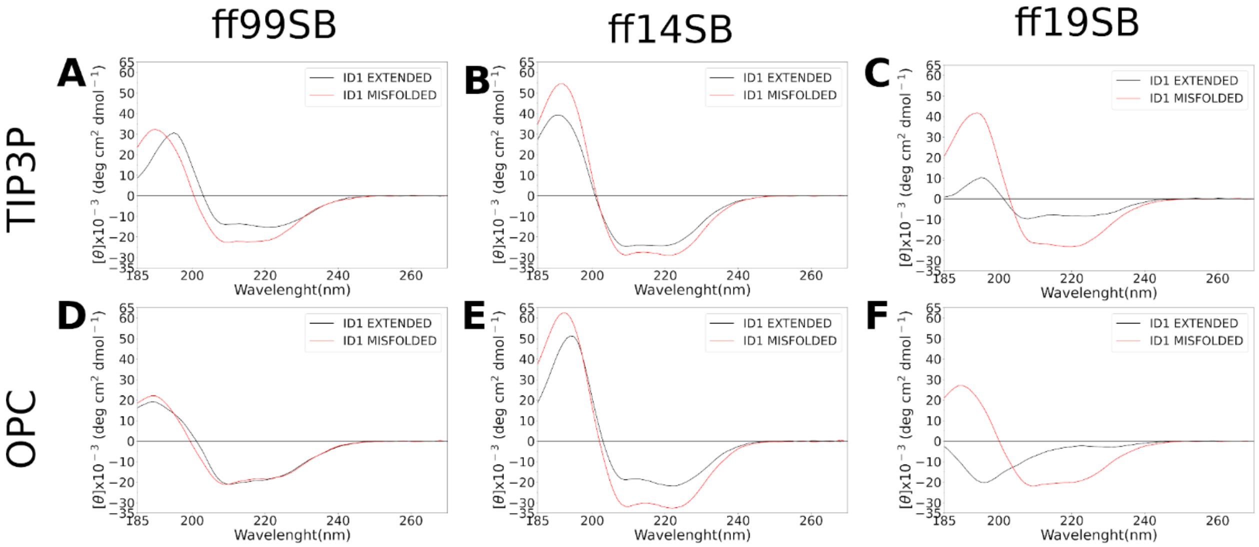

Figure S95. SESCA CD spectra of ID1 calculated on the main cluster obtained from the last 500 ns of aMD trajectory. Results from extended and misfolded simulations are reported in black and red lines, respectively.

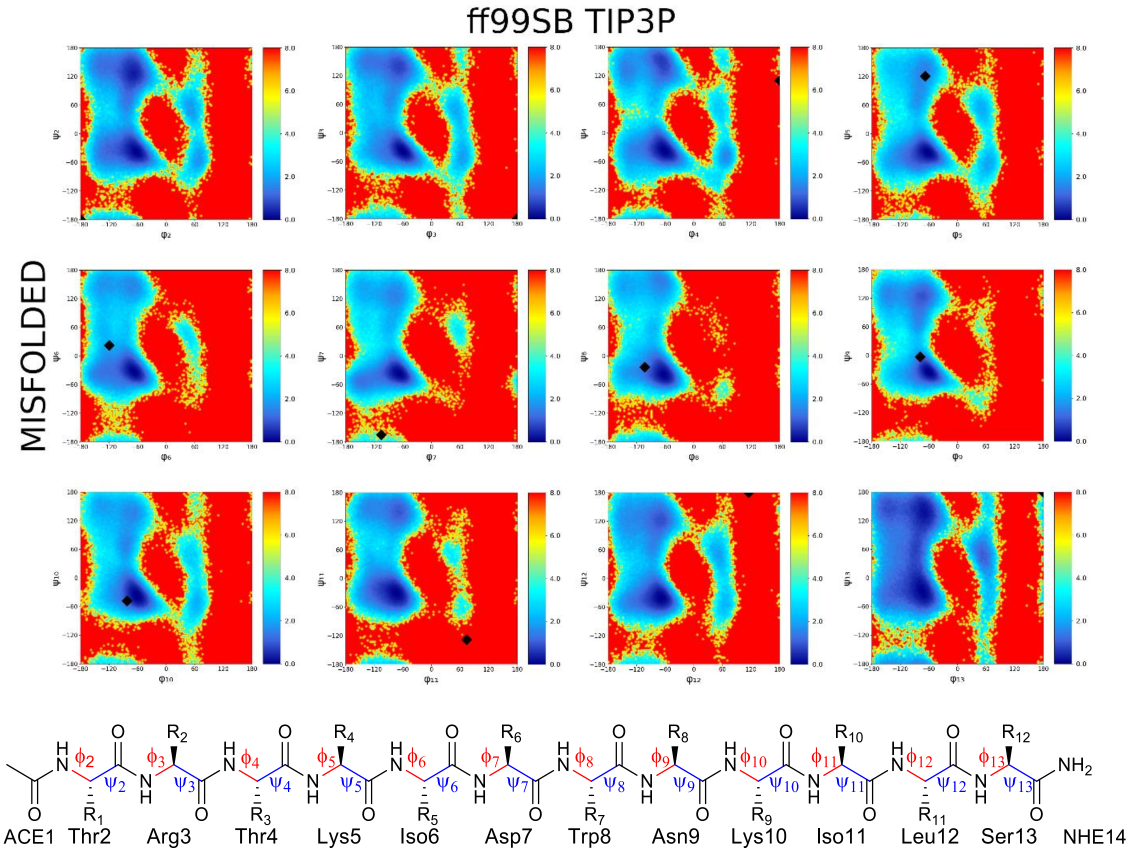

Figure S96. PMFs obtained from φ and ψ dihedral distributions of ID2 residues for the ff99SB/TIP3P combination from extended (top panels) and misfolded (bottom panels) conformations. PMFs are shown as heat maps (0.0 – 8.0 kcal/mol range) from dark blue to red. The native conformation's φ and ψ angles are reported as black diamonds. ID2 sequence and representation of φ and ψ angles numbering are also shown.

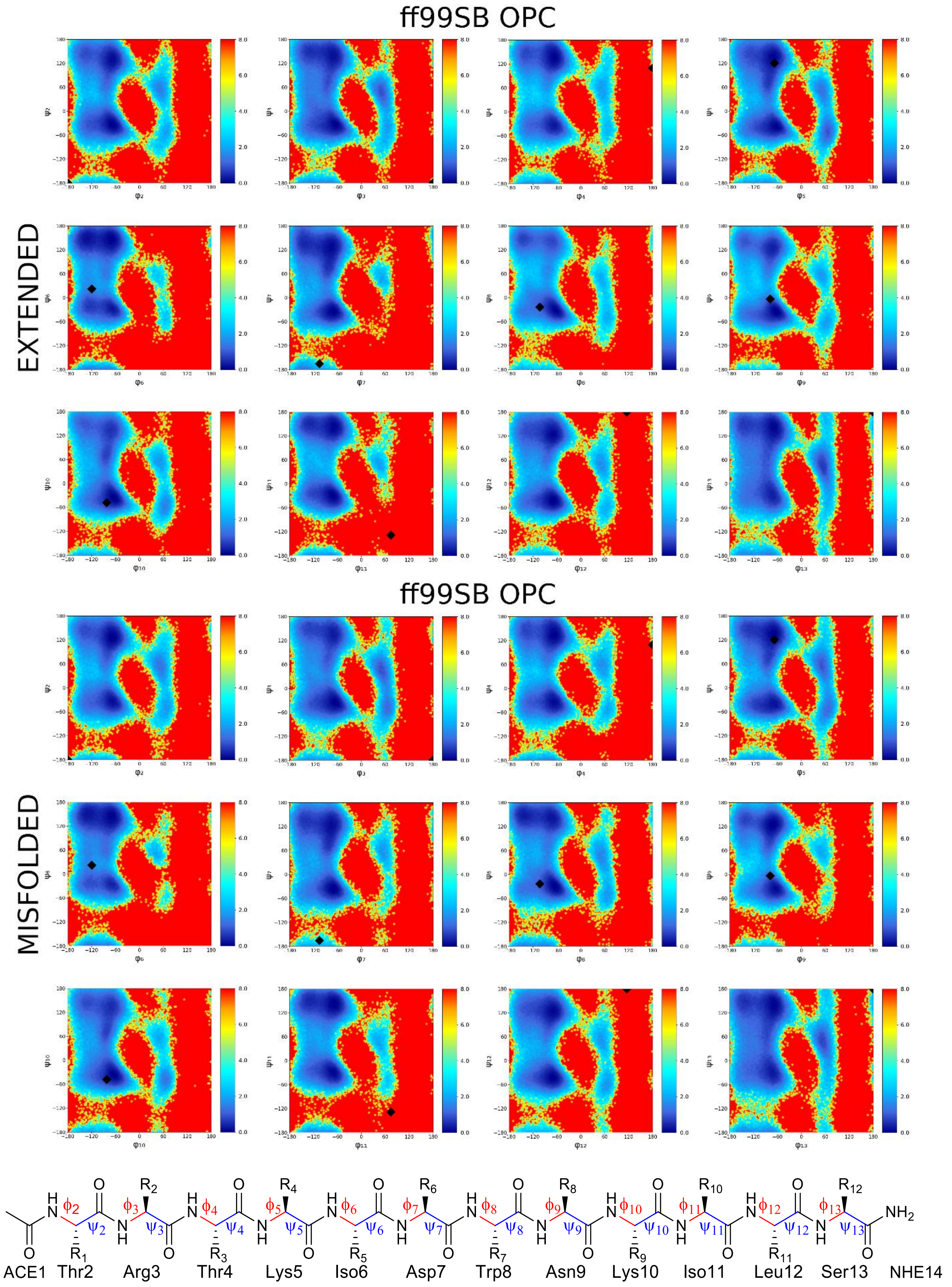

Figure S97. PMFs obtained from φ and ψ dihedral distributions of ID2 residues for the ff99SB/OPC combination from extended (top panels) and misfolded (bottom panels) conformations. PMFs are shown as heat maps (0.0 – 8.0 kcal/mol range) from dark blue to red. The native conformation's φ and ψ angles are reported as black diamonds. ID2 sequence and representation of φ and ψ angles numbering are also shown.

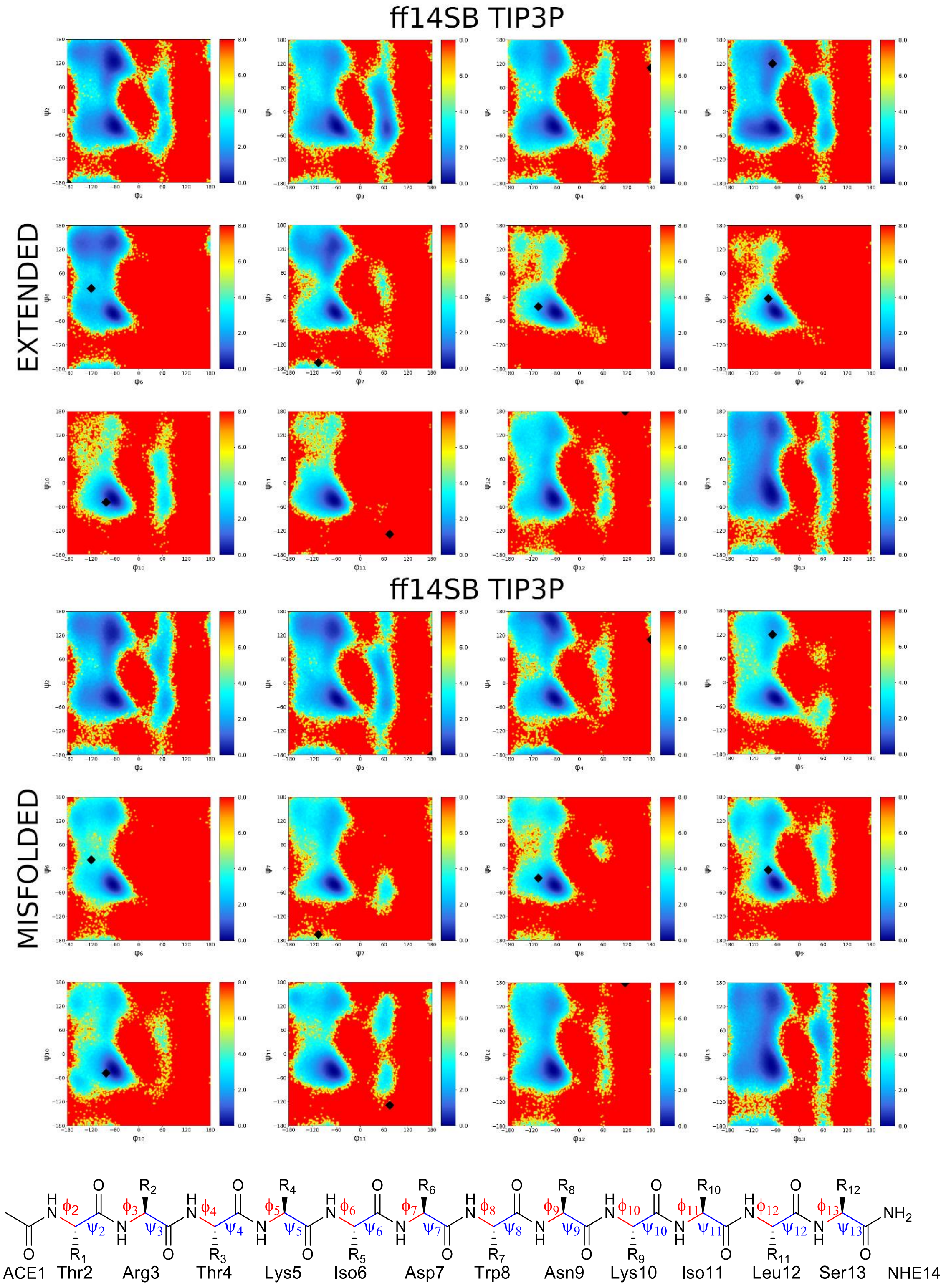

Figure S98. PMFs obtained from φ and ψ dihedral distributions of ID2 residues for the ff14SB/TIP3P combination from extended (top panels) and misfolded (bottom panels) conformations. PMFs are shown as heat maps (0.0 – 8.0 kcal/mol range) from dark blue to red. The native conformation's φ and ψ angles are reported as black diamonds. ID2 sequence and representation of φ and ψ angles numbering are also shown.

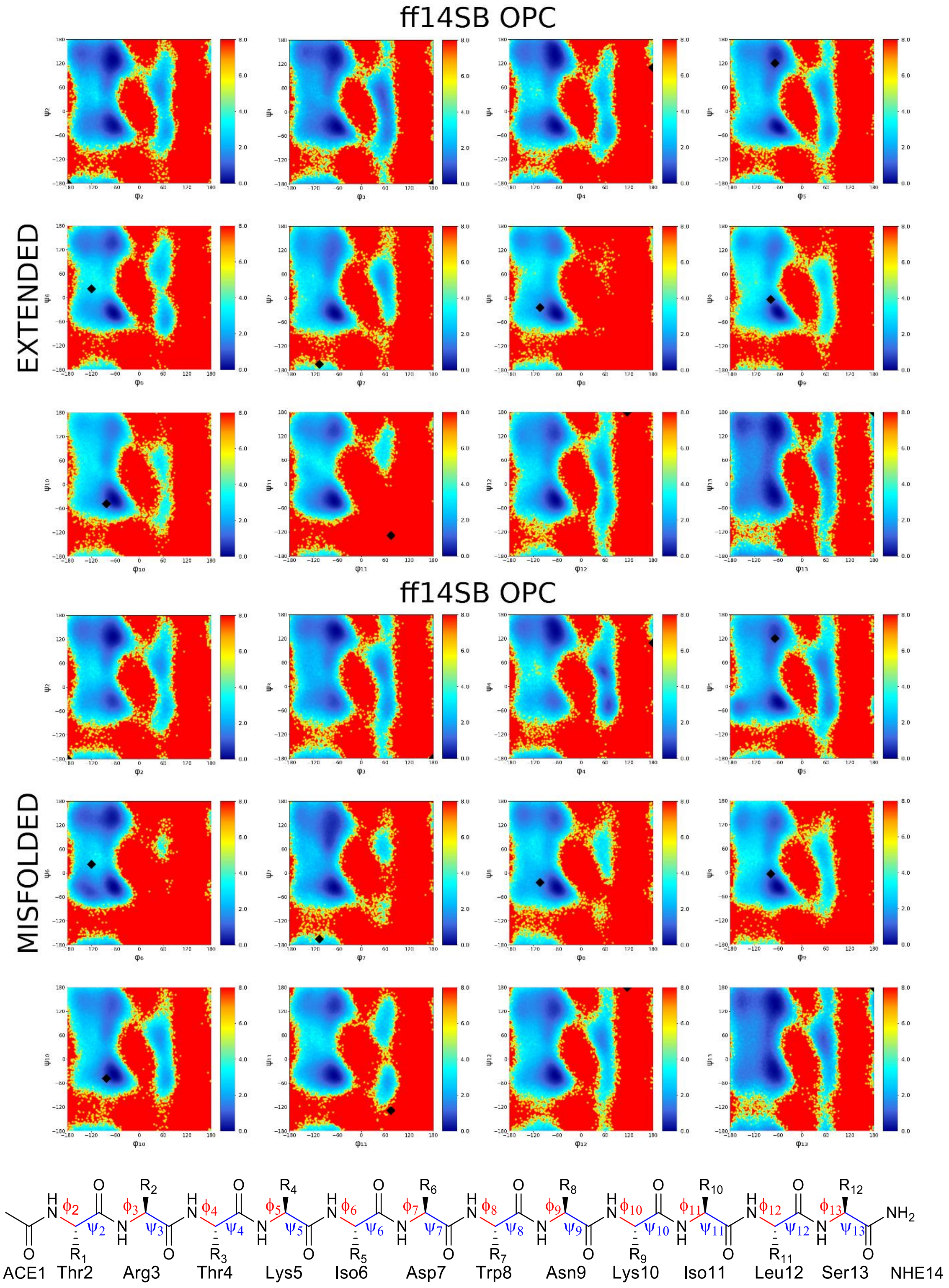

Figure S99. PMFs obtained from φ and ψ dihedral distributions of ID2 residues for the ff14SB/OPC combination from extended (top panels) and misfolded (bottom panels) conformations. PMFs are shown as heat maps (0.0 – 8.0 kcal/mol range) from dark blue to red. The native conformation's φ and ψ angles are reported as black diamonds. ID2 sequence and representation of φ and ψ angles numbering are also shown.

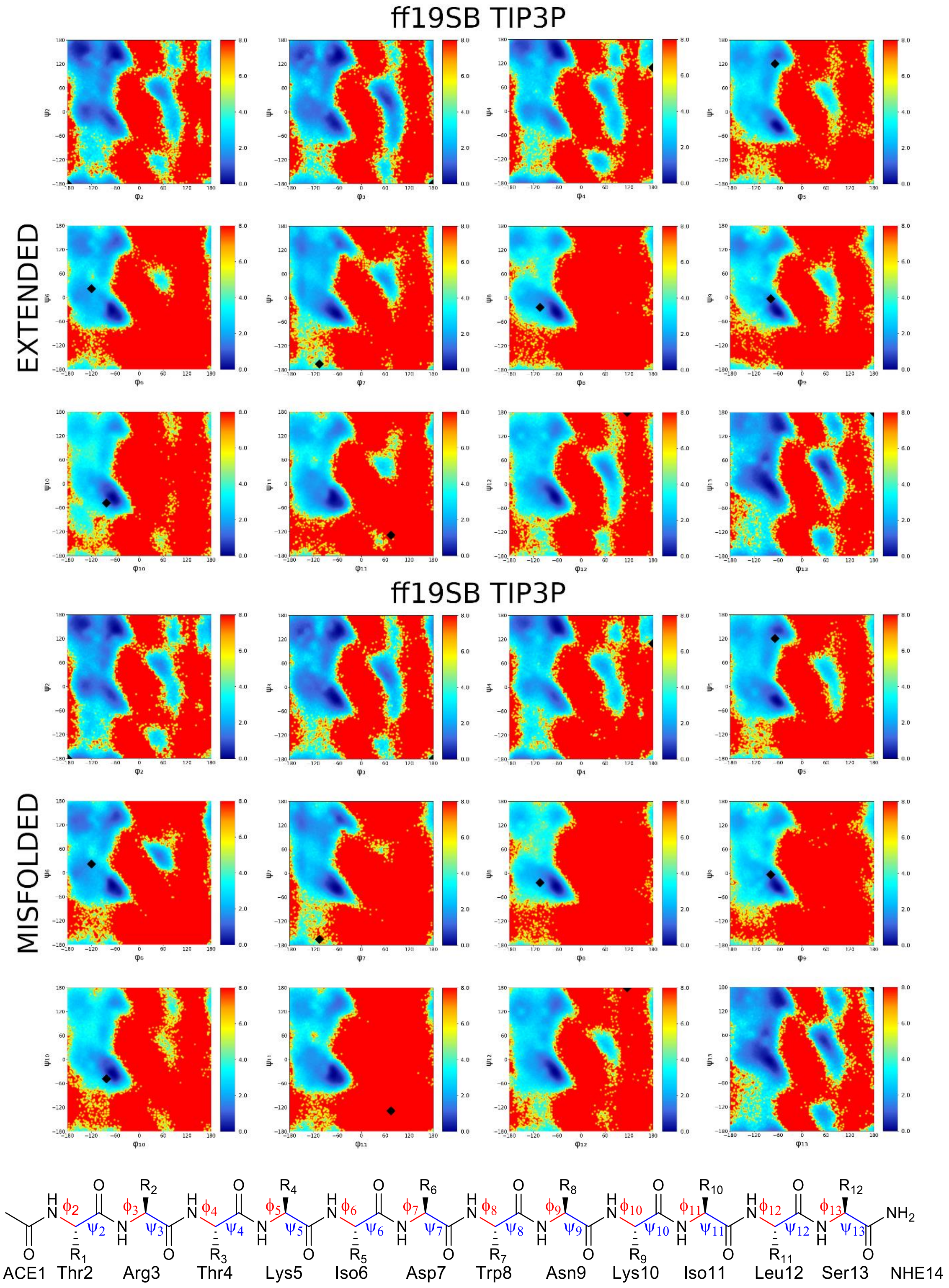

Figure S100. PMFs obtained from φ and ψ dihedral distributions of ID2 residues for the ff19SB/TIP3P combination from extended (top panels) and misfolded (bottom panels) conformations. PMFs are shown as heat maps (0.0 – 8.0 kcal/mol range) from dark blue to red. The native conformation's φ and ψ angles are reported as black diamonds. ID2 sequence and representation of φ and ψ angles numbering are also shown.

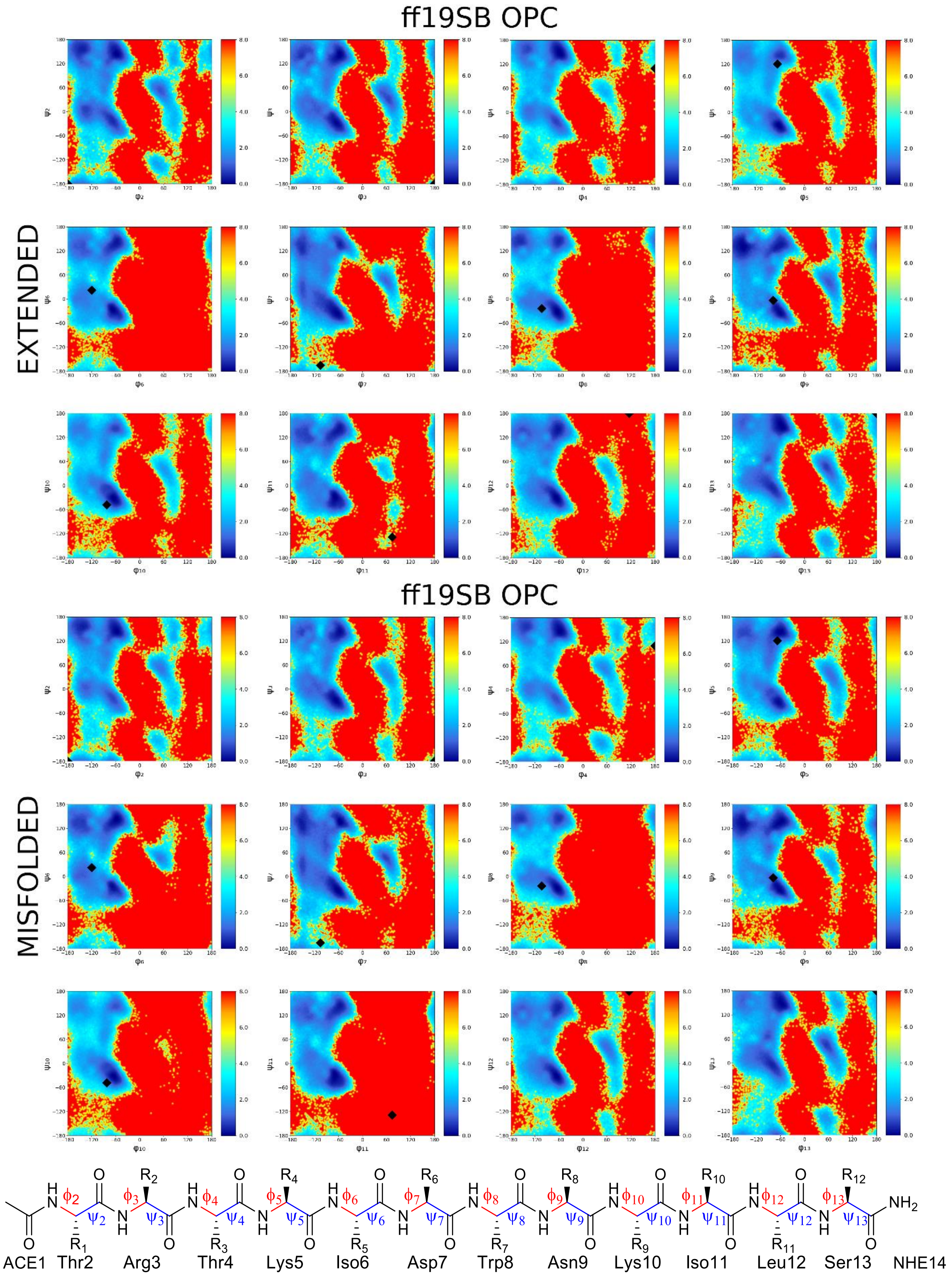

Figure S101. PMFs obtained from φ and ψ dihedral distributions of ID2 residues for the ff19SB/OPC combination from extended (top panels) and misfolded (bottom panels) conformations. PMFs are shown as heat maps (0.0 – 8.0 kcal/mol range) from dark blue to red. The native conformation's φ and ψ angles are reported as black diamonds. ID2 sequence and representation of φ and ψ angles numbering are also shown.

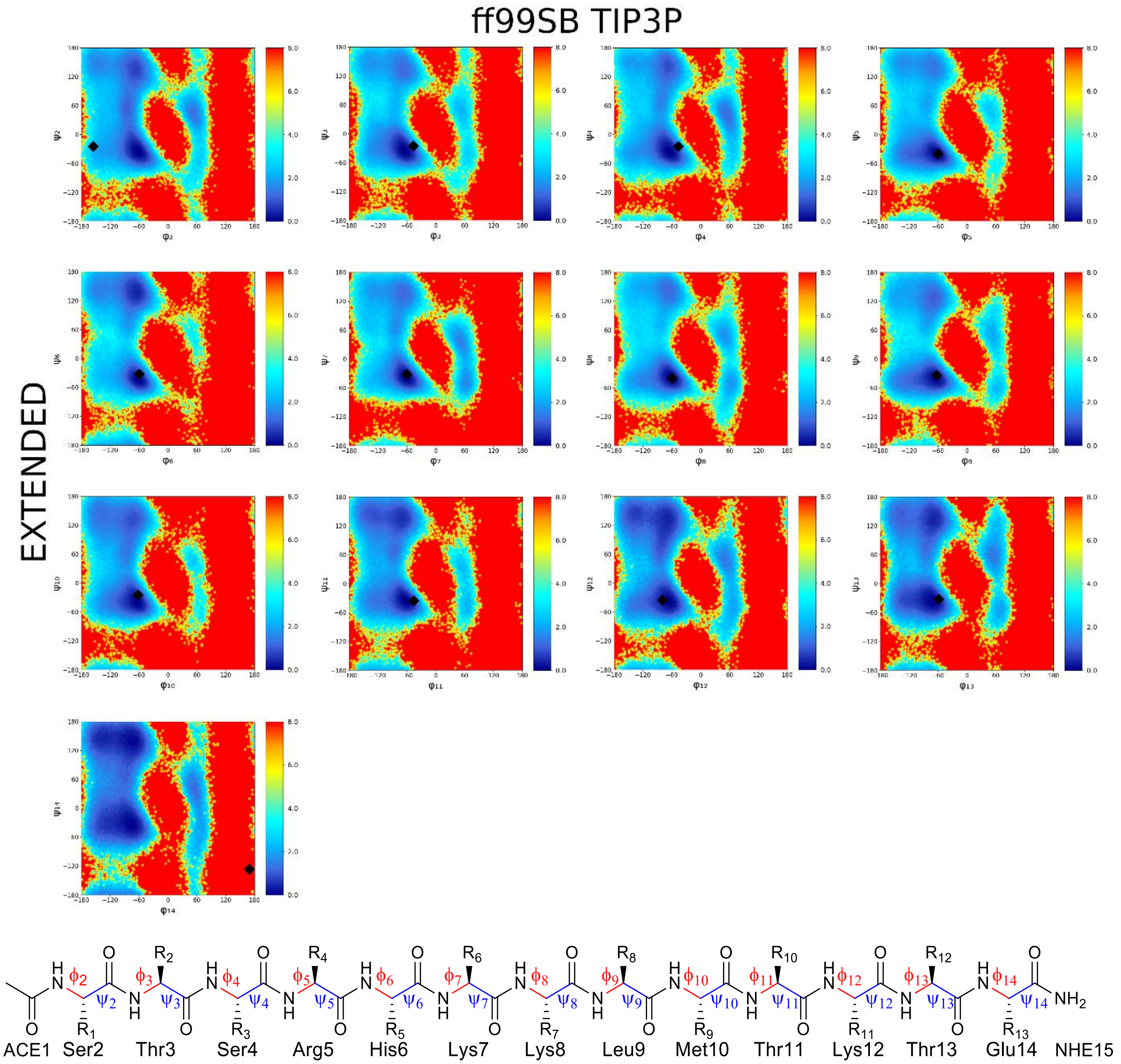

Figure S102. PMFs obtained from φ and ψ dihedral distributions of ID3 residues for ff99SB/TIP3P combination from an extended conformation. PMFs are shown as heat maps (0.0 – 8.0 kcal/mol range) from dark blue to red. The native conformation's φ and ψ angles are reported as black diamonds. ID3 sequence and representation of φ and ψ angles numbering are also shown.

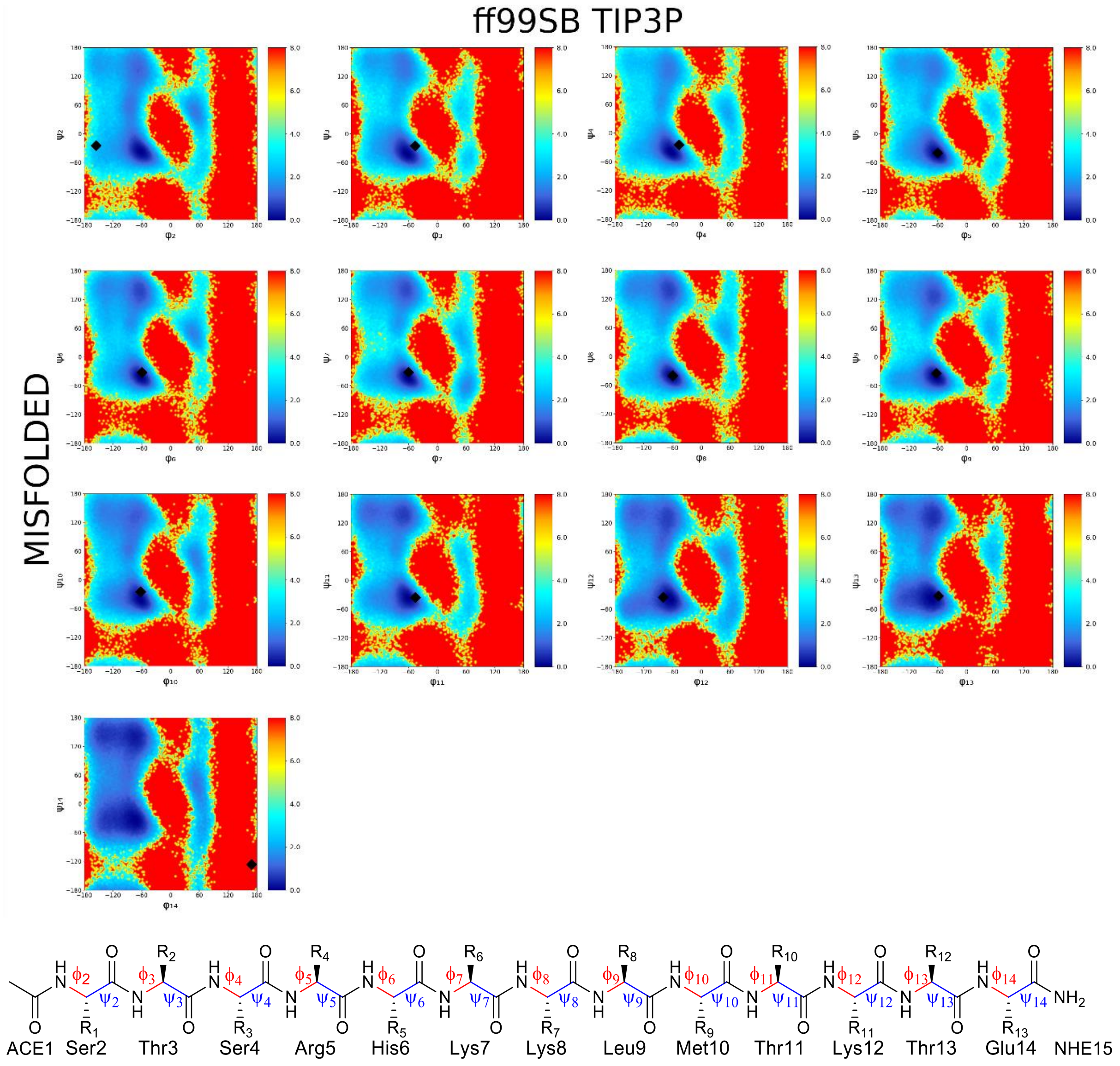

Figure S103. PMFs obtained from φ and ψ dihedral distributions of ID3 residues for the ff99SB/TIP3P combination from a misfolded conformation. PMFs are shown as heat maps (0.0 – 8.0 kcal/mol range) from dark blue to red. The native conformation's φ and ψ angles are reported as black diamonds. ID3 sequence and representation of φ and ψ angles numbering are also shown.

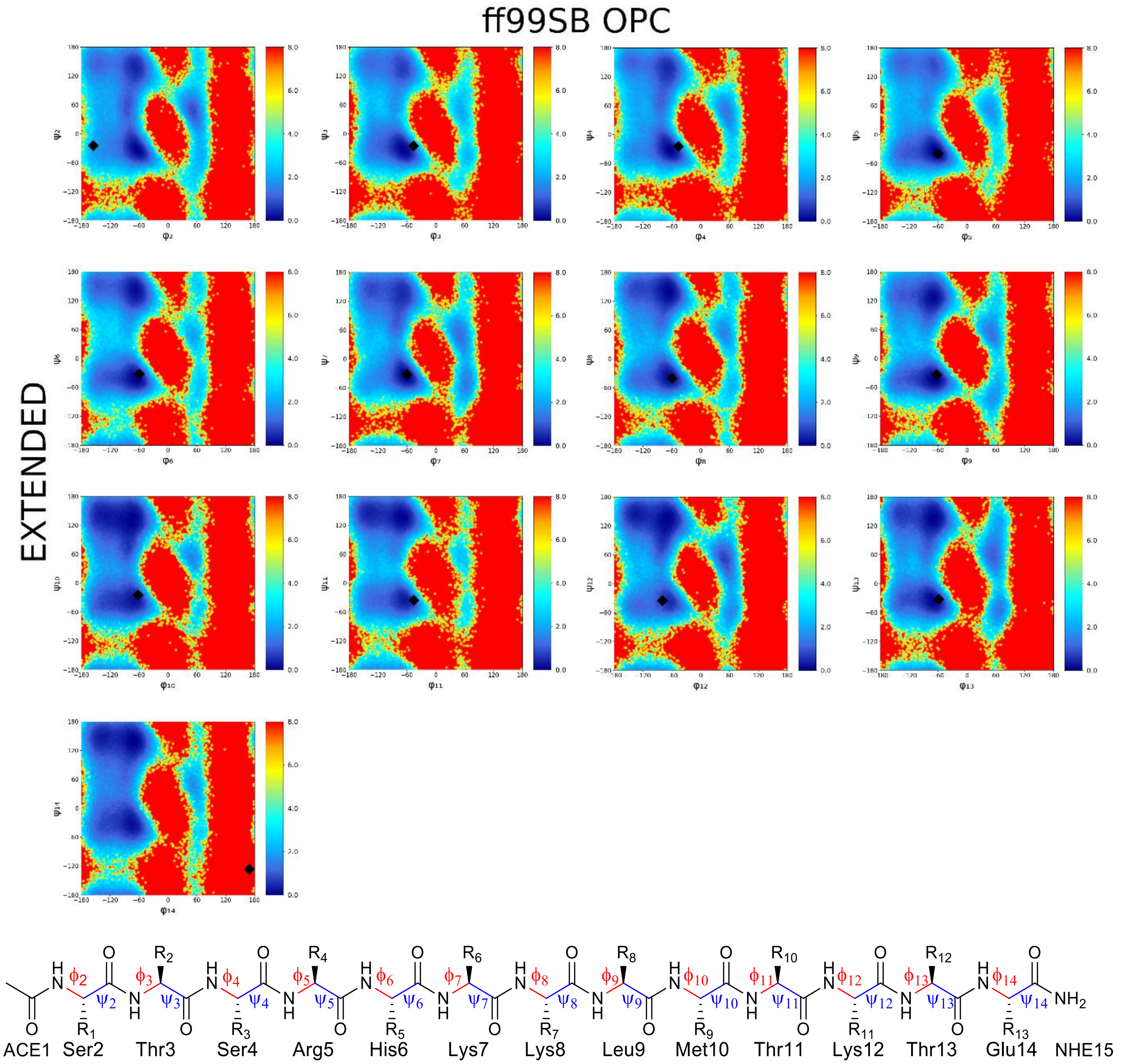

Figure S104. PMFs obtained from φ and ψ dihedral distributions of ID3 residues for the ff99SB/OPC combination from an extended conformation. PMFs are shown as heat maps (0.0 – 8.0 kcal/mol range) from dark blue to red. The native conformation's φ and ψ angles are reported as black diamonds. ID3 sequence and representation of φ and ψ angles numbering are also shown.

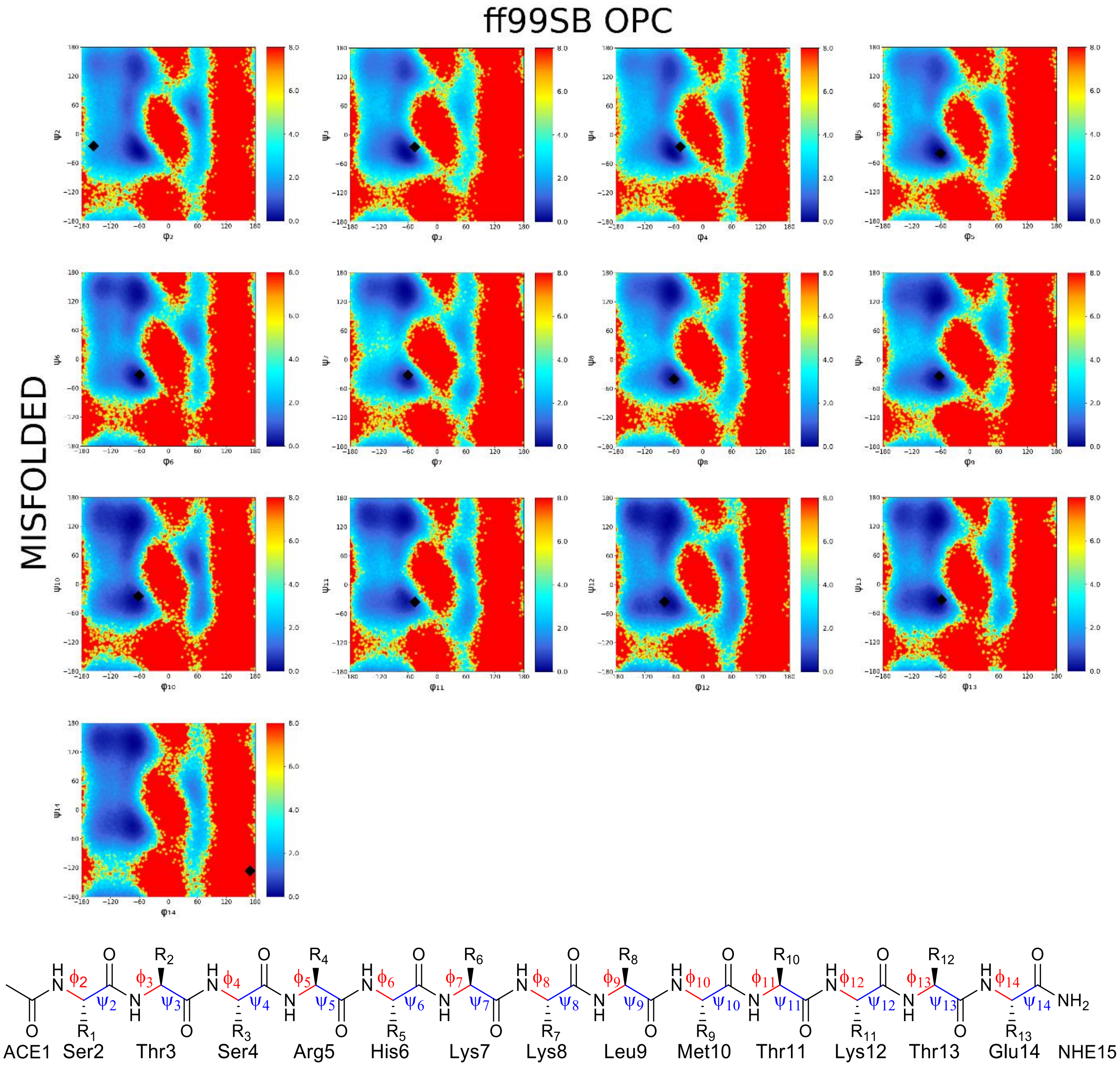

Figure S105. PMFs obtained from φ and ψ dihedral distributions of ID3 residues for the ff99SB/OPC combination from a misfolded conformation. PMFs are shown as heat maps (0.0 – 8.0 kcal/mol range) from dark blue to red. The native conformation's φ and ψ angles are reported as black diamonds. ID3 sequence and representation of φ and ψ angles numbering are also shown.

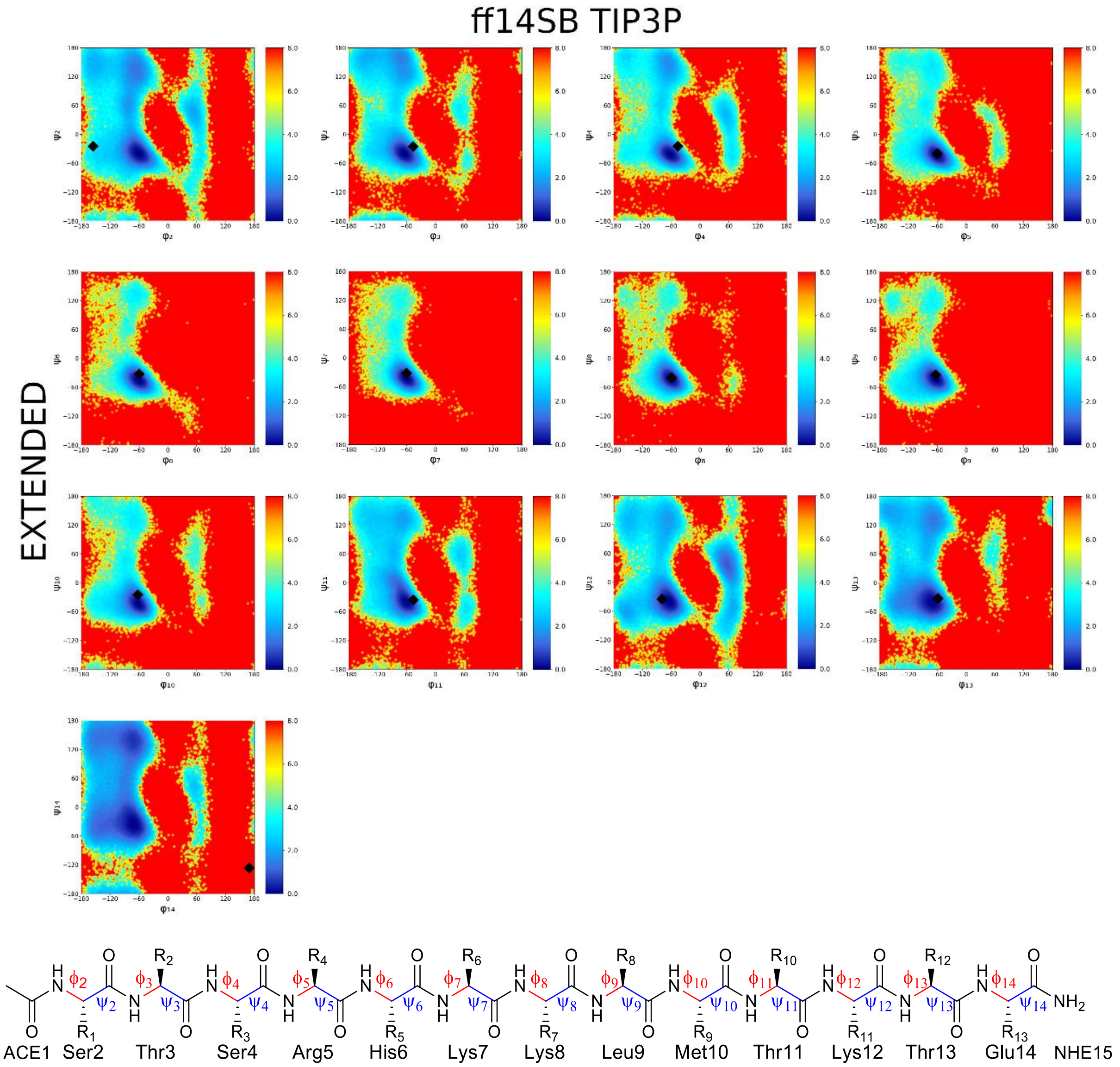

Figure S106. PMFs obtained from φ and ψ dihedral distributions of ID3 residues for the ff14SB/TIP3P combination from an extended conformation. PMFs are shown as heat maps (0.0 – 8.0 kcal/mol range) from dark blue to red. The native conformation's φ and ψ angles are reported as black diamonds. ID3 sequence and representation of φ and ψ angles numbering are also shown.

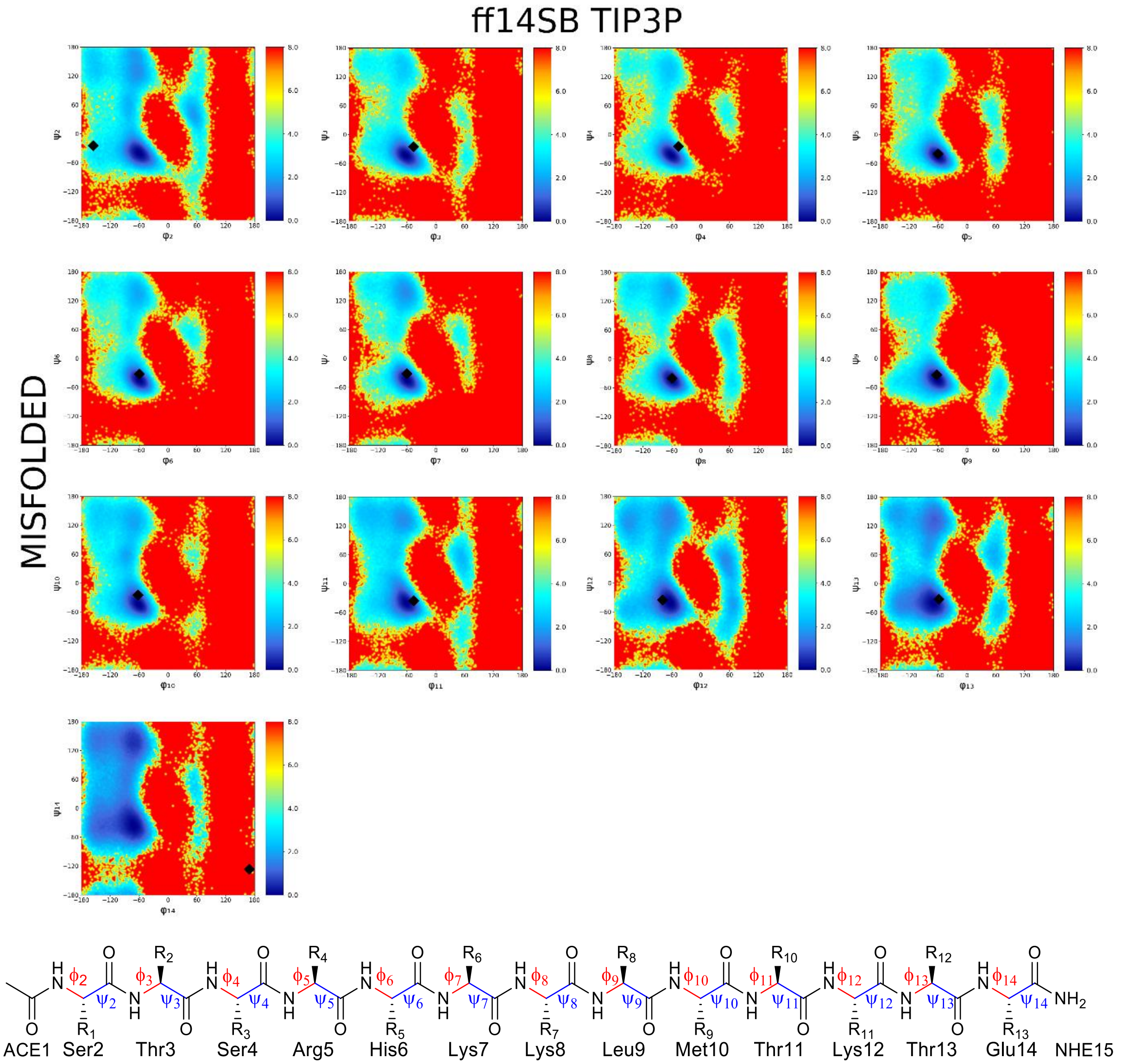

Figure S107. PMFs obtained from φ and ψ dihedral distributions of ID3 residues for the ff14SB/TIP3P combination from a misfolded conformation. PMFs are shown as heat maps (0.0 – 8.0 kcal/mol range) from dark blue to red. The native conformation's φ and ψ angles are reported as black diamonds. ID3 sequence and representation of φ and ψ angles numbering are also shown.

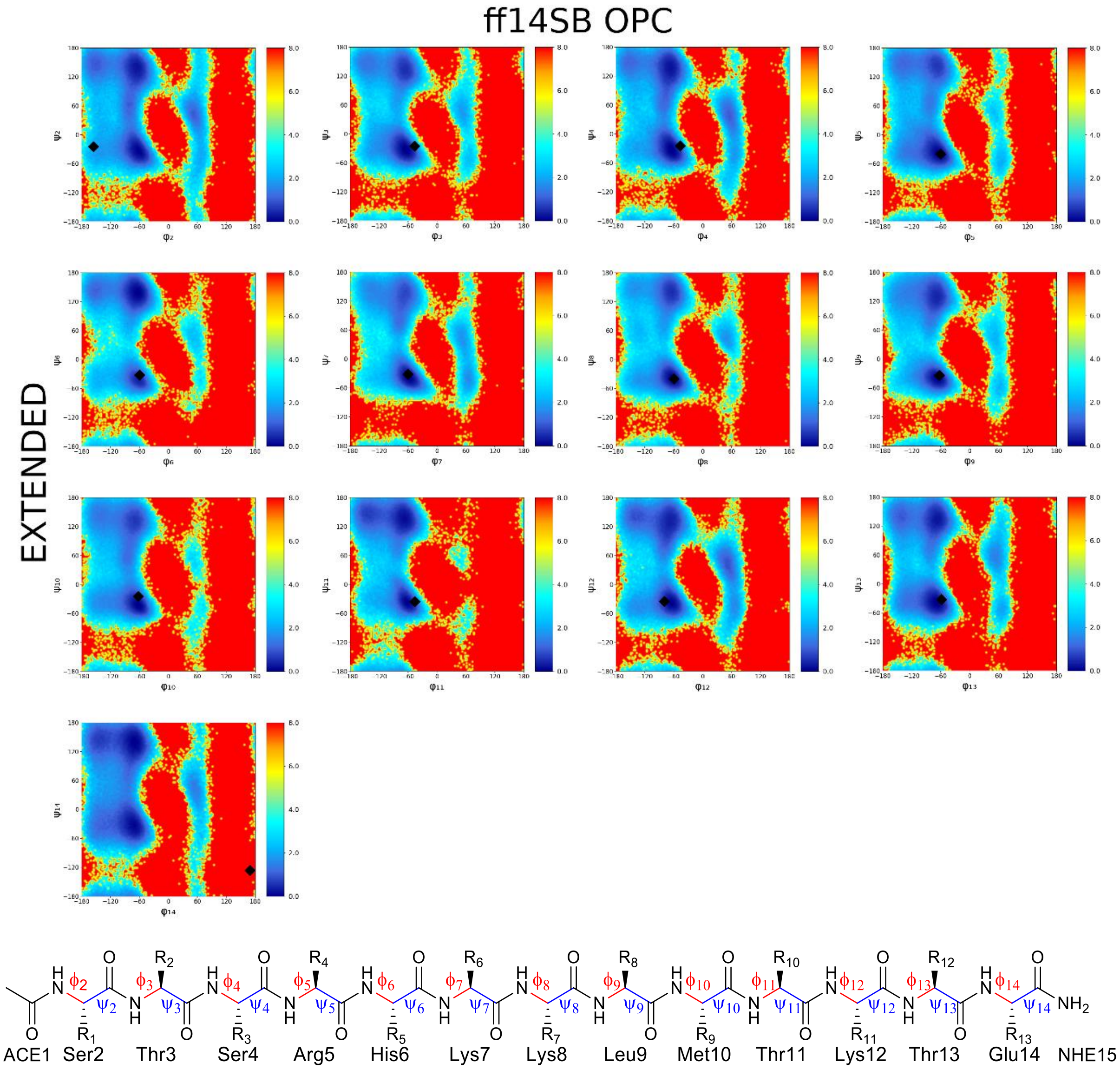

Figure S108. PMFs obtained from φ and ψ dihedral distributions of ID3 residues for the ff14SB/OPC combination from an extended conformation. PMFs are shown as heat maps (0.0 – 8.0 kcal/mol range) from dark blue to red. The native conformation's φ and ψ angles are reported as black diamonds. ID3 sequence and representation of φ and ψ angles numbering are also shown.

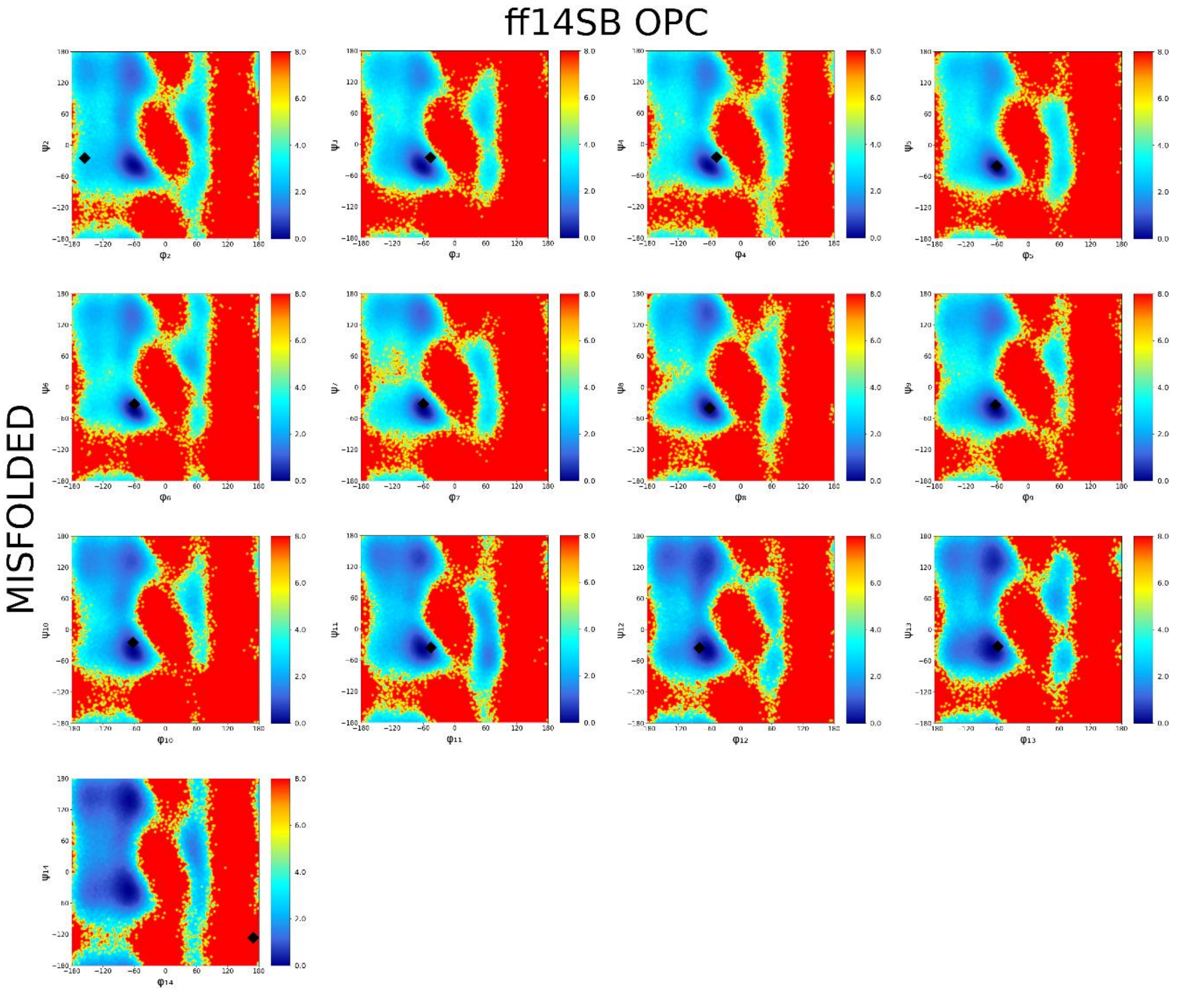

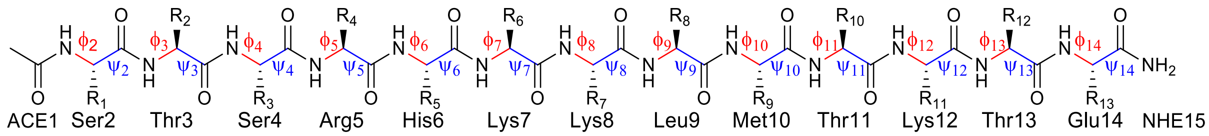

Figure S109. PMFs obtained from φ and ψ dihedral distributions of ID3 residues for the ff14SB/OPC combination from a misfolded conformation. PMFs are shown as heat maps (0.0 – 8.0 kcal/mol range) from dark blue to red. The native conformation's φ and ψ angles are reported as black diamonds. ID3 sequence and representation of φ and ψ angles numbering are also shown.

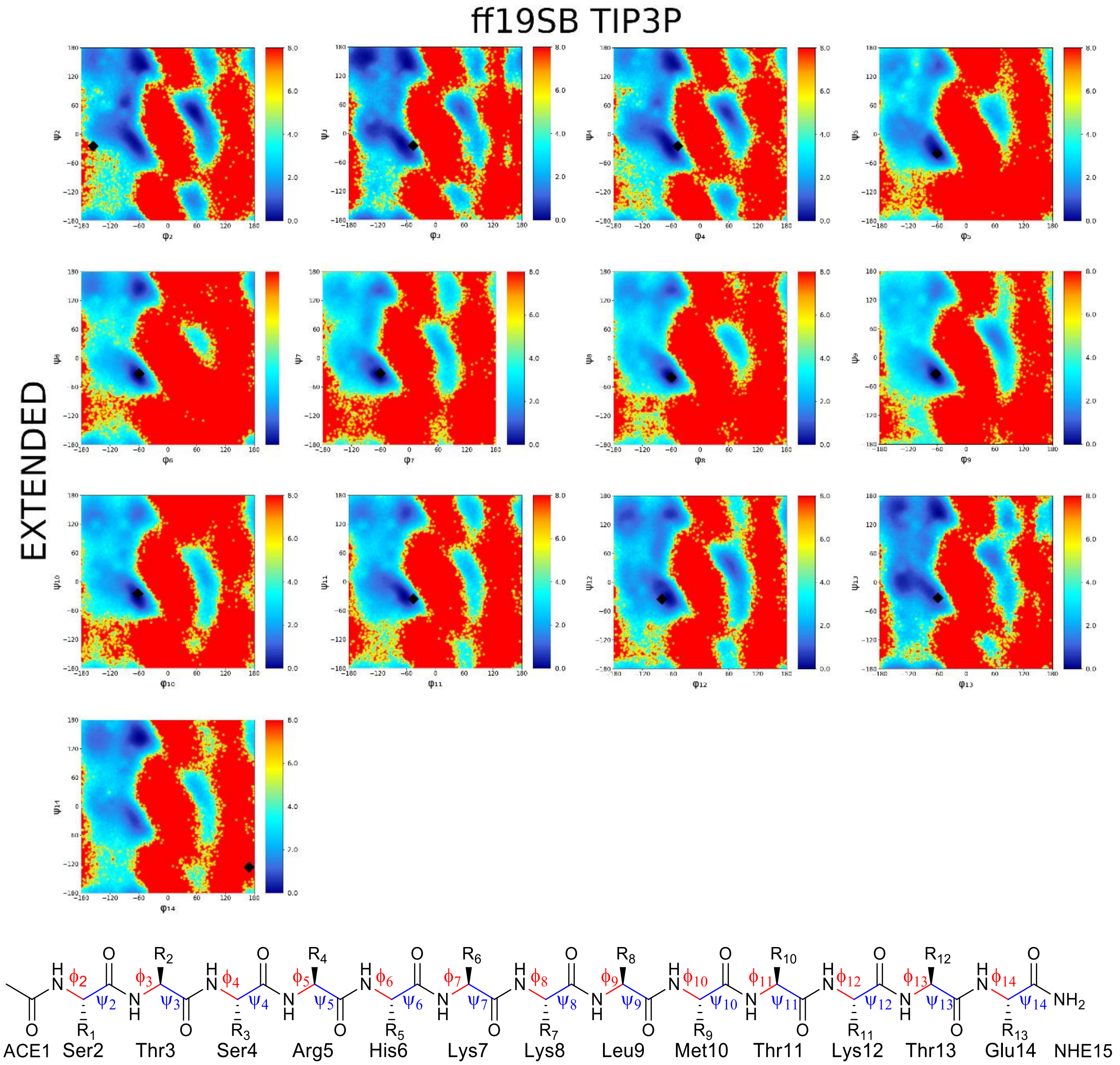

Figure S110. PMFs obtained from φ and ψ dihedral distributions of ID3 residues for the ff19SB/TIP3P combination from an extended conformation. PMFs are shown as heat maps (0.0 – 8.0 kcal/mol range) from dark blue to red. The native conformation's φ and ψ angles are reported as black diamonds. ID3 sequence and representation of φ and ψ angles numbering are also shown.

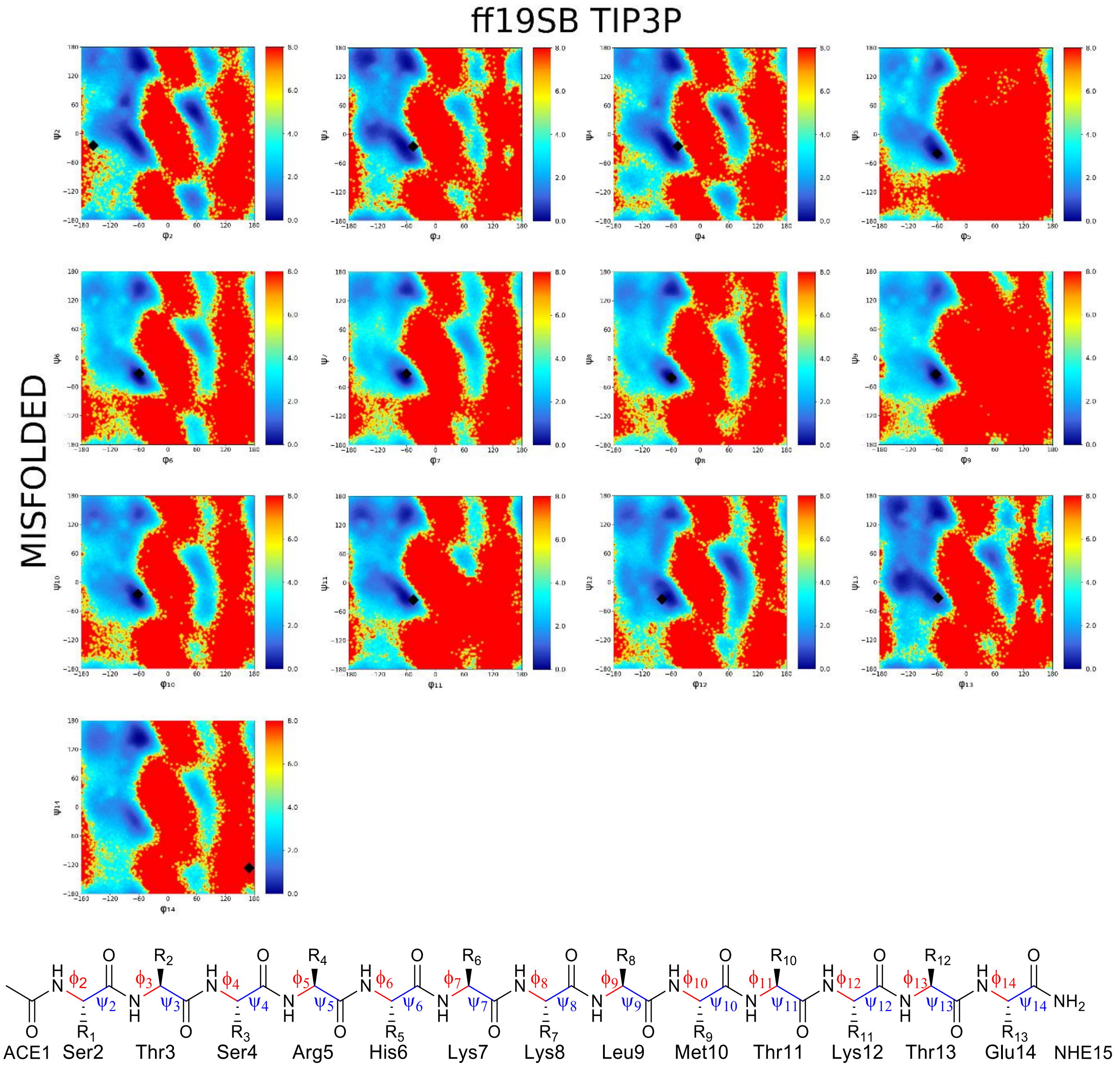

Figure S111. PMFs obtained from φ and ψ dihedral distributions of ID3 residues for the ff19SB/TIP3P combination from a misfolded conformation. PMFs are shown as heat maps (0.0 – 8.0 kcal/mol range) from dark blue to red. The native conformation's φ and ψ angles are reported as black diamonds. ID3 sequence and representation of φ and ψ angles numbering are also shown.

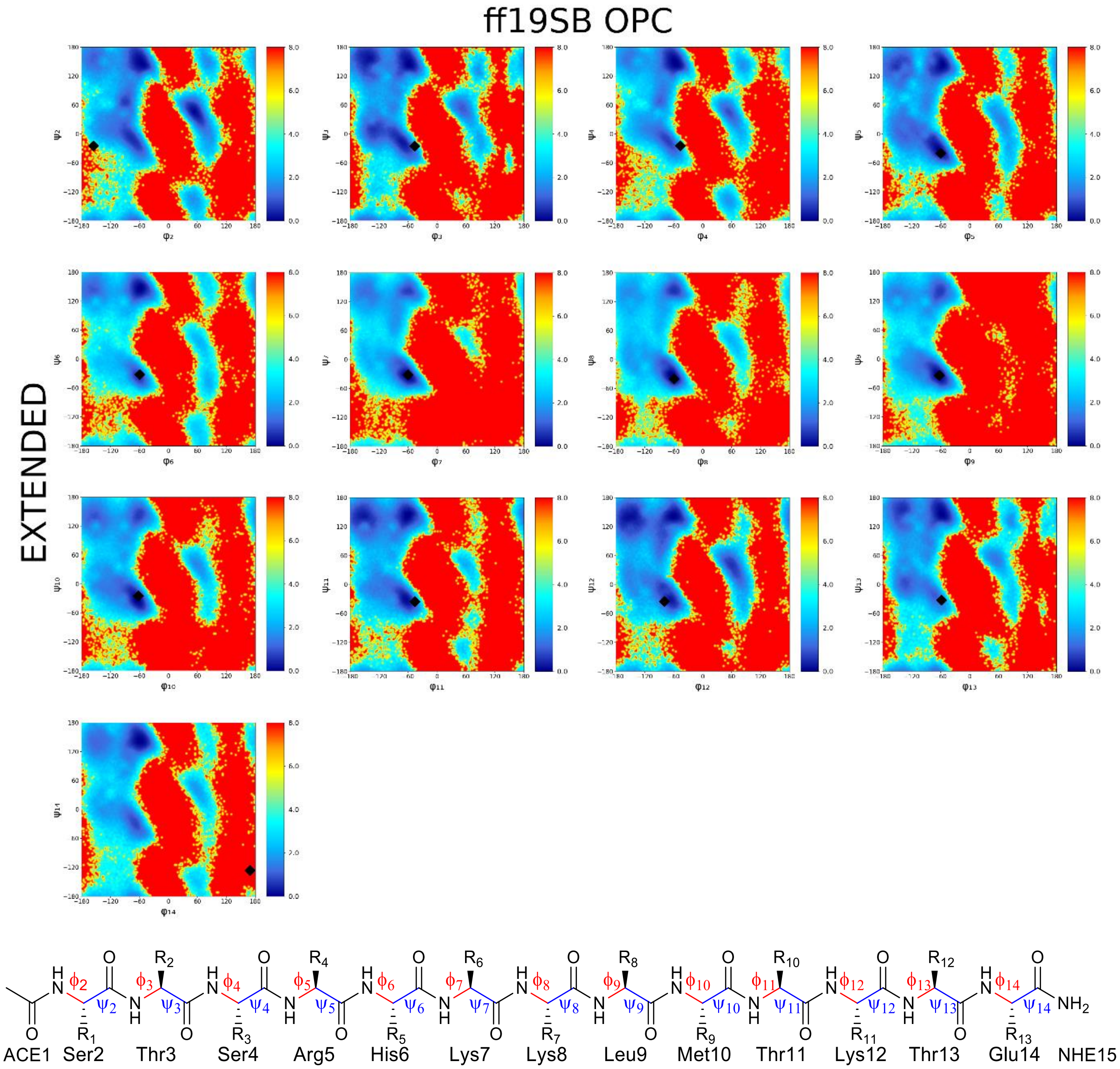

Figure S112. PMFs obtained from φ and ψ dihedral distributions of ID3 residues for the ff19SB/OPC combination from an extended conformation. PMFs are shown as heat maps (0.0 – 8.0 kcal/mol range) from dark blue to red. The native conformation's φ and ψ angles are reported as black diamonds. ID3 sequence and representation of φ and ψ angles numbering are also shown.

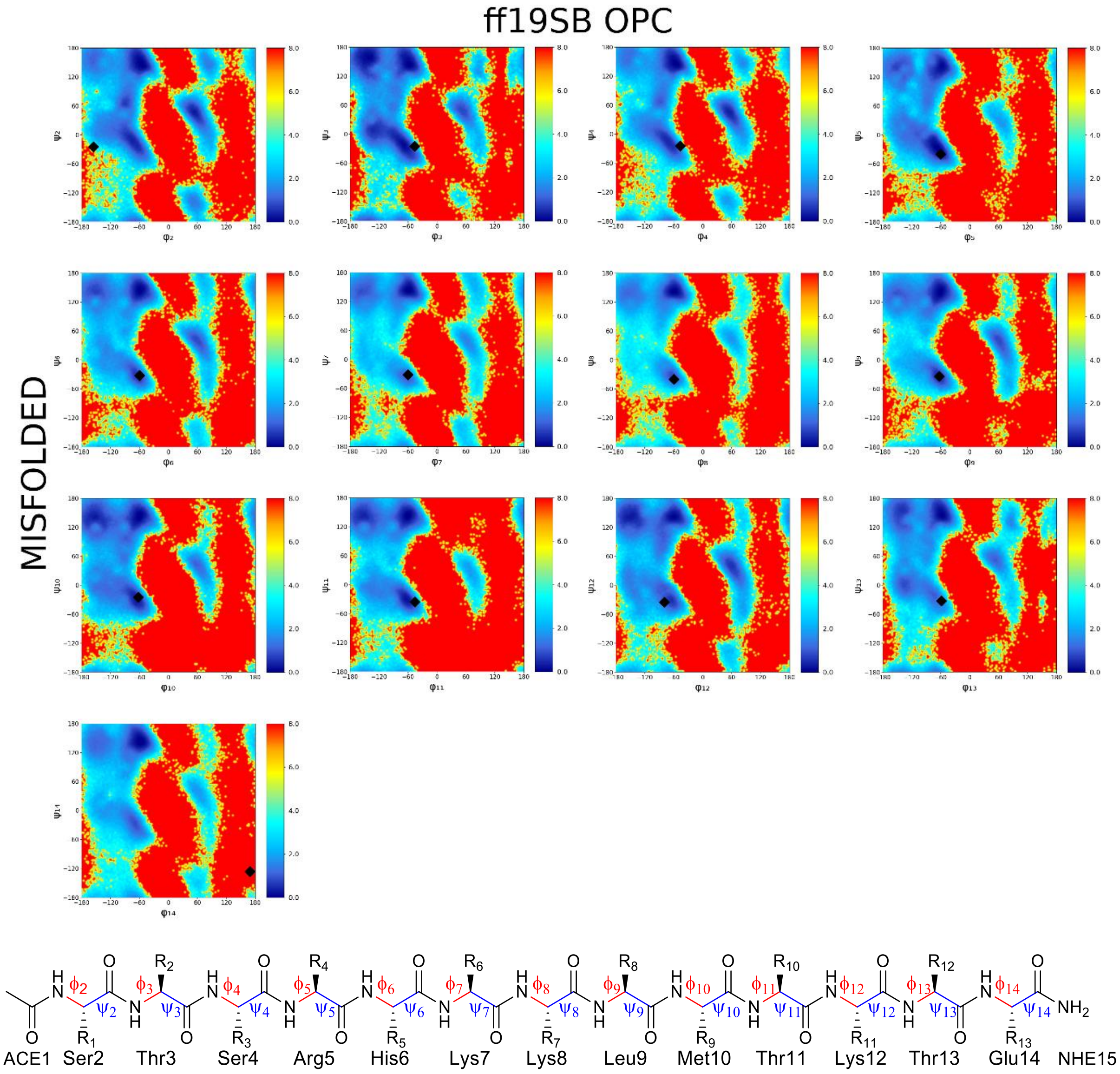

Figure S113. PMFs obtained from φ and ψ dihedral distributions of ID3 residues for the ff19SB/OPC combination from a misfolded conformation. PMFs are shown as heat maps (0.0 – 8.0 kcal/mol range) from dark blue to red. The native conformation's φ and ψ angles are reported as black diamonds. ID3 sequence and representation of φ and ψ angles numbering are also shown.

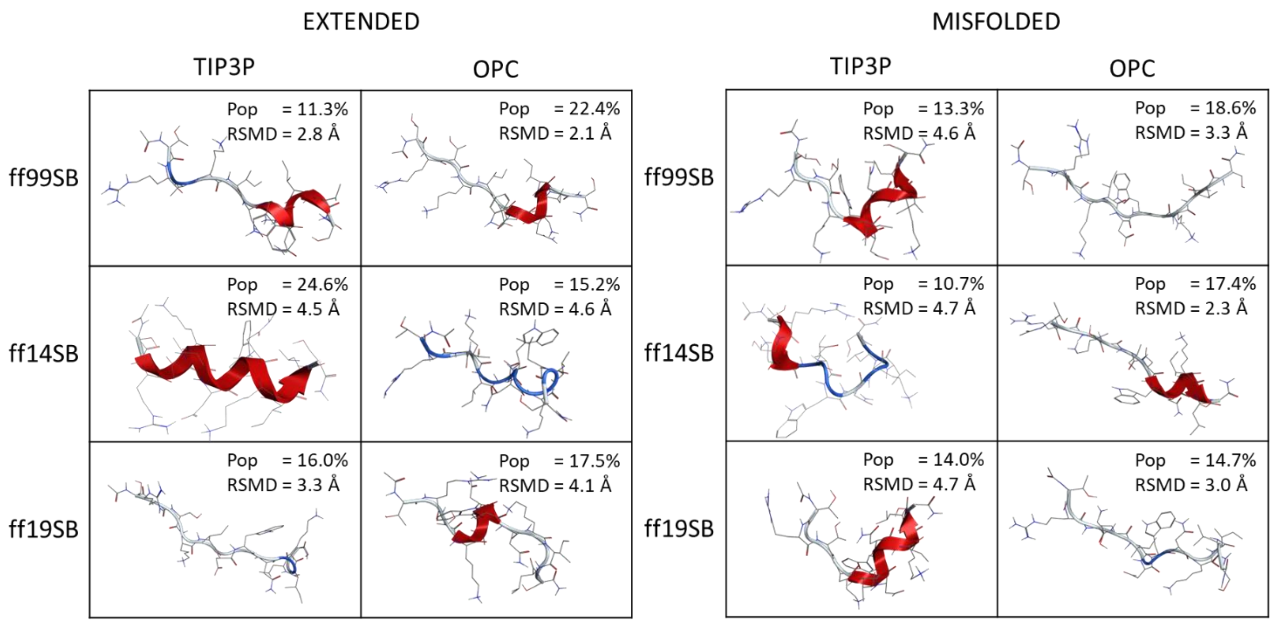

Figure S114. Representative conformation and population (pop%) of the second clusters from ID2 trajectories. RMSDs vs native structure are also shown.

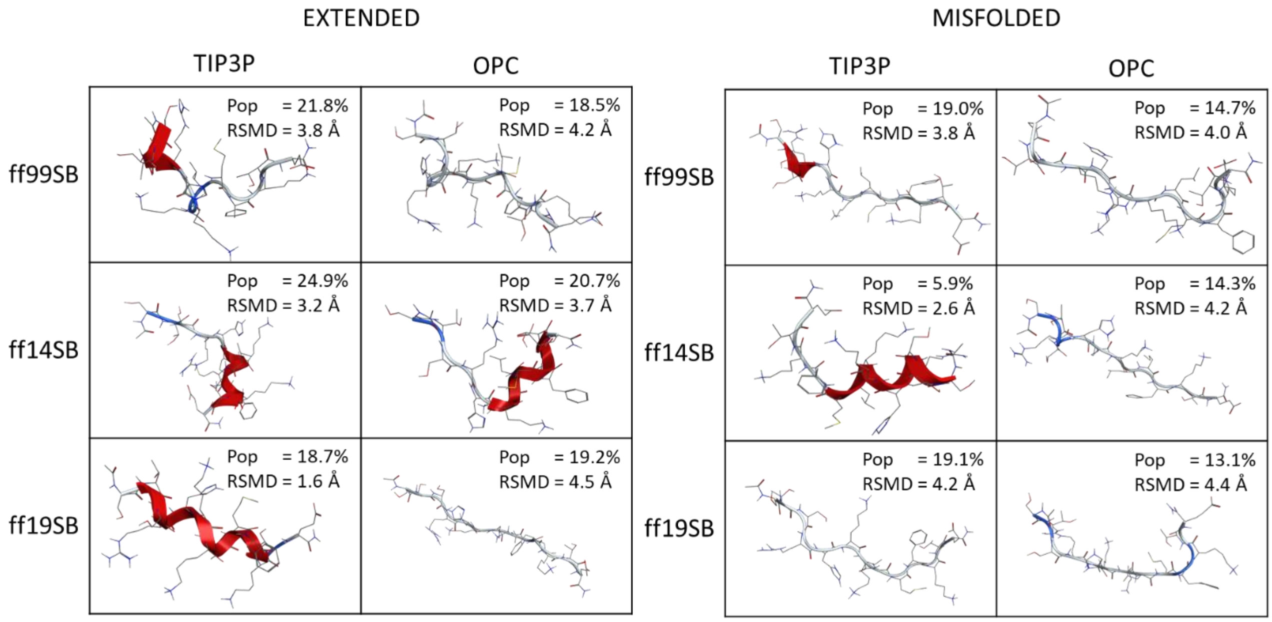

Figure S115. Representative conformation and population (pop%) of the second clusters from ID3 trajectories. RMSDs vs native structure are also shown.

Table S10. H-bond analyses from ID2 trajectories. Only H-bonds involving the backbone are included in the table (donor N-H, acceptor C=O). Native H-bonds are highlighted in bold.

NATIVE		
acceptor	donor	Distance
**TRP8**	**LEU12**	**2.52**
**THR4**	**ILE6**	**3.16**
**TRP8**	**LYS10**	**3.21**
**ASP7**	**ASN9**	**3.51**
**ASP7**	**LYS10**	**3.54**
**LYS5**	**ASP7**	**3.68**
**ASN9**	**LEU12**	**3.92**
**ASN9**	**ILE11**	**4.13**

ff99SB OPC							ff99SB TIP3P					
EXTENDED			MISFOLDED				EXTENDED			MISFOLDED		
acceptor	donor	occ%	acceptor	donor	occ%		acceptor	donor	occ%	acceptor	donor	occ%
**TRP8**	**LEU12**	23.83	ASN9	SER13	18.21		**TRP8**	**LEU12**	6.58	**ASN9**	**ILE11**	5.02
ASN9	SER13	21.21	LYS5	ASN9	17.94		**THR4**	**ILE6**	5.49			
LYS5	ASN9	20.76	TRP8	LEU12	17.24		ASN9	SER13	5.42			
THR4	TRP8	17.57	THR4	TRP8	14.43		**ASN9**	**ILE11**	5.04			
ILE6	LYS10	13.69	THR2	ILE6	12.52							
THR2	ILE6	12.48	ILE6	LYS10	12.19							
ARG3	ASP7	11.66	ARG3	ASP7	11.98							
TRP8	ILE11	9.84	ACE1	LYS5	8.34							
THR4	ASP7	7.66	THR2	LYS5	7.32							
THR2	LYS5	7.37	TRP8	ILE11	7.25							
ASP7	ILE11	7.35	THR4	ASP7	6.16							
ACE1	LYS5	6.38	ASP7	ILE11	5.49							
ASP7	LYS10	6.07	ARG3	TRP8	5.33							

ff14SB OPC							ff14SB TIP3P					
EXTENDED			MISFOLDED				EXTENDED			MISFOLDED		
acceptor	donor	occ%	acceptor	donor	occ%		acceptor	donor	occ%	acceptor	donor	occ%
**TRP8**	**LEU12**	21.07	**TRP8**	**LEU12**	14.48		**TRP8**	**LEU12**	43.80	LYS5	ASN9	34.73
ASN9	SER13	16.54	ASN9	SER13	13.31		ASN9	SER13	32.73	**TRP8**	**LEU12**	34.35
LYS5	ASN9	15.67	LYS5	ASN9	7.78		ASP7	ILE11	17.49	ASN9	SER13	32.10
THR4	TRP8	11.90	THR4	TRP8	7.24		ACE1	LYS5	16.26	THR4	TRP8	31.02
ILE6	LYS10	9.60	THR2	ILE6	6.60		LYS5	ASN9	14.05	ILE6	LYS10	26.98
TRP8	ILE11	9.34	ASP7	ILE11	6.57		**ASP7**	**LYS10**	13.33	ASP7	ILE11	16.05
ASP7	ILE11	7.37	TRP8	ILE11	6.11		ILE6	LYS10	13.16	ARG3	ASP7	14.02
**ASP7**	**LYS10**	5.09	**ASP7**	**LYS10**	6.09		THR2	ILE6	12.04	THR2	ILE6	10.92
			ARG3	LYS5	5.35		ARG3	ASP7	10.51	TRP8	ILE11	7.68
			**THR4**	**ILE6**	5.08		THR2	THR4	10.23	THR4	ASP7	6.43
			THR2	ASP7	5.07		THR4	TRP8	9.43	ACE1	LYS5	6.14
							THR2	LYS5	9.35	THR2	LYS5	5.27
							TRP8	ILE11	9.11			
							ACE1	ILE6	7.38			
							ILE6	ARG3	6.30			

ff19SB OPC						
EXTENDED				MISFOLDED		
acceptor	donor	occ%		acceptor	donor	occ%
LYS5	ASN9	8.84		ASN9	SER13	6.87
THR4	ASP7	6.32		**TRP8**	**LEU12**	5.89
THR4	TRP8	6.11				
ASN9	SER13	5.41				

ff19SB TIP3P						
EXTENDED				MISFOLDED		
acceptor	donor	occ%		acceptor	donor	occ%
**TRP8**	**LEU12**	20.56		**TRP8**	**LEU12**	24.94
ASN9	SER13	17.23		ASN9	SER13	16.97
THR4	TRP8	15.66		LYS5	ASN9	13.61
LYS5	ASN9	15.54		**ASP7**	**LYS10**	10.49
ILE6	LYS10	9.85		ASP7	ILE11	10.13
ASP7	ILE11	8.63		THR4	TRP8	9.96
THR4	ASP7	7.66		ILE6	LYS10	9.49
**ASP7**	**LYS10**	7.05		TRP8	ILE11	7.73
TRP8	ILE11	6.77		ILE6	ASN9	6.92

Table S11. H-bond analyses from ID3 trajectories. Only H-bonds involving the backbone are included in the table (donor N-H, acceptor C=O). Native H-bonds are highlighted in bold.

NATIVE		
acceptor	donor	Distance
**THR3**	**ARG5**	**2.56**
**MET10**	**THR13**	**2.59**
**SER2**	**SER4**	**2.59**
**THR3**	**HIE6**	**2.61**
**SER2**	**ARG5**	**2.7**
**MET10**	**LYS12**	**2.78**
**ARG5**	**LYS8**	**2.83**
**HIE6**	**LEU9**	**2.88**
**MET10**	**GLU14**	**2.92**
**SER4**	**LYS7**	**2.93**
**LYS7**	**MET10**	**2.95**
**LYS12**	**GLU14**	**3.01**
**ARG5**	**LYS7**	**3.07**
**LYS8**	**PHE11**	**3.08**
**LEU9**	**PHE11**	**3.08**
**HIE6**	**LYS8**	**3.09**
**LYS7**	**LEU9**	**3.19**
**SER4**	**HIE6**	**3.25**
**LYS8**	**MET10**	**3.26**
**LEU9**	**LYS12**	**3.3**
**HIE6**	**MET10**	**3.34**
**PHE11**	**GLU14**	**3.48**
**LYS7**	**PHE11**	**3.49**
**SER4**	**LYS8**	**3.56**
**PHE11**	**THR13**	**3.67**
**ARG5**	**LEU9**	**3.68**
**THR3**	**LYS7**	**3.78**
**LEU9**	**THR13**	**3.99**
**GLU14**	**LYS12**	**4.16**

ff99SB OPC	ff99SB TIP3P

EXTENDED		
acceptor	donor	occ%
**1792**	**LYS7**	7.65
SER2	HIE6	6.12
**HIE6**	**MET10**	5.10
**SER4**	**LYS8**	5.01

MISFOLDED		
acceptor	donor	occ%
SER2	HIP6	8.15
**THR3**	**LYS7**	7.12
ACE1	ARG5	5.41

EXTENDED		
acceptor	donor	occ%
**HIE6**	**MET10**	17.34
SER2	HIE6	15.73
**ARG5**	**LEU9**	14.42
**LYS7**	**PHE11**	14.16
**SER4**	**LYS8**	12.11
ACE1	ARG5	10.18
**THR3**	**LYS7**	9.37
**MET10**	**GLU14**	8.13
LYS8	LYS12	7.92
**SER2**	**ARG5**	7.02
**LEU9**	**THR13**	6.04
**HIE6**	**LEU9**	5.01

MISFOLDED		
acceptor	donor	occ%
**THR3**	**LYS7**	18.82
SER2	HIP6	18.13
**SER4**	**LYS8**	15.22
HIP6	MET10	15.17
ACE1	ARG5	14.59
**LYS7**	**PHE11**	13.56
**MET10**	**GLU14**	11.67
LYS8	LYS12	11.44
**ARG5**	**LEU9**	11.24
**LEU9**	**THR13**	7.87

ff14SB OPC

EXTENDED		
acceptor	donor	occ%
**HIE6**	**MET10**	13.64
**LYS7**	**PHE11**	10.50
**MET10**	**GLU14**	9.14
SER2	HIE6	6.32
**LEU9**	**THR13**	5.44
**HIE6**	**LEU9**	5.04

MISFOLDED		
acceptor	donor	occ%
**THR3**	**LYS7**	27.30
SER2	HIP6	25.49
**SER4**	**LYS8**	24.71
HIP6	MET10	23.50
**ARG5**	**LEU9**	19.84
ACE1	ARG5	17.80
**LYS7**	**PHE11**	16.73
**LEU9**	**THR13**	10.52
LYS8	LYS12	10.38
**MET10**	**GLU14**	10.33

ff14SB TIP3P

EXTENDED		
acceptor	donor	occ%
**HIE6**	**MET10**	50.07
**LYS7**	**PHE11**	42.78
**SER4**	**LYS8**	41.18
**ARG5**	**LEU9**	39.36
**THR3**	**LYS7**	37.22
**MET10**	**GLU14**	26.65
LYS8	LYS12	26.30
SER2	HIE6	25.43
**LEU9**	**THR13**	23.56
ACE1	ARG5	22.46
LYS8	THR13	5.00

MISFOLDED		
acceptor	donor	occ%
**THR3**	**LYS7**	48.00
SER2	HIP6	43.97
**SER4**	**LYS8**	40.34
HIP6	MET10	37.80
**ARG5**	**LEU9**	35.00
**LYS7**	**PHE11**	32.72
ACE1	ARG5	30.94
**MET10**	**GLU14**	26.32
LYS8	LYS12	25.39
**LEU9**	**THR13**	21.77

ff19SB OPC

EXTENDED		
acceptor	donor	occ%
**HIE6**	**MET10**	9.60
**LYS7**	**PHE11**	9.57
**HIE6**	**LEU9**	8.39
**LYS7**	**MET10**	5.89

MISFOLDED		
acceptor	donor	occ%

ff19SB TIP3P

EXTENDED		
acceptor	donor	occ%
**HIE6**	**MET10**	13.28
**SER4**	**LYS8**	11.79
**LYS7**	**PHE11**	11.03
**ARG5**	**LEU9**	9.60
LYS8	LYS12	8.91
**LEU9**	**LYS12**	6.82
**LYS8**	**PHE11**	6.66
**HIE6**	**LEU9**	6.13
**THR3**	**LYS7**	6.09
**LEU9**	**THR13**	5.63
**MET10**	**GLU14**	5.49
**SER4**	**LYS7**	5.17

MISFOLDED		
acceptor	donor	occ%
HIP6	MET10	11.72
**ARG5**	**LEU9**	11.58
**LYS7**	**PHE11**	10.63
**SER4**	**LYS8**	10.11
**THR3**	**LYS7**	5.80

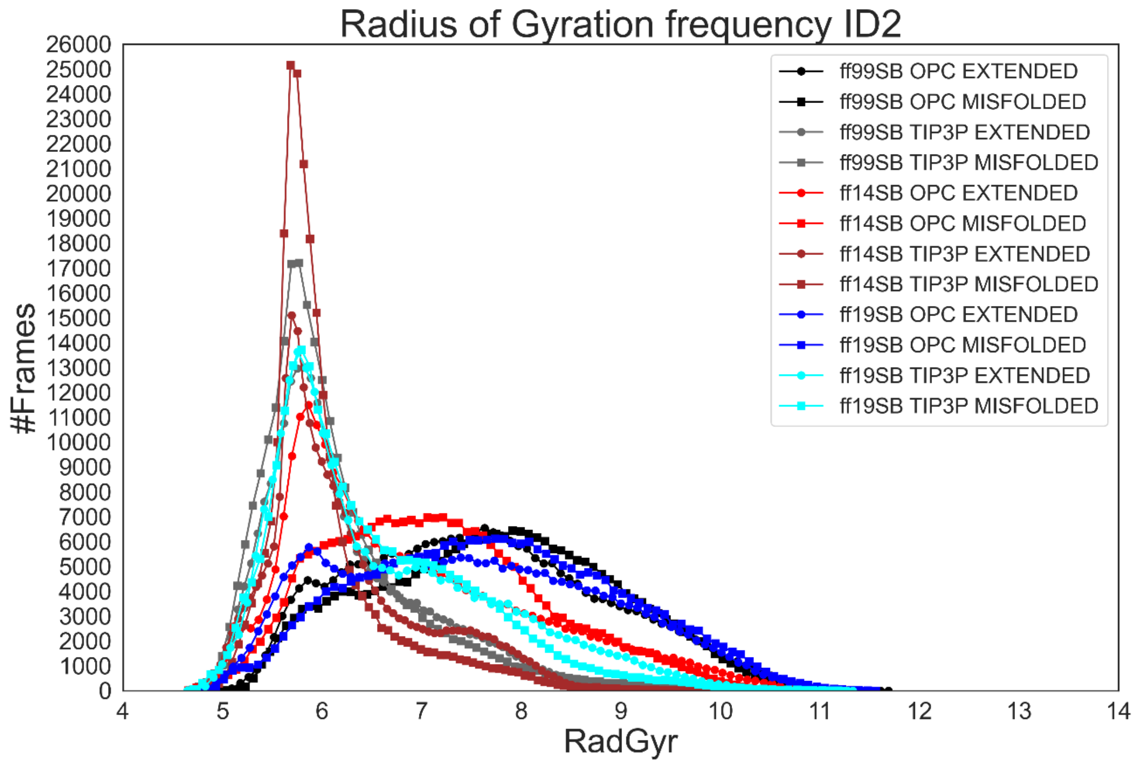

Figure S116. Radius of gyration frequencies calculated on the last 500 ns of ID2 trajectories.

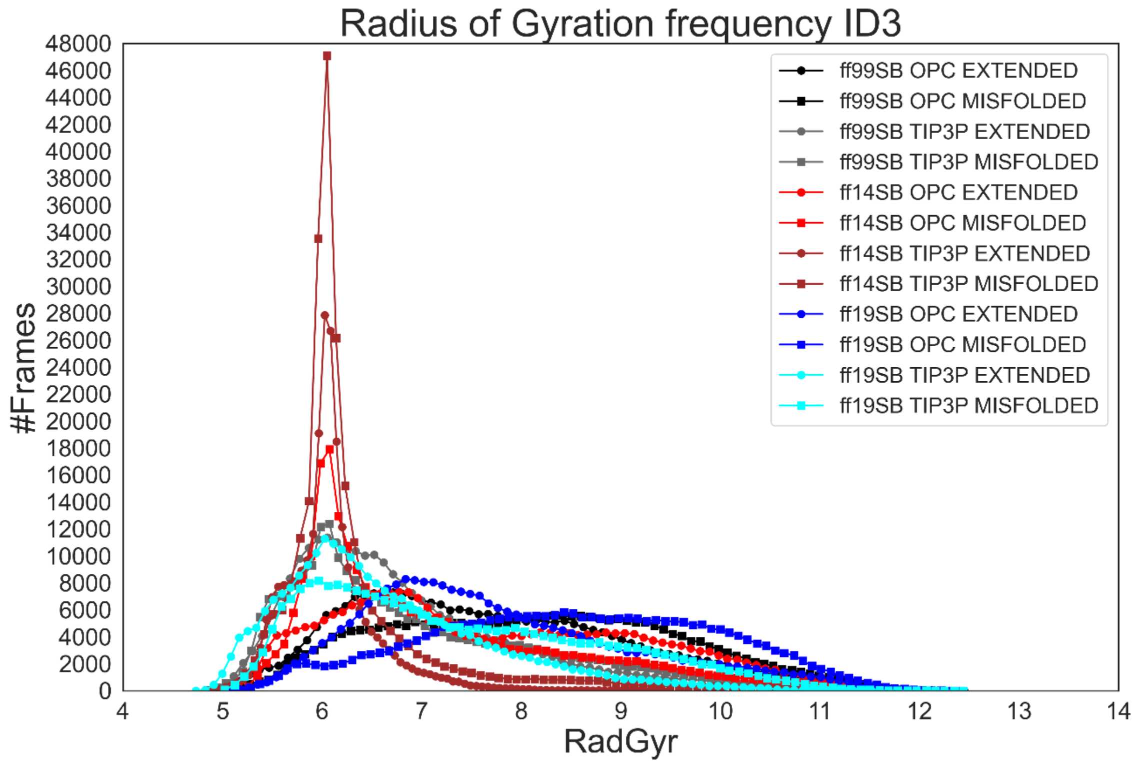

Figure S117. Radius of gyration frequencies calculated on the last 500 ns of ID3 trajectories.

PDB coordinate of the starting structures.

H1 extended

```
ATOM 1 CH3 ACE 1 33.886 24.866 20.687 1.00 0.00 C
ATOM 2 C ACE 1 34.349 25.487 22.029 1.00 0.00 C
ATOM 3 O ACE 1 35.523 25.237 22.383 1.00 0.00 O
ATOM 4 N LYS 2 33.495 26.383 22.610 1.00 0.00 N
ATOM 5 CA LYS 2 33.521 27.193 23.881 1.00 0.00 C
ATOM 6 CB LYS 2 34.328 28.544 23.721 1.00 0.00 C
ATOM 7 CG LYS 2 33.715 29.463 22.665 1.00 0.00 C
ATOM 8 CD LYS 2 34.444 30.836 22.501 1.00 0.00 C
ATOM 9 CE LYS 2 34.253 31.767 23.672 1.00 0.00 C
ATOM 10 NZ LYS 2 34.819 33.142 23.409 1.00 0.00 N1+
ATOM 11 C LYS 2 32.059 27.345 24.436 1.00 0.00 C
ATOM 12 O LYS 2 31.046 27.373 23.727 1.00 0.00 O
ATOM 13 N LEU 3 31.923 27.417 25.779 1.00 0.00 N
ATOM 14 CA LEU 3 30.600 27.108 26.345 1.00 0.00 C
ATOM 15 CB LEU 3 30.847 25.882 27.303 1.00 0.00 C
ATOM 16 CG LEU 3 29.715 25.411 28.161 1.00 0.00 C
ATOM 17 CD1 LEU 3 28.525 24.979 27.268 1.00 0.00 C
ATOM 18 CD2 LEU 3 30.147 24.264 28.963 1.00 0.00 C
ATOM 19 C LEU 3 30.098 28.295 27.216 1.00 0.00 C
ATOM 20 O LEU 3 30.770 28.660 28.174 1.00 0.00 O
ATOM 21 N THR 4 28.943 28.772 26.922 1.00 0.00 N
ATOM 22 CA THR 4 28.124 29.588 27.862 1.00 0.00 C
ATOM 23 CB THR 4 27.292 30.615 27.131 1.00 0.00 C
ATOM 24 CG2 THR 4 28.245 31.849 26.715 1.00 0.00 C
ATOM 25 OG1 THR 4 26.516 30.116 26.042 1.00 0.00 O
ATOM 26 C THR 4 27.170 28.751 28.688 1.00 0.00 C
ATOM 27 O THR 4 26.631 27.757 28.164 1.00 0.00 O
ATOM 28 N TRP 5 26.992 29.046 30.013 1.00 0.00 N
ATOM 29 CA TRP 5 26.156 28.193 30.953 1.00 0.00 C
ATOM 30 CB TRP 5 26.186 28.781 32.341 1.00 0.00 C
ATOM 31 CG TRP 5 25.582 30.165 32.384 1.00 0.00 C
ATOM 32 CD1 TRP 5 26.288 31.318 32.276 1.00 0.00 C
ATOM 33 NE1 TRP 5 25.329 32.344 32.241 1.00 0.00 N
ATOM 34 CE2 TRP 5 24.063 31.879 32.527 1.00 0.00 C
ATOM 35 CZ2 TRP 5 22.846 32.559 32.674 1.00 0.00 C
ATOM 36 CH2 TRP 5 21.722 31.764 32.868 1.00 0.00 C
ATOM 37 CZ3 TRP 5 21.804 30.382 33.043 1.00 0.00 C
ATOM 38 CE3 TRP 5 23.013 29.735 32.875 1.00 0.00 C
ATOM 39 CD2 TRP 5 24.210 30.482 32.643 1.00 0.00 C
ATOM 40 C TRP 5 24.728 27.828 30.447 1.00 0.00 C
ATOM 41 O TRP 5 24.091 26.781 30.816 1.00 0.00 O
ATOM 42 N GLN 6 24.172 28.762 29.668 1.00 0.00 N
ATOM 43 CA GLN 6 22.885 28.523 28.973 1.00 0.00 C
ATOM 44 CB GLN 6 22.498 29.852 28.287 1.00 0.00 C
ATOM 45 CG GLN 6 22.587 31.121 29.099 1.00 0.00 C
ATOM 46 CD GLN 6 22.412 32.291 28.172 1.00 0.00 C
ATOM 47 OE1 GLN 6 23.316 32.556 27.384 1.00 0.00 O
ATOM 48 NE2 GLN 6 21.203 32.738 28.006 1.00 0.00 N
ATOM 49 C GLN 6 22.814 27.395 27.871 1.00 0.00 C
ATOM 50 O GLN 6 21.666 27.039 27.608 1.00 0.00 O
ATOM 51 N GLU 7 23.965 26.797 27.497 1.00 0.00 N
ATOM 52 CA GLU 7 23.863 25.708 26.404 1.00 0.00 C
ATOM 53 CB GLU 7 25.123 25.744 25.455 1.00 0.00 C
ATOM 54 CG GLU 7 25.083 27.024 24.556 1.00 0.00 C
ATOM 55 CD GLU 7 26.410 27.203 23.864 1.00 0.00 C
ATOM 56 OE1 GLU 7 27.263 27.882 24.478 1.00 0.00 O
ATOM 57 OE2 GLU 7 26.527 26.829 22.708 1.00 0.00 O1-
ATOM 58 C GLU 7 23.726 24.335 27.089 1.00 0.00 C
ATOM 59 O GLU 7 23.169 23.430 26.517 1.00 0.00 O
ATOM 60 N LEU 8 24.224 24.139 28.346 1.00 0.00 N
ATOM 61 CA LEU 8 23.976 22.903 29.089 1.00 0.00 C
ATOM 62 CB LEU 8 24.497 23.062 30.515 1.00 0.00 C
ATOM 63 CG LEU 8 26.076 23.131 30.667 1.00 0.00 C
ATOM 64 CD1 LEU 8 26.270 22.916 32.210 1.00 0.00 C
ATOM 65 CD2 LEU 8 26.762 21.929 30.005 1.00 0.00 C
ATOM 66 C LEU 8 22.526 22.391 29.168 1.00 0.00 C
ATOM 67 O LEU 8 22.319 21.195 29.108 1.00 0.00 O
ATOM 68 N TYR 9 21.535 23.194 29.197 1.00 0.00 N
ATOM 69 CA TYR 9 20.129 22.872 29.380 1.00 0.00 C
ATOM 70 CB TYR 9 19.533 23.856 30.394 1.00 0.00 C
ATOM 71 CG TYR 9 20.105 23.674 31.790 1.00 0.00 C
ATOM 72 CD1 TYR 9 19.691 22.512 32.511 1.00 0.00 C
ATOM 73 CE1 TYR 9 20.159 22.268 33.858 1.00 0.00 C
ATOM 74 CZ TYR 9 21.124 23.171 34.354 1.00 0.00 C
ATOM 75 OH TYR 9 21.570 23.102 35.677 1.00 0.00 O
ATOM 76 CE2 TYR 9 21.672 24.205 33.572 1.00 0.00 C
ATOM 77 CD2 TYR 9 21.107 24.533 32.340 1.00 0.00 C
ATOM 78 C TYR 9 19.399 22.689 28.048 1.00 0.00 C
ATOM 79 O TYR 9 18.208 22.392 28.037 1.00 0.00 O
ATOM 80 N GLN 10 20.046 22.820 26.873 1.00 0.00 N
ATOM 81 CA GLN 10 19.725 22.145 25.615 1.00 0.00 C
ATOM 82 CB GLN 10 19.687 23.127 24.450 1.00 0.00 C
ATOM 83 CG GLN 10 18.307 23.884 24.531 1.00 0.00 C
ATOM 84 CD GLN 10 18.061 24.905 23.403 1.00 0.00 C
ATOM 85 OE1 GLN 10 18.436 24.799 22.242 1.00 0.00 O
ATOM 86 NE2 GLN 10 17.273 25.924 23.717 1.00 0.00 N
ATOM 87 C GLN 10 20.656 20.884 25.312 1.00 0.00 C
ATOM 88 O GLN 10 20.165 19.845 24.826 1.00 0.00 O
ATOM 89 N LEU 11 21.923 20.962 25.703 1.00 0.00 N
ATOM 90 CA LEU 11 22.962 19.918 25.650 1.00 0.00 C
ATOM 91 CB LEU 11 24.332 20.488 26.010 1.00 0.00 C
ATOM 92 CG LEU 11 25.533 19.676 25.462 1.00 0.00 C
ATOM 93 CD1 LEU 11 25.720 19.993 23.943 1.00 0.00 C
ATOM 94 CD2 LEU 11 26.969 19.826 26.081 1.00 0.00 C
ATOM 95 C LEU 11 22.673 18.635 26.492 1.00 0.00 C
ATOM 96 O LEU 11 22.929 17.594 26.006 1.00 0.00 O
ATOM 97 N LYS 12 22.036 18.756 27.632 1.00 0.00 N
ATOM 98 CA LYS 12 21.665 17.559 28.524 1.00 0.00 C
ATOM 99 CB LYS 12 21.353 18.149 29.969 1.00 0.00 C
ATOM 100 CG LYS 12 21.380 17.101 31.048 1.00 0.00 C
ATOM 101 CD LYS 12 21.158 17.717 32.393 1.00 0.00 C
ATOM 102 CE LYS 12 21.488 16.864 33.631 1.00 0.00 C
ATOM 103 NZ LYS 12 20.608 15.707 33.921 1.00 0.00 N1+
ATOM 104 C LYS 12 20.617 16.610 27.890 1.00 0.00 C
ATOM 105 O LYS 12 20.562 15.449 28.258 1.00 0.00 O
ATOM 106 N TYR 13 19.785 17.090 26.935 1.00 0.00 N
ATOM 107 CA TYR 13 18.585 16.440 26.332 1.00 0.00 C
ATOM 108 CB TYR 13 17.327 17.308 26.571 1.00 0.00 C
ATOM 109 CG TYR 13 16.976 17.423 28.029 1.00 0.00 C
ATOM 110 CD1 TYR 13 16.988 18.661 28.705 1.00 0.00 C
ATOM 111 CE1 TYR 13 16.699 18.801 30.061 1.00 0.00 C
ATOM 112 CZ TYR 13 16.417 17.628 30.792 1.00 0.00 C
ATOM 113 OH TYR 13 16.188 17.721 32.137 1.00 0.00 O
ATOM 114 CE2 TYR 13 16.410 16.340 30.155 1.00 0.00 C
ATOM 115 CD2 TYR 13 16.697 16.268 28.734 1.00 0.00 C
ATOM 116 C TYR 13 18.859 16.159 24.831 1.00 0.00 C
ATOM 117 O TYR 13 18.509 15.087 24.317 1.00 0.00 O
ATOM 118 N LYS 14 19.424 17.062 24.051 1.00 0.00 N
ATOM 119 CA LYS 14 19.886 16.800 22.689 1.00 0.00 C
ATOM 120 CB LYS 14 20.306 18.148 22.094 1.00 0.00 C
ATOM 121 CG LYS 14 20.753 18.075 20.557 1.00 0.00 C
ATOM 122 CD LYS 14 21.616 19.231 20.084 1.00 0.00 C
ATOM 123 CE LYS 14 20.796 20.538 20.141 1.00 0.00 C
ATOM 124 NZ LYS 14 21.556 21.656 19.644 1.00 0.00 N1+
ATOM 125 C LYS 14 20.837 15.685 22.495 1.00 0.00 C
ATOM 126 O LYS 14 20.658 14.937 21.495 1.00 0.00 O
ATOM 127 N GLY 15 21.813 15.564 23.421 1.00 0.00 N
ATOM 128 CA GLY 15 23.008 14.810 23.424 1.00 0.00 C
ATOM 129 C GLY 15 24.087 15.227 22.415 1.00 0.00 C
ATOM 130 O GLY 15 23.803 15.951 21.463 1.00 0.00 O
ATOM 131 N ILE 16 25.315 14.697 22.628 1.00 0.00 N
ATOM 132 CA ILE 16 26.475 15.229 21.913 1.00 0.00 C
ATOM 133 CB ILE 16 27.655 14.961 22.930 1.00 0.00 C
ATOM 134 CG2 ILE 16 27.542 15.868 24.167 1.00 0.00 C
ATOM 135 CG1 ILE 16 28.106 13.472 23.104 1.00 0.00 C
ATOM 136 CD1 ILE 16 29.241 13.161 24.170 1.00 0.00 C
ATOM 137 C ILE 16 26.685 14.560 20.547 1.00 0.00 C
ATOM 138 O ILE 16 26.046 13.594 20.109 1.00 0.00 O
ATOM 139 N NHE 17 27.545 15.187 19.756 1.00 0.00 N
TER 140 NHE 17
```

H1 misfolded

```
ATOM  1  CH3 ACE  1  16.354  36.122  18.628  1.00  0.00  C
ATOM  2  C  ACE  1  16.263  34.604  18.876  1.00  0.00  C
ATOM  3  O  ACE  1  15.147  34.127  19.105  1.00  0.00  O
ATOM  4  N  LYS  2  17.384  33.844  18.658  1.00  0.00  N
ATOM  5  CA  LYS  2  17.445  32.376  18.946  1.00  0.00  C
ATOM  6  CB  LYS  2  17.580  31.658  17.542  1.00  0.00  C
ATOM  7  CG  LYS  2  17.449  30.116  17.547  1.00  0.00  C
ATOM  8  CD  LYS  2  17.430  29.435  16.187  1.00  0.00  C
ATOM  9  CE  LYS  2  18.808  29.378  15.551  1.00  0.00  C
ATOM  10  NZ  LYS  2  19.789  28.389  16.064  1.00  0.00  N1+
ATOM  11  C  LYS  2  18.653  32.048  19.804  1.00  0.00  C
ATOM  12  O  LYS  2  19.602  32.839  19.852  1.00  0.00  O
ATOM  13  N  LEU  3  18.656  30.926  20.485  1.00  0.00  N
ATOM  14  CA  LEU  3  19.738  30.456  21.339  1.00  0.00  C
ATOM  15  CB  LEU  3  19.199  29.293  22.198  1.00  0.00  C
ATOM  16  CG  LEU  3  20.199  28.788  23.213  1.00  0.00  C
ATOM  17  CD1 LEU  3  20.671  29.787  24.256  1.00  0.00  C
ATOM  18  CD2 LEU  3  19.597  27.635  23.974  1.00  0.00  C
ATOM  19  C  LEU  3  20.969  29.933  20.524  1.00  0.00  C
ATOM  20  O  LEU  3  20.817  29.097  19.630  1.00  0.00  O
ATOM  21  N  THR  4  22.165  30.418  20.816  1.00  0.00  N
ATOM  22  CA  THR  4  23.411  29.913  20.259  1.00  0.00  C
ATOM  23  CB  THR  4  24.020  30.965  19.267  1.00  0.00  C
ATOM  24  CG2 THR  4  23.177  31.206  18.019  1.00  0.00  C
ATOM  25  OG1 THR  4  24.339  32.238  19.851  1.00  0.00  O
ATOM  26  C  THR  4  24.423  29.597  21.337  1.00  0.00  C
ATOM  27  O  THR  4  24.617  30.282  22.322  1.00  0.00  O
ATOM  28  N  TRP  5  25.125  28.410  21.206  1.00  0.00  N
ATOM  29  CA  TRP  5  26.331  28.005  21.913  1.00  0.00  C
ATOM  30  CB  TRP  5  26.751  26.684  21.191  1.00  0.00  C
ATOM  31  CG  TRP  5  27.720  25.815  21.941  1.00  0.00  C
ATOM  32  CD1 TRP  5  27.434  24.660  22.583  1.00  0.00  C
ATOM  33  NE1 TRP  5  28.644  24.052  22.902  1.00  0.00  N
ATOM  34  CE2 TRP  5  29.690  24.831  22.556  1.00  0.00  C
ATOM  35  CZ2 TRP  5  31.104  24.602  22.607  1.00  0.00  C
ATOM  36  CH2 TRP  5  32.026  25.566  22.177  1.00  0.00  C
ATOM  37  CZ3 TRP  5  31.458  26.573  21.401  1.00  0.00  C
ATOM  38  CE3 TRP  5  30.038  26.820  21.340  1.00  0.00  C
ATOM  39  CD2 TRP  5  29.130  25.972  21.935  1.00  0.00  C
ATOM  40  C  TRP  5  27.411  29.119  21.982  1.00  0.00  C
ATOM  41  O  TRP  5  27.613  29.785  20.985  1.00  0.00  O
ATOM  42  N  GLN  6  28.125  29.253  23.112  1.00  0.00  N
ATOM  43  CA  GLN  6  29.264  30.128  23.304  1.00  0.00  C
ATOM  44  CB  GLN  6  28.911  31.119  24.400  1.00  0.00  C
ATOM  45  CG  GLN  6  29.950  32.195  24.595  1.00  0.00  C
ATOM  46  CD  GLN  6  29.691  33.005  25.773  1.00  0.00  C
ATOM  47  OE1 GLN  6  28.703  33.743  25.843  1.00  0.00  O
ATOM  48  NE2 GLN  6  30.555  32.922  26.764  1.00  0.00  N
ATOM  49  C  GLN  6  30.613  29.338  23.521  1.00  0.00  C
ATOM  50  O  GLN  6  31.478  29.475  22.658  1.00  0.00  O
ATOM  51  N  GLU  7  30.757  28.662  24.625  1.00  0.00  N
ATOM  52  CA  GLU  7  31.953  27.969  25.143  1.00  0.00  C
ATOM  53  CB  GLU  7  32.888  28.982  25.857  1.00  0.00  C
ATOM  54  CG  GLU  7  32.170  29.773  26.962  1.00  0.00  C
ATOM  55  CD  GLU  7  33.142  30.587  27.760  1.00  0.00  C
ATOM  56  OE1 GLU  7  33.329  30.175  28.912  1.00  0.00  O
ATOM  57  OE2 GLU  7  33.675  31.717  27.415  1.00  0.00  O1-
ATOM  58  C  GLU  7  31.759  26.791  26.071  1.00  0.00  C
ATOM  59  O  GLU  7  30.680  26.654  26.730  1.00  0.00  O
ATOM  60  N  LEU  8  32.760  25.962  26.276  1.00  0.00  N
ATOM  61  CA  LEU  8  32.834  24.854  27.255  1.00  0.00  C
ATOM  62  CB  LEU  8  33.878  23.833  26.748  1.00  0.00  C
ATOM  63  CG  LEU  8  33.968  22.503  27.516  1.00  0.00  C
ATOM  64  CD1 LEU  8  32.698  21.763  27.089  1.00  0.00  C
ATOM  65  CD2 LEU  8  35.241  21.739  27.005  1.00  0.00  C
ATOM  66  C  LEU  8  33.128  25.391  28.682  1.00  0.00  C
ATOM  67  O  LEU  8  34.027  26.204  28.867  1.00  0.00  O
ATOM  68  N  TYR  9  32.337  24.905  29.664  1.00  0.00  N
ATOM  69  CA  TYR  9  32.409  25.298  31.083  1.00  0.00  C
ATOM  70  CB  TYR  9  31.238  26.248  31.411  1.00  0.00  C
ATOM  71  CG  TYR  9  31.119  26.892  32.747  1.00  0.00  C
ATOM  72  CD1 TYR  9  29.916  26.753  33.379  1.00  0.00  C
ATOM  73  CE1 TYR  9  29.654  27.315  34.658  1.00  0.00  C
ATOM  74  CZ  TYR  9  30.677  27.931  35.368  1.00  0.00  C
ATOM  75  OH  TYR  9  30.354  28.439  36.610  1.00  0.00  O
ATOM  76  CE2 TYR  9  31.996  28.002  34.755  1.00  0.00  C
ATOM  77  CD2 TYR  9  32.199  27.525  33.439  1.00  0.00  C
ATOM  78  C  TYR  9  32.306  24.033  32.000  1.00  0.00  C
ATOM  79  O  TYR  9  32.253  22.911  31.478  1.00  0.00  O
ATOM  80  N  GLN  10  32.485  24.175  33.348  1.00  0.00  N
ATOM  81  CA  GLN  10  32.258  23.036  34.253  1.00  0.00  C
ATOM  82  CB  GLN  10  32.435  23.560  35.763  1.00  0.00  C
ATOM  83  CG  GLN  10  33.890  23.714  36.151  1.00  0.00  C
ATOM  84  CD  GLN  10  34.333  24.030  37.528  1.00  0.00  C
ATOM  85  OE1 GLN  10  34.381  25.161  37.990  1.00  0.00  O
ATOM  86  NE2 GLN  10  34.681  23.002  38.314  1.00  0.00  N
ATOM  87  C  GLN  10  30.837  22.455  33.989  1.00  0.00  C
ATOM  88  O  GLN  10  30.649  21.279  33.725  1.00  0.00  O
ATOM  89  N  LEU  11  29.829  23.359  34.103  1.00  0.00  N
ATOM  90  CA  LEU  11  28.428  22.992  33.843  1.00  0.00  C
ATOM  91  CB  LEU  11  27.492  23.898  34.670  1.00  0.00  C
ATOM  92  CG  LEU  11  25.985  23.718  34.684  1.00  0.00  C
ATOM  93  CD1 LEU  11  25.568  22.310  34.970  1.00  0.00  C
ATOM  94  CD2 LEU  11  25.306  24.595  35.690  1.00  0.00  C
ATOM  95  C  LEU  11  28.164  23.054  32.294  1.00  0.00  C
ATOM  96  O  LEU  11  27.909  24.141  31.797  1.00  0.00  O
ATOM  97  N  LYS  12  28.264  21.849  31.652  1.00  0.00  N
ATOM  98  CA  LYS  12  28.120  21.651  30.182  1.00  0.00  C
ATOM  99  CB  LYS  12  26.654  21.295  29.829  1.00  0.00  C
ATOM  100  CG  LYS  12  26.165  19.896  30.240  1.00  0.00  C
ATOM  101  CD  LYS  12  24.707  19.636  29.852  1.00  0.00  C
ATOM  102  CE  LYS  12  24.010  18.371  30.344  1.00  0.00  C
ATOM  103  NZ  LYS  12  22.795  18.040  29.592  1.00  0.00  N1+
ATOM  104  C  LYS  12  28.711  22.848  29.383  1.00  0.00  C
ATOM  105  O  LYS  12  29.926  22.936  29.224  1.00  0.00  O
ATOM  106  N  TYR  13  27.891  23.805  28.925  1.00  0.00  N
ATOM  107  CA  TYR  13  28.384  24.923  28.093  1.00  0.00  C
ATOM  108  CB  TYR  13  28.671  24.456  26.691  1.00  0.00  C
ATOM  109  CG  TYR  13  27.807  23.477  25.978  1.00  0.00  C
ATOM  110  CD1 TYR  13  28.184  22.091  25.961  1.00  0.00  C
ATOM  111  CE1 TYR  13  27.233  21.176  25.501  1.00  0.00  C
ATOM  112  CZ  TYR  13  25.968  21.526  25.043  1.00  0.00  C
ATOM  113  OH  TYR  13  25.162  20.527  24.522  1.00  0.00  O
ATOM  114  CE2 TYR  13  25.585  22.903  25.018  1.00  0.00  C
ATOM  115  CD2 TYR  13  26.529  23.857  25.484  1.00  0.00  C
ATOM  116  C  TYR  13  27.393  26.121  28.215  1.00  0.00  C
ATOM  117  O  TYR  13  26.214  26.002  28.553  1.00  0.00  O
ATOM  118  N  LYS  14  27.904  27.356  28.071  1.00  0.00  N
ATOM  119  CA  LYS  14  26.982  28.537  28.065  1.00  0.00  C
ATOM  120  CB  LYS  14  27.938  29.703  28.410  1.00  0.00  C
ATOM  121  CG  LYS  14  28.747  29.455  29.640  1.00  0.00  C
ATOM  122  CD  LYS  14  29.582  30.620  30.027  1.00  0.00  C
ATOM  123  CE  LYS  14  30.438  30.213  31.262  1.00  0.00  C
ATOM  124  NZ  LYS  14  31.220  31.359  31.752  1.00  0.00  N1+
ATOM  125  C  LYS  14  26.453  28.790  26.707  1.00  0.00  C
ATOM  126  O  LYS  14  27.148  28.429  25.769  1.00  0.00  O
ATOM  127  N  GLY  15  25.394  29.560  26.658  1.00  0.00  N
ATOM  128  CA  GLY  15  24.767  30.035  25.457  1.00  0.00  C
ATOM  129  C  GLY  15  24.173  31.476  25.662  1.00  0.00  C
ATOM  130  O  GLY  15  23.902  31.906  26.797  1.00  0.00  O
ATOM  131  N  ILE  16  24.074  32.207  24.582  1.00  0.00  N
ATOM  132  CA  ILE  16  23.423  33.582  24.512  1.00  0.00  C
ATOM  133  CB  ILE  16  24.437  34.691  24.501  1.00  0.00  C
ATOM  134  CG2 ILE  16  25.291  34.921  25.774  1.00  0.00  C
ATOM  135  CG1 ILE  16  25.175  34.743  23.107  1.00  0.00  C
ATOM  136  CD1 ILE  16  26.340  33.834  22.974  1.00  0.00  C
ATOM  137  C  ILE  16  22.341  33.521  23.427  1.00  0.00  C
ATOM  138  O  ILE  16  22.400  32.859  22.373  1.00  0.00  O
ATOM  139  N  NHE  17  21.219  34.128  23.683  1.00  0.00  N
TER  140    NHE  17
```

H2 extended

ATOM 1 CH3 ACE 1 18.616 22.973 24.268 1.00 0.00 C
ATOM 2 C ACE 1 17.319 22.718 23.497 1.00 0.00 C
ATOM 3 O ACE 1 16.325 22.950 24.135 1.00 0.00 O
ATOM 4 N ALA 2 17.399 22.348 22.178 1.00 0.00 N
ATOM 5 CA ALA 2 16.245 21.977 21.352 1.00 0.00 C
ATOM 6 CB ALA 2 16.376 22.769 20.037 1.00 0.00 C
ATOM 7 C ALA 2 15.956 20.463 21.131 1.00 0.00 C
ATOM 8 O ALA 2 15.127 20.176 20.242 1.00 0.00 O
ATOM 9 N AIB 3 16.717 19.554 21.753 1.00 0.00 N
ATOM 10 CA AIB 3 16.899 18.097 21.482 1.00 0.00 C
ATOM 11 CB1 AIB 3 15.605 17.462 21.843 1.00 0.00 C
ATOM 12 CB2 AIB 3 18.007 17.657 22.449 1.00 0.00 C
ATOM 13 C AIB 3 17.317 17.864 19.972 1.00 0.00 C
ATOM 14 O AIB 3 16.905 16.856 19.354 1.00 0.00 O
ATOM 15 N ALA 4 18.206 18.694 19.391 1.00 0.00 N
ATOM 16 CA ALA 4 18.691 18.534 18.024 1.00 0.00 C
ATOM 17 CB ALA 4 19.360 19.880 17.652 1.00 0.00 C
ATOM 18 C ALA 4 19.708 17.388 17.776 1.00 0.00 C
ATOM 19 O ALA 4 20.542 16.977 18.623 1.00 0.00 O
ATOM 20 N AIB 5 19.591 16.819 16.543 1.00 0.00 N
ATOM 21 CA AIB 5 20.489 15.789 16.006 1.00 0.00 C
ATOM 22 CB1 AIB 5 19.981 15.663 14.550 1.00 0.00 C
ATOM 23 CB2 AIB 5 20.289 14.477 16.723 1.00 0.00 C
ATOM 24 C AIB 5 22.000 16.237 15.973 1.00 0.00 C
ATOM 25 O AIB 5 22.919 15.409 16.045 1.00 0.00 O
ATOM 26 N AIB 6 22.241 17.605 16.061 1.00 0.00 N
ATOM 27 CA AIB 6 23.433 18.358 15.575 1.00 0.00 C
ATOM 28 CB1 AIB 6 23.313 19.835 15.991 1.00 0.00 C
ATOM 29 CB2 AIB 6 23.394 18.398 14.087 1.00 0.00 C
ATOM 30 C AIB 6 24.801 17.776 16.113 1.00 0.00 C
ATOM 31 O AIB 6 25.888 17.815 15.456 1.00 0.00 O
ATOM 32 N NHE 7 24.805 17.527 17.371 1.00 0.00 N
TER 33 NHE 7

H2 misfolded

ATOM 1 CH3 ACE 1 15.700 20.901 23.583 1.00 0.00 C
ATOM 2 C ACE 1 15.782 20.376 22.147 1.00 0.00 C
ATOM 3 O ACE 1 14.816 19.818 21.631 1.00 0.00 O
ATOM 4 N ALA 2 16.941 20.556 21.510 1.00 0.00 N
ATOM 5 CA ALA 2 17.145 20.104 20.149 1.00 0.00 C
ATOM 6 CB ALA 2 16.156 20.768 19.196 1.00 0.00 C
ATOM 7 C ALA 2 18.549 20.437 19.667 1.00 0.00 C
ATOM 8 O ALA 2 19.343 21.015 20.405 1.00 0.00 O
ATOM 9 N AIB 3 18.856 20.067 18.421 1.00 0.00 N
ATOM 10 CA AIB 3 20.177 20.329 17.837 1.00 0.00 C
ATOM 11 CB1 AIB 3 21.195 19.529 18.664 1.00 0.00 C
ATOM 12 CB2 AIB 3 20.522 21.820 17.879 1.00 0.00 C
ATOM 13 C AIB 3 20.284 19.808 16.392 1.00 0.00 C
ATOM 14 O AIB 3 19.346 19.263 15.879 1.00 0.00 O
ATOM 15 N ALA 4 21.451 20.000 15.773 1.00 0.00 N
ATOM 16 CA ALA 4 21.679 19.556 14.412 1.00 0.00 C
ATOM 17 CB ALA 4 23.091 19.900 13.952 1.00 0.00 C
ATOM 18 C ALA 4 21.504 18.050 14.289 1.00 0.00 C
ATOM 19 O ALA 4 21.114 17.551 13.237 1.00 0.00 O
ATOM 20 N AIB 5 21.794 17.325 15.372 1.00 0.00 N
ATOM 21 CA AIB 5 21.667 15.863 15.383 1.00 0.00 C
ATOM 22 CB1 AIB 5 22.653 15.337 16.437 1.00 0.00 C
ATOM 23 CB2 AIB 5 21.995 15.261 14.012 1.00 0.00 C
ATOM 24 C AIB 5 20.265 15.408 15.830 1.00 0.00 C
ATOM 25 O AIB 5 20.004 14.237 15.890 1.00 0.00 O
ATOM 26 N AIB 6 19.392 16.371 16.135 1.00 0.00 N
ATOM 27 CA AIB 6 18.027 16.068 16.575 1.00 0.00 C
ATOM 28 CB1 AIB 6 18.143 15.314 17.908 1.00 0.00 C
ATOM 29 CB2 AIB 6 17.285 15.206 15.548 1.00 0.00 C
ATOM 30 C AIB 6 17.215 17.345 16.866 1.00 0.00 C
ATOM 31 O AIB 6 17.723 18.425 16.723 1.00 0.00 O
ATOM 32 N NHE 7 15.956 17.174 17.274 1.00 0.00 N
TER 33 NHE 7

B1 extended

```
ATOM  1 CH3 ACE 1  19.051 33.832 35.759 1.00 0.00  C
ATOM  2 C   ACE 1  18.351 33.143 34.583 1.00 0.00  C
ATOM  3 O   ACE 1  17.199 33.400 34.331 1.00 0.00  O
ATOM  4 N   GLY 2  19.106 32.321 33.780 1.00 0.00  N
ATOM  5 CA  GLY 2  18.554 31.853 32.435 1.00 0.00  C
ATOM  6 C   GLY 2  19.280 30.641 31.839 1.00 0.00  C
ATOM  7 O   GLY 2  20.329 30.069 32.455 1.00 0.00  O
ATOM  8 N   GLU 3  18.690 30.057 30.782 1.00 0.00  N
ATOM  9 CA  GLU 3  19.126 28.740 30.137 1.00 0.00  C
ATOM 10 CB  GLU 3  18.047 28.303 29.011 1.00 0.00  C
ATOM 11 CG  GLU 3  18.009 26.878 28.481 1.00 0.00  C
ATOM 12 CD  GLU 3  17.686 25.810 29.566 1.00 0.00  C
ATOM 13 OE1 GLU 3  16.590 25.685 30.118 1.00 0.00  O
ATOM 14 OE2 GLU 3  18.584 25.028 29.955 1.00 0.00  O1-
ATOM 15 C   GLU 3  20.497 28.844 29.597 1.00 0.00  C
ATOM 16 O   GLU 3  21.281 27.899 29.667 1.00 0.00  O
ATOM 17 N   TRP 4  20.946 30.045 29.171 1.00 0.00  N
ATOM 18 CA  TRP 4  22.256 30.415 28.703 1.00 0.00  C
ATOM 19 CB  TRP 4  22.213 31.929 28.355 1.00 0.00  C
ATOM 20 CG  TRP 4  22.087 33.014 29.361 1.00 0.00  C
ATOM 21 CD1 TRP 4  20.943 33.623 29.746 1.00 0.00  C
ATOM 22 NE1 TRP 4  21.214 34.587 30.722 1.00 0.00  N
ATOM 23 CE2 TRP 4  22.585 34.527 31.053 1.00 0.00  C
ATOM 24 CZ2 TRP 4  23.323 35.266 31.971 1.00 0.00  C
ATOM 25 CH2 TRP 4  24.682 34.964 32.135 1.00 0.00  C
ATOM 26 CZ3 TRP 4  25.247 34.001 31.319 1.00 0.00  C
ATOM 27 CE3 TRP 4  24.509 33.342 30.352 1.00 0.00  C
ATOM 28 CD2 TRP 4  23.163 33.567 30.154 1.00 0.00  C
ATOM 29 C   TRP 4  23.425 30.165 29.661 1.00 0.00  C
ATOM 30 O   TRP 4  24.549 30.036 29.213 1.00 0.00  O
ATOM 31 N   THR 5  23.125 30.021 30.981 1.00 0.00  N
ATOM 32 CA  THR 5  24.124 29.538 31.953 1.00 0.00  C
ATOM 33 CB  THR 5  23.654 29.541 33.388 1.00 0.00  C
ATOM 34 CG2 THR 5  23.335 30.915 33.938 1.00 0.00  C
ATOM 35 OG1 THR 5  22.552 28.629 33.555 1.00 0.00  O
ATOM 36 C   THR 5  24.614 28.160 31.639 1.00 0.00  C
ATOM 37 O   THR 5  25.730 27.827 31.944 1.00 0.00  O
ATOM 38 N   TYR 6  23.740 27.260 31.121 1.00 0.00  N
ATOM 39 CA  TYR 6  23.954 25.794 30.892 1.00 0.00  C
ATOM 40 CB  TYR 6  22.707 25.285 31.624 1.00 0.00  C
ATOM 41 CG  TYR 6  22.579 23.755 31.605 1.00 0.00  C
ATOM 42 CD1 TYR 6  23.684 23.029 32.097 1.00 0.00  C
ATOM 43 CE1 TYR 6  23.689 21.585 32.093 1.00 0.00  C
ATOM 44 CZ  TYR 6  22.523 20.909 31.654 1.00 0.00  C
ATOM 45 OH  TYR 6  22.468 19.548 31.777 1.00 0.00  O
ATOM 46 CE2 TYR 6  21.481 21.605 30.960 1.00 0.00  C
ATOM 47 CD2 TYR 6  21.520 23.022 30.975 1.00 0.00  C
ATOM 48 C   TYR 6  24.133 25.466 29.432 1.00 0.00  C
ATOM 49 O   TYR 6  24.879 24.521 29.187 1.00 0.00  O
ATOM 50 N   ASP 7  23.417 26.167 28.598 1.00 0.00  N
ATOM 51 CA  ASP 7  23.338 25.843 27.163 1.00 0.00  C
ATOM 52 CB  ASP 7  21.940 25.171 26.854 1.00 0.00  C
ATOM 53 CG  ASP 7  21.548 25.223 25.399 1.00 0.00  C
ATOM 54 OD1 ASP 7  22.373 25.078 24.471 1.00 0.00  O
ATOM 55 OD2 ASP 7  20.374 25.349 25.132 1.00 0.00  O1-
ATOM 56 C   ASP 7  23.529 27.094 26.312 1.00 0.00  C
ATOM 57 O   ASP 7  22.675 28.010 26.238 1.00 0.00  O
ATOM 58 N   ASP 8  24.683 27.127 25.632 1.00 0.00  N
ATOM 59 CA  ASP 8  25.117 28.249 24.756 1.00 0.00  C
ATOM 60 CB  ASP 8  25.875 29.350 25.614 1.00 0.00  C
ATOM 61 CG  ASP 8  26.075 30.700 24.967 1.00 0.00  C
ATOM 62 OD1 ASP 8  27.201 31.229 24.868 1.00 0.00  O
ATOM 63 OD2 ASP 8  25.100 31.257 24.394 1.00 0.00  O1-
ATOM 64 C   ASP 8  26.002 27.814 23.563 1.00 0.00  C
ATOM 65 O   ASP 8  26.288 26.603 23.390 1.00 0.00  O
ATOM 66 N   ALA 9  25.997 28.614 22.567 1.00 0.00  N
ATOM 67 CA  ALA 9  26.384 28.249 21.202 1.00 0.00  C
ATOM 68 CB  ALA 9  26.161 29.489 20.276 1.00 0.00  C
ATOM 69 C   ALA 9  27.813 27.627 21.049 1.00 0.00  C
ATOM 70 O   ALA 9  28.015 27.005 20.004 1.00 0.00  O
ATOM 71 N   THR 10  28.785 27.822 21.929 1.00 0.00  N
ATOM 72 CA  THR 10  30.201 27.331 21.932 1.00 0.00  C
ATOM 73 CB  THR 10  31.107 28.545 22.201 1.00 0.00  C
ATOM 74 CG2 THR 10  31.067 29.466 20.995 1.00 0.00  C
ATOM 75 OG1 THR 10  30.744 29.178 23.434 1.00 0.00  O
ATOM 76 C   THR 10  30.575 26.197 22.908 1.00 0.00  C
ATOM 77 O   THR 10  31.581 25.536 22.765 1.00 0.00  O
ATOM 78 N   LYS 11  29.803 26.124 23.991 1.00 0.00  N
ATOM 79 CA  LYS 11  29.803 25.082 25.051 1.00 0.00  C
ATOM 80 CB  LYS 11  29.587 25.679 26.406 1.00 0.00  C
ATOM 81 CG  LYS 11  28.132 26.163 26.713 1.00 0.00  C
ATOM 82 CD  LYS 11  28.163 27.048 28.012 1.00 0.00  C
ATOM 83 CE  LYS 11  27.899 26.242 29.290 1.00 0.00  C
ATOM 84 NZ  LYS 11  28.258 27.060 30.484 1.00 0.00  N1+
ATOM 85 C   LYS 11  28.894 23.843 24.695 1.00 0.00  C
ATOM 86 O   LYS 11  29.318 22.686 24.988 1.00 0.00  O
ATOM 87 N   THR 12  27.840 24.038 23.860 1.00 0.00  N
ATOM 88 CA  THR 12  26.937 22.902 23.511 1.00 0.00  C
ATOM 89 CB  THR 12  25.375 23.288 23.476 1.00 0.00  C
ATOM 90 CG2 THR 12  24.509 22.023 23.483 1.00 0.00  C
ATOM 91 OG1 THR 12  24.997 24.039 24.634 1.00 0.00  O
ATOM 92 C   THR 12  27.465 22.206 22.275 1.00 0.00  C
ATOM 93 O   THR 12  27.541 20.981 22.228 1.00 0.00  O
ATOM 94 N   PHE 13  27.825 23.003 21.238 1.00 0.00  N
ATOM 95 CA  PHE 13  28.243 22.461 19.934 1.00 0.00  C
ATOM 96 CB  PHE 13  27.479 23.052 18.752 1.00 0.00  C
ATOM 97 CG  PHE 13  25.943 23.070 18.974 1.00 0.00  C
ATOM 98 CD1 PHE 13  25.173 21.903 18.967 1.00 0.00  C
ATOM 99 CE1 PHE 13  23.816 21.888 19.347 1.00 0.00  C
ATOM 100 CZ  PHE 13  23.258 23.056 19.807 1.00 0.00  C
ATOM 101 CE2 PHE 13  24.030 24.246 19.861 1.00 0.00  C
ATOM 102 CD2 PHE 13  25.341 24.274 19.386 1.00 0.00  C
ATOM 103 C   PHE 13  29.733 22.448 19.943 1.00 0.00  C
ATOM 104 O   PHE 13  30.287 22.931 18.965 1.00 0.00  O
ATOM 105 N   THR 14  30.352 21.774 20.881 1.00 0.00  N
ATOM 106 CA  THR 14  31.807 21.539 20.841 1.00 0.00  C
ATOM 107 CB  THR 14  32.149 20.641 22.042 1.00 0.00  C
ATOM 108 CG2 THR 14  32.091 21.198 23.433 1.00 0.00  C
ATOM 109 OG1 THR 14  31.245 19.592 22.058 1.00 0.00  O
ATOM 110 C   THR 14  32.357 20.989 19.526 1.00 0.00  C
ATOM 111 O   THR 14  33.535 21.376 19.167 1.00 0.00  O
ATOM 112 N   VAL 15  31.533 20.290 18.722 1.00 0.00  N
ATOM 113 CA  VAL 15  31.728 19.848 17.317 1.00 0.00  C
ATOM 114 CB  VAL 15  30.522 19.069 16.832 1.00 0.00  C
ATOM 115 CG1 VAL 15  30.436 17.686 17.471 1.00 0.00  C
ATOM 116 CG2 VAL 15  29.121 19.824 16.926 1.00 0.00  C
ATOM 117 C   VAL 15  32.033 20.920 16.276 1.00 0.00  C
ATOM 118 O   VAL 15  32.582 20.641 15.194 1.00 0.00  O
ATOM 119 N   THR 16  31.735 22.215 16.615 1.00 0.00  N
ATOM 120 CA  THR 16  32.098 23.411 15.890 1.00 0.00  C
ATOM 121 CB  THR 16  31.202 24.626 16.233 1.00 0.00  C
ATOM 122 CG2 THR 16  29.748 24.342 15.915 1.00 0.00  C
ATOM 123 OG1 THR 16  31.298 24.973 17.570 1.00 0.00  O
ATOM 124 C   THR 16  33.628 23.793 15.976 1.00 0.00  C
ATOM 125 O   THR 16  34.049 24.807 15.396 1.00 0.00  O
ATOM 126 N   GLU 17  34.410 22.994 16.738 1.00 0.00  N
ATOM 127 CA  GLU 17  35.850 23.317 17.023 1.00 0.00  C
ATOM 128 CB  GLU 17  35.850 23.966 18.427 1.00 0.00  C
ATOM 129 CG  GLU 17  37.295 24.559 18.699 1.00 0.00  C
ATOM 130 CD  GLU 17  37.548 24.750 20.196 1.00 0.00  C
ATOM 131 OE1 GLU 17  36.652 25.322 20.914 1.00 0.00  O
ATOM 132 OE2 GLU 17  38.718 24.458 20.533 1.00 0.00  O1-
ATOM 133 C   GLU 17  36.611 21.936 16.972 1.00 0.00  C
ATOM 134 O   GLU 17  37.625 21.663 17.595 1.00 0.00  O
ATOM 135 N   NHE 18  36.172 21.042 16.100 1.00 0.00  N
TER 136  NHE 18
```

B1 misfolded

```
ATOM 1 CH3 ACE 1 30.243 19.667 35.960 1.00 0.00 C
ATOM 2 C ACE 1 30.138 21.124 35.507 1.00 0.00 C
ATOM 3 O ACE 1 30.051 22.030 36.334 1.00 0.00 O
ATOM 4 N GLY 2 30.148 21.343 34.212 1.00 0.00 N
ATOM 5 CA GLY 2 30.055 22.684 33.670 1.00 0.00 C
ATOM 6 C GLY 2 30.090 22.645 32.149 1.00 0.00 C
ATOM 7 O GLY 2 29.300 23.317 31.490 1.00 0.00 O
ATOM 8 N GLU 3 31.010 21.854 31.592 1.00 0.00 N
ATOM 9 CA GLU 3 31.144 21.730 30.155 1.00 0.00 C
ATOM 10 CB GLU 3 32.268 20.765 29.789 1.00 0.00 C
ATOM 11 CG GLU 3 32.375 20.664 28.287 1.00 0.00 C
ATOM 12 CD GLU 3 33.500 19.699 27.921 1.00 0.00 C
ATOM 13 OE1 GLU 3 33.195 18.483 27.789 1.00 0.00 O
ATOM 14 OE2 GLU 3 34.653 20.186 27.778 1.00 0.00 O1-
ATOM 15 C GLU 3 29.859 21.207 29.528 1.00 0.00 C
ATOM 16 O GLU 3 29.406 21.726 28.510 1.00 0.00 O
ATOM 17 N TRP 4 29.273 20.175 30.139 1.00 0.00 N
ATOM 18 CA TRP 4 28.047 19.586 29.640 1.00 0.00 C
ATOM 19 CB TRP 4 27.596 18.428 30.523 1.00 0.00 C
ATOM 20 CG TRP 4 26.321 17.748 30.082 1.00 0.00 C
ATOM 21 CD1 TRP 4 25.713 16.712 30.676 1.00 0.00 C
ATOM 22 NE1 TRP 4 24.545 16.414 29.904 1.00 0.00 N
ATOM 23 CE2 TRP 4 24.477 17.249 28.896 1.00 0.00 C
ATOM 24 CZ2 TRP 4 23.500 17.313 27.895 1.00 0.00 C
ATOM 25 CH2 TRP 4 23.673 18.305 26.938 1.00 0.00 C
ATOM 26 CZ3 TRP 4 24.728 19.144 26.989 1.00 0.00 C
ATOM 27 CE3 TRP 4 25.701 19.061 28.007 1.00 0.00 C
ATOM 28 CD2 TRP 4 25.526 18.062 28.972 1.00 0.00 C
ATOM 29 C TRP 4 26.921 20.611 29.606 1.00 0.00 C
ATOM 30 O TRP 4 26.188 20.699 28.624 1.00 0.00 O
ATOM 31 N THR 5 26.787 21.386 30.684 1.00 0.00 N
ATOM 32 CA THR 5 25.755 22.399 30.774 1.00 0.00 C
ATOM 33 CB THR 5 25.826 23.138 32.107 1.00 0.00 C
ATOM 34 CG2 THR 5 25.636 22.147 33.249 1.00 0.00 C
ATOM 35 OG1 THR 5 27.101 23.773 32.232 1.00 0.00 O
ATOM 36 C THR 5 25.899 23.429 29.663 1.00 0.00 C
ATOM 37 O THR 5 24.916 23.805 29.028 1.00 0.00 O
ATOM 38 N TYR 6 27.131 23.887 29.429 1.00 0.00 N
ATOM 39 CA TYR 6 27.400 24.869 28.397 1.00 0.00 C
ATOM 40 CB TYR 6 28.884 25.215 28.343 1.00 0.00 C
ATOM 41 CG TYR 6 29.122 26.239 27.259 1.00 0.00 C
ATOM 42 CD1 TYR 6 30.417 26.715 27.017 1.00 0.00 C
ATOM 43 CE1 TYR 6 30.637 27.665 26.012 1.00 0.00 C
ATOM 44 CZ TYR 6 29.564 28.138 25.249 1.00 0.00 C
ATOM 45 OH TYR 6 29.779 29.059 24.272 1.00 0.00 O
ATOM 46 CE2 TYR 6 28.270 27.661 25.490 1.00 0.00 C
ATOM 47 CD2 TYR 6 28.049 26.712 26.494 1.00 0.00 C
ATOM 48 C TYR 6 26.993 24.350 27.026 1.00 0.00 C
ATOM 49 O TYR 6 26.367 25.066 26.248 1.00 0.00 O
ATOM 50 N ASP 7 27.350 23.097 26.731 1.00 0.00 N
ATOM 51 CA ASP 7 27.021 22.487 25.458 1.00 0.00 C
ATOM 52 CB ASP 7 27.546 21.058 25.383 1.00 0.00 C
ATOM 53 CG ASP 7 27.180 20.452 24.030 1.00 0.00 C
ATOM 54 OD1 ASP 7 28.007 20.599 23.091 1.00 0.00 O
ATOM 55 OD2 ASP 7 26.078 19.847 23.949 1.00 0.00 O1-
ATOM 56 C ASP 7 25.515 22.442 25.245 1.00 0.00 C
ATOM 57 O ASP 7 25.028 22.766 24.164 1.00 0.00 O
ATOM 58 N ASP 8 24.777 22.038 26.281 1.00 0.00 N
ATOM 59 CA ASP 8 23.332 21.951 26.204 1.00 0.00 C
ATOM 60 CB ASP 8 22.740 21.464 27.522 1.00 0.00 C
ATOM 61 CG ASP 8 21.220 21.387 27.400 1.00 0.00 C
ATOM 62 OD1 ASP 8 20.729 20.298 26.999 1.00 0.00 O
ATOM 63 OD2 ASP 8 20.564 22.417 27.710 1.00 0.00 O1-
ATOM 64 C ASP 8 22.717 23.309 25.892 1.00 0.00 C
ATOM 65 O ASP 8 21.831 23.412 25.049 1.00 0.00 O
ATOM 66 N ALA 9 23.193 24.351 26.577 1.00 0.00 N
ATOM 67 CA ALA 9 22.691 25.695 26.371 1.00 0.00 C
ATOM 68 CB ALA 9 23.404 26.692 27.279 1.00 0.00 C
ATOM 69 C ALA 9 22.900 26.144 24.933 1.00 0.00 C
ATOM 70 O ALA 9 21.997 26.709 24.318 1.00 0.00 O
ATOM 71 N THR 10 24.096 25.891 24.395 1.00 0.00 N
ATOM 72 CA THR 10 24.418 26.269 23.034 1.00 0.00 C
ATOM 73 CB THR 10 25.843 25.864 22.674 1.00 0.00 C
ATOM 74 CG2 THR 10 26.821 26.557 23.617 1.00 0.00 C
ATOM 75 OG1 THR 10 25.982 24.446 22.801 1.00 0.00 O
ATOM 76 C THR 10 23.479 25.596 22.042 1.00 0.00 C
ATOM 77 O THR 10 22.982 26.239 21.119 1.00 0.00 O
ATOM 78 N LYS 11 23.238 24.298 22.233 1.00 0.00 N
ATOM 79 CA LYS 11 22.362 23.544 21.358 1.00 0.00 C
ATOM 80 CB LYS 11 22.263 22.089 21.802 1.00 0.00 C
ATOM 81 CG LYS 11 21.336 21.331 20.858 1.00 0.00 C
ATOM 82 CD LYS 11 21.236 19.876 21.302 1.00 0.00 C
ATOM 83 CE LYS 11 20.308 19.119 20.357 1.00 0.00 C
ATOM 84 NZ LYS 11 20.212 17.716 20.785 1.00 0.00 N1+
ATOM 85 C LYS 11 20.956 24.126 21.355 1.00 0.00 C
ATOM 86 O LYS 11 20.349 24.284 20.299 1.00 0.00 O
ATOM 87 N THR 12 20.440 24.447 22.543 1.00 0.00 N
ATOM 88 CA THR 12 19.112 25.010 22.674 1.00 0.00 C
ATOM 89 CB THR 12 18.771 25.281 24.136 1.00 0.00 C
ATOM 90 CG2 THR 12 18.828 23.975 24.922 1.00 0.00 C
ATOM 91 OG1 THR 12 19.715 26.206 24.681 1.00 0.00 O
ATOM 92 C THR 12 18.997 26.325 21.918 1.00 0.00 C
ATOM 93 O THR 12 18.018 26.555 21.211 1.00 0.00 O
ATOM 94 N PHE 13 20.002 27.191 22.068 1.00 0.00 N
ATOM 95 CA PHE 13 20.011 28.478 21.401 1.00 0.00 C
ATOM 96 CB PHE 13 21.271 29.265 21.745 1.00 0.00 C
ATOM 97 CG PHE 13 21.371 30.624 21.095 1.00 0.00 C
ATOM 98 CD1 PHE 13 22.485 31.436 21.338 1.00 0.00 C
ATOM 99 CE1 PHE 13 22.577 32.696 20.735 1.00 0.00 C
ATOM 100 CZ PHE 13 21.556 33.144 19.889 1.00 0.00 C
ATOM 101 CE2 PHE 13 20.442 32.332 19.645 1.00 0.00 C
ATOM 102 CD2 PHE 13 20.349 31.073 20.248 1.00 0.00 C
ATOM 103 C PHE 13 19.964 28.311 19.889 1.00 0.00 C
ATOM 104 O PHE 13 19.210 29.003 19.208 1.00 0.00 O
ATOM 105 N THR 14 20.775 27.388 19.364 1.00 0.00 N
ATOM 106 CA THR 14 20.823 27.134 17.938 1.00 0.00 C
ATOM 107 CB THR 14 21.828 26.036 17.611 1.00 0.00 C
ATOM 108 CG2 THR 14 23.216 26.460 18.078 1.00 0.00 C
ATOM 109 OG1 THR 14 21.448 24.829 18.277 1.00 0.00 O
ATOM 110 C THR 14 19.464 26.694 17.413 1.00 0.00 C
ATOM 111 O THR 14 19.011 27.171 16.375 1.00 0.00 O
ATOM 112 N VAL 15 18.812 25.779 18.136 1.00 0.00 N
ATOM 113 CA VAL 15 17.510 25.279 17.742 1.00 0.00 C
ATOM 114 CB VAL 15 16.995 24.248 18.740 1.00 0.00 C
ATOM 115 CG1 VAL 15 16.874 24.889 20.117 1.00 0.00 C
ATOM 116 CG2 VAL 15 15.626 23.746 18.292 1.00 0.00 C
ATOM 117 C VAL 15 16.490 26.405 17.667 1.00 0.00 C
ATOM 118 O VAL 15 15.719 26.486 16.713 1.00 0.00 O
ATOM 119 N THR 16 16.486 27.277 18.679 1.00 0.00 N
ATOM 120 CA THR 16 15.562 28.392 18.725 1.00 0.00 C
ATOM 121 CB THR 16 15.772 29.227 19.984 1.00 0.00 C
ATOM 122 CG2 THR 16 15.545 28.356 21.215 1.00 0.00 C
ATOM 123 OG1 THR 16 17.107 29.737 20.001 1.00 0.00 O
ATOM 124 C THR 16 15.749 29.308 17.523 1.00 0.00 C
ATOM 125 O THR 16 14.775 29.727 16.901 1.00 0.00 O
ATOM 126 N GLU 17 17.006 29.618 17.199 1.00 0.00 N
ATOM 127 CA GLU 17 17.316 30.481 16.076 1.00 0.00 C
ATOM 128 CB GLU 17 18.822 30.670 15.929 1.00 0.00 C
ATOM 129 CG GLU 17 19.103 31.573 14.752 1.00 0.00 C
ATOM 130 CD GLU 17 20.611 31.762 14.604 1.00 0.00 C
ATOM 131 OE1 GLU 17 21.226 30.940 13.874 1.00 0.00 O
ATOM 132 OE2 GLU 17 21.134 32.728 15.222 1.00 0.00 O1-
ATOM 133 C GLU 17 16.792 29.895 14.774 1.00 0.00 C
ATOM 134 O GLU 17 16.192 28.822 14.769 1.00 0.00 O
ATOM 135 N NHE 18 17.015 30.596 13.663 1.00 0.00 N
TER 136 NHE 18
```

B2 extended

```
ATOM 1 CH3 ACE 1 28.608 21.610 32.298 1.00 0.00 C
ATOM 2 C ACE 1 29.240 22.384 31.135 1.00 0.00 C
ATOM 3 O ACE 1 29.406 23.622 31.282 1.00 0.00 O
ATOM 4 N SER 2 29.453 21.666 30.018 1.00 0.00 N
ATOM 5 CA SER 2 29.732 22.254 28.643 1.00 0.00 C
ATOM 6 CB SER 2 30.900 21.463 28.035 1.00 0.00 C
ATOM 7 OG SER 2 31.572 22.388 27.260 1.00 0.00 O
ATOM 8 C SER 2 28.518 22.440 27.776 1.00 0.00 C
ATOM 9 O SER 2 27.356 22.034 28.140 1.00 0.00 O
ATOM 10 N TRP 3 28.656 23.153 26.639 1.00 0.00 N
ATOM 11 CA TRP 3 27.523 23.593 25.762 1.00 0.00 C
ATOM 12 CB TRP 3 28.053 24.667 24.898 1.00 0.00 C
ATOM 13 CG TRP 3 28.394 25.978 25.633 1.00 0.00 C
ATOM 14 CD1 TRP 3 29.605 26.355 26.004 1.00 0.00 C
ATOM 15 NE1 TRP 3 29.532 27.360 26.916 1.00 0.00 N
ATOM 16 CE2 TRP 3 28.214 27.723 27.232 1.00 0.00 C
ATOM 17 CZ2 TRP 3 27.706 28.702 28.108 1.00 0.00 C
ATOM 18 CH2 TRP 3 26.333 28.774 28.260 1.00 0.00 C
ATOM 19 CZ3 TRP 3 25.473 27.875 27.575 1.00 0.00 C
ATOM 20 CE3 TRP 3 26.047 26.953 26.616 1.00 0.00 C
ATOM 21 CD2 TRP 3 27.409 26.803 26.474 1.00 0.00 C
ATOM 22 C TRP 3 26.834 22.512 25.015 1.00 0.00 C
ATOM 23 O TRP 3 25.887 22.712 24.321 1.00 0.00 O
ATOM 24 N THR 4 27.301 21.234 25.063 1.00 0.00 N
ATOM 25 CA THR 4 26.759 19.943 24.464 1.00 0.00 C
ATOM 26 CB THR 4 27.796 18.821 24.565 1.00 0.00 C
ATOM 27 CG2 THR 4 28.595 18.590 23.290 1.00 0.00 C
ATOM 28 OG1 THR 4 28.736 19.049 25.651 1.00 0.00 O
ATOM 29 C THR 4 25.417 19.451 25.088 1.00 0.00 C
ATOM 30 O THR 4 24.654 18.647 24.455 1.00 0.00 O
ATOM 31 N TRP 5 25.180 19.807 26.373 1.00 0.00 N
ATOM 32 CA TRP 5 24.208 19.233 27.306 1.00 0.00 C
ATOM 33 CB TRP 5 24.827 18.854 28.665 1.00 0.00 C
ATOM 34 CG TRP 5 25.740 17.653 28.616 1.00 0.00 C
ATOM 35 CD1 TRP 5 25.680 16.566 27.801 1.00 0.00 C
ATOM 36 NE1 TRP 5 26.569 15.594 28.175 1.00 0.00 N
ATOM 37 CE2 TRP 5 27.394 16.018 29.116 1.00 0.00 C
ATOM 38 CZ2 TRP 5 28.547 15.541 29.735 1.00 0.00 C
ATOM 39 CH2 TRP 5 29.130 16.261 30.755 1.00 0.00 C
ATOM 40 CZ3 TRP 5 28.683 17.516 31.128 1.00 0.00 C
ATOM 41 CE3 TRP 5 27.550 18.077 30.488 1.00 0.00 C
ATOM 42 CD2 TRP 5 26.889 17.316 29.491 1.00 0.00 C
ATOM 43 C TRP 5 23.020 20.236 27.453 1.00 0.00 C
ATOM 44 O TRP 5 22.073 19.879 28.201 1.00 0.00 O
ATOM 45 N GLU 6 23.054 21.383 26.761 1.00 0.00 N
ATOM 46 CA GLU 6 21.873 22.292 26.659 1.00 0.00 C
ATOM 47 CB GLU 6 22.303 23.725 27.059 1.00 0.00 C
ATOM 48 CG GLU 6 22.721 23.701 28.568 1.00 0.00 C
ATOM 49 CD GLU 6 23.050 25.140 29.036 1.00 0.00 C
ATOM 50 OE1 GLU 6 24.097 25.257 29.745 1.00 0.00 O
ATOM 51 OE2 GLU 6 22.365 26.164 28.672 1.00 0.00 O1-
ATOM 52 C GLU 6 21.058 22.265 25.318 1.00 0.00 C
ATOM 53 O GLU 6 20.534 23.255 24.830 1.00 0.00 O
ATOM 54 N ASN 7 21.236 21.184 24.564 1.00 0.00 N
ATOM 55 CA ASN 7 20.725 20.855 23.295 1.00 0.00 C
ATOM 56 CB ASN 7 21.297 19.414 22.986 1.00 0.00 C
ATOM 57 CG ASN 7 21.523 19.146 21.490 1.00 0.00 C
ATOM 58 OD1 ASN 7 22.446 19.591 20.888 1.00 0.00 O
ATOM 59 ND2 ASN 7 20.669 18.441 20.874 1.00 0.00 N
ATOM 60 C ASN 7 19.195 20.867 23.124 1.00 0.00 C
ATOM 61 O ASN 7 18.395 20.668 24.035 1.00 0.00 O
ATOM 62 N GLY 8 18.829 21.100 21.877 1.00 0.00 N
ATOM 63 CA GLY 8 17.458 21.148 21.365 1.00 0.00 C
ATOM 64 C GLY 8 16.554 22.344 21.709 1.00 0.00 C
ATOM 65 O GLY 8 15.437 22.495 21.231 1.00 0.00 O
ATOM 66 N LYS 9 17.025 23.197 22.626 1.00 0.00 N
ATOM 67 CA LYS 9 16.309 24.393 23.063 1.00 0.00 C
ATOM 68 CB LYS 9 16.810 24.874 24.500 1.00 0.00 C
ATOM 69 CG LYS 9 16.675 23.793 25.562 1.00 0.00 C
ATOM 70 CD LYS 9 15.270 23.199 25.749 1.00 0.00 C
ATOM 71 CE LYS 9 15.353 22.212 26.938 1.00 0.00 C
ATOM 72 NZ LYS 9 13.964 21.717 27.189 1.00 0.00 N1+
ATOM 73 C LYS 9 16.151 25.582 22.086 1.00 0.00 C
ATOM 74 O LYS 9 15.306 26.417 22.176 1.00 0.00 O
ATOM 75 N TRP 10 16.999 25.527 21.092 1.00 0.00 N
ATOM 76 CA TRP 10 17.321 26.553 20.122 1.00 0.00 C
ATOM 77 CB TRP 10 18.784 27.127 20.394 1.00 0.00 C
ATOM 78 CG TRP 10 18.952 27.885 21.681 1.00 0.00 C
ATOM 79 CD1 TRP 10 19.549 27.338 22.762 1.00 0.00 C
ATOM 80 NE1 TRP 10 19.468 28.206 23.878 1.00 0.00 N
ATOM 81 CE2 TRP 10 18.893 29.356 23.506 1.00 0.00 C
ATOM 82 CZ2 TRP 10 18.580 30.519 24.245 1.00 0.00 C
ATOM 83 CH2 TRP 10 17.937 31.642 23.600 1.00 0.00 C
ATOM 84 CZ3 TRP 10 17.394 31.392 22.316 1.00 0.00 C
ATOM 85 CE3 TRP 10 17.729 30.261 21.607 1.00 0.00 C
ATOM 86 CD2 TRP 10 18.506 29.187 22.133 1.00 0.00 C
ATOM 87 C TRP 10 17.406 25.945 18.686 1.00 0.00 C
ATOM 88 O TRP 10 17.922 24.824 18.582 1.00 0.00 O
ATOM 89 N THR 11 16.954 26.624 17.669 1.00 0.00 N
ATOM 90 CA THR 11 16.900 26.161 16.209 1.00 0.00 C
ATOM 91 CB THR 11 15.763 26.801 15.378 1.00 0.00 C
ATOM 92 CG2 THR 11 14.352 26.291 15.692 1.00 0.00 C
ATOM 93 OG1 THR 11 15.703 28.147 15.797 1.00 0.00 O
ATOM 94 C THR 11 18.307 26.334 15.478 1.00 0.00 C
ATOM 95 O THR 11 18.325 25.896 14.323 1.00 0.00 O
ATOM 96 N TRP 12 19.286 26.964 16.034 1.00 0.00 N
ATOM 97 CA TRP 12 20.536 27.319 15.320 1.00 0.00 C
ATOM 98 CB TRP 12 20.923 28.819 15.465 1.00 0.00 C
ATOM 99 CG TRP 12 20.760 29.409 16.863 1.00 0.00 C
ATOM 100 CD1 TRP 12 21.404 28.970 17.955 1.00 0.00 C
ATOM 101 NE1 TRP 12 21.120 29.749 19.009 1.00 0.00 N
ATOM 102 CE2 TRP 12 20.232 30.740 18.711 1.00 0.00 C
ATOM 103 CZ2 TRP 12 19.571 31.695 19.462 1.00 0.00 C
ATOM 104 CH2 TRP 12 18.588 32.588 18.900 1.00 0.00 C
ATOM 105 CZ3 TRP 12 18.254 32.309 17.592 1.00 0.00 C
ATOM 106 CE3 TRP 12 18.820 31.276 16.840 1.00 0.00 C
ATOM 107 CD2 TRP 12 19.938 30.546 17.322 1.00 0.00 C
ATOM 108 C TRP 12 21.600 26.375 15.769 1.00 0.00 C
ATOM 109 O TRP 12 21.669 25.883 16.860 1.00 0.00 O
ATOM 110 N LYS 13 22.504 26.092 14.806 1.00 0.00 N
ATOM 111 CA LYS 13 23.678 25.181 14.892 1.00 0.00 C
ATOM 112 CB LYS 13 23.589 23.825 14.290 1.00 0.00 C
ATOM 113 CG LYS 13 22.606 22.954 15.034 1.00 0.00 C
ATOM 114 CD LYS 13 22.711 21.451 14.811 1.00 0.00 C
ATOM 115 CE LYS 13 22.001 21.163 13.431 1.00 0.00 C
ATOM 116 NZ LYS 13 22.004 19.726 13.112 1.00 0.00 N1+
ATOM 117 C LYS 13 25.040 25.831 14.504 1.00 0.00 C
ATOM 118 O LYS 13 25.172 26.598 13.530 1.00 0.00 O
ATOM 119 N NHE 14 26.102 25.549 15.260 1.00 0.00 N
TER 120 NHE 14
```

B2 misfolded

```
ATOM 1 CH3 ACE 1 27.375 19.046 34.055 1.00 0.00 C
ATOM 2 C ACE 1 27.392 20.412 33.367 1.00 0.00 C
ATOM 3 O ACE 1 27.360 21.446 34.032 1.00 0.00 O
ATOM 4 N SER 2 27.440 20.412 32.055 1.00 0.00 N
ATOM 5 CA SER 2 27.461 21.648 31.298 1.00 0.00 C
ATOM 6 CB SER 2 28.676 22.496 31.664 1.00 0.00 C
ATOM 7 OG SER 2 28.660 23.704 30.899 1.00 0.00 O
ATOM 8 C SER 2 27.518 21.375 29.802 1.00 0.00 C
ATOM 9 O SER 2 26.793 21.994 29.027 1.00 0.00 O
ATOM 10 N TRP 3 28.383 20.442 29.398 1.00 0.00 N
ATOM 11 CA TRP 3 28.532 20.090 28.000 1.00 0.00 C
ATOM 12 CB TRP 3 29.585 19.001 27.817 1.00 0.00 C
ATOM 13 CG TRP 3 29.810 18.567 26.389 1.00 0.00 C
ATOM 14 CD1 TRP 3 30.671 17.635 25.957 1.00 0.00 C
ATOM 15 NE1 TRP 3 30.535 17.567 24.535 1.00 0.00 N
ATOM 16 CE2 TRP 3 29.627 18.429 24.152 1.00 0.00 C
ATOM 17 CZ2 TRP 3 29.180 18.692 22.851 1.00 0.00 C
ATOM 18 CH2 TRP 3 28.197 19.667 22.729 1.00 0.00 C
ATOM 19 CZ3 TRP 3 27.716 20.310 23.815 1.00 0.00 C
ATOM 20 CE3 TRP 3 28.181 20.028 25.116 1.00 0.00 C
ATOM 21 CD2 TRP 3 29.171 19.046 25.239 1.00 0.00 C
ATOM 22 C TRP 3 27.222 19.573 27.423 1.00 0.00 C
ATOM 23 O TRP 3 26.827 19.962 26.326 1.00 0.00 O
ATOM 24 N THR 4 26.548 18.693 28.167 1.00 0.00 N
ATOM 25 CA THR 4 25.289 18.126 27.727 1.00 0.00 C
ATOM 26 CB THR 4 24.734 17.153 28.762 1.00 0.00 C
ATOM 27 CG2 THR 4 25.730 16.019 28.978 1.00 0.00 C
ATOM 28 OG1 THR 4 24.521 17.841 29.998 1.00 0.00 O
ATOM 29 C THR 4 24.246 19.214 27.507 1.00 0.00 C
ATOM 30 O THR 4 23.539 19.204 26.503 1.00 0.00 O
ATOM 31 N TRP 5 24.154 20.153 28.451 1.00 0.00 N
ATOM 32 CA TRP 5 23.202 21.241 28.357 1.00 0.00 C
ATOM 33 CB TRP 5 23.306 22.168 29.564 1.00 0.00 C
ATOM 34 CG TRP 5 22.350 23.337 29.556 1.00 0.00 C
ATOM 35 CD1 TRP 5 22.236 24.292 30.489 1.00 0.00 C
ATOM 36 NE1 TRP 5 21.204 25.180 30.054 1.00 0.00 N
ATOM 37 CE2 TRP 5 20.731 24.757 28.907 1.00 0.00 C
ATOM 38 CZ2 TRP 5 19.715 25.326 28.130 1.00 0.00 C
ATOM 39 CH2 TRP 5 19.410 24.664 26.946 1.00 0.00 C
ATOM 40 CZ3 TRP 5 20.068 23.541 26.589 1.00 0.00 C
ATOM 41 CE3 TRP 5 21.089 22.986 27.386 1.00 0.00 C
ATOM 42 CD2 TRP 5 21.397 23.653 28.579 1.00 0.00 C
ATOM 43 C TRP 5 23.443 22.075 27.107 1.00 0.00 C
ATOM 44 O TRP 5 22.503 22.420 26.395 1.00 0.00 O
ATOM 45 N GLU 6 24.711 22.399 26.841 1.00 0.00 N
ATOM 46 CA GLU 6 25.072 23.190 25.681 1.00 0.00 C
ATOM 47 CB GLU 6 26.578 23.413 25.617 1.00 0.00 C
ATOM 48 CG GLU 6 26.913 24.239 24.398 1.00 0.00 C
ATOM 49 CD GLU 6 28.423 24.462 24.333 1.00 0.00 C
ATOM 50 OE1 GLU 6 29.105 23.610 23.705 1.00 0.00 O
ATOM 51 OE2 GLU 6 28.879 25.484 24.912 1.00 0.00 O1-
ATOM 52 C GLU 6 24.649 22.498 24.394 1.00 0.00 C
ATOM 53 O GLU 6 24.093 23.131 23.498 1.00 0.00 O
ATOM 54 N ASN 7 24.913 21.192 24.302 1.00 0.00 N
ATOM 55 CA ASN 7 24.560 20.420 23.127 1.00 0.00 C
ATOM 56 CB ASN 7 24.975 18.962 23.284 1.00 0.00 C
ATOM 57 CG ASN 7 24.604 18.151 22.050 1.00 0.00 C
ATOM 58 OD1 ASN 7 24.044 18.687 21.097 1.00 0.00 O
ATOM 59 ND2 ASN 7 24.919 16.853 22.071 1.00 0.00 N
ATOM 60 C ASN 7 23.059 20.454 22.880 1.00 0.00 C
ATOM 61 O ASN 7 22.617 20.644 21.750 1.00 0.00 O
ATOM 62 N GLY 8 22.274 20.269 23.944 1.00 0.00 N
ATOM 63 CA GLY 8 20.828 20.278 23.841 1.00 0.00 C
ATOM 64 C GLY 8 20.344 21.619 23.308 1.00 0.00 C
ATOM 65 O GLY 8 19.485 21.668 22.430 1.00 0.00 O
ATOM 66 N LYS 9 20.896 22.710 23.844 1.00 0.00 N
ATOM 67 CA LYS 9 20.520 24.044 23.423 1.00 0.00 C
ATOM 68 CB LYS 9 21.302 25.103 24.192 1.00 0.00 C
ATOM 69 CG LYS 9 20.877 26.491 23.721 1.00 0.00 C
ATOM 70 CD LYS 9 21.659 27.550 24.492 1.00 0.00 C
ATOM 71 CE LYS 9 21.235 28.937 24.020 1.00 0.00 C
ATOM 72 NZ LYS 9 21.988 29.958 24.762 1.00 0.00 N1+
ATOM 73 C LYS 9 20.796 24.249 21.940 1.00 0.00 C
ATOM 74 O LYS 9 19.959 24.788 21.218 1.00 0.00 O
ATOM 75 N TRP 10 21.974 23.817 21.486 1.00 0.00 N
ATOM 76 CA TRP 10 22.355 23.954 20.094 1.00 0.00 C
ATOM 77 CB TRP 10 23.746 23.379 19.847 1.00 0.00 C
ATOM 78 CG TRP 10 24.232 23.476 18.420 1.00 0.00 C
ATOM 79 CD1 TRP 10 25.408 23.051 17.938 1.00 0.00 C
ATOM 80 NE1 TRP 10 25.422 23.355 16.541 1.00 0.00 N
ATOM 81 CE2 TRP 10 24.293 23.937 16.220 1.00 0.00 C
ATOM 82 CZ2 TRP 10 23.889 24.399 14.962 1.00 0.00 C
ATOM 83 CH2 TRP 10 22.628 24.984 14.903 1.00 0.00 C
ATOM 84 CZ3 TRP 10 21.858 25.086 16.007 1.00 0.00 C
ATOM 85 CE3 TRP 10 22.286 24.613 17.264 1.00 0.00 C
ATOM 86 CD2 TRP 10 23.555 24.026 17.324 1.00 0.00 C
ATOM 87 C TRP 10 21.380 23.220 19.184 1.00 0.00 C
ATOM 88 O TRP 10 20.960 23.754 18.160 1.00 0.00 O
ATOM 89 N THR 11 21.020 21.990 19.561 1.00 0.00 N
ATOM 90 CA THR 11 20.099 21.190 18.781 1.00 0.00 C
ATOM 91 CB THR 11 19.862 19.832 19.435 1.00 0.00 C
ATOM 92 CG2 THR 11 21.185 19.084 19.546 1.00 0.00 C
ATOM 93 OG1 THR 11 19.310 20.022 20.740 1.00 0.00 O
ATOM 94 C THR 11 18.750 21.882 18.644 1.00 0.00 C
ATOM 95 O THR 11 18.180 21.928 17.557 1.00 0.00 O
ATOM 96 N TRP 12 18.241 22.423 19.754 1.00 0.00 N
ATOM 97 CA TRP 12 16.965 23.109 19.755 1.00 0.00 C
ATOM 98 CB TRP 12 16.623 23.629 21.147 1.00 0.00 C
ATOM 99 CG TRP 12 15.306 24.361 21.248 1.00 0.00 C
ATOM 100 CD1 TRP 12 14.773 24.912 22.348 1.00 0.00 C
ATOM 101 NE1 TRP 12 13.524 25.492 21.961 1.00 0.00 N
ATOM 102 CE2 TRP 12 13.336 25.285 20.681 1.00 0.00 C
ATOM 103 CZ2 TRP 12 12.248 25.681 19.894 1.00 0.00 C
ATOM 104 CH2 TRP 12 12.306 25.328 18.551 1.00 0.00 C
ATOM 105 CZ3 TRP 12 13.360 24.642 18.058 1.00 0.00 C
ATOM 106 CE3 TRP 12 14.444 24.253 18.870 1.00 0.00 C
ATOM 107 CD2 TRP 12 14.386 24.610 20.222 1.00 0.00 C
ATOM 108 C TRP 12 16.983 24.300 18.806 1.00 0.00 C
ATOM 109 O TRP 12 16.042 24.501 18.041 1.00 0.00 O
ATOM 110 N LYS 13 18.058 25.089 18.858 1.00 0.00 N
ATOM 111 CA LYS 13 18.195 26.254 18.006 1.00 0.00 C
ATOM 112 CB LYS 13 19.512 26.975 18.268 1.00 0.00 C
ATOM 113 CG LYS 13 19.617 28.191 17.354 1.00 0.00 C
ATOM 114 CD LYS 13 20.935 28.911 17.617 1.00 0.00 C
ATOM 115 CE LYS 13 21.040 30.127 16.703 1.00 0.00 C
ATOM 116 NZ LYS 13 22.310 30.823 16.956 1.00 0.00 N1+
ATOM 117 C LYS 13 18.163 25.863 16.535 1.00 0.00 C
ATOM 118 O LYS 13 18.035 24.687 16.205 1.00 0.00 O
ATOM 119 N NHE 14 18.280 26.848 15.646 1.00 0.00 N
TER 120 NHE 14
```

B3 extended

```
ATOM 1 CH3 ACE 1 31.907 40.461 23.519 1.00 0.00 C
ATOM 2 C ACE 1 31.519 39.316 22.646 1.00 0.00 C
ATOM 3 O ACE 1 30.714 39.525 21.672 1.00 0.00 O
ATOM 4 N GLN 2 31.846 38.129 23.180 1.00 0.00 N
ATOM 5 CA GLN 2 31.472 36.833 22.648 1.00 0.00 C
ATOM 6 CB GLN 2 32.617 36.244 21.778 1.00 0.00 C
ATOM 7 CG GLN 2 33.028 37.136 20.605 1.00 0.00 C
ATOM 8 CD GLN 2 33.494 36.373 19.423 1.00 0.00 C
ATOM 9 OE1 GLN 2 32.737 35.597 18.771 1.00 0.00 O
ATOM 10 NE2 GLN 2 34.787 36.529 18.924 1.00 0.00 N
ATOM 11 C GLN 2 31.019 35.786 23.705 1.00 0.00 C
ATOM 12 O GLN 2 31.478 35.828 24.872 1.00 0.00 O
ATOM 13 N ILE 3 30.185 34.872 23.221 1.00 0.00 N
ATOM 14 CA ILE 3 29.799 33.688 23.872 1.00 0.00 C
ATOM 15 CB ILE 3 28.223 33.735 24.121 1.00 0.00 C
ATOM 16 CG2 ILE 3 27.809 34.717 25.214 1.00 0.00 C
ATOM 17 CG1 ILE 3 27.412 33.761 22.757 1.00 0.00 C
ATOM 18 CD1 ILE 3 26.093 33.002 22.733 1.00 0.00 C
ATOM 19 C ILE 3 30.183 32.293 23.340 1.00 0.00 C
ATOM 20 O ILE 3 30.542 32.185 22.162 1.00 0.00 O
ATOM 21 N PHE 4 30.133 31.306 24.241 1.00 0.00 N
ATOM 22 CA PHE 4 30.585 29.930 24.051 1.00 0.00 C
ATOM 23 CB PHE 4 31.618 29.535 25.056 1.00 0.00 C
ATOM 24 CG PHE 4 33.022 30.173 24.876 1.00 0.00 C
ATOM 25 CD1 PHE 4 33.558 31.167 25.726 1.00 0.00 C
ATOM 26 CE1 PHE 4 34.914 31.454 25.702 1.00 0.00 C
ATOM 27 CZ PHE 4 35.721 30.868 24.663 1.00 0.00 C
ATOM 28 CE2 PHE 4 35.152 29.963 23.709 1.00 0.00 C
ATOM 29 CD2 PHE 4 33.792 29.717 23.800 1.00 0.00 C
ATOM 30 C PHE 4 29.340 28.965 24.211 1.00 0.00 C
ATOM 31 O PHE 4 28.373 29.294 24.900 1.00 0.00 O
ATOM 32 N VAL 5 29.370 27.880 23.433 1.00 0.00 N
ATOM 33 CA VAL 5 28.225 26.876 23.312 1.00 0.00 C
ATOM 34 CB VAL 5 28.301 25.944 22.080 1.00 0.00 C
ATOM 35 CG1 VAL 5 28.482 26.818 20.847 1.00 0.00 C
ATOM 36 CG2 VAL 5 29.559 25.029 22.172 1.00 0.00 C
ATOM 37 C VAL 5 27.961 26.030 24.587 1.00 0.00 C
ATOM 38 O VAL 5 27.047 25.196 24.648 1.00 0.00 O
ATOM 39 N LYS 6 28.771 26.261 25.589 1.00 0.00 N
ATOM 40 CA LYS 6 28.770 25.516 26.887 1.00 0.00 C
ATOM 41 CB LYS 6 29.712 26.175 27.894 1.00 0.00 C
ATOM 42 CG LYS 6 29.761 25.308 29.075 1.00 0.00 C
ATOM 43 CD LYS 6 30.692 25.833 30.143 1.00 0.00 C
ATOM 44 CE LYS 6 31.027 24.900 31.348 1.00 0.00 C
ATOM 45 NZ LYS 6 31.910 25.685 32.306 1.00 0.00 N1+
ATOM 46 C LYS 6 27.401 25.429 27.390 1.00 0.00 C
ATOM 47 O LYS 6 26.938 24.326 27.714 1.00 0.00 O
ATOM 48 N THR 7 26.676 26.534 27.386 1.00 0.00 N
ATOM 49 CA THR 7 25.344 26.749 27.941 1.00 0.00 C
ATOM 50 CB THR 7 25.127 28.275 28.259 1.00 0.00 C
ATOM 51 CG2 THR 7 26.131 28.858 29.179 1.00 0.00 C
ATOM 52 OG1 THR 7 25.196 29.021 27.073 1.00 0.00 O
ATOM 53 C THR 7 24.132 26.292 27.015 1.00 0.00 C
ATOM 54 O THR 7 23.078 25.982 27.513 1.00 0.00 O
ATOM 55 N LEU 8 24.313 26.250 25.680 1.00 0.00 N
ATOM 56 CA LEU 8 23.509 25.638 24.651 1.00 0.00 C
ATOM 57 CB LEU 8 23.945 26.298 23.303 1.00 0.00 C
ATOM 58 CG LEU 8 23.178 25.711 22.117 1.00 0.00 C
ATOM 59 CD1 LEU 8 21.733 26.250 22.049 1.00 0.00 C
ATOM 60 CD2 LEU 8 23.993 26.138 20.880 1.00 0.00 C
ATOM 61 C LEU 8 23.574 24.125 24.598 1.00 0.00 C
ATOM 62 O LEU 8 22.544 23.437 24.463 1.00 0.00 O
ATOM 63 N THR 9 24.747 23.533 24.655 1.00 0.00 N
ATOM 64 CA THR 9 24.957 22.112 24.720 1.00 0.00 C
ATOM 65 CB THR 9 26.480 21.881 24.860 1.00 0.00 C
ATOM 66 CG2 THR 9 26.832 20.429 24.588 1.00 0.00 C
ATOM 67 OG1 THR 9 27.357 22.589 23.969 1.00 0.00 O
ATOM 68 C THR 9 24.236 21.484 25.960 1.00 0.00 C
ATOM 69 O THR 9 23.571 20.470 25.840 1.00 0.00 O
ATOM 70 N GLY 10 24.253 22.238 27.056 1.00 0.00 N
ATOM 71 CA GLY 10 23.442 21.890 28.193 1.00 0.00 C
ATOM 72 C GLY 10 21.938 22.086 28.065 1.00 0.00 C
ATOM 73 O GLY 10 21.197 21.621 28.950 1.00 0.00 O
ATOM 74 N LYS 11 21.358 22.642 27.010 1.00 0.00 N
ATOM 75 CA LYS 11 19.977 22.591 26.584 1.00 0.00 C
ATOM 76 CB LYS 11 19.639 23.959 26.032 1.00 0.00 C
ATOM 77 CG LYS 11 19.116 24.912 27.153 1.00 0.00 C
ATOM 78 CD LYS 11 18.953 26.373 26.701 1.00 0.00 C
ATOM 79 CE LYS 11 18.454 27.297 27.827 1.00 0.00 C
ATOM 80 NZ LYS 11 16.986 27.060 28.100 1.00 0.00 N1+
ATOM 81 C LYS 11 19.701 21.495 25.458 1.00 0.00 C
ATOM 82 O LYS 11 18.545 21.239 25.131 1.00 0.00 O
ATOM 83 N THR 12 20.733 20.813 24.876 1.00 0.00 N
ATOM 84 CA THR 12 20.708 20.088 23.671 1.00 0.00 C
ATOM 85 CB THR 12 21.294 20.710 22.455 1.00 0.00 C
ATOM 86 CG2 THR 12 20.187 21.567 21.741 1.00 0.00 C
ATOM 87 OG1 THR 12 22.483 21.472 22.585 1.00 0.00 O
ATOM 88 C THR 12 21.225 18.610 23.764 1.00 0.00 C
ATOM 89 O THR 12 20.779 17.767 23.068 1.00 0.00 O
ATOM 90 N ILE 13 22.197 18.321 24.657 1.00 0.00 N
ATOM 91 CA ILE 13 22.849 17.034 24.749 1.00 0.00 C
ATOM 92 CB ILE 13 24.158 17.181 25.682 1.00 0.00 C
ATOM 93 CG2 ILE 13 23.806 17.390 27.129 1.00 0.00 C
ATOM 94 CG1 ILE 13 25.081 15.971 25.540 1.00 0.00 C
ATOM 95 CD1 ILE 13 25.729 15.791 24.199 1.00 0.00 C
ATOM 96 C ILE 13 21.926 15.860 25.192 1.00 0.00 C
ATOM 97 O ILE 13 22.157 14.775 24.744 1.00 0.00 O
ATOM 98 N THR 14 20.953 16.079 26.096 1.00 0.00 N
ATOM 99 CA THR 14 19.901 15.165 26.380 1.00 0.00 C
ATOM 100 CB THR 14 19.333 15.429 27.773 1.00 0.00 C
ATOM 101 CG2 THR 14 20.358 15.067 28.832 1.00 0.00 C
ATOM 102 OG1 THR 14 19.157 16.885 27.959 1.00 0.00 O
ATOM 103 C THR 14 18.735 15.234 25.478 1.00 0.00 C
ATOM 104 O THR 14 17.856 14.383 25.642 1.00 0.00 O
ATOM 105 N LEU 15 18.733 16.126 24.442 1.00 0.00 N
ATOM 106 CA LEU 15 17.765 16.257 23.330 1.00 0.00 C
ATOM 107 CB LEU 15 17.460 17.832 22.954 1.00 0.00 C
ATOM 108 CG LEU 15 16.416 17.938 21.821 1.00 0.00 C
ATOM 109 CD1 LEU 15 15.034 17.389 22.028 1.00 0.00 C
ATOM 110 CD2 LEU 15 16.257 19.396 21.423 1.00 0.00 C
ATOM 111 C LEU 15 18.311 15.449 22.093 1.00 0.00 C
ATOM 112 O LEU 15 17.657 14.503 21.647 1.00 0.00 O
ATOM 113 N GLU 16 19.379 15.979 21.432 1.00 0.00 N
ATOM 114 CA GLU 16 19.946 15.381 20.205 1.00 0.00 C
ATOM 115 CB GLU 16 20.482 16.463 19.284 1.00 0.00 C
ATOM 116 CG GLU 16 19.404 17.475 18.885 1.00 0.00 C
ATOM 117 CD GLU 16 19.786 18.233 17.584 1.00 0.00 C
ATOM 118 OE1 GLU 16 20.679 19.118 17.696 1.00 0.00 O
ATOM 119 OE2 GLU 16 19.249 17.974 16.480 1.00 0.00 O1-
ATOM 120 C GLU 16 20.918 14.213 20.528 1.00 0.00 C
ATOM 121 O GLU 16 21.240 13.487 19.582 1.00 0.00 O
ATOM 122 N NHE 17 21.422 14.088 21.715 1.00 0.00 N
TER 123 NHE 17
```

B3 misfolded

```
ATOM 1 CH3 ACE 1 27.711 17.233 34.712 1.00 0.00 C
ATOM 2 C ACE 1 27.971 18.665 34.241 1.00 0.00 C
ATOM 3 O ACE 1 28.066 19.582 35.054 1.00 0.00 O
ATOM 4 N GLN 2 28.083 18.850 32.945 1.00 0.00 N
ATOM 5 CA GLN 2 28.330 20.164 32.386 1.00 0.00 C
ATOM 6 CB GLN 2 29.641 20.744 32.907 1.00 0.00 C
ATOM 7 CG GLN 2 29.863 22.124 32.298 1.00 0.00 C
ATOM 8 CD GLN 2 31.165 22.738 32.794 1.00 0.00 C
ATOM 9 OE1 GLN 2 31.881 22.128 33.584 1.00 0.00 O
ATOM 10 NE2 GLN 2 31.469 23.952 32.327 1.00 0.00 N
ATOM 11 C GLN 2 28.417 20.107 30.869 1.00 0.00 C
ATOM 12 O GLN 2 27.835 20.941 30.179 1.00 0.00 O
ATOM 13 N ILE 3 29.147 19.118 30.348 1.00 0.00 N
ATOM 14 CA ILE 3 29.308 18.956 28.917 1.00 0.00 C
ATOM 15 CB ILE 3 30.192 17.744 28.640 1.00 0.00 C
ATOM 16 CG2 ILE 3 29.540 16.495 29.223 1.00 0.00 C
ATOM 17 CG1 ILE 3 30.360 17.573 27.133 1.00 0.00 C
ATOM 18 CD1 ILE 3 31.244 16.362 26.856 1.00 0.00 C
ATOM 19 C ILE 3 27.963 18.744 28.236 1.00 0.00 C
ATOM 20 O ILE 3 27.687 19.348 27.201 1.00 0.00 O
ATOM 21 N PHE 4 27.126 17.883 28.820 1.00 0.00 N
ATOM 22 CA PHE 4 25.818 17.596 28.268 1.00 0.00 C
ATOM 23 CB PHE 4 25.071 16.582 29.128 1.00 0.00 C
ATOM 24 CG PHE 4 23.692 16.217 28.633 1.00 0.00 C
ATOM 25 CD1 PHE 4 22.913 15.296 29.344 1.00 0.00 C
ATOM 26 CE1 PHE 4 21.634 14.959 28.884 1.00 0.00 C
ATOM 27 CZ PHE 4 21.134 15.543 27.714 1.00 0.00 C
ATOM 28 CE2 PHE 4 21.912 16.464 27.003 1.00 0.00 C
ATOM 29 CD2 PHE 4 23.191 16.802 27.463 1.00 0.00 C
ATOM 30 C PHE 4 24.967 18.856 28.190 1.00 0.00 C
ATOM 31 O PHE 4 24.316 19.107 27.178 1.00 0.00 O
ATOM 32 N VAL 5 24.974 19.649 29.263 1.00 0.00 N
ATOM 33 CA VAL 5 24.207 20.877 29.313 1.00 0.00 C
ATOM 34 CB VAL 5 24.394 21.588 30.649 1.00 0.00 C
ATOM 35 CG1 VAL 5 25.868 21.927 30.842 1.00 0.00 C
ATOM 36 CG2 VAL 5 23.572 22.873 30.660 1.00 0.00 C
ATOM 37 C VAL 5 24.635 21.836 28.211 1.00 0.00 C
ATOM 38 O VAL 5 23.794 22.427 27.537 1.00 0.00 O
ATOM 39 N LYS 6 25.948 21.989 28.029 1.00 0.00 N
ATOM 40 CA LYS 6 26.483 22.874 27.013 1.00 0.00 C
ATOM 41 CB LYS 6 28.007 22.861 27.021 1.00 0.00 C
ATOM 42 CG LYS 6 28.529 23.801 25.940 1.00 0.00 C
ATOM 43 CD LYS 6 30.054 23.789 25.948 1.00 0.00 C
ATOM 44 CE LYS 6 30.576 24.729 24.867 1.00 0.00 C
ATOM 45 NZ LYS 6 32.046 24.717 24.874 1.00 0.00 N1+
ATOM 46 C LYS 6 26.022 22.454 25.625 1.00 0.00 C
ATOM 47 O LYS 6 25.614 23.292 24.823 1.00 0.00 O
ATOM 48 N THR 7 26.086 21.151 25.342 1.00 0.00 N
ATOM 49 CA THR 7 25.676 20.626 24.055 1.00 0.00 C
ATOM 50 CB THR 7 25.853 19.112 23.998 1.00 0.00 C
ATOM 51 CG2 THR 7 27.321 18.763 24.222 1.00 0.00 C
ATOM 52 OG1 THR 7 25.056 18.501 25.015 1.00 0.00 O
ATOM 53 C THR 7 24.211 20.934 23.778 1.00 0.00 C
ATOM 54 O THR 7 23.860 21.356 22.679 1.00 0.00 O
ATOM 55 N LEU 8 23.357 20.723 24.781 1.00 0.00 N
ATOM 56 CA LEU 8 21.937 20.977 24.644 1.00 0.00 C
ATOM 57 CB LEU 8 21.193 20.652 25.935 1.00 0.00 C
ATOM 58 CG LEU 8 19.706 20.934 25.750 1.00 0.00 C
ATOM 59 CD1 LEU 8 19.162 20.068 24.618 1.00 0.00 C
ATOM 60 CD2 LEU 8 18.962 20.608 27.041 1.00 0.00 C
ATOM 61 C LEU 8 21.672 22.439 24.310 1.00 0.00 C
ATOM 62 O LEU 8 20.870 22.742 23.431 1.00 0.00 O
ATOM 63 N THR 9 22.351 23.345 25.017 1.00 0.00 N
ATOM 64 CA THR 9 22.187 24.768 24.794 1.00 0.00 C
ATOM 65 CB THR 9 23.077 25.576 25.734 1.00 0.00 C
ATOM 66 CG2 THR 9 22.707 25.263 27.180 1.00 0.00 C
ATOM 67 OG1 THR 9 24.447 25.231 25.506 1.00 0.00 O
ATOM 68 C THR 9 22.555 25.145 23.367 1.00 0.00 C
ATOM 69 O THR 9 21.837 25.902 22.717 1.00 0.00 O
ATOM 70 N GLY 10 23.679 24.614 22.878 1.00 0.00 N
ATOM 71 CA GLY 10 24.137 24.896 21.533 1.00 0.00 C
ATOM 72 C GLY 10 23.097 24.449 20.514 1.00 0.00 C
ATOM 73 O GLY 10 22.792 25.178 19.573 1.00 0.00 O
ATOM 74 N LYS 11 22.552 23.245 20.704 1.00 0.00 N
ATOM 75 CA LYS 11 21.551 22.707 19.805 1.00 0.00 C
ATOM 76 CB LYS 11 21.100 21.322 20.254 1.00 0.00 C
ATOM 77 CG LYS 11 20.049 20.790 19.284 1.00 0.00 C
ATOM 78 CD LYS 11 19.598 19.405 19.732 1.00 0.00 C
ATOM 79 CE LYS 11 18.547 18.874 18.762 1.00 0.00 C
ATOM 80 NZ LYS 11 18.112 17.538 19.193 1.00 0.00 N1+
ATOM 81 C LYS 11 20.322 23.603 19.755 1.00 0.00 C
ATOM 82 O LYS 11 19.801 23.886 18.677 1.00 0.00 O
ATOM 83 N THR 12 19.859 24.050 20.924 1.00 0.00 N
ATOM 84 CA THR 12 18.696 24.910 21.008 1.00 0.00 C
ATOM 85 CB THR 12 18.385 25.272 22.457 1.00 0.00 C
ATOM 86 CG2 THR 12 18.109 23.999 23.251 1.00 0.00 C
ATOM 87 OG1 THR 12 19.503 25.957 23.028 1.00 0.00 O
ATOM 88 C THR 12 18.916 26.207 20.242 1.00 0.00 C
ATOM 89 O THR 12 18.040 26.651 19.503 1.00 0.00 O
ATOM 90 N ILE 13 20.091 26.815 20.420 1.00 0.00 N
ATOM 91 CA ILE 13 20.422 28.054 19.748 1.00 0.00 C
ATOM 92 CB ILE 13 21.825 28.495 20.154 1.00 0.00 C
ATOM 93 CG2 ILE 13 22.830 27.418 19.758 1.00 0.00 C
ATOM 94 CG1 ILE 13 22.172 29.800 19.446 1.00 0.00 C
ATOM 95 CD1 ILE 13 23.575 30.240 19.851 1.00 0.00 C
ATOM 96 C ILE 13 20.384 27.885 18.236 1.00 0.00 C
ATOM 97 O ILE 13 19.834 28.726 17.527 1.00 0.00 O
ATOM 98 N THR 14 20.970 26.792 17.742 1.00 0.00 N
ATOM 99 CA THR 14 21.001 26.516 16.320 1.00 0.00 C
ATOM 100 CB THR 14 21.729 25.208 16.030 1.00 0.00 C
ATOM 101 CG2 THR 14 23.163 25.301 16.540 1.00 0.00 C
ATOM 102 OG1 THR 14 21.058 24.132 16.691 1.00 0.00 O
ATOM 103 C THR 14 19.593 26.400 15.754 1.00 0.00 C
ATOM 104 O THR 14 19.297 26.956 14.699 1.00 0.00 O
ATOM 105 N LEU 15 18.723 25.672 16.459 1.00 0.00 N
ATOM 106 CA LEU 15 17.353 25.485 16.026 1.00 0.00 C
ATOM 107 CB LEU 15 16.580 24.617 17.012 1.00 0.00 C
ATOM 108 CG LEU 15 15.146 24.444 16.522 1.00 0.00 C
ATOM 109 CD1 LEU 15 15.154 23.774 15.151 1.00 0.00 C
ATOM 110 CD2 LEU 15 14.373 23.575 17.507 1.00 0.00 C
ATOM 111 C LEU 15 16.627 26.818 15.913 1.00 0.00 C
ATOM 112 O LEU 15 15.927 27.066 14.934 1.00 0.00 O
ATOM 113 N GLU 16 16.797 27.679 16.920 1.00 0.00 N
ATOM 114 CA GLU 16 16.160 28.980 16.930 1.00 0.00 C
ATOM 115 CB GLU 16 16.522 29.758 18.191 1.00 0.00 C
ATOM 116 CG GLU 16 15.840 31.105 18.163 1.00 0.00 C
ATOM 117 CD GLU 16 16.202 31.884 19.426 1.00 0.00 C
ATOM 118 OE1 GLU 16 17.225 32.616 19.378 1.00 0.00 O
ATOM 119 OE2 GLU 16 15.450 31.740 20.428 1.00 0.00 O1-
ATOM 120 C GLU 16 16.593 29.811 15.730 1.00 0.00 C
ATOM 121 O GLU 16 17.385 29.356 14.909 1.00 0.00 O
ATOM 122 N NHE 17 16.075 31.035 15.625 1.00 0.00 N
TER 123 NHE 17
```

ID1 extended

```
ATOM   1 CH3 ACE 1  19.290 14.515 17.295 1.00 0.00  C
ATOM   2 C   ACE 1  20.294 15.592 17.607 1.00 0.00  C
ATOM   3 O   ACE 1  21.149 15.419 18.379 1.00 0.00  O
ATOM   4 N   ILE 2  20.232 16.772 16.895 1.00 0.00  N
ATOM   5 CA  ILE 2  21.240 17.823 16.853 1.00 0.00  C
ATOM   6 CB  ILE 2  21.774 18.003 15.376 1.00 0.00  C
ATOM   7 CG2 ILE 2  22.586 19.332 15.299 1.00 0.00  C
ATOM   8 CG1 ILE 2  22.488 16.784 14.841 1.00 0.00  C
ATOM   9 CD1 ILE 2  22.771 16.820 13.313 1.00 0.00  C
ATOM  10 C   ILE 2  20.474 19.079 17.364 1.00 0.00  C
ATOM  11 O   ILE 2  19.383 19.390 16.928 1.00 0.00  O
ATOM  12 N   ASN 3  21.044 19.793 18.325 1.00 0.00  N
ATOM  13 CA  ASN 3  20.577 21.054 18.905 1.00 0.00  C
ATOM  14 CB  ASN 3  21.358 21.299 20.165 1.00 0.00  C
ATOM  15 CG  ASN 3  20.796 22.226 21.169 1.00 0.00  C
ATOM  16 OD1 ASN 3  20.002 23.094 20.851 1.00 0.00  O
ATOM  17 ND2 ASN 3  21.289 22.154 22.387 1.00 0.00  N
ATOM  18 C   ASN 3  20.617 22.201 17.840 1.00 0.00  C
ATOM  19 O   ASN 3  21.632 22.425 17.212 1.00 0.00  O
ATOM  20 N   TRP 4  19.454 22.827 17.621 1.00 0.00  N
ATOM  21 CA  TRP 4  19.340 23.922 16.684 1.00 0.00  C
ATOM  22 CB  TRP 4  17.970 23.812 15.958 1.00 0.00  C
ATOM  23 CG  TRP 4  16.731 23.695 16.774 1.00 0.00  C
ATOM  24 CD1 TRP 4  15.732 24.602 16.789 1.00 0.00  C
ATOM  25 NE1 TRP 4  14.791 24.197 17.754 1.00 0.00  N
ATOM  26 CE2 TRP 4  15.076 22.954 18.267 1.00 0.00  C
ATOM  27 CZ2 TRP 4  14.525 22.170 19.283 1.00 0.00  C
ATOM  28 CH2 TRP 4  15.103 20.992 19.686 1.00 0.00  C
ATOM  29 CZ3 TRP 4  16.214 20.503 19.023 1.00 0.00  C
ATOM  30 CE3 TRP 4  16.816 21.280 17.969 1.00 0.00  C
ATOM  31 CD2 TRP 4  16.318 22.552 17.628 1.00 0.00  C
ATOM  32 C   TRP 4  19.522 25.336 17.354 1.00 0.00  C
ATOM  33 O   TRP 4  19.795 26.283 16.582 1.00 0.00  O
ATOM  34 N   LEU 5  19.448 25.433 18.695 1.00 0.00  N
ATOM  35 CA  LEU 5  19.331 26.668 19.528 1.00 0.00  C
ATOM  36 CB  LEU 5  18.076 26.369 20.478 1.00 0.00  C
ATOM  37 CG  LEU 5  17.669 27.455 21.471 1.00 0.00  C
ATOM  38 CD1 LEU 5  17.815 28.891 20.982 1.00 0.00  C
ATOM  39 CD2 LEU 5  16.246 27.183 21.987 1.00 0.00  C
ATOM  40 C   LEU 5  20.714 26.985 20.204 1.00 0.00  C
ATOM  41 O   LEU 5  21.211 28.189 20.320 1.00 0.00  O
ATOM  42 N   LYS 6  21.421 25.942 20.781 1.00 0.00  N
ATOM  43 CA  LYS 6  22.623 26.157 21.645 1.00 0.00  C
ATOM  44 CB  LYS 6  22.342 25.783 23.159 1.00 0.00  C
ATOM  45 CG  LYS 6  21.306 26.831 23.679 1.00 0.00  C
ATOM  46 CD  LYS 6  20.890 26.586 25.146 1.00 0.00  C
ATOM  47 CE  LYS 6  20.050 27.860 25.495 1.00 0.00  C
ATOM  48 NZ  LYS 6  19.665 28.108 26.904 1.00 0.00  N1+
ATOM  49 C   LYS 6  23.914 25.517 21.087 1.00 0.00  C
ATOM  50 O   LYS 6  24.673 24.876 21.786 1.00 0.00  O
ATOM  51 N   LEU 7  24.165 25.508 19.823 1.00 0.00  N
ATOM  52 CA  LEU 7  25.184 24.651 19.219 1.00 0.00  C
ATOM  53 CB  LEU 7  24.797 24.451 17.702 1.00 0.00  C
ATOM  54 CG  LEU 7  25.750 23.528 16.877 1.00 0.00  C
ATOM  55 CD1 LEU 7  25.849 22.049 17.329 1.00 0.00  C
ATOM  56 CD2 LEU 7  24.986 23.481 15.505 1.00 0.00  C
ATOM  57 C   LEU 7  26.651 25.033 19.445 1.00 0.00  C
ATOM  58 O   LEU 7  27.604 24.229 19.575 1.00 0.00  O
ATOM  59 N   GLY 8  26.930 26.341 19.717 1.00 0.00  N
ATOM  60 CA  GLY 8  28.161 26.895 20.268 1.00 0.00  C
ATOM  61 C   GLY 8  28.485 26.342 21.703 1.00 0.00  C
ATOM  62 O   GLY 8  29.559 26.698 22.225 1.00 0.00  O
ATOM  63 N   LYS 9  27.596 25.628 22.424 1.00 0.00  N
ATOM  64 CA  LYS 9  27.817 25.042 23.773 1.00 0.00  C
ATOM  65 CB  LYS 9  26.858 25.646 24.741 1.00 0.00  C
ATOM  66 CG  LYS 9  27.420 26.971 25.174 1.00 0.00  C
ATOM  67 CD  LYS 9  26.593 27.540 26.290 1.00 0.00  C
ATOM  68 CE  LYS 9  27.158 28.886 26.884 1.00 0.00  C
ATOM  69 NZ  LYS 9  26.278 29.231 28.021 1.00 0.00  N1+
ATOM  70 C   LYS 9  27.870 23.441 23.846 1.00 0.00  C
ATOM  71 O   LYS 9  28.900 22.867 24.244 1.00 0.00  O
ATOM  72 N   MET 10  26.857 22.854 23.203 1.00 0.00  N
ATOM  73 CA  MET 10  26.578 21.426 22.972 1.00 0.00  C
ATOM  74 CB  MET 10  25.800 20.802 24.165 1.00 0.00  C
ATOM  75 CG  MET 10  26.771 20.407 25.307 1.00 0.00  C
ATOM  76 SD  MET 10  26.354 18.897 26.380 1.00 0.00  S
ATOM  77 CE  MET 10  27.700 17.757 25.967 1.00 0.00  C
ATOM  78 C   MET 10  25.777 21.121 21.655 1.00 0.00  C
ATOM  79 O   MET 10  24.792 21.773 21.267 1.00 0.00  O
ATOM  80 N   VAL 11  26.286 20.093 20.969 1.00 0.00  N
ATOM  81 CA  VAL 11  25.529 19.450 19.837 1.00 0.00  C
ATOM  82 CB  VAL 11  26.534 18.545 18.997 1.00 0.00  C
ATOM  83 CG1 VAL 11  25.862 17.810 17.819 1.00 0.00  C
ATOM  84 CG2 VAL 11  28.007 18.990 18.692 1.00 0.00  C
ATOM  85 C   VAL 11  24.380 18.619 20.252 1.00 0.00  C
ATOM  86 O   VAL 11  23.416 18.699 19.483 1.00 0.00  O
ATOM  87 N   ILE 12  24.369 17.854 21.344 1.00 0.00  N
ATOM  88 CA  ILE 12  23.202 16.970 21.682 1.00 0.00  C
ATOM  89 CB  ILE 12  23.642 15.677 22.393 1.00 0.00  C
ATOM  90 CG2 ILE 12  24.307 15.809 23.771 1.00 0.00  C
ATOM  91 CG1 ILE 12  22.473 14.716 22.483 1.00 0.00  C
ATOM  92 CD1 ILE 12  22.846 13.286 22.808 1.00 0.00  C
ATOM  93 C   ILE 12  22.179 17.848 22.514 1.00 0.00  C
ATOM  94 O   ILE 12  22.517 18.764 23.250 1.00 0.00  O
ATOM  95 N   ASP 13  20.902 17.659 22.168 1.00 0.00  N
ATOM  96 CA  ASP 13  19.772 18.461 22.704 1.00 0.00  C
ATOM  97 CB  ASP 13  18.508 18.190 21.907 1.00 0.00  C
ATOM  98 CG  ASP 13  17.311 19.065 22.261 1.00 0.00  C
ATOM  99 OD1 ASP 13  16.174 18.504 22.313 1.00 0.00  O
ATOM 100 OD2 ASP 13  17.467 20.289 22.515 1.00 0.00  O1-
ATOM 101 C   ASP 13  19.519 17.963 24.171 1.00 0.00  C
ATOM 102 O   ASP 13  19.834 16.872 24.538 1.00 0.00  O
ATOM 103 N   ALA 14  18.872 18.814 24.916 1.00 0.00  N
ATOM 104 CA  ALA 14  18.868 18.670 26.397 1.00 0.00  C
ATOM 105 CB  ALA 14  18.356 19.964 27.047 1.00 0.00  C
ATOM 106 C   ALA 14  18.237 17.346 26.849 1.00 0.00  C
ATOM 107 O   ALA 14  18.666 16.785 27.844 1.00 0.00  O
ATOM 108 N   LEU 15  17.152 16.862 26.128 1.00 0.00  N
ATOM 109 CA  LEU 15  16.374 15.615 26.241 1.00 0.00  C
ATOM 110 CB  LEU 15  14.855 15.929 26.300 1.00 0.00  C
ATOM 111 CG  LEU 15  14.420 16.877 27.418 1.00 0.00  C
ATOM 112 CD1 LEU 15  12.889 16.914 27.299 1.00 0.00  C
ATOM 113 CD2 LEU 15  14.781 16.378 28.853 1.00 0.00  C
ATOM 114 C   LEU 15  16.468 14.671 25.067 1.00 0.00  C
ATOM 115 O   LEU 15  16.804 15.105 24.022 1.00 0.00  O
ATOM 116 N   NHE 16  16.214 13.359 25.169 1.00 0.00  N
TER 117  NHE 16
```

ID1 misfolded

```
ATOM   1 CH3 ACE  1  26.596 16.649 34.163 1.00 0.00  C
ATOM   2 C   ACE  1  26.968 18.042 33.650 1.00 0.00  C
ATOM   3 O   ACE  1  27.217 18.952 34.439 1.00 0.00  O
ATOM   4 N   ILE  2  27.001 18.202 32.347 1.00 0.00  N
ATOM   5 CA  ILE  2  27.340 19.479 31.749 1.00 0.00  C
ATOM   6 CB  ILE  2  28.743 19.888 32.187 1.00 0.00  C
ATOM   7 CG2 ILE  2  29.745 18.831 31.733 1.00 0.00  C
ATOM   8 CG1 ILE  2  29.100 21.230 31.559 1.00 0.00  C
ATOM   9 CD1 ILE  2  30.502 21.640 31.996 1.00 0.00  C
ATOM  10 C   ILE  2  27.307 19.399 30.230 1.00 0.00  C
ATOM  11 O   ILE  2  26.765 20.282 29.571 1.00 0.00  O
ATOM  12 N   ASN  3  27.891 18.334 29.675 1.00 0.00  N
ATOM  13 CA  ASN  3  27.927 18.143 28.239 1.00 0.00  C
ATOM  14 CB  ASN  3  28.651 16.850 27.875 1.00 0.00  C
ATOM  15 CG  ASN  3  28.689 16.649 26.367 1.00 0.00  C
ATOM  16 OD1 ASN  3  28.175 17.475 25.615 1.00 0.00  O
ATOM  17 ND2 ASN  3  29.300 15.547 25.926 1.00 0.00  N
ATOM  18 C   ASN  3  26.520 18.066 27.661 1.00 0.00  C
ATOM  19 O   ASN  3  26.232 18.686 26.640 1.00 0.00  O
ATOM  20 N   TRP  4  25.645 17.303 28.319 1.00 0.00  N
ATOM  21 CA  TRP  4  24.277 17.148 27.870 1.00 0.00  C
ATOM  22 CB  TRP  4  23.495 16.226 28.799 1.00 0.00  C
ATOM  23 CG  TRP  4  22.053 16.002 28.413 1.00 0.00  C
ATOM  24 CD1 TRP  4  21.154 15.243 29.053 1.00 0.00  C
ATOM  25 NE1 TRP  4  19.928 15.336 28.321 1.00 0.00  N
ATOM  26 CE2 TRP  4  20.108 16.124 27.289 1.00 0.00  C
ATOM  27 CZ2 TRP  4  19.174 16.491 26.312 1.00 0.00  C
ATOM  28 CH2 TRP  4  19.635 17.347 25.321 1.00 0.00  C
ATOM  29 CZ3 TRP  4  20.912 17.786 25.319 1.00 0.00  C
ATOM  30 CE3 TRP  4  21.835 17.404 26.314 1.00 0.00  C
ATOM  31 CD2 TRP  4  21.370 16.541 27.314 1.00 0.00  C
ATOM  32 C   TRP  4  23.556 18.489 27.833 1.00 0.00  C
ATOM  33 O   TRP  4  22.862 18.797 26.867 1.00 0.00  O
ATOM  34 N   LEU  5  23.726 19.287 28.889 1.00 0.00  N
ATOM  35 CA  LEU  5  23.095 20.588 28.974 1.00 0.00  C
ATOM  36 CB  LEU  5  23.454 21.289 30.281 1.00 0.00  C
ATOM  37 CG  LEU  5  22.772 22.652 30.330 1.00 0.00  C
ATOM  38 CD1 LEU  5  21.260 22.467 30.252 1.00 0.00  C
ATOM  39 CD2 LEU  5  23.130 23.352 31.637 1.00 0.00  C
ATOM  40 C   LEU  5  23.536 21.487 27.828 1.00 0.00  C
ATOM  41 O   LEU  5  22.713 22.157 27.207 1.00 0.00  O
ATOM  42 N   LYS  6  24.842 21.501 27.548 1.00 0.00  N
ATOM  43 CA  LYS  6  25.387 22.316 26.480 1.00 0.00  C
ATOM  44 CB  LYS  6  26.898 22.144 26.377 1.00 0.00  C
ATOM  45 CG  LYS  6  27.433 23.015 25.245 1.00 0.00  C
ATOM  46 CD  LYS  6  28.945 22.844 25.142 1.00 0.00  C
ATOM  47 CE  LYS  6  29.479 23.715 24.009 1.00 0.00  C
ATOM  48 NZ  LYS  6  30.936 23.550 23.909 1.00 0.00  N1+
ATOM  49 C   LYS  6  24.783 21.934 25.137 1.00 0.00  C
ATOM  50 O   LYS  6  24.406 22.803 24.353 1.00 0.00  O
ATOM  51 N   LEU  7  24.691 20.629 24.872 1.00 0.00  N
ATOM  52 CA  LEU  7  24.135 20.137 23.627 1.00 0.00  C
ATOM  53 CB  LEU  7  24.150 18.613 23.583 1.00 0.00  C
ATOM  54 CG  LEU  7  23.557 18.137 22.261 1.00 0.00  C
ATOM  55 CD1 LEU  7  24.386 18.687 21.105 1.00 0.00  C
ATOM  56 CD2 LEU  7  23.571 16.613 22.217 1.00 0.00  C
ATOM  57 C   LEU  7  22.693 20.594 23.454 1.00 0.00  C
ATOM  58 O   LEU  7  22.306 21.040 22.376 1.00 0.00  O
ATOM  59 N   GLY  8  21.898 20.482 24.520 1.00 0.00  N
ATOM  60 CA  GLY  8  20.506 20.882 24.483 1.00 0.00  C
ATOM  61 C   GLY  8  20.388 22.361 24.143 1.00 0.00  C
ATOM  62 O   GLY  8  19.564 22.748 23.317 1.00 0.00  O
ATOM  63 N   LYS  9  21.215 23.189 24.785 1.00 0.00  N
ATOM  64 CA  LYS  9  21.202 24.619 24.551 1.00 0.00  C
ATOM  65 CB  LYS  9  22.244 25.326 25.410 1.00 0.00  C
ATOM  66 CG  LYS  9  22.197 26.826 25.134 1.00 0.00  C
ATOM  67 CD  LYS  9  23.239 27.533 25.995 1.00 0.00  C
ATOM  68 CE  LYS  9  23.193 29.033 25.718 1.00 0.00  C
ATOM  69 NZ  LYS  9  24.197 29.714 26.547 1.00 0.00  N1+
ATOM  70 C   LYS  9  21.506 24.938 23.094 1.00 0.00  C
ATOM  71 O   LYS  9  20.835 25.767 22.483 1.00 0.00  O
ATOM  72 N   MET 10  22.523 24.276 22.537 1.00 0.00  N
ATOM  73 CA  MET 10  22.913 24.491 21.158 1.00 0.00  C
ATOM  74 CB  MET 10  24.099 23.611 20.780 1.00 0.00  C
ATOM  75 CG  MET 10  24.477 23.866 19.324 1.00 0.00  C
ATOM  76 SD  MET 10  25.885 22.827 18.859 1.00 0.00  S
ATOM  77 CE  MET 10  26.092 23.328 17.163 1.00 0.00  C
ATOM  78 C   MET 10  21.769 24.164 20.208 1.00 0.00  C
ATOM  79 O   MET 10  21.495 24.919 19.278 1.00 0.00  O
ATOM  80 N   VAL 11  21.101 23.032 20.445 1.00 0.00  N
ATOM  81 CA  VAL 11  19.992 22.610 19.613 1.00 0.00  C
ATOM  82 CB  VAL 11  19.411 21.288 20.103 1.00 0.00  C
ATOM  83 CG1 VAL 11  18.911 21.452 21.536 1.00 0.00  C
ATOM  84 CG2 VAL 11  18.250 20.879 19.204 1.00 0.00  C
ATOM  85 C   VAL 11  18.874 23.642 19.624 1.00 0.00  C
ATOM  86 O   VAL 11  18.325 23.978 18.577 1.00 0.00  O
ATOM  87 N   ILE 12  18.538 24.147 20.814 1.00 0.00  N
ATOM  88 CA  ILE 12  17.489 25.136 20.958 1.00 0.00  C
ATOM  89 CB  ILE 12  17.342 25.512 22.428 1.00 0.00  C
ATOM  90 CG2 ILE 12  18.659 26.087 22.940 1.00 0.00  C
ATOM  91 CG1 ILE 12  16.239 26.554 22.579 1.00 0.00  C
ATOM  92 CD1 ILE 12  16.091 26.928 24.050 1.00 0.00  C
ATOM  93 C   ILE 12  17.812 26.395 20.164 1.00 0.00  C
ATOM  94 O   ILE 12  16.950 26.934 19.472 1.00 0.00  O
ATOM  95 N   ASP 13  19.057 26.862 20.265 1.00 0.00  N
ATOM  96 CA  ASP 13  19.489 28.052 19.559 1.00 0.00  C
ATOM  97 CB  ASP 13  20.953 28.363 19.850 1.00 0.00  C
ATOM  98 CG  ASP 13  21.368 29.620 19.090 1.00 0.00  C
ATOM  99 OD1 ASP 13  21.217 30.724 19.677 1.00 0.00  O
ATOM 100 OD2 ASP 13  21.831 29.465 17.928 1.00 0.00  O1-
ATOM 101 C   ASP 13  19.340 27.880 18.054 1.00 0.00  C
ATOM 102 O   ASP 13  18.850 28.776 17.370 1.00 0.00  O
ATOM 103 N   ALA 14  19.765 26.724 17.539 1.00 0.00  N
ATOM 104 CA  ALA 14  19.677 26.439 16.121 1.00 0.00  C
ATOM 105 CB  ALA 14  20.230 25.054 15.804 1.00 0.00  C
ATOM 106 C   ALA 14  18.234 26.485 15.642 1.00 0.00  C
ATOM 107 O   ALA 14  17.941 27.067 14.598 1.00 0.00  O
ATOM 108 N   LEU 15  17.330 25.867 16.405 1.00 0.00  N
ATOM 109 CA  LEU 15  15.923 25.839 16.056 1.00 0.00  C
ATOM 110 CB  LEU 15  15.116 25.071 17.098 1.00 0.00  C
ATOM 111 CG  LEU 15  13.645 25.064 16.694 1.00 0.00  C
ATOM 112 CD1 LEU 15  13.492 24.391 15.335 1.00 0.00  C
ATOM 113 CD2 LEU 15  12.836 24.296 17.734 1.00 0.00  C
ATOM 114 C   LEU 15  15.352 27.248 15.971 1.00 0.00  C
ATOM 115 O   LEU 15  16.066 28.224 16.186 1.00 0.00  O
ATOM 116 N   NHE 16  14.059 27.349 15.655 1.00 0.00  N
TER  117     NHE 16
```

ID2 extended

```
ATOM 1 CH3 ACE 1 13.222 26.065 31.411 1.00 0.00 C
ATOM 2 C ACE 1 14.552 26.714 31.111 1.00 0.00 C
ATOM 3 O ACE 1 14.553 27.931 31.039 1.00 0.00 O
ATOM 4 N THR 2 15.678 25.915 30.996 1.00 0.00 N
ATOM 5 CA THR 2 16.998 26.489 31.117 1.00 0.00 C
ATOM 6 CB THR 2 17.772 25.953 32.302 1.00 0.00 C
ATOM 7 CG2 THR 2 17.217 26.475 33.624 1.00 0.00 C
ATOM 8 OG1 THR 2 17.722 24.554 32.326 1.00 0.00 O
ATOM 9 C THR 2 17.865 26.303 29.812 1.00 0.00 C
ATOM 10 O THR 2 19.035 26.728 29.766 1.00 0.00 O
ATOM 11 N ARG 3 17.294 25.576 28.836 1.00 0.00 N
ATOM 12 CA ARG 3 17.885 25.182 27.572 1.00 0.00 C
ATOM 13 CB ARG 3 16.815 24.603 26.546 1.00 0.00 C
ATOM 14 CG ARG 3 17.129 24.545 25.064 1.00 0.00 C
ATOM 15 CD ARG 3 16.049 23.762 24.268 1.00 0.00 C
ATOM 16 NE ARG 3 16.149 23.956 22.804 1.00 0.00 N
ATOM 17 CZ ARG 3 15.218 23.928 21.920 1.00 0.00 C
ATOM 18 NH1 ARG 3 14.077 23.420 22.201 1.00 0.00 N
ATOM 19 NH2 ARG 3 15.457 24.172 20.636 1.00 0.00 N1+
ATOM 20 C ARG 3 18.688 26.255 26.904 1.00 0.00 C
ATOM 21 O ARG 3 19.667 25.948 26.143 1.00 0.00 O
ATOM 22 N THR 4 18.323 27.466 27.025 1.00 0.00 N
ATOM 23 CA THR 4 18.887 28.685 26.440 1.00 0.00 C
ATOM 24 CB THR 4 18.017 29.900 26.599 1.00 0.00 C
ATOM 25 CG2 THR 4 16.758 29.621 25.755 1.00 0.00 C
ATOM 26 OG1 THR 4 17.703 30.140 27.949 1.00 0.00 O
ATOM 27 C THR 4 20.275 29.015 26.903 1.00 0.00 C
ATOM 28 O THR 4 20.959 29.780 26.268 1.00 0.00 O
ATOM 29 N LYS 5 20.761 28.396 28.041 1.00 0.00 N
ATOM 30 CA LYS 5 22.203 28.378 28.431 1.00 0.00 C
ATOM 31 CB LYS 5 22.211 27.615 29.805 1.00 0.00 C
ATOM 32 CG LYS 5 23.558 27.723 30.610 1.00 0.00 C
ATOM 33 CD LYS 5 23.450 26.878 31.862 1.00 0.00 C
ATOM 34 CE LYS 5 24.629 27.098 32.825 1.00 0.00 C
ATOM 35 NZ LYS 5 24.242 26.493 34.084 1.00 0.00 N1+
ATOM 36 C LYS 5 23.149 27.648 27.436 1.00 0.00 C
ATOM 37 O LYS 5 24.301 28.047 27.416 1.00 0.00 O
ATOM 38 N ILE 6 22.689 26.587 26.722 1.00 0.00 N
ATOM 39 CA ILE 6 23.528 25.672 25.937 1.00 0.00 C
ATOM 40 CB ILE 6 23.894 24.424 26.679 1.00 0.00 C
ATOM 41 CG2 ILE 6 24.869 24.742 27.824 1.00 0.00 C
ATOM 42 CG1 ILE 6 22.648 23.565 27.082 1.00 0.00 C
ATOM 43 CD1 ILE 6 22.973 22.344 27.961 1.00 0.00 C
ATOM 44 C ILE 6 23.013 25.336 24.458 1.00 0.00 C
ATOM 45 O ILE 6 23.640 24.680 23.647 1.00 0.00 O
ATOM 46 N ASP 7 21.852 25.897 24.093 1.00 0.00 N
ATOM 47 CA ASP 7 21.087 25.653 22.851 1.00 0.00 C
ATOM 48 CB ASP 7 19.771 26.458 22.915 1.00 0.00 C
ATOM 49 CG ASP 7 18.715 26.129 21.862 1.00 0.00 C
ATOM 50 OD1 ASP 7 18.220 27.031 21.191 1.00 0.00 O
ATOM 51 OD2 ASP 7 18.459 24.927 21.719 1.00 0.00 O1-
ATOM 52 C ASP 7 21.915 26.000 21.564 1.00 0.00 C
ATOM 53 O ASP 7 21.654 25.472 20.466 1.00 0.00 O
ATOM 54 N TRP 8 22.873 26.897 21.686 1.00 0.00 N
ATOM 55 CA TRP 8 23.740 27.500 20.661 1.00 0.00 C
ATOM 56 CB TRP 8 24.375 28.799 21.235 1.00 0.00 C
ATOM 57 CG TRP 8 24.988 28.704 22.611 1.00 0.00 C
ATOM 58 CD1 TRP 8 24.302 28.873 23.796 1.00 0.00 C
ATOM 59 NE1 TRP 8 25.204 28.736 24.869 1.00 0.00 N
ATOM 60 CE2 TRP 8 26.508 28.604 24.468 1.00 0.00 C
ATOM 61 CZ2 TRP 8 27.720 28.626 25.174 1.00 0.00 C
ATOM 62 CH2 TRP 8 28.897 28.381 24.433 1.00 0.00 C
ATOM 63 CZ3 TRP 8 28.883 28.375 23.002 1.00 0.00 C
ATOM 64 CE3 TRP 8 27.612 28.428 22.276 1.00 0.00 C
ATOM 65 CD2 TRP 8 26.394 28.540 23.036 1.00 0.00 C
ATOM 66 C TRP 8 24.820 26.595 20.048 1.00 0.00 C
ATOM 67 O TRP 8 25.238 26.937 18.981 1.00 0.00 O
ATOM 68 N ASN 9 25.169 25.435 20.597 1.00 0.00 N
ATOM 69 CA ASN 9 26.307 24.535 20.294 1.00 0.00 C
ATOM 70 CB ASN 9 27.209 24.355 21.513 1.00 0.00 C
ATOM 71 CG ASN 9 28.647 23.745 21.302 1.00 0.00 C
ATOM 72 OD1 ASN 9 28.811 22.921 20.381 1.00 0.00 O
ATOM 73 ND2 ASN 9 29.622 24.022 22.161 1.00 0.00 N
ATOM 74 C ASN 9 25.772 23.226 19.765 1.00 0.00 C
ATOM 75 O ASN 9 24.670 22.820 20.075 1.00 0.00 O
ATOM 76 N LYS 10 26.376 22.549 18.739 1.00 0.00 N
ATOM 77 CA LYS 10 25.872 21.387 18.027 1.00 0.00 C
ATOM 78 CB LYS 10 26.685 21.301 16.726 1.00 0.00 C
ATOM 79 CG LYS 10 26.429 22.467 15.736 1.00 0.00 C
ATOM 80 CD LYS 10 24.960 22.724 15.329 1.00 0.00 C
ATOM 81 CE LYS 10 25.086 23.323 13.904 1.00 0.00 C
ATOM 82 NZ LYS 10 23.880 23.605 13.058 1.00 0.00 N1+
ATOM 83 C LYS 10 25.908 20.066 18.812 1.00 0.00 C
ATOM 84 O LYS 10 25.331 19.126 18.305 1.00 0.00 O
ATOM 85 N ILE 11 26.463 20.017 20.019 1.00 0.00 N
ATOM 86 CA ILE 11 26.342 18.857 20.958 1.00 0.00 C
ATOM 87 CB ILE 11 27.651 17.950 20.966 1.00 0.00 C
ATOM 88 CG2 ILE 11 27.935 17.430 19.542 1.00 0.00 C
ATOM 89 CG1 ILE 11 28.840 18.735 21.601 1.00 0.00 C
ATOM 90 CD1 ILE 11 30.112 17.953 21.669 1.00 0.00 C
ATOM 91 C ILE 11 26.049 19.145 22.406 1.00 0.00 C
ATOM 92 O ILE 11 26.286 20.216 22.931 1.00 0.00 O
ATOM 93 N LEU 12 25.620 18.137 23.159 1.00 0.00 N
ATOM 94 CA LEU 12 25.164 18.135 24.560 1.00 0.00 C
ATOM 95 CB LEU 12 26.412 18.148 25.518 1.00 0.00 C
ATOM 96 CG LEU 12 27.374 16.945 25.295 1.00 0.00 C
ATOM 97 CD1 LEU 12 28.547 16.799 26.319 1.00 0.00 C
ATOM 98 CD2 LEU 12 26.602 15.719 25.527 1.00 0.00 C
ATOM 99 C LEU 12 24.055 19.205 24.823 1.00 0.00 C
ATOM 100 O LEU 12 24.036 19.711 25.916 1.00 0.00 O
ATOM 101 N SER 13 23.197 19.391 23.849 1.00 0.00 N
ATOM 102 CA SER 13 22.150 20.461 23.861 1.00 0.00 C
ATOM 103 CB SER 13 22.482 21.594 22.955 1.00 0.00 C
ATOM 104 OG SER 13 22.939 21.051 21.715 1.00 0.00 O
ATOM 105 C SER 13 20.741 19.884 23.559 1.00 0.00 C
ATOM 106 O SER 13 19.835 20.665 23.294 1.00 0.00 O
ATOM 107 N NHE 14 20.601 18.541 23.322 1.00 0.00 N
TER 108 NHE 14
```

ID2 misfolded

```
ATOM 1 CH3 ACE 1 23.745 18.437 32.753 1.00 0.00 C
ATOM 2 C ACE 1 24.121 19.761 32.084 1.00 0.00 C
ATOM 3 O ACE 1 24.322 20.768 32.761 1.00 0.00 O
ATOM 4 N THR 2 24.212 19.756 30.774 1.00 0.00 N
ATOM 5 CA THR 2 24.561 20.953 30.034 1.00 0.00 C
ATOM 6 CB THR 2 25.934 21.473 30.449 1.00 0.00 C
ATOM 7 CG2 THR 2 25.923 21.804 31.938 1.00 0.00 C
ATOM 8 OG1 THR 2 26.922 20.471 30.191 1.00 0.00 O
ATOM 9 C THR 2 24.600 20.681 28.538 1.00 0.00 C
ATOM 10 O THR 2 24.077 21.464 27.748 1.00 0.00 O
ATOM 11 N ARG 3 25.222 19.566 28.148 1.00 0.00 N
ATOM 12 CA ARG 3 25.327 19.196 26.751 1.00 0.00 C
ATOM 13 CB ARG 3 26.084 17.882 26.587 1.00 0.00 C
ATOM 14 CG ARG 3 26.172 17.529 25.106 1.00 0.00 C
ATOM 15 CD ARG 3 26.930 16.215 24.942 1.00 0.00 C
ATOM 16 NE ARG 3 27.035 15.838 23.515 1.00 0.00 N
ATOM 17 CZ ARG 3 27.649 14.735 23.100 1.00 0.00 C
ATOM 18 NH1 ARG 3 28.217 13.883 23.949 1.00 0.00 N
ATOM 19 NH2 ARG 3 27.685 14.493 21.792 1.00 0.00 N1+
ATOM 20 C ARG 3 23.950 19.021 26.127 1.00 0.00 C
ATOM 21 O ARG 3 23.700 19.500 25.023 1.00 0.00 O
ATOM 22 N THR 4 23.055 18.329 26.837 1.00 0.00 N
ATOM 23 CA THR 4 21.711 18.093 26.351 1.00 0.00 C
ATOM 24 CB THR 4 20.899 17.281 27.355 1.00 0.00 C
ATOM 25 CG2 THR 4 21.578 15.935 27.586 1.00 0.00 C
ATOM 26 OG1 THR 4 20.821 17.994 28.593 1.00 0.00 O
ATOM 27 C THR 4 20.976 19.405 26.113 1.00 0.00 C
ATOM 28 O THR 4 20.322 19.575 25.086 1.00 0.00 O
ATOM 29 N LYS 5 21.086 20.333 27.066 1.00 0.00 N
ATOM 30 CA LYS 5 20.434 21.622 26.956 1.00 0.00 C
ATOM 31 CB LYS 5 20.723 22.489 28.178 1.00 0.00 C
ATOM 32 CG LYS 5 20.021 23.834 28.024 1.00 0.00 C
ATOM 33 CD LYS 5 20.310 24.701 29.246 1.00 0.00 C
ATOM 34 CE LYS 5 19.609 26.046 29.093 1.00 0.00 C
ATOM 35 NZ LYS 5 19.888 26.881 30.271 1.00 0.00 N1+
ATOM 36 C LYS 5 20.916 22.378 25.726 1.00 0.00 C
ATOM 37 O LYS 5 20.114 22.948 24.990 1.00 0.00 O
ATOM 38 N ILE 6 22.232 22.381 25.504 1.00 0.00 N
ATOM 39 CA ILE 6 22.816 23.065 24.367 1.00 0.00 C
ATOM 40 CB ILE 6 24.330 22.887 24.389 1.00 0.00 C
ATOM 41 CG2 ILE 6 24.668 21.402 24.319 1.00 0.00 C
ATOM 42 CG1 ILE 6 24.944 23.606 23.193 1.00 0.00 C
ATOM 43 CD1 ILE 6 26.459 23.428 23.215 1.00 0.00 C
ATOM 44 C ILE 6 22.279 22.505 23.058 1.00 0.00 C
ATOM 45 O ILE 6 21.927 23.261 22.154 1.00 0.00 O
ATOM 46 N ASP 7 22.217 21.175 22.957 1.00 0.00 N
ATOM 47 CA ASP 7 21.725 20.520 21.761 1.00 0.00 C
ATOM 48 CB ASP 7 21.762 19.003 21.912 1.00 0.00 C
ATOM 49 CG ASP 7 21.237 18.352 20.634 1.00 0.00 C
ATOM 50 OD1 ASP 7 22.078 18.090 19.734 1.00 0.00 O
ATOM 51 OD2 ASP 7 19.999 18.123 20.571 1.00 0.00 O1-
ATOM 52 C ASP 7 20.287 20.924 21.468 1.00 0.00 C
ATOM 53 O ASP 7 19.944 21.223 20.326 1.00 0.00 O
ATOM 54 N TRP 8 19.445 20.932 22.504 1.00 0.00 N
ATOM 55 CA TRP 8 18.051 21.298 22.355 1.00 0.00 C
ATOM 56 CB TRP 8 17.318 21.223 23.690 1.00 0.00 C
ATOM 57 CG TRP 8 15.854 21.587 23.636 1.00 0.00 C
ATOM 58 CD1 TRP 8 14.988 21.604 24.659 1.00 0.00 C
ATOM 59 NE1 TRP 8 13.724 22.010 24.130 1.00 0.00 N
ATOM 60 CE2 TRP 8 13.848 22.223 22.842 1.00 0.00 C
ATOM 61 CZ2 TRP 8 12.862 22.633 21.937 1.00 0.00 C
ATOM 62 CH2 TRP 8 13.271 22.784 20.617 1.00 0.00 C
ATOM 63 CZ3 TRP 8 14.548 22.543 20.253 1.00 0.00 C
ATOM 64 CE3 TRP 8 15.526 22.131 21.183 1.00 0.00 C
ATOM 65 CD2 TRP 8 15.113 21.979 22.513 1.00 0.00 C
ATOM 66 C TRP 8 17.910 22.718 21.826 1.00 0.00 C
ATOM 67 O TRP 8 17.115 22.972 20.926 1.00 0.00 O
ATOM 68 N ASN 9 18.688 23.645 22.390 1.00 0.00 N
ATOM 69 CA ASN 9 18.648 25.033 21.975 1.00 0.00 C
ATOM 70 CB ASN 9 19.634 25.875 22.779 1.00 0.00 C
ATOM 71 CG ASN 9 19.593 27.332 22.344 1.00 0.00 C
ATOM 72 OD1 ASN 9 18.831 27.695 21.449 1.00 0.00 O
ATOM 73 ND2 ASN 9 20.416 28.170 22.978 1.00 0.00 N
ATOM 74 C ASN 9 19.008 25.175 20.503 1.00 0.00 C
ATOM 75 O ASN 9 18.343 25.901 19.766 1.00 0.00 O
ATOM 76 N LYS 10 20.065 24.480 20.076 1.00 0.00 N
ATOM 77 CA LYS 10 20.508 24.532 18.697 1.00 0.00 C
ATOM 78 CB LYS 10 21.731 23.647 18.481 1.00 0.00 C
ATOM 79 CG LYS 10 22.166 23.728 17.021 1.00 0.00 C
ATOM 80 CD LYS 10 23.389 22.844 16.805 1.00 0.00 C
ATOM 81 CE LYS 10 23.823 22.925 15.345 1.00 0.00 C
ATOM 82 NZ LYS 10 25.002 22.073 15.136 1.00 0.00 N1+
ATOM 83 C LYS 10 19.415 24.053 17.752 1.00 0.00 C
ATOM 84 O LYS 10 19.162 24.676 16.724 1.00 0.00 O
ATOM 85 N ILE 11 18.766 22.940 18.104 1.00 0.00 N
ATOM 86 CA ILE 11 17.705 22.383 17.289 1.00 0.00 C
ATOM 87 CB ILE 11 17.165 21.121 17.953 1.00 0.00 C
ATOM 88 CG2 ILE 11 16.616 21.467 19.333 1.00 0.00 C
ATOM 89 CG1 ILE 11 16.048 20.534 17.095 1.00 0.00 C
ATOM 90 CD1 ILE 11 15.507 19.272 17.759 1.00 0.00 C
ATOM 91 C ILE 11 16.561 23.374 17.125 1.00 0.00 C
ATOM 92 O ILE 11 16.050 23.559 16.023 1.00 0.00 O
ATOM 93 N LEU 12 16.161 24.014 18.226 1.00 0.00 N
ATOM 94 CA LEU 12 15.084 24.981 18.201 1.00 0.00 C
ATOM 95 CB LEU 12 14.832 25.559 19.590 1.00 0.00 C
ATOM 96 CG LEU 12 13.692 26.570 19.520 1.00 0.00 C
ATOM 97 CD1 LEU 12 12.429 25.879 19.020 1.00 0.00 C
ATOM 98 CD2 LEU 12 13.440 27.147 20.909 1.00 0.00 C
ATOM 99 C LEU 12 15.408 26.138 17.268 1.00 0.00 C
ATOM 100 O LEU 12 14.565 26.560 16.480 1.00 0.00 O
ATOM 101 N SER 13 16.637 26.653 17.358 1.00 0.00 N
ATOM 102 CA SER 13 17.068 27.758 16.524 1.00 0.00 C
ATOM 103 CB SER 13 18.510 28.146 16.832 1.00 0.00 C
ATOM 104 OG SER 13 18.899 29.236 15.992 1.00 0.00 O
ATOM 105 C SER 13 16.988 27.393 15.048 1.00 0.00 C
ATOM 106 O SER 13 16.596 26.282 14.701 1.00 0.00 O
ATOM 107 N NHE 14 17.359 28.328 14.175 1.00 0.00 N
TER 108 NHE 14
```

ID3 extended

```
ATOM  1 CH3 ACE 1  17.825  17.213  28.169  1.00  0.00  C
ATOM  2 C   ACE 1  18.367  18.323  27.318  1.00  0.00  C
ATOM  3 O   ACE 1  19.273  19.060  27.703  1.00  0.00  O
ATOM  4 N   SER 2  17.788  18.481  26.134  1.00  0.00  N
ATOM  5 CA  SER 2  18.089  19.569  25.113  1.00  0.00  C
ATOM  6 CB  SER 2  17.361  20.849  25.531  1.00  0.00  C
ATOM  7 OG  SER 2  16.024  20.651  25.918  1.00  0.00  O
ATOM  8 C   SER 2  19.528  19.905  24.749  1.00  0.00  C
ATOM  9 O   SER 2  19.853  21.071  24.400  1.00  0.00  O
ATOM  10 N  THR 3  20.431  18.979  24.992  1.00  0.00  N
ATOM  11 CA THR 3  21.866  19.085  24.794  1.00  0.00  C
ATOM  12 CB THR 3  22.591  17.842  25.360  1.00  0.00  C
ATOM  13 CG2 THR 3  23.126  18.165  26.781  1.00  0.00  C
ATOM  14 OG1 THR 3  21.627  16.762  25.474  1.00  0.00  O
ATOM  15 C  THR 3  22.215  19.193  23.333  1.00  0.00  C
ATOM  16 O  THR 3  23.247  19.723  22.980  1.00  0.00  O
ATOM  17 N  SER 4  21.266  18.785  22.476  1.00  0.00  N
ATOM  18 CA SER 4  21.449  18.876  21.020  1.00  0.00  C
ATOM  19 CB SER 4  21.109  17.517  20.406  1.00  0.00  C
ATOM  20 OG SER 4  19.802  17.093  20.777  1.00  0.00  O
ATOM  21 C  SER 4  20.653  20.077  20.462  1.00  0.00  C
ATOM  22 O  SER 4  20.945  20.482  19.327  1.00  0.00  O
ATOM  23 N  ARG 5  19.615  20.624  21.178  1.00  0.00  N
ATOM  24 CA ARG 5  18.691  21.682  20.688  1.00  0.00  C
ATOM  25 CB ARG 5  17.295  21.468  21.069  1.00  0.00  C
ATOM  26 CG ARG 5  16.512  20.442  20.216  1.00  0.00  C
ATOM  27 CD ARG 5  15.100  20.208  20.833  1.00  0.00  C
ATOM  28 NE ARG 5  14.450  19.116  20.129  1.00  0.00  N
ATOM  29 CZ ARG 5  13.493  18.323  20.541  1.00  0.00  C
ATOM  30 NH1 ARG 5  12.903  18.431  21.677  1.00  0.00  N
ATOM  31 NH2 ARG 5  13.010  17.245  19.861  1.00  0.00  N1+
ATOM  32 C  ARG 5  19.138  23.092  21.072  1.00  0.00  C
ATOM  33 O  ARG 5  19.426  23.902  20.143  1.00  0.00  O
ATOM  34 N  HIE 6  19.230  23.412  22.374  1.00  0.00  N
ATOM  35 CA HIE 6  19.394  24.748  22.894  1.00  0.00  C
ATOM  36 CB HIE 6  18.519  24.806  24.162  1.00  0.00  C
ATOM  37 CG HIE 6  17.049  24.720  23.999  1.00  0.00  C
ATOM  38 ND1 HIE 6  16.295  23.735  24.717  1.00  0.00  N
ATOM  39 CE1 HIE 6  15.016  24.021  24.611  1.00  0.00  C
ATOM  40 NE2 HIE 6  14.921  25.050  23.709  1.00  0.00  N
ATOM  41 CD2 HIE 6  16.184  25.595  23.404  1.00  0.00  C
ATOM  42 C  HIE 6  20.799  25.127  23.394  1.00  0.00  C
ATOM  43 O  HIE 6  21.086  26.286  23.405  1.00  0.00  O
ATOM  44 N  LYS 7  21.643  24.108  23.745  1.00  0.00  N
ATOM  45 CA LYS 7  22.960  24.275  24.427  1.00  0.00  C
ATOM  46 CB LYS 7  23.188  23.232  25.466  1.00  0.00  C
ATOM  47 CG LYS 7  22.075  23.266  26.560  1.00  0.00  C
ATOM  48 CD LYS 7  22.225  22.248  27.736  1.00  0.00  C
ATOM  49 CE LYS 7  20.904  21.978  28.526  1.00  0.00  C
ATOM  50 NZ LYS 7  21.093  20.949  29.594  1.00  0.00  N1+
ATOM  51 C  LYS 7  24.141  24.523  23.433  1.00  0.00  C
ATOM  52 O  LYS 7  25.279  24.373  23.894  1.00  0.00  O
ATOM  53 N  LYS 8  23.834  24.967  22.184  1.00  0.00  N
ATOM  54 CA LYS 8  24.843  25.137  21.104  1.00  0.00  C
ATOM  55 CB LYS 8  24.149  25.197  19.752  1.00  0.00  C
ATOM  56 CG LYS 8  23.241  23.975  19.346  1.00  0.00  C
ATOM  57 CD LYS 8  22.740  24.293  17.972  1.00  0.00  C
ATOM  58 CE LYS 8  23.720  23.958  16.936  1.00  0.00  C
ATOM  59 NZ LYS 8  23.204  23.661  15.584  1.00  0.00  N1+
ATOM  60 C  LYS 8  25.702  26.401  21.164  1.00  0.00  C
ATOM  61 O  LYS 8  25.267  27.356  21.819  1.00  0.00  O
ATOM  62 N  LEU 9  26.882  26.396  20.613  1.00  0.00  N
ATOM  63 CA LEU 9  28.032  27.280  21.042  1.00  0.00  C
ATOM  64 CB LEU 9  29.303  26.952  20.149  1.00  0.00  C
ATOM  65 CG LEU 9  30.503  27.793  20.655  1.00  0.00  C
ATOM  66 CD1 LEU 9  30.931  27.435  22.109  1.00  0.00  C
ATOM  67 CD2 LEU 9  31.721  27.497  19.770  1.00  0.00  C
ATOM  68 C  LEU 9  27.577  28.760  21.024  1.00  0.00  C
ATOM  69 O  LEU 9  27.792  29.558  21.964  1.00  0.00  O
ATOM  70 N  MET 10  26.896  29.133  19.947  1.00  0.00  N
ATOM  71 CA MET 10  26.634  30.548  19.704  1.00  0.00  C
ATOM  72 CB MET 10  26.488  30.744  18.194  1.00  0.00  C
ATOM  73 CG MET 10  25.200  30.518  17.482  1.00  0.00  C
ATOM  74 SD MET 10  24.587  28.810  17.748  1.00  0.00  S
ATOM  75 CE MET 10  23.371  28.664  16.409  1.00  0.00  C
ATOM  76 C  MET 10  25.470  31.133  20.481  1.00  0.00  C
ATOM  77 O  MET 10  25.203  32.311  20.464  1.00  0.00  O
ATOM  78 N  PHE 11  24.745  30.232  21.208  1.00  0.00  N
ATOM  79 CA PHE 11  23.726  30.528  22.167  1.00  0.00  C
ATOM  80 CB PHE 11  22.539  29.663  21.832  1.00  0.00  C
ATOM  81 CG PHE 11  21.243  30.054  22.546  1.00  0.00  C
ATOM  82 CD1 PHE 11  20.920  29.527  23.863  1.00  0.00  C
ATOM  83 CE1 PHE 11  19.755  29.887  24.482  1.00  0.00  C
ATOM  84 CZ PHE 11  18.859  30.773  23.878  1.00  0.00  C
ATOM  85 CE2 PHE 11  19.090  31.236  22.541  1.00  0.00  C
ATOM  86 CD2 PHE 11  20.331  30.956  21.957  1.00  0.00  C
ATOM  87 C  PHE 11  24.206  30.442  23.606  1.00  0.00  C
ATOM  88 O  PHE 11  23.583  31.042  24.506  1.00  0.00  O
ATOM  89 N  LYS 12  25.365  29.853  23.914  1.00  0.00  N
ATOM  90 CA LYS 12  25.798  29.491  25.267  1.00  0.00  C
ATOM  91 CB LYS 12  27.080  28.659  25.129  1.00  0.00  C
ATOM  92 CG LYS 12  27.297  27.829  26.408  1.00  0.00  C
ATOM  93 CD LYS 12  28.567  27.006  26.466  1.00  0.00  C
ATOM  94 CE LYS 12  28.902  26.684  27.946  1.00  0.00  C
ATOM  95 NZ LYS 12  29.613  25.394  28.181  1.00  0.00  N1+
ATOM  96 C  LYS 12  25.980  30.760  26.136  1.00  0.00  C
ATOM  97 O  LYS 12  25.916  30.586  27.324  1.00  0.00  O
ATOM  98 N  THR 13  26.226  31.953  25.611  1.00  0.00  N
ATOM  99 CA THR 13  26.376  33.166  26.370  1.00  0.00  C
ATOM  100 CB THR 13  27.227  34.110  25.537  1.00  0.00  C
ATOM  101 CG2 THR 13  28.649  33.656  25.380  1.00  0.00  C
ATOM  102 OG1 THR 13  26.568  34.274  24.233  1.00  0.00  O
ATOM  103 C  THR 13  25.113  33.969  26.795  1.00  0.00  C
ATOM  104 O  THR 13  25.074  34.910  27.567  1.00  0.00  O
ATOM  105 N  GLU 14  23.939  33.577  26.210  1.00  0.00  N
ATOM  106 CA GLU 14  22.591  34.079  26.338  1.00  0.00  C
ATOM  107 CB GLU 14  21.808  34.089  24.957  1.00  0.00  C
ATOM  108 CG GLU 14  22.294  35.114  23.989  1.00  0.00  C
ATOM  109 CD GLU 14  21.991  36.521  24.361  1.00  0.00  C
ATOM  110 OE1 GLU 14  22.829  37.437  24.043  1.00  0.00  O
ATOM  111 OE2 GLU 14  20.912  36.883  24.902  1.00  0.00  O1-
ATOM  112 C  GLU 14  21.882  33.337  27.513  1.00  0.00  C
ATOM  113 O  GLU 14  22.346  32.349  28.109  1.00  0.00  O
ATOM  114 N  NHE 15  20.688  33.737  27.917  1.00  0.00  N
TER  115  NHE 15
```

ID3 misfolded

```
ATOM 1 CH3 ACE 1 27.050 19.552 32.630 1.00 0.00 C
ATOM 2 C ACE 1 27.280 19.635 31.120 1.00 0.00 C
ATOM 3 O ACE 1 26.614 20.402 30.428 1.00 0.00 O
ATOM 4 N SER 2 28.227 18.840 30.616 1.00 0.00 N
ATOM 5 CA SER 2 28.541 18.826 29.201 1.00 0.00 C
ATOM 6 CB SER 2 29.657 17.831 28.896 1.00 0.00 C
ATOM 7 OG SER 2 29.938 17.845 27.494 1.00 0.00 O
ATOM 8 C SER 2 27.325 18.429 28.375 1.00 0.00 C
ATOM 9 O SER 2 27.032 19.053 27.357 1.00 0.00 O
ATOM 10 N THR 3 26.618 17.385 28.815 1.00 0.00 N
ATOM 11 CA THR 3 25.442 16.910 28.117 1.00 0.00 C
ATOM 12 CB THR 3 24.826 15.711 28.831 1.00 0.00 C
ATOM 13 CG2 THR 3 25.846 14.579 28.895 1.00 0.00 C
ATOM 14 OG1 THR 3 24.450 16.089 30.159 1.00 0.00 O
ATOM 15 C THR 3 24.378 17.996 28.032 1.00 0.00 C
ATOM 16 O THR 3 23.783 18.205 26.977 1.00 0.00 O
ATOM 17 N SER 4 24.141 18.688 29.148 1.00 0.00 N
ATOM 18 CA SER 4 23.154 19.748 29.196 1.00 0.00 C
ATOM 19 CB SER 4 23.085 20.371 30.587 1.00 0.00 C
ATOM 20 OG SER 4 22.103 21.411 30.594 1.00 0.00 O
ATOM 21 C SER 4 23.488 20.854 28.206 1.00 0.00 C
ATOM 22 O SER 4 22.614 21.335 27.489 1.00 0.00 O
ATOM 23 N ARG 5 24.760 21.258 28.169 1.00 0.00 N
ATOM 24 CA ARG 5 25.205 22.304 27.269 1.00 0.00 C
ATOM 25 CB ARG 5 26.698 22.569 27.432 1.00 0.00 C
ATOM 26 CG ARG 5 27.126 23.672 26.470 1.00 0.00 C
ATOM 27 CD ARG 5 28.619 23.937 26.632 1.00 0.00 C
ATOM 28 NE ARG 5 29.072 25.005 25.712 1.00 0.00 N
ATOM 29 CZ ARG 5 30.332 25.426 25.655 1.00 0.00 C
ATOM 30 NH1 ARG 5 31.283 24.911 26.429 1.00 0.00 N
ATOM 31 NH2 ARG 5 30.628 26.393 24.792 1.00 0.00 N1+
ATOM 32 C ARG 5 24.956 21.921 25.818 1.00 0.00 C
ATOM 33 O ARG 5 24.470 22.733 25.033 1.00 0.00 O
ATOM 34 N HIP 6 25.290 20.679 25.462 1.00 0.00 N
ATOM 35 CA HIP 6 25.101 20.193 24.109 1.00 0.00 C
ATOM 36 CB HIP 6 25.565 18.747 23.976 1.00 0.00 C
ATOM 37 CG HIP 6 25.408 18.151 22.598 1.00 0.00 C
ATOM 38 ND1 HIP 6 25.784 16.844 22.313 1.00 0.00 N1+
ATOM 39 CE1 HIP 6 25.528 16.613 21.039 1.00 0.00 C
ATOM 40 NE2 HIP 6 25.015 17.694 20.504 1.00 0.00 N
ATOM 41 CD2 HIP 6 24.923 18.676 21.441 1.00 0.00 C
ATOM 42 C HIP 6 23.635 20.251 23.706 1.00 0.00 C
ATOM 43 O HIP 6 23.308 20.689 22.605 1.00 0.00 O
ATOM 44 N LYS 7 22.751 19.807 24.601 1.00 0.00 N
ATOM 45 CA LYS 7 21.326 19.809 24.337 1.00 0.00 C
ATOM 46 CB LYS 7 20.546 19.251 25.523 1.00 0.00 C
ATOM 47 CG LYS 7 19.055 19.271 25.205 1.00 0.00 C
ATOM 48 CD LYS 7 18.275 18.712 26.392 1.00 0.00 C
ATOM 49 CE LYS 7 16.784 18.732 26.075 1.00 0.00 C
ATOM 50 NZ LYS 7 16.032 18.194 27.218 1.00 0.00 N1+
ATOM 51 C LYS 7 20.820 21.220 24.070 1.00 0.00 C
ATOM 52 O LYS 7 20.056 21.442 23.135 1.00 0.00 O
ATOM 53 N LYS 8 21.250 22.175 24.898 1.00 0.00 N
ATOM 54 CA LYS 8 20.841 23.557 24.750 1.00 0.00 C
ATOM 55 CB LYS 8 21.476 24.436 25.823 1.00 0.00 C
ATOM 56 CG LYS 8 21.023 25.879 25.632 1.00 0.00 C
ATOM 57 CD LYS 8 21.658 26.757 26.706 1.00 0.00 C
ATOM 58 CE LYS 8 21.203 28.201 26.516 1.00 0.00 C
ATOM 59 NZ LYS 8 21.815 29.047 27.549 1.00 0.00 N1+
ATOM 60 C LYS 8 21.256 24.109 23.393 1.00 0.00 C
ATOM 61 O LYS 8 20.467 24.772 22.722 1.00 0.00 O
ATOM 62 N LEU 9 22.498 23.833 22.989 1.00 0.00 N
ATOM 63 CA LEU 9 23.011 24.301 21.717 1.00 0.00 C
ATOM 64 CB LEU 9 24.451 23.846 21.506 1.00 0.00 C
ATOM 65 CG LEU 9 24.953 24.356 20.160 1.00 0.00 C
ATOM 66 CD1 LEU 9 24.893 25.880 20.140 1.00 0.00 C
ATOM 67 CD2 LEU 9 26.393 23.901 19.948 1.00 0.00 C
ATOM 68 C LEU 9 22.179 23.764 20.561 1.00 0.00 C
ATOM 69 O LEU 9 21.836 24.507 19.643 1.00 0.00 O
ATOM 70 N MET 10 21.856 22.470 20.606 1.00 0.00 N
ATOM 71 CA MET 10 21.067 21.841 19.566 1.00 0.00 C
ATOM 72 CB MET 10 20.844 20.363 19.865 1.00 0.00 C
ATOM 73 CG MET 10 20.013 19.738 18.749 1.00 0.00 C
ATOM 74 SD MET 10 19.739 17.982 19.089 1.00 0.00 S
ATOM 75 CE MET 10 18.772 17.537 17.663 1.00 0.00 C
ATOM 76 C MET 10 19.702 22.501 19.437 1.00 0.00 C
ATOM 77 O MET 10 19.244 22.778 18.331 1.00 0.00 O
ATOM 78 N PHE 11 19.050 22.753 20.575 1.00 0.00 N
ATOM 79 CA PHE 11 17.744 23.378 20.587 1.00 0.00 C
ATOM 80 CB PHE 11 17.230 23.549 22.013 1.00 0.00 C
ATOM 81 CG PHE 11 15.869 24.193 22.127 1.00 0.00 C
ATOM 82 CD1 PHE 11 15.297 24.402 23.388 1.00 0.00 C
ATOM 83 CE1 PHE 11 14.036 25.000 23.495 1.00 0.00 C
ATOM 84 CZ PHE 11 13.346 25.390 22.340 1.00 0.00 C
ATOM 85 CE2 PHE 11 13.918 25.182 21.080 1.00 0.00 C
ATOM 86 CD2 PHE 11 15.180 24.584 20.973 1.00 0.00 C
ATOM 87 C PHE 11 17.789 24.754 19.940 1.00 0.00 C
ATOM 88 O PHE 11 16.922 25.097 19.140 1.00 0.00 O
ATOM 89 N LYS 12 18.805 25.547 20.291 1.00 0.00 N
ATOM 90 CA LYS 12 18.959 26.881 19.745 1.00 0.00 C
ATOM 91 CB LYS 12 20.199 27.565 20.312 1.00 0.00 C
ATOM 92 CG LYS 12 20.326 28.962 19.714 1.00 0.00 C
ATOM 93 CD LYS 12 21.565 29.647 20.280 1.00 0.00 C
ATOM 94 CE LYS 12 21.691 31.044 19.684 1.00 0.00 C
ATOM 95 NZ LYS 12 22.886 31.704 20.230 1.00 0.00 N1+
ATOM 96 C LYS 12 19.101 26.840 18.230 1.00 0.00 C
ATOM 97 O LYS 12 18.475 27.626 17.523 1.00 0.00 O
ATOM 98 N THR 13 19.930 25.918 17.732 1.00 0.00 N
ATOM 99 CA THR 13 20.151 25.778 16.307 1.00 0.00 C
ATOM 100 CB THR 13 21.138 24.654 16.013 1.00 0.00 C
ATOM 101 CG2 THR 13 22.472 24.960 16.687 1.00 0.00 C
ATOM 102 OG1 THR 13 20.621 23.421 16.519 1.00 0.00 O
ATOM 103 C THR 13 18.853 25.459 15.579 1.00 0.00 C
ATOM 104 O THR 13 18.561 26.044 14.538 1.00 0.00 O
ATOM 105 N GLU 14 18.071 24.528 16.132 1.00 0.00 N
ATOM 106 CA GLU 14 16.810 24.135 15.536 1.00 0.00 C
ATOM 107 CB GLU 14 16.122 23.057 16.367 1.00 0.00 C
ATOM 108 CG GLU 14 14.814 22.677 15.715 1.00 0.00 C
ATOM 109 CD GLU 14 14.126 21.597 16.547 1.00 0.00 C
ATOM 110 OE1 GLU 14 13.340 21.983 17.453 1.00 0.00 O
ATOM 111 OE2 GLU 14 14.393 20.398 16.270 1.00 0.00 O1-
ATOM 112 C GLU 14 15.861 25.320 15.433 1.00 0.00 C
ATOM 113 O GLU 14 16.204 26.431 15.829 1.00 0.00 O
ATOM 114 N NHE 15 14.662 25.088 14.899 1.00 0.00 N
TER 115 NHE 15
```
